# Supplementary material for: A versatile regulatory toolkit of arabinose-inducible artificial transcription factors for Enterobacteriaceae
Source: Commun Biol. 2023 Oct 3;6:1005. doi: 10.1038/s42003-023-05363-3 (PMC10547716; doi:10.1038/s42003-023-05363-3)
Supplement: Supplementary file 2 — Supplementary Information [file 42003_2023_5363_MOESM2_ESM.pdf]

Supplementary Information

Supplementary Figures

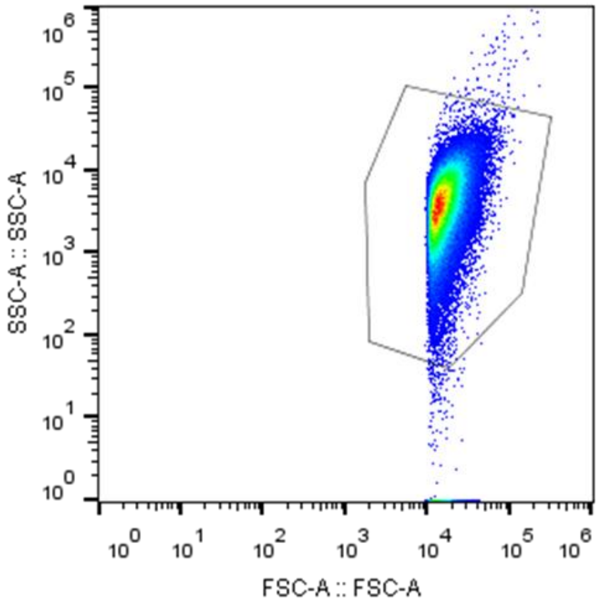

Supplementary Figure 1. Gating strategy. Bacterial populations were identified by plotting SSC vs. FSC on a log scale. The gated population (in the outlined area) was used for all subsequent measurements of fluorescence. Abbreviations: SSC, side scatter; FSC, forward scatter.

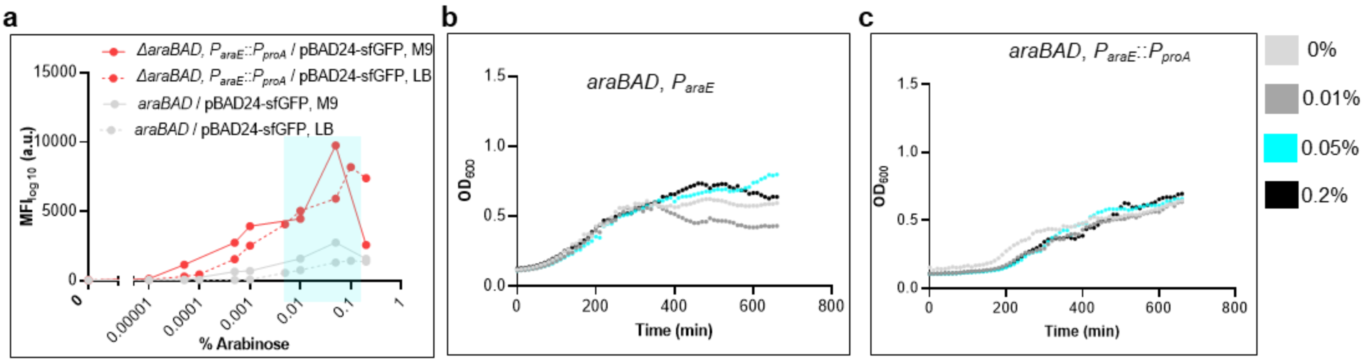

Supplementary Fig. 2. Characterization of arabinose induction system of *Salmonella* in M9 minimal medium. a Characterizing dose-dependency of arabinose-based gene expression from a plasmid in *Salmonella* in M9 minimal medium. The medium was supplemented with 0.4% glycerol and 0.1% casamino acids. Grey, wild-type; red, *Salmonella enterica* mutant with *araBAD* deletion and decoupled arabinose-dependent transporter-reporter system. As a reporter, sfGFP, under the control of the P_{BAD} promoter, encoded on plasmid pBAD24-sfGFP was used. sfGFP-expression was measured by flow cytometry in the presence of 0.00001%, 0.00005%, 0.0001%, 0.0005%, 0.001%, 0.005%, 0.01%, 0.05%, 0.1%, and 0.2% arabinose. Each data point of represents the mean of fluorescent intensity for 10,000 cells per sample. The 'induction window' is highlighted in blue. Growth curve of wild-type cells expressing *araE* using (**b**) its native promoter P_{araE}, and (**c**) *araBAD*-mutated background, expressing *araE* using P_{proA} in the absence (0%) and

presence of different arabinose concentrations, including 0.01%, 0.05%, and 0.2%. Abbreviations: a.u., arbitrary units; P_{BAD}, arabinose-responsive promoter; OD_{600}, optical intensity at 600 nm. Data are the average obtained from three independent colonies. The full data are shown in Supplementary Data 2.

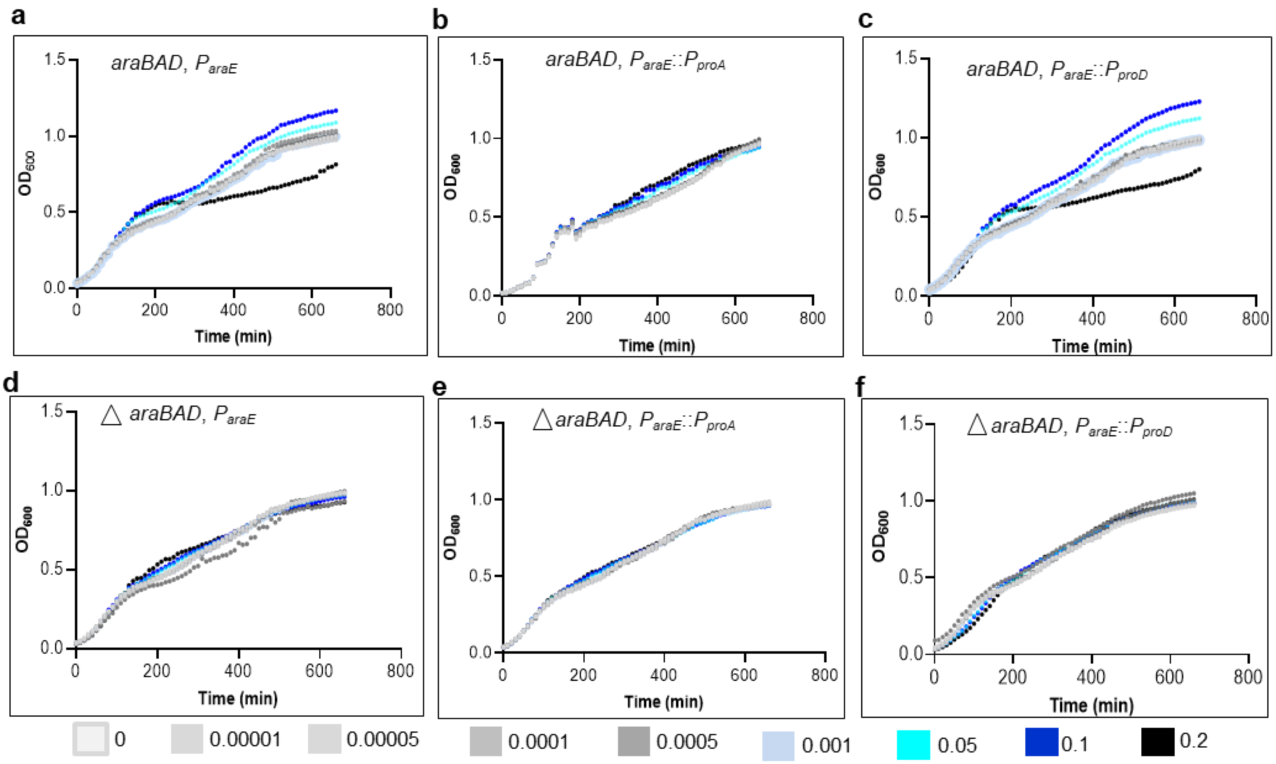

Supplementary Fig. 3. Time-course performance of *Salmonella* with tuned *araE* expression level treated with various arabinose concentrations in LB medium. The growth curve of wild-type *Salmonella enterica* cells expressing *araE* using (**a**) its native promoter P_{araE}, (**b**) arabinose-independent promoters P_{proA} and ((**c**) P_{proD}, *araBAD*-mutated background, expressing araE using promoters (**d**) P_{araE}, (**e**) P_{proA}, and (**f**) P_{proD} in absence and presence of different arabinose concentrations 0.00001%, 0.00005%, 0.0001%, 0005%, 0.001%, 0.05%, 0.1%, and 0.2%. Abbreviations: P_{BAD}, arabinose-responsive promoter; OD_{600}, optical intensity at 600 nm. Data are the average obtained from three independent colonies. The full data are shown in Supplementary Data 3.

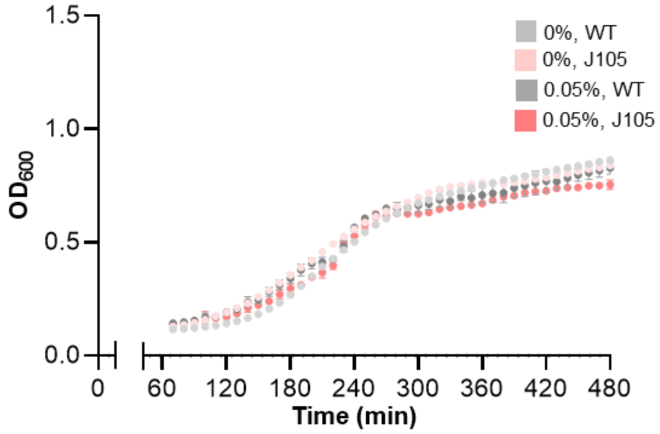

Supplementary Fig. 4. Time-course performance of CRISPR/dCas9-derived ATFs targeting J105 in *Salmonella*. *Salmonella enterica* cells harbor chromosomally-integrated arabinose-inducible dCas9, plasmid-encoded MCP-linker-SoxS(R93A), J105-targeting crRNA and *J1*-derived synthetic promoter[1] to control mRFP1 expression. Light grey, WT in non-inducing medium (LB medium without arabinose); dark grey, WT in inducing medium (0.05% arabinose added to LB medium), light red, CRISPR/dCas9-derived ATFs targeting J105 in non-inducing medium (LB medium without arabinose); dark red, CRISPR/dCas9-derived ATFs targeting J105 in inducing medium (0.05% arabinose added to LB medium). Abbreviations: dCas9, catalytically inactive Cas9; mRFP1, monomeric red fluorescent protein 1, OD_{600}, optical intensity at 600 nm; WT, wild-type harboring *araBAD* and expressing *araE* using P_{araE}. Data are the average obtained from three independent colonies. Full data is shown in Supplementary Data 5.

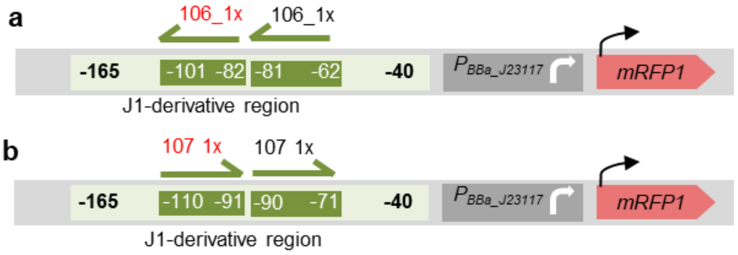

Supplementary Fig. 5. *J1*-derived synthetic promoter harboring two copies of gRNA binding site. The *J1* synthetic promoter[1] has potential crRNA binding sites with an appropriately positioned PAM sequence every ten bases on both sense and antisense strands upstream of a weak BBa_J23117 minimal promoter driving the mRFP1 reporter gene. **a** J106_2x harbors second copy of the J106 binding site was placed within *J1* synthetic region in antisense, resulting in effective CRISPR/dCas9 gene activation. **b** J107_2x carries a second copy of the J107 target site placed within the *J1* synthetic region in the sense strand, resulting in effective CRISPR/dCas9 gene activation. The expressed ATF targets a *J1*-derived synthetic promoter upstream of the mRFP1 reporter gene. Abbreviations: dCas9, catalytically inactive Cas9; mRFP1, monomeric red fluorescent protein 1, PAM, protospacer adjacent motif.

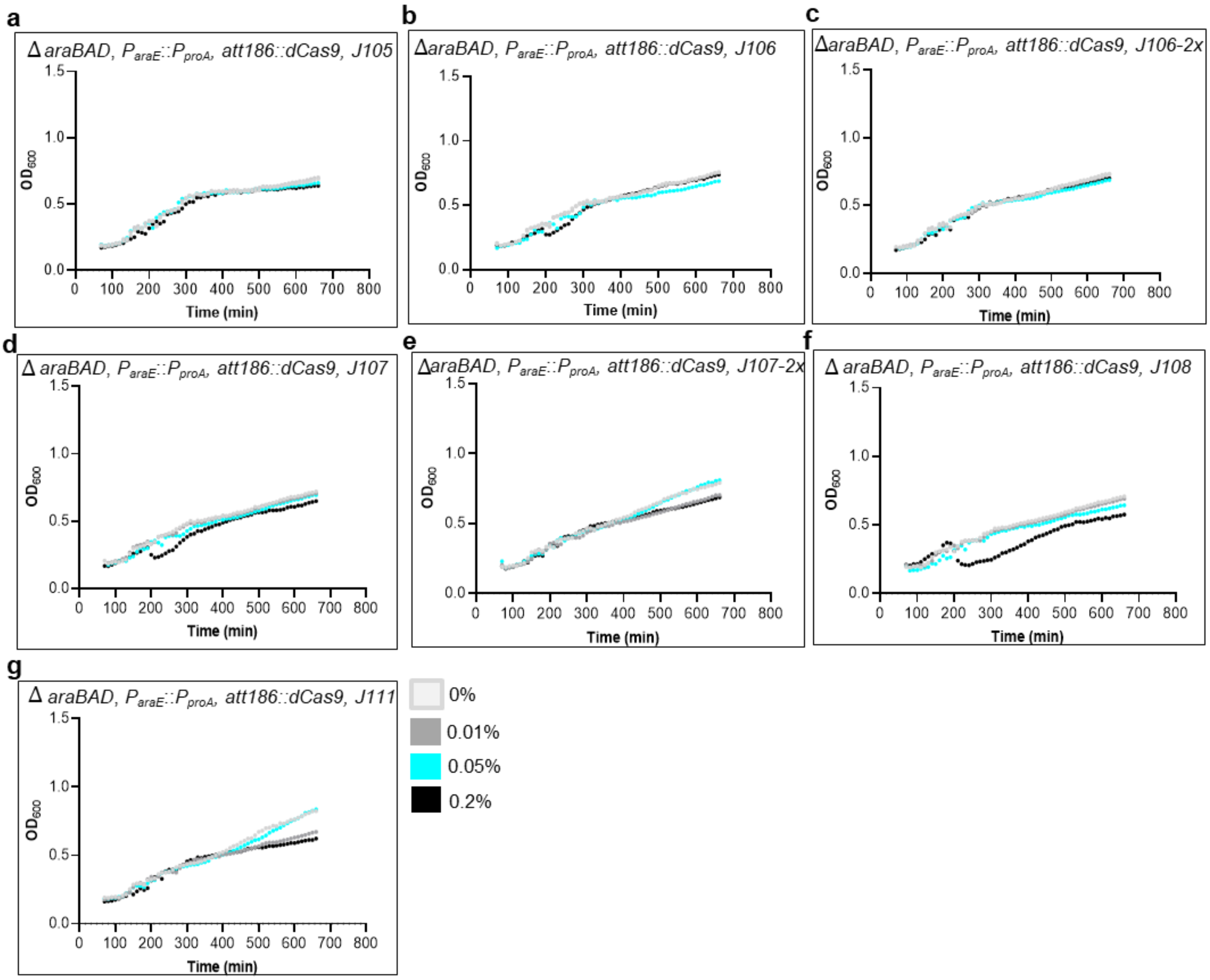

Supplementary Fig. 6. **Time-course performance of CRISPR/dCas9-derived ATFs in *Salmonella* rewired for arabinose catabolism**. *Salmonella enterica* background with deleted *araBAD* and P_{proA}-derived *araE* transporter expression was engineered to harbor chromosomally-integrated dCas9, plasmid-encoded MCP-linker-SoxS(R93A), J1-derived synthetic promoter to control mRFP1 expression and gRNAs targeting J105 (**a**), J106 (**b**), J106-2x (**c**), J107 (**d**), J107-2x (**e**), J108 (**f**), and J111 (**g**). The growth curve of cells was measured in the absence and presence of different arabinose concentrations, including 0.01%, 0.05%, and 0.2% added to M9 minimal medium (supplemented with 0.4% glycerol and 0.1% casamino acids). Abbreviations: P_{BAD}, arabinose-responsive promoter; OD_{600}, optical intensity at 600 nm. Data are the average obtained from three independent colonies. The full data are shown in Supplementary Data 6.

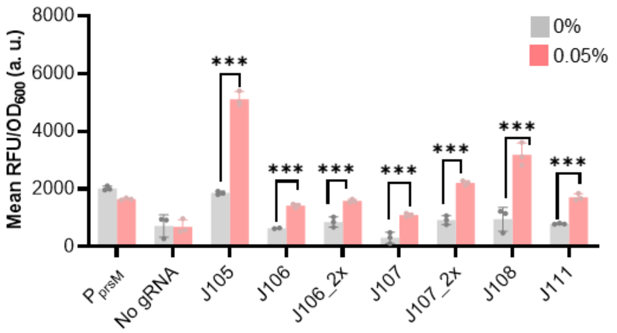

Supplementary Fig. 7. **Library of arabinose-inducible, CRISPR/dCas9-derived ATFs in *Salmonella*.** Arabinose-inducible CRISPR/Cas9-derived ATF was integrated into the genome of *Salmonella enterica*. Next, a plasmid expressing MCP-linker-SoxS(R93A), J1-derived synthetic promoter to control mRFP1 expression and gRNAs targeting *J105*, *J106*, *J106-2x*, J107, *J107-2x*, *J108*, or *J111* was transformed into the cell. Fluorescence output was measured after 4h. Grey, non-inducing medium; red, inducing medium. Data are expressed as the mean ± SD of the RFU obtained from three biological replicates, normalized to the OD_{600}. Asterisks indicate a statistically significant difference from the non-inducing medium (two-sided *t*-test, $***p \leq 0.001$). Abbreviations: a. u., arbitrary units; gRNA, guide RNA; mRFP1, monomeric red fluorescent protein; OD_{600}, optical intensity at 600 nm; RFU, relative fluorescent unit. LB medium was used, and for induction, 0.05% arabinose was added to the medium. The full data are shown in Supplementary Data 7.

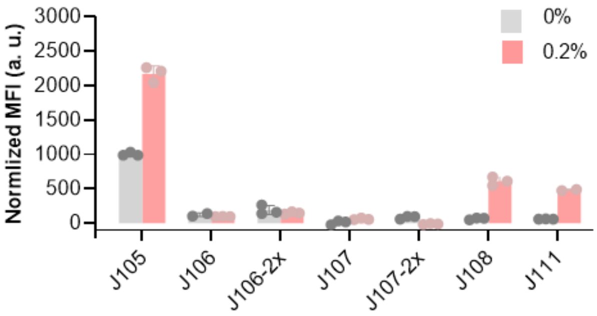

Supplementary Fig. 8. **Arabinose-inducible, CRISPR/dCas9-derived ATF for tunable gene expression in *E. coli***. Arabinose-inducible CRISPR/Cas9-derived ATF was integrated into the genome of *Escherichia coli*. Next, a plasmid expressing MCP-linker-SoxS(R93A), J1-derived synthetic promoter to control mRFP1 expression and gRNAs targeting *J105* or *J108* was transformed into the cell. Values reported are mRFP1 fluorescence levels normalized to that of wild-type *E. coli*. Grey, non-inducing medium; red, inducing medium. Data are expressed as the mean ± SD of the MFI obtained from three independent colonies, normalized to WT *E. coli* 10β. LB medium was used, and 0.2% arabinose was added to the medium for 4h induction. Abbreviations: a. u., arbitrary units; gRNA, guide RNA; mRFP1, monomeric red fluorescent protein; MFI, mean fluorescent intensity; OD_{600}, optical intensity at 600 nm. Asterisks indicate a

statistically significant difference from the non-inducing medium (two-sided *t*-test; ∗∗∗$p \leq 0.001$, ∗∗∗∗$p \leq 0.0001$). The full data are shown in Supplementary Data 8.

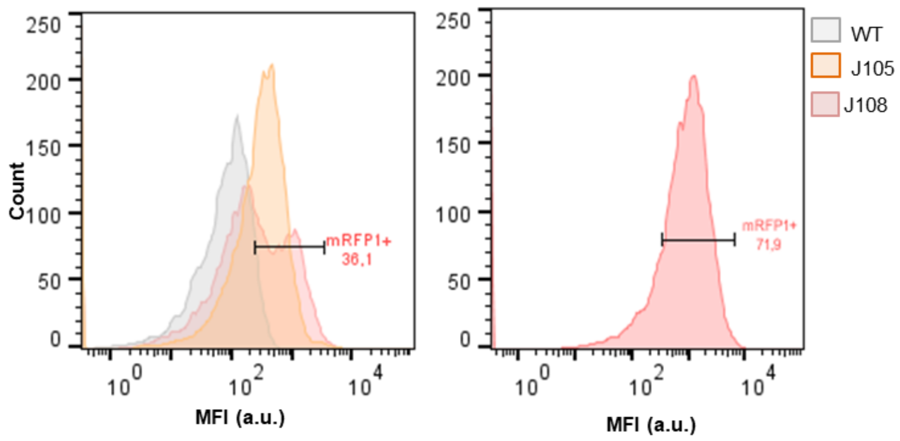

Supplementary Fig. 9. Histogram of mRFP1 fluorescence of *E. coli*. Arabinose-inducible CRISPR/Cas9-derived ATF was integrated into the genome of *Escherichia coli*. Next, a plasmid expressing MCP-linker-SoxS(R93A), *J1*-derived synthetic promoter to control mRFP1 expression and gRNAs targeting *J105*, *or J108* was transformed into the cell. Histogram of mRFP1 fluorescence was measured in inducing LB medium after 4h (left) and inducing M9 minimal medium (supplemented 0.4% glycerol and 0.1% casamino acids) after 8h (right). For induction, 0.2% arabinose was added to the medium. Abbreviations: a. u., arbitrary units; gRNA, guide RNA; mRFP1, monomeric red fluorescent protein; MFI, mean fluorescent intensity; WT, wild type.

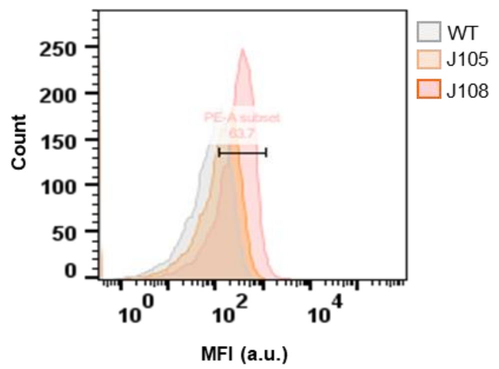

Supplementary Fig. 10. Fluorescence distribution of an mRFP1-expressing *Salmonella* strain. Arabinose-inducible CRISPR/Cas9-derived ATF was integrated into the genome of *Salmonella enterica*. Next, a plasmid expressing MCP-linker-SoxS(R93A), J1-derived synthetic promoter to control mRFP1 expression and gRNAs targeting *J105* or *J108* was transformed into the cell. The fluorescence histogram of mRFP1 was measured in an inducing medium after 4h. For induction, 0.05% arabinose was added to the LB medium. Abbreviations: a. u., arbitrary units; gRNA, guide RNA; mRFP1, monomeric red fluorescent protein; MFI, mean fluorescent intensity; WT, wild type.

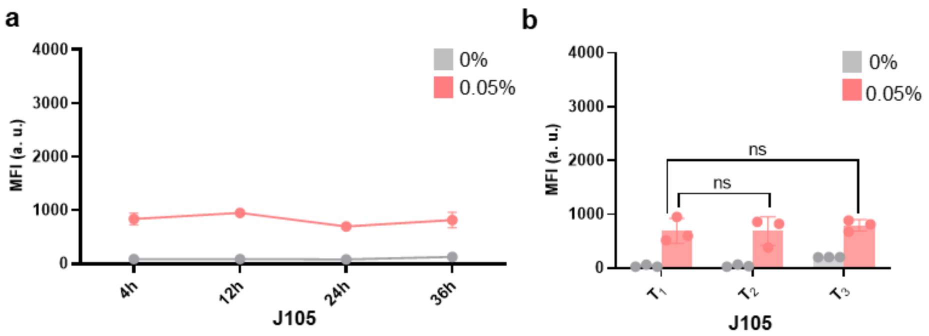

Supplementary Figure 11. **Stability of reporter gene expression controlled by arabinose-inducible, CRISPR/dCas9-derived ATF in *Salmonella***. **a** Time course of fluorescent reporter expression controlled by arabinose-inducible CRISPR/dCas9-derived ATF targeting *J105* in *Salmonella*. The fluorescent output of mRFP1 was measured by flow cytometry after 4h, 12h, 24h, and 36h. **b** Stability of fluorescent reporter expression controlled by arabinose-inducible, CRISPR/dCas9-derived ATF targeting *J105* in *Salmonella*. The fluorescent output of mRFP1 was measured by flow cytometry for three subsequent subcultures after 4h. In both cases, arabinose-inducible CRISPR/Cas9-derived ATF was integrated into the genome of *Salmonella enterica*. Next, a plasmid expressing MCP-linker-SoxS(R93A), J1-derived synthetic promoter to control mRFP1 expression and gRNAs targeting gRNA targeting *J105* was transformed into the cell. Grey, non-inducing medium; green, inducing medium. M9 minimal medium (supplemented with 0.4% glycerol and 0.1% casamino acids) was used, and for induction, 0.05% arabinose was added to the medium. Data are expressed as the mean ± SD of the MFI obtained from three biological replicates. Two-sided *t*-test was performed. *ns* indicates no statistically significant difference from the T_1, using a two-sided *t*-test. Abbreviations: a. u., arbitrary units; mRFP1, monomeric red fluorescent protein; MFI, mean fluorescent intensity. The full data are shown in Supplementary Data 12.

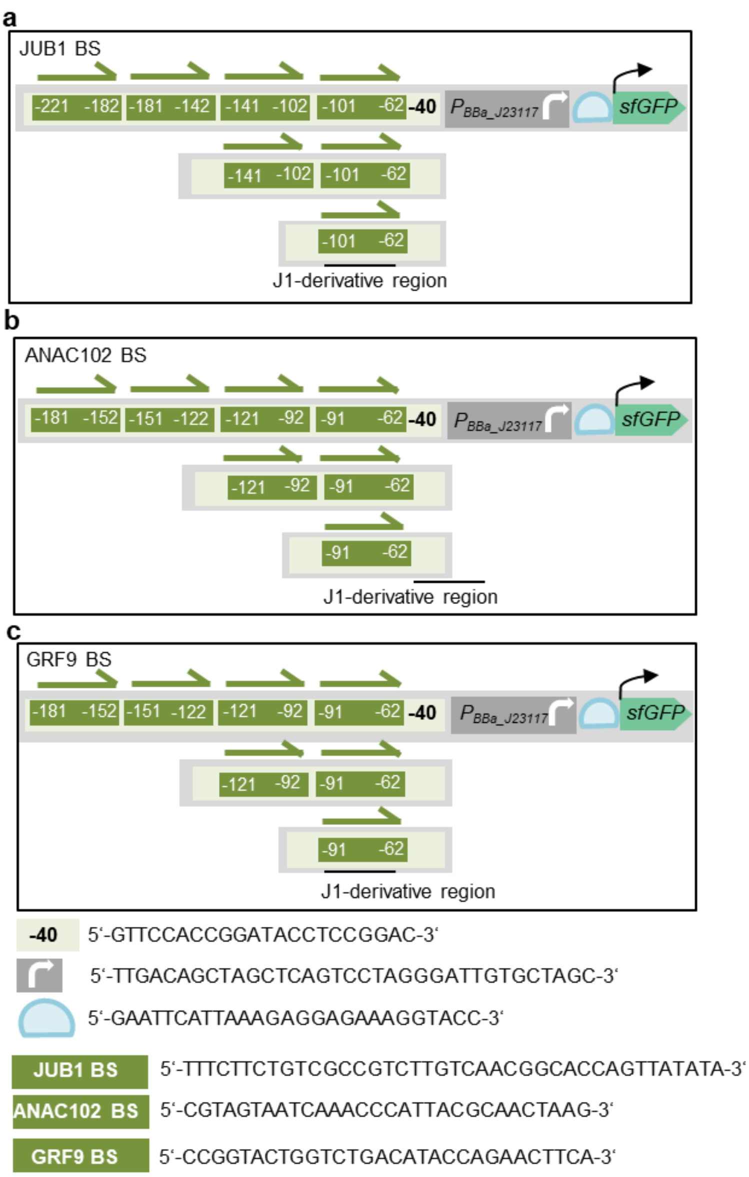

Supplementary Figure 12. **Synthetic promoter harboring binding site of plant TF**. **a** JUB1-, **b** ANAC102-, and **c** GRF9-synthetic promoters. The design principle of Dong *et al*.[1] is implemented to include the first copy of the plant TF binding site between TSS and -80 on the sense strand -62 to -101 for JUB1, -62 to -91 for ANAC102 and GRF9. The second copy of the plant TF binding site was added upstream of the first TF binding site according to the design principle described by Ho *et al*.[2] between -81 and -118 to achieve the high transcriptional output (-102 to -141 for JUB1, -92 to -121 for ANAC102 and GRF9). The expressed ATF targets a *J1*-derived synthetic promoter upstream of the sfGFP reporter gene. Abbreviations: ANAC102, NAC TF 102; BS, binding site; GRF9, growth regulatory factor; JUB1, JUNGBRUNNEN1; sfGFP, super-folder green fluorescent protein; TF, transcription factor; TSS, transcriptional start site.

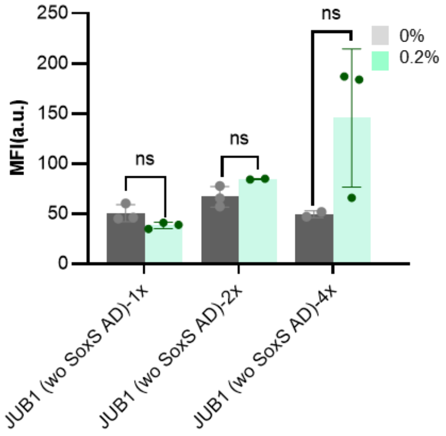

Supplementary Figure 13. **The transcriptional activity of JUB1 TF in *E. coli***. Expression plasmid encoding full-length JUB1 TF and reporter plasmid harboring synthetic promoter upstream of sfGFP were co-transformed into the *Escherichia coli* cell. The transactivation capacity in combination with one (1x), two (2x), or four (4x) copies of BS within synthetic promoter was tested in the non-inducing and inducing M9 minimal medium (supplemented with 0.4% glycerol and 0.1% casamino acids) after 8h. For induction, 0.2% arabinose was used. Two-sided *t*-test was performed (ns, not significant). Abbreviations: AD, activation domain; a. u., arbitrary units; BS, binding site; JUB1, JUNGBRUNNEN1; MFI, mean fluorescence intensity; sfGFP, super-folder green fluorescent protein; TF, transcription factor. The full data are shown in Supplementary Data 10.

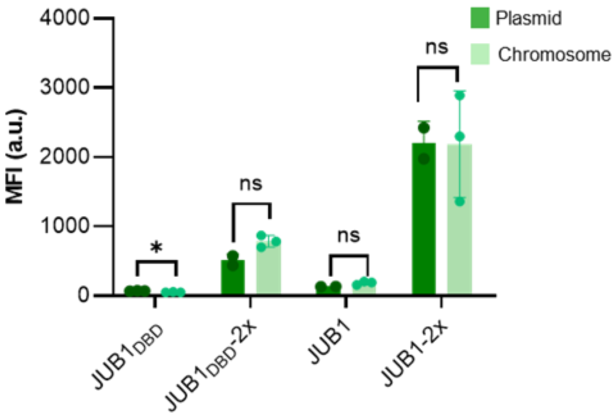

Supplementary Figure 14. Arabinose-inducible, JUB1$_{DBD}$-derived ATFs expressed from plasmid vs. chromosome in *E. coli* in LB. ATF derived from full-length plant JUB1 or only its DBD, fused to SoxS(R93A) via a linker, was chromosomally integrated at the *att186* site (dark green) of *Escherichia coli*, or expression plasmids harboring full-length plant JUB1 or only its DBD-derived ATF was introduced into the cell with reporter cassette (light green). The reporter plasmid harbors a synthetic promoter placed upstream of sfGFP. The ATF transactivation capacity in combination with two copies (2x) of BS within synthetic promoter was tested in the inducing medium after 4h induction. The induction medium contains 0.2% arabinose added to the

LB medium. The strains harboring expression cassette but no reporter cassette was used as a negative control. Two-sided *t*-test was performed (ns, not significant). Abbreviations: ATF, artificial transcription factor; a. u., arbitrary units; BS, binding site; JUB1, JUNGBRUNNEN1; MFI, mean fluorescence intensity; sfGFP, super-folder green fluorescent protein. The full data are shown in Supplementary Data 12.

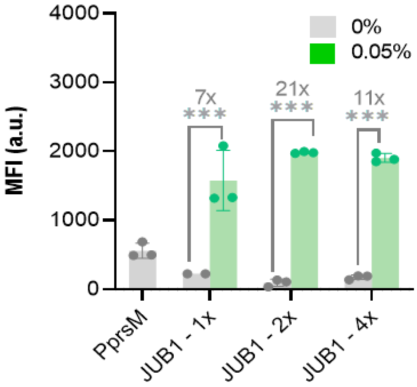

Supplementary Figure 15. Arabinose-inducible, JUB1-derived ATF in *Salmonella* in LB medium. Expression plasmid encoding full-length JUB1-derived ATF fused to SoxS(R93A) via a linker and reporter plasmid harboring synthetic promoter upstream of sfGFP reporter were co-transformed into the *Salmonella enterica* cell. The transactivation capacity of JUB1-derived ATFs in combination with one (1x), two (2x), or four (4x) copies of BS was tested in a wild-type background in the absence of arabinose (grey), and the presence of arabinose (green) after 4h (induction). Constitutive promoter P_{prsM} was used as control. Data are expressed as the mean ± SD of the MFI obtained from three biological replicates. "*x*" top of the columns represents the fold induction compared to the non-inducing medium. Asterisks indicate a statistically significant difference (two-sided *t*-test; ns, not significant; $*p \leq 0.05$; $***p \leq 0.001$). For induction, 0.05% arabinose was added to the LB medium. Abbreviations: ATF, artificial transcription factor; a. u., arbitrary units; BS, binding site; JUB1, JUNGBRUNNEN1; MFI, mean fluorescence intensity; sfGFP, super-folder green fluorescent protein. The full data are shown in Supplementary Data 13.

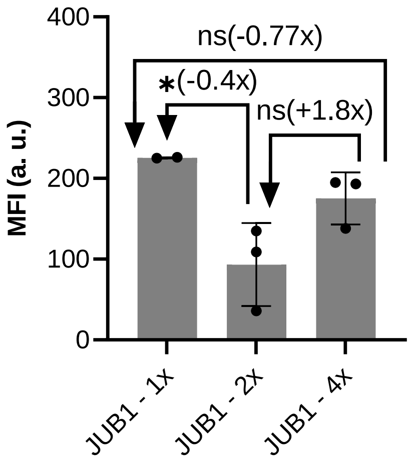

Supplementary Figure 16. **Basal expression of plant-derived ATF used for tunable gene expression in *E. coli***. Plasmids harboring full-length JUB1-derived ATF fused to SoxS(R93A) via a linker and reporter cassettes were co-transformed into the *Escherichia coli* cell. The transactivation capacity of plasmid-encoded JUB1-derived ATFs in combination with one (1x), two (2x), or four (4x) copies of BS was tested in a wild-type background in the M9 minimal medium (supplemented with 0.4% glycerol and 0.1% casamino acids) without arabinose after 4h. Data are expressed as the mean ± SD of the MFI obtained from three biological replicates. "*x*" top of the columns represents the fold induction compared to the non-inducing medium. Asterisks indicate a statistically significant difference (two-sided *t*-test; ns, not significant; ∗p ≤ 0.05). Abbreviations: ATF, artificial transcription factor; a. u., arbitrary units; BS, binding site; JUB1, JUNGBRUNNEN1; MFI, mean fluorescence intensity; sfGFP, super-folder green fluorescent protein. The full data are shown in Supplementary Data 14.

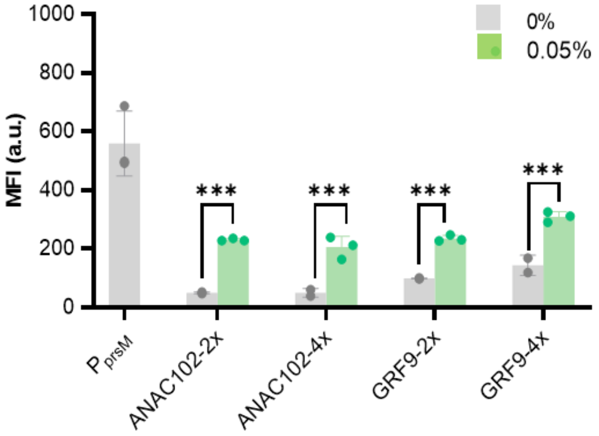

Supplementary Fig. 17. Arabinose-inducible ATFs derived from plant ANAC102 and GRF9 TFs for *Salmonella*. Expression plasmid encoding full-length plant-derived ATF fused to SoxS(R93A) via a linker and reporter plasmid harboring synthetic promoter upstream of sfGFP reporter were co-transformed into the *Salmonella enterica* cell. The transactivation capacity of

ANAC102 and GRF9-derived ATFs were tested against the two (2x) and four (4x) copies of its binding site driving sfGFP reporter expression. Fluorescence output is measured in the absence (grey) and presence of 0.05% arabinose added to LB medium (light green) after 4h (induction). P_{prsM}, positive control. The MFI of each sample was calculated via FlowJo. Data are expressed as the mean ± SD of the MFI obtained from three independent colonies. Asterisks indicate a statistically significant difference from the non-inducing medium (two-sided *t*-test; $***p \leq 0.001$). Abbreviations: ATF, artificial transcription factor; a. u., arbitrary units; BS, binding site; ANAC102, NAC TF 102; GRF9, growth regulatory factor; sfGFP, super-folder green fluorescent protein. The full data are shown in Supplementary Data 15.

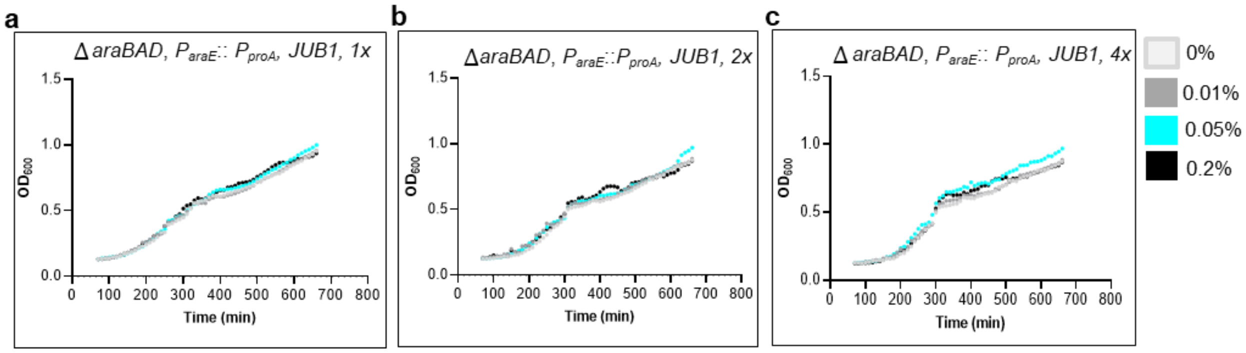

Supplementary Fig. 18. **Time-course performance of plant-derived ATFs in *Salmonella* optimized for arabinose catabolism and transport**. *Salmonella enterica* background with deleted *araBAD* and P_{proA}-derived *araE* transporter expression was co-transformed expression plasmid encoding JUB1-derived ATF fused to SoxS(R93A) via a linker and reporter plasmid harboring synthetic promoter with one (**a**), two (**b**), or four (**c**) copies of its binding site driving sfGFP reporter expression. The growth curve of cells was measured in the absence and presence of different arabinose concentrations, including 0.01%, 0.05%, and 0.2% added to M9 minimal medium (supplemented with 0.4% glycerol and 0.1% casamino acids). Data are expressed as the mean of the OD_{600} obtained from three biological replicates. Abbreviations: BS, binding site; JUB1, JUNGBRUNNEN1; OD_{600}, optical intensity at 600 nm. sfGFP, super-folder green fluorescent protein. Data are the average obtained from three independent colonies. The full data are shown in Supplementary Data 16.

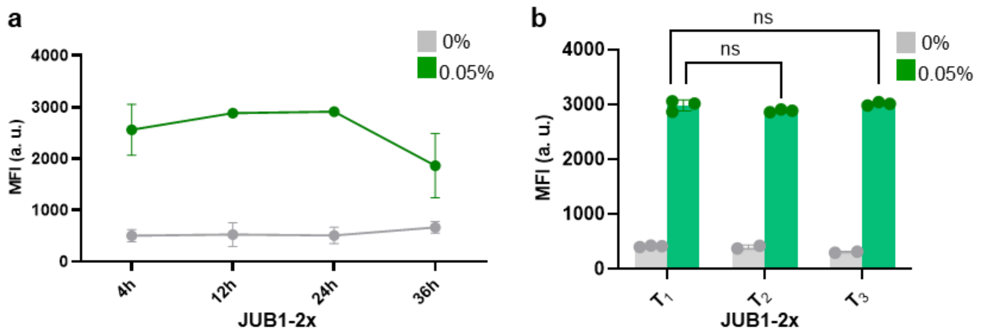

Supplementary Fig. 19. **Stability of reporter gene expression controlled by arabinose-inducible JUB1-derived ATF in *Salmonella***. Expression plasmid encoding full-length JUB1-derived ATF fused to SoxS(R93A) via a linker and reporter plasmid harboring two copies of JUB1 binding site within a synthetic promoter upstream of sfGFP reporter were co-transformed into *Salmonella enterica* cells. **a** Time course of fluorescent reporter expression controlled by arabinose-inducible JUB1-derived ATF in *Salmonella*. The fluorescent output of sfGFP was measured by flow cytometry after 4h, 12h, 24h, and 36h. **b** Stability of fluorescent reporter expression controlled by arabinose-inducible JUB1-derived ATF in *Salmonella*. The fluorescent output of sfGFP was measured by flow cytometry for three subsequent subcultures after 4h. Grey, non-inducing medium; green, inducing medium. M9 minimal medium, supplemented with 0.4% glycerol and 0.1% casamino acids, was used. For induction, 0.2% arabinose was used. Data are expressed as the mean ± SD of the MFI obtained from three biological replicates. *ns* indicates no statistically significant difference from the T_1, using a two-paired *t*-test (ns, not significant). Abbreviations: a. u., arbitrary units; BS, binding site; JUB1, JUNGBRUNNEN1; MFI, mean fluorescent intensity; sfGFP, super-folder green fluorescent protein. The full data are shown in Supplementary Data 17.

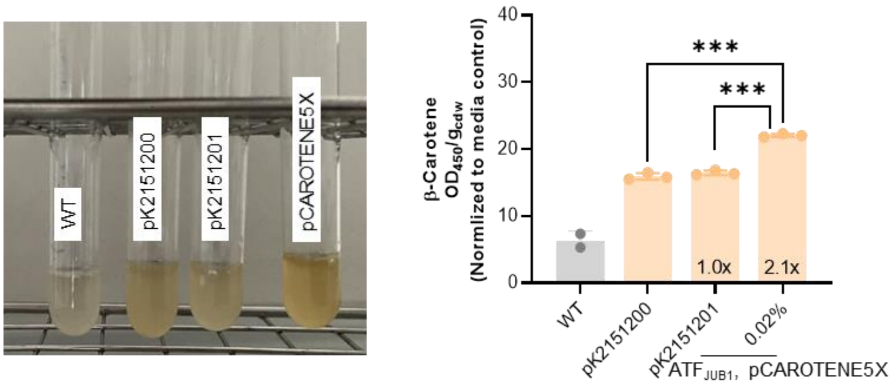

Supplementary Fig. 20. **Production of β-carotene in *E. coli* strains after 4h**. The JUB1-derived ATF donor was integrated into the *att186* site of *Escherichia coli*. Followed by transformation of pCAROTEN5X that harbors five copies of JUB1 BS upstream of an artificial operon encoding biosynthetic pathway genes, β-carotene production was quantified in the presence of 0.2% arabinose (WT/pCAROTENE5X) added to LB medium after 4h (induction).

Representative cultures of the constructed β-carotene producing strains were shown on the left. The β-carotene absorbance at 450 nm was measured using the method reported by Lian *et al*[3], and was divided to cdw. The data were normalized to that of the medium. Values represent the mean ± SD of three independent colonies in the presence of 0.2% arabinose. *E. coli* strains containing pK2151200 (WT/pK2151200) and pK2151201 (WT/pK2151201) were used as controls. Asterisks indicate a statistically significant difference (*t*-test; ∗∗∗$p \leq 0.001$). "*x*" inside the columns represents the fold induction compared to pK2151201. Abbreviations: ATF, artificial transcription factor; BS, binding site; cdw, cell dry weight; JUB1, NAC TF J UNGBRUNNEN1; WT, wild-type. The full data are shown in Supplementary Data 20.

Supplementary Methods

Construction of plasmids and strains

Construction of S. Typhimurium *araBAD background strains*

EM12441 (*ΔaraBAD*::*sfGFP*): sfGFP together with a downstream terminator and 40-bp overhangs homologous to the regions up- and downstream of the *araBAD* operon was PCR-amplified from the plasmid pEM8317 (lab collection) using primers DaraBAD-sfGFP-rev and DaraBAD-sfGFP-fv. The PCR product was introduced by electroporation into a (S. Typhimurium LT2) strain with a *tetRA* cassette replacing the araBAD operon and harboring the λ-RED-helper plasmid pKD46 (TH6706)[4] to facilitate λ red-mediated replacement of tetRA with sfGFP. Successful recombinants were selected for tetracycline-sensitivity (TcS) using TcS plates[5].

EM12709 (*ΔaraBAD*::*sfGFP*, P_{araE}::P_{proA}): Strain EM12441 (generated in this study) was transformed with pKD46[4]. In the generated strain EM12472, λ red-mediated recombineering was used to replace P_{araE} first with the *tetRA* expression module. To do this, EM12472 was transformed with PCR-amplified *tetRA* (ParaE-tetRA-fv/ParaE-tetRA-rev on genomic DNA of TH3730, Kelly T. Hughes). Followed by selection on 15 µg/ml tetracycline plate, the generated strain was named EM12498. Next, to subsequently replace *tetRA* with P_{proA}, PCR-amplified P_{proA} (primers PproX-araE-fv/PproX-araE-rev on genomic DNA of strain EM9661, lab collection) was transformed into the strain EM12498 to be replaced with t*etRA* (using λ red-mediated recombineering). The generated strain was named EM12709.

EM12746 (*araBAD*, P_{araE}::P_{proA}): The (native) *araBAD* operon of a *S.* Typhimurium wild-type strain (strain TH437, Kelly T. Hughes) was transferred to EM12709 (generated in this study) using bacteriophage P_{22}-mediated transduction. To screen the positive clone, selection was performed for the capability to use arabinose as the sole carbon source on a carbon-free plate supplemented with arabinose.

EM12886 (*araBAD*/pBAD24-sfGFP): Plasmid pBAD24-sfGFPx1 (Addgene, #51558) was transformed into wild-type *S.* Typhimurium LT2 (TH437, Kelly T. Hughes). To screen the positive clone, selection was performed for capability to use arabinose as the sole carbon source on a carbon-free plate supplemented with arabinose.

EM12877 (*ΔaraBAD*, P_{araE}::P_{proA}): The P_{araE}::P_{proA} fragment of EM12709 (generated in this study) was transferred into TH6701 in which the *araBAD* operon is replaced with *tetRA* (Kelly T. Hughes) using bacteriophage P_{22}-mediated transduction.

EM12887 (*ΔaraBAD*/pBAD24-sfGFP): Plasmid pBAD24-sfGFPx1 (Addgene, #51558) was introduced into *S.* Typhimurium LT2, in which the *araBAD* operon is replaced with *tetRA* (TH6701, Kelly T. Hughes).

EM12888 (P_{araE}::P_{proA}/pBAD24-sfGFP): Plasmid pBAD24-sfGFPx1 (Addgene, #51558) was introduced into strain EM12746 (generated in this study).

EM12889 (*ΔaraBAD*, P_{araE}::P_{proA}/pBAD24-sfGFP): Plasmid pBAD24-sfGFPx1 (Addgene, #51558) was introduced into strain EM12887 (generated in this study).

EM12967 (*araBAD*, P_{araE}::P_{proD}): PCR-amplified P_{proD} (primers PproX-araE-fv/PproX-araE-rev on genomic DNA of strain EM8513, lab collection) was introduced into strain EM12498 (generated in this study). Using λ red-mediated recombineering[6], *tetRA* was replaced with P_{proD} to construct strain EM12712. Next, the *araBAD* fragment was transferred from TH437 (Kelly T. Hughes) to EM12712 using P_{22} (Bacteriophage *S.* Typhimurium) transduction to generate the EM12967 strain.

EM13000 (*ΔaraBAD*, P_{araE}::P_{proD}): *ΔaraBAD925*::*tetRA* fragment of TH6701 was transferred into EM12967, carrying pKD46 (generated in this study) using λ red-mediated recombineering[6].

Expression plasmids for dCas9-derived ATFs

pOSIP-KO_PBAD-dCas9: The gBlock "N-$dCas9_{opt}$", containing N-terminal region of dCas9 codon-optimized for expression in *S.* Typhimurium LT2 ($dCas9_{opt}$) was synthesized by IDT (Dessau-Rosslau, Germany). The PCR-amplified "N-$dCas9_{opt}$" fragment (primer pair N-dCas9-fv/N-dCas9-rv, on "N-$dCas9_{opt}$") was cloned in *Asc*I/*Pme*I-digested pG0A0-1-1[7] using the NEBuilder HiFi DNA assembly strategy to generate "pG0A0-1-1_N-dCas9". The gBlock "C-$dCas9_{opt}$-T_{rrnBT1}", containing C-terminal of dCas9 that is codon-optimized for expression in *S.* Typhimurium ($dCas9_{opt}$), fused to bacterial *rrnBT1* terminator (T_{rrnBT1}), was synthesized by IDT

(Dessau-Rosslau, Germany). The PCR-amplified "C-*dCas9*$_{opt}$-*T*$_{rrnBT1}$" fragment (primer pair C-dCas9-fv/C-dCas9-rv on gBlock "C-*dCas9*$_{opt}$-*T*$_{rrnBT1}$") was cloned into *Xba*I/*Pme*I-digested pG0A0-1-1_N-dCas9 using the NEBuilder HiFi DNA assembly strategy to generate pG0A0-1-1_dCas9. PCR-amplified *P*$_{BAD}$ fused to a synthetic strong RBS (as described by Dong *et al*. (2018)[1] (primer LacI-AraC-ParaB-fv/LacI-AraC-ParaB-rv on "placI-araC-PBAD-RBS", ATG Bio-Synthesis) was cloned into *Sal*I-digested pG0A-0-1-1_dCas9 using the NEBuilder HiFi DNA assembly strategy to generate "pG0A-1-1_PBAD_dCas9". Next, the PCR-amplified "P$_{BAD}$-dCas9-*T*$_{rrnBT1}$" fragment (primer pair dCas9-fv/dCas9-rv on "pG0A-1-1_PBAD_dCas9") was cloned into *Bam*HI/*Spe*I-digested pOSIP-KO[8] to generate the "pOSIP-KO_PBAD-dCas9" plasmid.

Reporter plasmids for dCas9-derived ATFs

pJ105: A 1094-bp fragment containing crRNA to target the J106 motif within the J1 promoter, together with its leader and terminator sequences was PCR-amplified using primer pair BBa_J23119-gRNA-J106-fv/BBa_J23119-gRNA-J106-rv from pCK005.6 (Addgene, #153025)[1]. Subsequently, it was cloned into a PCR-amplified 1869 bp fragment of pUC19 (NEB, primer pair PUC19-fv/PUC19-rv) using the NEBuilder HiFi DNA assembly strategy to generate plasmid p106. Next, the J106-targeting crRNA region of plasmid p106 was replaced with crRNA to target the J105 motif within the *J1* region of the synthetic promoter[1]. To this end, the "p105" fragment was PCR-amplified (primer pair crRNA-J105-fv/crRNA-J105-rev on p106) and followed by NEBuilder HiFi DNA assembly-mediated circularisation of the PCR product, the plasmid p105 was generated. Subsequently, p105 was digested with *Acl*I and *Aat*II to remove a 690 bp fragment. The *J1* region of the synthetic promoter fused to mRFP1 and its downstream terminator was PCR-amplified (primer pair J1-RFP-fv/J1-RFP-rv on pJF076Sa, Addgene, #113322[1]) and cloned into the linearized p105 plasmid using the NEBuilder HiFi DNA assembly strategy. The generated plasmid was called "pJ105".

pSJ105: PCR-amplified P$_{tetR}$-tetR-T$_{tetR}$ fragment (primer pair TET-R-fv/TET-R-rv on genomic DNA TH3730, gift from Kelly T. Hughes) and PCR-amplified fragment containing coding regions for P$_{BAD}$, MCP optimized for expression in *S.* Typhimurium LT2 (optMCP), a five amino acid linker, SoxS activation domain optimized for expression in *S.* Typhimurium LT2 with enhanced transcriptional activity (SoxS(R93A)$_{opt}$), and synthetic T$_{BBa_B002}$ terminator (primer pair MCP-SOX-fv/MCP-SOX-rv on gBlock "pAra_optMCP_linker_optSOXA_R93A_BBa_B002")[1] were assembled in *Asc*I/*Bam*HI-digested high-copy AVA plasmid (harbouring origin of replication (ori) *pBR322*)[9] using the NEBuilder HiFi DNA assembly strategy. The generated plasmid was named "AVA_TetR_SOXS_a". Next, fragment "*P*$_{BAD}$-*optMCP-linker-optSOXS_R93A-BBa_T*$_{BBa-B002}$" (primer pair TET-MCP-SOX-fv /TET-MCP-SOX-rv on "AVA_TetR_SoxS_a") was assembled in

PCR-amplified backbone pJ105 (primer pair J-crRNA-fv/ J-crRNA-rv) to generate plasmid pSJ105.

pSJ106: Fragment "*P_{BAD}-optMCP-linker-optSOXS_R93A-BBa_$T_{BBa\text{-}B002}$*"(primer pair TET-MCP-SOX-fv /TET-MCP-SOX-rv on "AVA_TetR_SoxS_a") was assembled in PCR-amplified backbone pCK005.6[1] (primer pair J-crRNA-fv/ J-crRNA-rv) to generate plasmid pSoxS106. Plasmid pSoxS106 was digested with *Acl*I and *Aat*II to remove 690 bp. The J1-containing promoter fused to mRFP1, together with its downstream terminator was PCR-amplified (primer pair J1-RFP-fv/J1-RFP-rv on pJF076Sa, Addgene, #113322)[1] and cloned into the linearized pS-106 plasmid using the NEBuilder HiFi DNA assembly strategy. The generated plasmid was called pSJ106.

pSJ106-2x: A 2337-bp fragment harboring a modified *J1* region with two copies of the J-106 motif (see **Results** Section), mRFP1, and bacterial T_{rrnBT1} terminator (J1-RFP-2x-fv/J1-RFP-rv on "pUC57_106_2x", BioCat Inc., Heidelberg, Germany) were cloned into *Acl*I/*Aat*II-digested pSoxS-106 using the NEBuilder HiFi DNA assembly strategy to construct "pSJ106-2x" plasmid.

pSJ107: The J106-targeting crRNA region of plasmid p106 was replaced with crRNA to target J107 motif within the *J1*-containing promoter[1] using the NEBuilder HiFi DNA assembly of PCR-amplified fragment (primer pair crRNA-J107-fv/crRNA-J107-rev on p106) to construct p107. Fragment "*P_{BAD}-optMCP-linker-optSOXS_R93A-BBa_$T_{BBa\text{-}B002}$*"(primer pair TET-MCP-SOX-fv/TET-MCP-SOX-rv on "AVA_TetR_SOXS_a") was assembled in PCR-amplified backbone p107 (primer pair J-crRNA-fv/ J-crRNA-rv) to generate plasmid pSoxS107. Plasmid pSoxS107 was digested with *Acl*I and *Aat*II to remove 690 bp. The J1 region of the synthetic promoter fused to mRFP1, together with its downstream terminator was PCR-amplified (primer pair J1-RFP-fv/J1-RFP-rv on pJF076Sa, Addgene, #113322)[1] and cloned into the linearized pSoxS-107 plasmid using the NEBuilder HiFi DNA assembly strategy. The generated plasmid was called pSJ107.

pSJ107-2x: A 2337-bp fragment harboring modified *J1* region of the synthetic promoter with two copies of J-107 motif (see Results Section), mRFP1 and bacterial T_{rrnBT1} terminator (J1-RFP-2x-fv/J1-RFP-rv on "pUC57_107_2x", BioCat Inc., Heidelberg, Germany) were cloned into *Acl*I/*Aat*II-digested pSoxS--106 using the NEBuilder HiFi DNA assembly strategy to construct "pSJ107-2x" plasmid.

pSJ-108: The J106-targeting crRNA region of plasmid p106 was replaced with crRNA to target J108 motif within J1 promoter[1] using the NEBuilder HiFi DNA assembly of PCR-amplified fragment (primer pair crRNA-J108-fv/crRNA-J108-rev on p106) to construct p108. Fragment "*P_{BAD}-optMCP-linker-optSOXS_R93A-BBa_$T_{BBa\text{-}B002}$*" (primer pair TET-MCP-SOX-fv/TET-MCP-

SOX-rv on “AVA_TetR_SoxS_a”) was assembled in PCR-amplified backbone p108 (primer pair J-crRNA-fv/ JcrRNA-rv) to generate plasmid pSoxS--108. Plasmid pS-108 was digested with *Acl*I and *Aat*II to remove 690 bp. The *J1* region of synthetic promoter fused to mRFP1, and its downstream terminator was PCR-amplified (primer pair J1-RFP-fv/J1-RFP-rv on pJF076Sa, Addgene, #113322[1]) and cloned into the linearized pSoxS108 plasmid using the NEBuilder HiFi DNA assembly strategy. The generated plasmid was called pSJ108.

pSJ111: The J106-targeting crRNA region of plasmid p106 was replaced with crRNA to target J111 motif within the *J1* region of synthetic promoter[1] using the NEBuilder HiFi DNA assembly of PCR-amplified fragment (primer pair crRNA-J111-fv/crRNA-J111-rev on p106) to construct p111. Fragment “*P_{BAD}-optMCP-linker-optSOXS_R93A-BBa_$T_{BBa\text{-}B002}$*“ (primer pair TET-MCP-SOX-fv/TET-MCP-SOX-rv on “AVA_TetR_SOXS_a”) was assembled in PCR-amplified backbone p108 (primer pair J-crRNA-fv/ J-crRNA-rv) to generate plasmid pSoxS111. Plasmid pS-111 was digested with *Acl*I and *Aat*II to remove 690 bp. J1 promoter fused to mRFP1, and its downstream terminator was PCR-amplified (primer pair J1-RFP-fv/J1-RFP-rv on pJF076Sa, Addgene, #113322[1]) and cloned into the linearized pSoxS111 plasmid using the NEBuilder HiFi DNA assembly strategy. The generated plasmid was called “pSJ111”.

dCas9 driver/reporter strains

"E. coli_att186::PBAD-dCas9": *E. coli* strain DH10B-ALT (Addgene, #61151), constitutively expressing *araC*, was used as the target strain for integration of the dCas9 cassette. The plasmid “pOSIP-KO_PBAD-dCas9” was introduced into the cells, and P_{BAD}-dCas9 cassette was clonetegrated into the *att186* site[8]. The positive clones were selected on LB supplemented with Kanamycin (25 μg/ml).

“E_SJ105”, “E_SJ-106”, “E_SJ-106_2x”, “E_SJ107”, “E_SJ-1072x”, “E_pSJ108” and “E_SJ111”: Strain ”E. coli, att186::PBAD-dCas9” was transformed with reporter plasmids pSJ-105, pSJ-106, pSJ106_2x, pSJ107, pSJ107 2x, pSJ108 and pSJ111. The positive clones were grown on LB supplemented with Kanamycin (25 μg/ml) and Ampicillin (50 μg/ml).

"LT2, att186::PBAD-dCas9": *Salmonella* strain TH437 (Kelly T. Hughes), natively expressing *araC*, was used as the target strain for integrating the dCas9 cassette. The plasmid pOSIP-KO_PBAD-dCas9 was introduced into the cells, and PBAD-dCas9 cassette was clonetegrated allowing integration into the *att186* site[8]. The positive clones were selected on LB supplemented with Kanamycin (25 μg/ml).

“LT2_J105”, “LT2_SJ105, LT2_no gRNA", “LT2_SJ106”, “LT2_SJ106_2x”, “LT2_SJ107”, “LT2_SJ107 2x”, “LT2_SJ108” and “LT2_SJ111”: Strain “*LT2*, att186::PBAD-dCas9” was transformed with reporter plasmids pJ105, pSJ105, pJF076Sa, pSJ106, pSJ106 2x, pSJ107, pSJ107 2x, pSJ108 and pSJ111 to generate strains. The positive clones were grown on LB supplemented with Kanamycin (25 μg/ml) and Ampicillin (50 μg/ml).

Plant-derived ATF and promoter pair clones

pJUB1, pJUB1DBD, pANAC102 and pGRF9: PCR-amplified JUB1 full-length (primer pair JUB-fv/JUB-rv, on “pGN003B-JUB1”), JUB1 DBD (primer pair JUBDBD-fv/JUB-rv, on “pGNPP004”), ANAC102 full-length (primer pair ANAC-fv/JUB-rv, on “pGNPP0033”) or GRF9 full-length (primer pair GRF-fv/JUB-rv, on “pGNPP002”) was assembled in PCR-amplified backbone plasmid containing the 5-aa linker fused to SoxS(R93A) activation domain optimized for expression in *S.* Typhimurium ($SoxS(R93A)_{opt}$) allowing for the arabinose-controlled expression of ATF (primer pair PBAD-SOX-fv/PBAD-SOX-rv, on *“*AVA_TetR_SOXS_a”, see "Reporter plasmids for dCas9-derived ATFs") using the NEBuilder HiFi DNA assembly strategy. The generated plasmids were pJUB1, pJUBDBD, pANAC102 and pGRF9.

pJUB2x: A synthetic fragment containing *Sal*I recognition site, two copies of JUB1 binding site fused to 3’-end of *J1* region of the synthetic promoter (84 bp upstream of ATG) and 171-bp of 5’-end of sfGFP ended with *Nco*I site at 3' was designed and generated by BioCat Inc. (Heidelberg, Germany), and cloned in plasmid pUC57 (pUC57-BsaI-free_ JUB2x). The plasmid was digested with *Sal*I and *Nco*I to obtain a 359-bp fragment. Next, the 359-bp fragment was cloned in *Sal*I/*Nco*I-digested low-copy pKH70-PrpsM-sfGFP (2632 bp in length, lab collection) using T4 ligase (NEB). The generated plasmid was called pJUB2x. Plasmid pKH70-PrpsM-sfGFP harbors ori 29807 (derived from ori p15A)[10].

pJUB0x: Annealed oligonucleotide JUB0X-fv/JUB0X-rv was assembled in *Sal*I/*Xcm*aI-digested pJUB2X to remove JUB1 binding sites. The generated plasmid was called pJUB0X.

pJUB1x, pJUB4x, pANAC2x, pANAC4x, pGRF2x and pGRF4x: The plasmid pJUB2x was digested with *Sal*I/*Xcm*I to remove 186 bp. One, two or four copies of the binding site of JUB1, ANAC102 and GRF9 were PCR-amplified from, respectively, “pGN005B-1xBS-JUB1”[9], “pGN005B-4xBS-JUB1”[9], “pGN005B-2xBS-JANAC102”[9], “pGN005B-4xBS-ANAC102”[9], “pGN005B-2xBS-GRF9”[9] and “pGN005B-4xBS-GRF9”[9] (primer pair BS-fv/BS-rv), and assembled in linearized pJUB2X plasmid using the NEBuilder HiFi DNA assembly strategy to generate “pJUB1x”, “pJUB4x”, “pANAC2x”, “pANAC4x”, “pGRF2x” and “pGRF4X”.

“pKO_PBAD-JUB1” and “pKO-PBAD-JUB1DBD”: PCR-amplified “P_{BAD}-JUB1-linker-SOXS(R93A)$_{opt}$-T_{rrnBT1}” (primer pair plantTF-fv/plantTF-rv on pJUB1) or “P_{BAD}-DBD of JUB1-linker- SOXS(R93A)$_{opt}$-T_{rrnBT1}” (primer pair plantTF-fv/plantTF-rv on “pJUB1DBD”) was cloned into *Bam*HI/*Spe*I-digested pOSIP-KO[8] to generate plasmids “pOSIP-KO_PBAD-JUB1” and “pOSIP-KO_PBAD-JUB1DBD”, respectively.

Plant driver/reporter strains

"*E. coli*_pJUB1" and "*E. coli*_pJUB1DBD": pJUB1 and pJUB1DBD were introduced into DH10B-ALT via heat-shock method. The positive clones were grown on LB supplemented with Kanamycin (25 μg/ml).

“*E. coli*_pJUB1_pJUB2X” and “*E. coli*_pJUB1DBD_pJUB2X”: “*E. coli*_pJUB1” and “*E. coli*_pJUB1DBD” were transformed with pJUB2X. The positive clones were grown on LB supplemented with Kanamycin (25 μg/ml) and Ampicillin (50 μg/ml).

"*E. coli*_ att186:: PBAD-JUB1" and "*E. coli*_ att186:: PBAD-JUB1DBD": *E. coli* strain DH10B-ALT (Addgene, #61151), constitutively expressing araC, was used as the target strain for integration of JUB-derived ATF cassettes. The plasmid “pKO_PBAD-JUB1” or “pKO-PBAD-JUB1DBD” was introduced into the cells, allowing integration into the *att186* site was clonetegrated using a kanamycin-resistant pOSIP[8]. The positive clones were grown on LB supplemented with Kanamycin (25 μg/ml).

"*E. coli*_ att186:: PBAD-JUB1_pJUB2X" and "*E. coli*_att186:: PBAD-JUB1DBD_pJUB2X ": "*E. coli*_att186:: PBAD-JUB1" and "*E. coli*_att186:: PBAD-JUB1DBD" were transformed with reporter plasmid pJUB2X. The positive clones were grown on LB supplemented with Kanamycin (25 μg/ml) and Ampicillin (50 μg/ml).

"LT2_pJUB1": pJUB1 was introduced into *S.* Typhimurium LT2 via electroporation. The positive clones were grown on LB supplemented with Kanamycin (25 μg/ml).

“LT2_pJUB1_pJUB0X”, “LT2_pJUB1_pJUB1X”, “LT2_pJUB1_pJUB4X”, “LT2_pJUB1_ pJUB5X” and “LT2_pJUB1_pJUB6X”: Strain “LT2_pJUB1” was transformed with plasmids pJUB0X, pJUB1X, pJUB2X, pJUB4X, pJUB5X or pJUB6X. The positive clones were grown on LB supplemented with Kanamycin (25 μg/ml) and Ampicillin (50 μg/ml).

"LT2_PRSM": Plasmid pKH70-PrpsM-sfGFP (lab collection) was introduced into *S.* Typhimurium LT2 via electroporation. The positive clones were grown on LB supplemented with Ampicillin (50 μg/ml).

"EM12877_PRSM": Plasmid pKH70-PrpsM-sfGFP (lab collection) was introduced into EM12877 via electroporation. The positive clones were grown on LB supplemented with Ampicillin (50 μg/ml).

"LT2_pANAC102_pANAC2X" and "LT2_pANAC102_pANAC4X": pANAC102 was introduced into *S.* Typhimurium LT2 via electroporation to generate strain "LT2_ pANAC102". The positive clones were grown on LB supplemented with Kanamycin (25 μg/ml). Plasmids "pANAC2X" and "pANAC4X" were introduced into strain "LT2_pANAC102" to generate strains "LT2_pANAC102_pANAC2X" and "LT2_pANAC102_pANAC4X", respectively. The positive clones were grown on LB supplemented with Kanamycin (25 μg/ml) and Ampicillin (50 μg/ml).

"LT2_pGRF9_pGRF2X" and "LT2_pGRF9_pGRF4X": pGRF9 was introduced into *S.* Typhimurium LT2 via electroporation to generate strain "LT2, pGRF9". The positive clones were grown on LB supplemented with Kanamycin (25 μg/ml). Plasmids "pGRF2X" and "pGRF4X" were introduced into strain "LT2_pGRF9" to generate strains "LT2_pGRF9_pGRF2X" and "LT2_pGRF9_pGRF4X", respectively. The positive clones were grown on LB supplemented with Kanamycin (25 μg/ml) and Ampicillin (50 μg/ml).

"LT2_ΔaraBAD_PproA::ParaE_pJUB1_pJUB2X" and "LT2_ΔaraBAD_PproA::ParaE_pJUB1_pJUB4X": Plasmid pJUB1 was introduced into strain "LT2_ΔaraBAD_PproA::ParaE". The positive clones were grown on LB supplemented with Kanamycin (25 μg/ml). Next, plasmids pJUB2X and pJUB4X were introduced into strain "LT2_ΔaraBAD_PproA::ParaE_pJUB1" to generate strains "LT2_ΔaraBAD_PproA::ParaE_pJUB1_pJUB2X" and "LT2_ΔaraBAD_PproA::ParaE_JUB1_pJUB4X", respectively. The positive clones were grown on LB supplemented with Kanamycin (25 μg/ml) and Ampicillin (50 μg/ml).

Supplementary Tables

Table S1. Strains used in present study.

**Strain name**	**Relevant genotype**	**Source**
TH437	*Salmonella enterica* serovar Typhimurium LT2	Kelly T. Hughes
DH10B-ALT	*Escherchia coli DH10b*,attBλ:: P_{cons}-AraC, PI_{aclq}-lacI, tetR	Addgene, #61151
TH6701	LT2, *ΔaraBAD925::tetRA*	Kelly T. Hughes
TH6706	LT2, Δ*araBAD925*::*tetRA* / pKD46	Kelly T. Hughes
EM12441	*LT2, ΔaraBAD::sfGFP*	This study
EM12709	*LT2, ΔaraBAD::sfGFP,* P_{araE}::P_{proA}/ pKD46	This study
EM12471	LT2, Δ*araBAD925*::*sfGFP*	This study
EM12472	LT2, Δ*araBAD925*::*sfGFP* / pKD46	This study
TH3730	LT2, P_{flhDC}::Tn10dTc$_{[del-25]}$(T-POP)	Kelly T. Hughes
EM12498	LT2, Δ*araBAD925*::*sfGFP,* P_{araE}::*tetRA*/pKD46	This study
EM9661	LT2, *Δhin-5717*::*FRT flgE6506* (*S171C*), $P_{flhDC23254}$::P_{proA}-RBS (Δbp -598 to AUG of flhD)	Lab collection
EM12886	LT2 / pBAD24-sfGFP1x	This study
EM12887	LT2, *araBAD925*::*tetRA* / pBAD24-sfGFP1x	This study
EM12888	LT2, P_{araE}::P_{proA} / pBAD24-sfGFP1x	This study
EM12889	LT2, *araBAD925*::*tetRA*, P_{araE}::P_{proA} / pBAD24-sfGFP1x	This study
EM12746	LT2, P_{araE}::P_{proA}	This study
EM8513	LT2, *Δhin-5717*::*FRT flgE6506* (*S171C*), $P_{flhDC23254}$::P_{proD}-RBS (Δbp -598 to AUG of flhD)	Lab collection
EM12712	LT2, Δ*araBAD925*::*sfGFP,* P_{araE}::P_{araD}	This study
EM12967	LT2, P_{araE}::P_{proD}	This study
EM12877	LT2, *araBAD925*::*tetRA,* P_{araE}::P_{proA}	This study
EM13000	LT2, *araBAD925*::*tetRA,* P_{araE}::P_{proD}	This study
*E. coli*_att186::PBAD-dCas9	DH10B-ALT, *att186*::P_{BAD}-*dCas9*-T_{rrnBT1}-$Kana_R$	This study
*E. coli*_SJ105	DH10B-ALT, *att186*::P_{BAD}-*dCas9*-T_{rrnBT1}-$Kana_R$ / pSOXJ-105	This study

*E. coli*_SJ106	DH10B-ALT, *att186*::P_{BAD}-*dCas9*-T_{rrnBT1}-Kana$_{R}$ / pSOXJ-106	This study
*E. coli*_SJ106_2x	DH10B-ALT, *att186*::P_{BAD}-*dCas9*-T_{rrnBT1}-Kana$_{R}$ / pSOXJ-106_2x	This study
*E. coli*_SJ107	DH10B-ALT, *att186*::P_{BAD}-*dCas9*-T_{rrnBT1}-Kana$_{R}$ / pSOXJ-107	This study
*E. coli*_SJ107_2x	DH10B-ALT, *att186*::P_{BAD}-*dCas9*-T_{rrnBT1}-Kana$_{R}$ / pSOXJ-107_2x	This study
*E. coli*_SJ108	DH10B-ALT, *att186*::P_{BAD}-*dCas9*-T_{rrnBT1}-Kana$_{R}$ / pSOXJ-108	This study
*E. coli*_SJ111	DH10B-ALT, *att186*::P_{BAD}-*dCas9*-T_{rrnBT1}-Kana$_{R}$ / pSOXJ-111	This study
LT2_att186::PBAD-dCas9	LT2, *att186*::P_{BAD}-*dCas9*-T_{rrnBT1}-Kana$_{R}$	This study
LT2_J105	LT2, *att186*::P_{BAD}-*dCas9*-T_{rrnBT1}-Kana$_{R}$ / pJ-105	This study
LT2_SJ105	LT2, *att186*::P_{BAD}-*dCas9*-T_{rrnBT1}-Kana$_{R}$ / pSOXJ-105	This study
LT2_no gRNA	LT2, *att186*::P_{BAD}-*dCas9*-T_{rrnBT1}-Kana$_{R}$ / pJF076Sa	This study
LT2_SJ106	LT2, *att186*::P_{BAD}-*dCas9*-T_{rrnBT1}-Kana$_{R}$ / pSOXJ-106	This study
LT2_SJ106_2x	LT2, *att186*::P_{BAD}-*dCas9*-T_{rrnBT1}-Kana$_{R}$ / pSOXJ-106_2x	This study
LT2_ SJ107	LT2, *att186*::P_{BAD}-*dCas9*-T_{rrnBT1}-Kana$_{R}$ / pSOXJ-107	This study
LT2_SJ107_2x	LT2, *att186*::P_{BAD}-*dCas9*-T_{rrnBT1}-Kana$_{R}$ / pSOXJ-107_2x	This study
LT2_SJ108_2x	LT2, *att186*::P_{BAD}-*dCas9*-T_{rrnBT1}-Kana$_{R}$ / pSOXJ-108	This study
LT2_SJ111_2x	LT2, *att186*::P_{BAD}-*dCas9*-T_{rrnBT1}-Kana$_{R}$ / pSOXJ-111	This study
EM1300_att186::PBAD-dCas9	LT2,*araBAD925*::*tetRA*, P_{araE}::P_{proD}, *att186*::P_{BAD}-*dCas9*-T_{rrnBT1}-Kana$_{R}$	This study
EM1300_SJ105	EM1300, *att186*::P_{BAD}-*dCas9*-T_{rrnBT1}-Kana$_{R}$ / pSOXJ-105	This study
EM1300_SJ106	EM1300, *att186*::P_{BAD}-*dCas9*-T_{rrnBT1}-Kana$_{R}$ / pSOXJ-106	This study
EM1300_SJ106_2x	EM1300, *att186*::P_{BAD}-*dCas9*-T_{rrnBT1}-Kana$_{R}$ / pSOXJ-106_2x	This study
EM1300_ SJ107	EM1300, *att186*::P_{BAD}-*dCas9*-T_{rrnBT1}-Kana$_{R}$ / pSOXJ-107	This study
EM1300_SJ107_2x	EM1300, *att186*::P_{BAD}-*dCas9*-T_{rrnBT1}-Kana$_{R}$ / pSOXJ-107_2x	This study
EM1300_SJ108_2x	EM1300, *att186*::P_{BAD}-*dCas9*-T_{rrnBT1}-Kana$_{R}$ / pSOXJ-108	This study
EM1300_SJ111_2x	EM1300, *att186*::P_{BAD}-*dCas9*-T_{rrnBT1}-Kana$_{R}$ / pSOXJ-111	This study
*E. coli*_pJUB1	DH10B-ALT / pJUB1	This study
*E. coli*_pJUB1DBD	DH10B-ALT / pJUB1DBD	This study
*E. coli*_pJUB1_pJUB2X	*E. coli* / pJUB1, pJUB2X	This study

*E. coli*_pJUB1DBD_pJUB2X	*E. coli /* pJUB1DBD, pJUB2X	This study
*E. coli*_ att186:: PBAD-JUB1	DH10B-ALT, *att186*::P_{BAD}-*JUB1-5aa linker-SOXS (R93A)*$_{opt}$-T_{rrnBT1}-$Kana_R$	This study
*E. coli*_ att186:: PBAD-JUB1DBD	DH10B-ALT, *att186*::P_{BAD}-*JUB1*$_{DBD}$*-5aa linker-SOXS (R93A)*$_{opt}$-T_{rrnBT1}-$Kana_R$	This study
*E. coli*_ att186:: PBAD-JUB1_pJUB2X	DH10B-ALT, *att186*::P_{BAD}-*JUB1-5aa linker-SOXS (R93A)*$_{opt}$-T_{rrnBT1}-$Kana_R$ / pJUB2X	This study
*E. coli*_att186:: PBAD-JUB1DBD_pJUB2X	DH10B-ALT, *att186*::P_{BAD}-*JUB1*$_{DBD}$*-5aa linker-SOXS (R93A)*$_{opt}$-T_{rrnBT1}-$Kana_R$ / pJUB2X	This study
LT2_pJUB1	LT2 / pJUB1	This study
LT2_pJUB1_pJUB0X	LT2 / pJUB1, pJUB0X	This study
LT2_pJUB1_pJUB1X	LT2 / pJUB1, pJUB1X	This study
LT2_pJUB1_pJUB1X	LT2 / pJUB1, pJUB2X	This study
LT2_pJUB1_pJUB4X	LT2 / pJUB1, pJUB4X	This study
LT2_pJUB1_ pJUB5X	LT2 / pJUB1, pJUB5X	This study
LT2_pJUB1_pJUB6X	LT2 / pJUB1, pJUB6X	This study
LT2_PRSM	LT2 / pKH70-PrpsM-sfGFP	This study
EM12877_PRSM	LT2, *araBAD925*::*tetRA*, P_{araE}::P_{proA} / pKH70-PrpsM-sfGFP	This study
LT2_pANAC102	LT2 / pANAC102	This study
LT2_pANAC102_pANAC2X	LT2 / pANAC102, pANAC2X	This study
LT2_pANAC102_pANAC4X	LT2 / pANAC102, pANAC4X	This study
LT2_pGRF9	LT2 / pGRF9	This study
LT2_pGRF9_pGRF2X	LT2 / pGRF9, pGRF2X	This study
LT2_pGRF9_pGRF4X	LT2 / pGRF9, pGRF4X	This study
EM12877_pJUB1	LT2, *araBAD925*::*tetRA,* P_{araE}::P_{proA} / pJUB1	This study
EM12877_pJUB1_pJUB1X	LT2, *araBAD925*::*tetRA*, P_{araE}::P_{proA} / pJUB1, pJUB1X	This study
EM12877_pJUB1_pJUB2X	LT2, *araBAD925*::*tetRA,* P_{araE}::P_{proA} / pJUB1, pJUB2X	This study
EM12877_pJUB1_pJUB4X	LT2, *araBAD925*::*tetRA,* P_{araE}::P_{proA} / pJUB1, pJUB4X	This study
RKLT2	LT2, ΔP_{RamR}::KanSceI (Δbp -139 to AUG of STM0580)	This study
RKLT3	LT2, *araBAD925*::*tetRA*, P_{araE}::P_{proA}, ΔP_{RamR}::KanSceI (Δbp -139 to AUG of STM0580)	This study
SALSOR 0.1	LT2, ΔP_{RamR}::ATG-RBS_{syn}-P_{RamR}-P_{RamRBS}-UP-RBS_{RiboJ}-sfGFP-T_{rrnBT1}(Δbp -139 to AUG of STM0580)	This study

SALSOR4X	LT2, *araBAD925::tetRA, ParaE::PproA*, ΔP_{RamR}::ATG-RBS$_{syn}$-P$_{synJUB1\text{-}4x}$-P$_{RamRBS}$-UP-RBS$_{RiboJ}$-sfGFP-T$_{rrnBT1}$(Δbp -139 to AUG of STM0580)	This study
SALSOR 0.2	LT2, *araBAD925*::*tetRA*, P_{araE}::P_{proA}, ΔP_{RamR}::ATG-RBS$_{syn}$-P$_{synJUB1\text{-}4x}$-P$_{RamRBS}$-UP-RBS$_{RiboJ}$-sfGFP-T$_{rrnBT1}$(Δbp -139 to AUG of STM0580)/pJUB1	This study
*E. coli*_pK2151200	DH10b, pK2151200	This study
*E. coli*_pK2151201	DH10b, pK2151201	This study
E. coli, att186:: PBAD-JUB1_pCAROTENE5X	DH10B-ALT, *att186*::P_{BAD}-*JUB1*-T_{BBa_B002}/pCAROTENE5X	This study

Table S2. Sequence of plasmids used in present study.

Plasmid name	Sequence (5' - 3')	Source
pEM8317	CTCGAGGATCCCGGGTACCTGCAGCTAGCGTCGACGAAAGGCTACGGCCGTTAATTGGTCGCCTGAGAAGTTACGGAGAGTAAAAT<br>GAAAGTTCGTGCTTCCGTCAAGAAATTATGCCGTAACTGCAAAATCGTTAAGCGTGATGGTGTCATCCGTGTGATTTGCAGTGCCGAG<br>CCGAAGCATAAACAGCGCCAAGGCTGATTTTTTCGCATATTTTTCTTGCAAAGTTGGGTTGAGCTGGCTAGATTAGCCAGCCAATCTTT<br>TGTATGTCTGTACGTTTCCATTTGAGTATCCTGAAAACGGGCTTTTCAGCATGGTACGTACATATTAAATAGTAGGAGTGCATAGTGGC<br>CCGTATAGCAGGCATTAACATTCCTGATCAGAAACACGCCGTGATCGCGTTAACTTCGATCTACGGTGTCGGCAAGACCCGTTCTAAA<br>GCCATCCTGGCTGGAATTCATAAGGAGGAAAAACATATGTCTAAAGGTGAAGAACTGTTCACCGGTGTTGTTCCGATCCTGGTTGAAC<br>TGGATGGTGATGTTAACGGCCACAAATTCTCTGTTCGTGGTGAAGGTGAAGGTGATGCAACCAACGGTAAACTGACCCTGAAATTCAT<br>CTGCACTACCGGTAAACTGCCGGTTCCATGGCCGACTCTGGTGACTACCCTGACCTATGGTGTTCAGTGTTTTTCTCGTTACCCGGAT<br>CACATGAAGCAGCATGATTTCTTCAAATCTGCAATGCCGGAAGGTTATGTACAGGAGCGCACCATTTCTTTCAAAGACGATGGCACCT<br>ACAAAACCCGTGCAGAGGTTAAATTTGAAGGTGATACTCTGGTGAACCGTATTGAACTGAAAGGCATTGATTTCAAAGAGGACGGCAA<br>CATCCTGGGCCACAAACTGGAATATAACTTCAACTCCCATAACGTTTACATCACCGCAGACAAACAGAAGAACGGTATCAAAGCTAACT<br>TCAAAATTCGCCATAACGTTGAAGACGGTAGCGTACAGCTGGCGGACCACTACCAGCAGAACACTCCGATCGGTGATGGTCCGGTTC<br>TGCTGCCGGATAACCACTACCTGTCCACCCAGTCTGTTCTGTCCAAAGACCCGAACGAAAAGCGCGACCACATGGTGCTGCTGGAGT<br>TCGTTACTGCAGCAGGTATCACGCACGGCATGGATGAGCTCTACAAATGATAAGCGGCCGCTCTAGAGGCATCAAATAAAACGAAAG<br>GCTCAGTCGAAAGACTGGGCCTTTCGTTTTATCTGTTGTTTGTCGGTGAACGCTCTCCTGAGTAGGACAAATCCGCCGCCCTAGACCT<br>AGGCGGAGATATTCCGCTTCCTCGCTCACTGACTCGCTCCGCTCGGTCGTTCGGCTGCGGCGAGCGGTACCGGCTTACTGGCGGGG<br>CGGAAATTTCCTGGAAGATGCCAGGAAGAAACTTAACAGGGAAGCGATAAGGCCACGGCAGAGCCGTTTTTCCATAGGCTCCGCCCC<br>CCTGACAAGCATCACGAAATCTGACGCTCAAATCAGTGGTGGCGAAACCCGACAGGACTTAAAGATACCAGGCGTTTCCCCCTGGTG<br>GCTCCCTCGTGCGCTCTCCTGTTCCTGCCTTTCGGTTTACCGGTGTCACTCCGCTGTTATGGCCGCGTTTATCTCATTCCACGCCTGA<br>CACTCGGTTCCGGGAAGGCAGTTCGCTCCAAGCTGGACTGTATGCACGAACCCCCCGTTCAGTCCGACTGCTGCGCCTGTTCCGGT<br>AACTATCGACTTGAGTCCAAGCCGGAAAGACACGACAAAGCGCCACTGGCAGCAGCCACTGGTAAGAGGGTGTACAAGAGATTACTG<br>ATGCAGAGTTCTTGAAGTCAGAGCCAAACTACGGCTAAACTGGAAGGACGAATTTGGTGGCAGCGGTCTTGTACACCCTGTTACCAC<br>GGTTCAGAAGTTCCCCAACTTACTGAACCTTCGAAAAACCACCTCCCCAGGTGGTTTTTTCGTTTTCAGAGCAAGAGATTACGACGCA<br>ACGGAAAGGATCTCAAGAGGTTCATCTTACTAGTGCTTGGATTCTCACCAATAAAAAACGCCCGGCGGCAACCGAGCGTTCTGAACAA<br>ATCCAGATGGAGTTCTGAGGTCATTACTGGATCTATCAACAGGAGTCCAAGCGAGCTCGTAAACTTGGTCTGACAGTTACCAATGCTT<br>AATCAGTGAGGCACCTATCTCAGCGATCTGTCTATTTCGTTCATCCATAGTTGCCTGACTCCCCGTCGTGTAGATAACTACGATACGG<br>GAGGGCTTACCATCTGGCCCCAGTGCTGCAATGATACCGCGAGACCCACGCTCACCGGCTCCAGATTTATCAGCAATAAACCAGCCA<br>GCCGGAAGGGCCGAGCGCAGAAGTGGTCCTGCAACTTTATCCGCCTCCATCCAGTCTATTAATTGTTGCCGGGAAGCTAGAGTAAGT<br>AGTTCGCCAGTTAATAGTTTGCGCAACGTTGTTGCCATTGCTACAGGCATCGTGGTGTCACGCTCGTCGTTTGGTATGGCTTCATTCA<br>GCTCCGGTTCCCAACGATCAAGGCGAGTTACATGATCCCCCATGTTGTGCAAAAAAGCGGTTAGCTCCTTCGGTCCTCCGATCGTTGT<br>CAGAAGTAAGTTGGCCGCAGTGTTATCACTCATGGTTATGGCAGCACTGCATAATTCTCTTACTGTCATGCCATCCGTAAGATGCTTTT<br>CTGTGACTGGTGAGTACTCAACCAAGTCATTCTGAGAATAGTGTATGCGGCGACCGAGTTGCTCTTGCCCGGCGTCAATACGGGATA<br>ATACCGCGCCACATAGCAGAACTTTAAAAGTGCTCATCATTGGAAAACGTTCTTCGGGGCGAAAACTCTCAAGGATCTTACCGCTGTT<br>GAGATCCAGTTCGATGTAACCCACTCGTGCACCCAACTGATCTTCAGCATCTTTTACTTTCACCAGCGTTTCTGGGTGAGCAAAAACA<br>GGAAGGCAAAATGCCGCAAAAAAGGGAATAAGGGCGACACGGAAATGTTGAATACTCATACTCTTCCTTTTTCAATATTATTGAAGCAT	Lab collection

	TTATCAGGGTTATTGTCTCATGAGCGGATACATATTTGAATGTATTTAGAAAAATAAACAAATAGGGGTTCCGCGCACATTTCCCCGAA<br>AAGTGCCACCTGACGTCTAAGAAACCATTATTATCATGACATTAACCTATAAAAATAGGCGTATCACGAGGCCCTTTCGTCTTCAC	
pKD46	CATCGATTTATTATGACAACTTGACGGCTACATCATTCACTTTTTCTTCACAACCGGCACGGAACTCGCTCGGGCTGGCCCCGGTGCA<br>TTTTTTAAATACCCGCGAGAAATAGAGTTGATCGTCAAAACCAACATTGCGACCGACGGTGGCGATAGGCATCCGGGTGGTGCTCAAA<br>AGCAGCTTCGCCTGGCTGATACGTTGGTCCTCGCGCCAGCTTAAGACGCTAATCCCTAACTGCTGGCGGAAAAGATGTGACAGACGC<br>GACGGCGACAAGCAAACATGCTGTGCGACGCTGGCGATATCAAAATTGCTGTCTGCCAGGTGATCGCTGATGTACTGACAAGCCTCG<br>CGTACCCGATTATCCATCGGTGGATGGAGCGACTCGTTAATCGCTTCCATGCGCCGCAGTAACAATTGCTCAAGCAGATTTATCGCCA<br>GCAGCTCCGAATAGCGCCCTTCCCCTTGCCCGGCGTTAATGATTTGCCCAAACAGGTCGCTGAAATGCGGCTGGTGCGCTTCATCCG<br>GGCGAAAGAACCCCGTATTGGCAAATATTGACGGCCAGTTAAGCCATTCATGCCAGTAGGCGCGCGGACGAAAGTAAACCCACTGGT<br>GATACCATTCGCGAGCCTCCGGATGACGACCGTAGTGATGAATCTCTCCTGGCGGGAACAGCAAAATATCACCCGGTCGGCAAACAA<br>ATTCTCGTCCCTGATTTTTCACCACCCCCTGACCGCGAATGGTGAGATTGAGAATATAACCTTTCATTCCCAGCGGTCGGTCGATAAAA<br>AAATCGAGATAACCGTTGGCCTCAATCGGCGTTAAACCCGCCACCAGATGGGCATTAAACGAGTATCCCGGCAGCAGGGGATCATTT<br>TGCGCTTCAGCCATACTTTTCATACTCCCGCCATTCAGAGAAGAAACCAATTGTCCATATTGCATCAGACATTGCCGTCACTGCGTCTT<br>TTACTGGCTCTTCTCGCTAACCAAACCGGTAACCCCGCTTATTAAAAGCATTCTGTAACAAAGCGGGACCAAAGCCATGACAAAAACG<br>CGTAACAAAAGTGTCTATAATCACGGCAGAAAAGTCCACATTGATTATTTGCACGGCGTCACACTTTGCTATGCCATAGCATTTTTATC<br>CATAAGATTAGCGGATCCTACCTGACGCTTTTTATCGCAACTCTCTACTGTTTCTCCATACCCGTTTTTTTGGGAATTCGAGCTCTAAG<br>GAGGTTATAAAAAATGGATATTAATACTGAAACTGAGATCAAGCAAAAGCATTCACTAACCCCCTTTCCTGTTTTCCTAATCAGCCCGG<br>CATTTCGCGGGCGATATTTTCACAGCTATTTCAGGAGTTCAGCCATGAACGCTTATTACATTCAGGATCGTCTTGAGGCTCAGAGCTG<br>GGCGCGTCACTACCAGCAGCTCGCCCGTGAAGAGAAAGAGGCAGAACTGGCAGACGACATGGAAAAAGGCCTGCCCCAGCACCTGT<br>TTGAATCGCTATGCATCGATCATTTGCAACGCCACGGGGCCAGCAAAAAATCCATTACCCGTGCGTTTGATGACGATGTTGAGTTTCA<br>GGAGCGCATGGCAGAACACATCCGGTACATGGTTGAAACCATTGCTCACCACCAGGTTGATATTGATTCAGAGGTATAAAACGAATGA<br>GTACTGCACTCGCAACGCTGGCTGGGAAGCTGGCTGAACGTGTCGGCATGGATTCTGTCGACCCACAGGAACTGATCACCACTCTTC<br>GCCAGACGGCATTTAAAGGTGATGCCAGCGATGCGCAGTTCATCGCATTACTGATCGTTGCCAACCAGTACGGCCTTAATCCGTGGA<br>CGAAAGAAATTTACGCCTTTCCTGATAAGCAGAATGGCATCGTTCCGGTGGTGGGCGTTGATGGCTGGTCCCGCATCATCAATGAAAA<br>CCAGCAGTTTGATGGCATGGACTTTGAGCAGGACAATGAATCCTGTACATGCCGGATTTACCGCAAGGACCGTAATCATCCGATCTGC<br>GTTACCGAATGGATGGATGAATGCCGCCGCGAACCATTCAAAACTCGCGAAGGCAGAGAAATCACGGGGCCGTGGCAGTCGCATCC<br>CAAACGGATGTTACGTCATAAAGCCATGATTCAGTGTGCCCGTCTGGCCTTCGGATTTGCTGGTATCTATGACAAGGATGAAGCCGAG<br>CGCATTGTCGAAAATACTGCATACACTGCAGAACGTCAGCCGGAACGCGACATCACTCCGGTTAACGATGAAACCATGCAGGAGATT<br>AACACTCTGCTGATCGCCCTGGATAAAACATGGGATGACGACTTATTGCCGCTCTGTTCCCAGATATTTCGCCGCGACATTCGTGCAT<br>CGTCAGAACTGACACAGGCCGAAGCAGTAAAAGCTCTTGGATTCCTGAAACAGAAAGCCGCAGAGCAGAAGGTGGCAGCATGACAC<br>CGGACATTATCCTGCAGCGTACCGGGATCGATGTGAGAGCTGTCGAACAGGGGGATGATGCGTGGCACAAATTACGGCTCGGCGTC<br>ATCACCGCTTCAGAAGTTCACAACGTGATAGCAAAACCCCGCTCCGGAAAGAAGTGGCCTGACATGAAAATGTCCTACTTCCACACCC<br>TGCTTGCTGAGGTTTGCACCGGTGTGGCTCCGGAAGTTAACGCTAAAGCACTGGCCTGGGGAAAACAGTACGAGAACGACGCCAGA<br>ACCCTGTTTGAATTCACTTCCGGCGTGAATGTTACTGAATCCCCGATCATCTATCGCGACGAAAGTATGCGTACCGCCTGCTCTCCCG<br>ATGGTTTATGCAGTGACGGCAACGGCCTTGAACTGAAATGCCCGTTTACCTCCCGGGATTTCATGAAGTTCCGGCTCGGTGGTTTCGA<br>GGCCATAAAGTCAGCTTACATGGCCCAGGTGCAGTACAGCATGTGGGTGACGCGAAAAAATGCCTGGTACTTTGCCAACTATGACCC<br>GCGTATGAAGCGTGAAGGCCTGCATTATGTCGTGATTGAGCGGGATGAAAAGTACATGGCGAGTTTTGACGAGATCGTGCCGGAGTT<br>CATCGAAAAAATGGACGAGGCACTGGCTGAAATTGGTTTTGTATTTGGGGAGCAATGGCGATGACGCATCCTCACGATAATATCCGG<br>GTAGGCGCAATCACTTTCGTCTACTCCGTTACAAAGCGAGGCTGGGTATTTCCCGGCCTTTCTGTTATCCGAAATCCACTGAAAGCAC<br>AGCGGCTGGCTGAGGAGATAAATAATAAACGAGGGGCTGTATGCACAAAGCATCTTCTGTTGAGTTAAGAACGAGTATCGAGATGGC<br>ACATAGCCTTGCTCAAATTGGAATCAGGTTTGTGCCAATACCAGTAGAAACAGACGAAGAATCCATGGGTATGGACAGTTTTCCCTTTG<br>ATATGTAACGGTGAACAGTTGTTCTACTTTTGTTTGTTAGTCTTGATGCTTCACTGATAGATACAAGAGCCATAAGAACCTCAGATCCTT<br>CCGTATTTAGCCAGTATGTTCTCTAGTGTGGTTCGTTGTTTTTGCGTGAGCCATGAGAACGAACCATTGAGATCATACTTACTTTGCAT<br>GTCACTCAAAAATTTTGCCTCAAAACTGGTGAGCTGAATTTTTGCAGTTAAAGCATCGTGTAGTGTTTTTCTTAGTCCGTTACGTAGGTA<br>GGAATCTGATGTAATGGTTGTTGGTATTTTGTCACCATTCATTTTTATCTGGTTGTTCTCAAGTTCGGTTACGAGATCCATTTGTCTATC<br>TAGTTCAACTTGGAAAATCAACGTATCAGTCGGGCGGCCTCGCTTATCAACCACCAATTTCATATTGCTGTAAGTGTTTAAATCTTTACT<br>TATTGGTTTCAAAACCCATTGGTTAAGCCTTTTAAACTCATGGTAGTTATTTTCAAGCATTAACATGAACTTAAATTCATCAAGGCTAATC<br>TCTATATTTGCCTTGTGAGTTTTCTTTTGTGTTAGTTCTTTTAATAACCACTCATAAATCCTCATAGAGTATTTGTTTTCAAAAGACTTAAC<br>ATGTTCCAGATTATATTTTATGAATTTTTTTAACTGGAAAAGATAAGGCAATATCTCTTCACTAAAAACTAATTCTAATTTTTCGCTTGAGA<br>ACTTGGCATAGTTTGTCCACTGGAAAATCTCAAAGCCTTTAACCAAAGGATTCCTGATTTCCACAGTTCTCGTCATCAGCTCTCTGGTT<br>GCTTTAGCTAATACACCATAAGCATTTTCCCTACTGATGTTCATCATCTGAGCGTATTGGTTATAAGTGAACGATACCGTCCGTTCTTTC<br>CTTGTAGGGTTTTCAATCGTGGGGTTGAGTAGTGCCACACAGCATAAAATTAGCTTGGTTTCATGCTCCGTTAAGTCATAGCGACTAAT<br>CGCTAGTTCATTTGCTTTGAAAACAACTAATTCAGACATACATCTCAATTGGTCTAGGTGATTTTAATCACTATACCAATTGAGATGGGC<br>TAGTCAATGATAATTACTAGTCCTTTTCCTTTGAGTTGTGGGTATCTGTAAATTCTGCTAGACCTTTGCTGGAAAACTTGTAAATTCTGC<br>TAGACCCTCTGTAAATTCCGCTAGACCTTTGTGTGTTTTTTTTGTTTATATTCAAGTGGTTATAATTTATAGAATAAAGAAAGAATAAAAA<br>AAGATAAAAAGAATAGATCCCAGCCCTGTGTATAACTCACTACTTTAGTCAGTTCCGCAGTATTACAAAAGGATGTCGCAAACGCTGTT<br>TGCTCCTCTACAAAACAGACCTTAAAACCCTAAAGGCTTAAGTAGCACCCTCGCAAGCTCGGTTGCGGCCGCAATCGGGCAAATCGC	Hoffmann *et al.*, 2017[6]

	TGAATATTCCTTTTGTCTCCGACCATCAGGCACCTGAGTCGCTGTCTTTTTCGTGACATTCAGTTCGCTGCGCTCACGGCTCTGGCAG<br>TGAATGGGGGTAAATGGCACTACAGGCGCCTTTTATGGATTCATGCAAGGAAACTACCCATAATACAAGAAAAGCCCGTCACGGGCTT<br>CTCAGGGCGTTTTATGGCGGGTCTGCTATGTGGTGCTATCTGACTTTTTGCTGTTCAGCAGTTCCTGCCCTCTGATTTTCCAGTCTGAC<br>CACTTCGGATTATCCCGTGACAGGTCATTCAGACTGGCTAATGCACCCAGTAAGGCAGCGGTATCATCAACGGGGTCTGACGCTCAG<br>TGGAACGAAAACTCACGTTAAGGGATTTTGGTCATGAGATTATCAAAAAGGATCTTCACCTAGATCCTTTTAAATTAAAAATGAAGTTTT<br>AAATCAATCTAAAGTATATATGAGTAAACTTGGTCTGACAGTTACCAATGCTTAATCAGTGAGGCACCTATCTCAGCGATCTGTCTATTT<br>CGTTCATCCATAGTTGCCTGACTCCCCGTCGTGTAGATAACTACGATACGGGAGGGCTTACCATCTGGCCCCAGTGCTGCAATGATAC<br>CGCGAGACCCACGCTCACCGGCTCCAGATTTATCAGCAATAAACCAGCCAGCCGGAAGGGCCGAGCGCAGAAGTGGTCCTGCAACT<br>TTATCCGCCTCCATCCAGTCTATTAATTGTTGCCGGGAAGCTAGAGTAAGTAGTTCGCCAGTTAATAGTTTGCGCAACGTTGTTGCCAT<br>TGCTACAGGCATCGTGGTGTCACGCTCGTCGTTTGGTATGGCTTCATTCAGCTCCGGTTCCCAACGATCAAGGCGAGTTACATGATCC<br>CCCATGTTGTGCAAAAAAGCGGTTAGCTCCTTCGGTCCTCCGATCGTTGTCAGAAGTAAGTTGGCCGCAGTGTTATCACTCATGGTTA<br>TGGCAGCACTGCATAATTCTCTTACTGTCATGCCATCCGTAAGATGCTTTTCTGTGACTGGTGAGTACTCAACCAAGTCATTCTGAGAA<br>TAGTGTATGCGGCGACCGAGTTGCTCTTGCCCGGCGTCAATACGGGATAATACCGCGCCACATAGCAGAACTTTAAAAGTGCTCATC<br>ATTGGAAAACGTTCTTCGGGGCGAAAACTCTCAAGGATCTTACCGCTGTTGAGATCCAGTTCGATGTAACCCACTCGTGCACCCAACT<br>GATCTTCAGCATCTTTTACTTTCACCAGCGTTTCTGGGTGAGCAAAAACAGGAAGGCAAAATGCCGCAAAAAAGGGAATAAGGGCGAC<br>ACGGAAATGTTGAATACTCATACTCTTCCTTTTTCAATATTATTGAAGCATTTATCAGGGTTATTGTCTCATGAGCGGATACATATTTGAA<br>TGTATTTAGAAAAATAAACAAATAGGGGTTCCGCGCACATTTCCCCGAAAAGTGCCACCTG	
pL0A_0-1-1	CCGTAGTTAGGCCACCACTTCAAGAACTCTGTAGCACCGCCTACATACCTCGCTCTGCTAATCCTGTTACCAGTGGCTGCTGCCAGTG<br>GCGATAAGTCGTGTCTTACCGGGTTGGACTCAAGACGATAGTTACCGGATAAGGCGCAGCGGTCGGGCTGAACGGGGGGTTCGTGC<br>ACACAGCCCAGCTTGGAGCGAACGACCTACACCGAACTGAGATACCTACAGCGTGAGCTATGAGAAAGCGCCACGCTTCCCGAAGG<br>GAGAAAGGCGGACAGGTATCCGGTAAGCGGCAGGGTCGGAACAGGAGAGCGCACGAGGGAGCTTCCAGGGGGAAACGCCTGGTAT<br>CTTTATAGTCCTGTCGGGTTTCGCCACCTCTGACTTGAGCGTCGATTTTTGTGATGCTCGTCAGGGGGGCGGAGCCTATGGAAAAAC<br>GCCAGCAACGCGGCCTTTTTACGGTTCCTGGCCTTTTGCTGGCCTTTTGCTCATGATATAATTAAATTGAAGCTCTAATTTGTGAGTTT<br>AGTATACATGCATTTACTTATAATACAGTTTTTTAGTTTTGCTGGCCGCATCTTCTCAAATATGCTTCCCAGCCTGCTTTTCTGTAACGTT<br>CACCCTCTACCTTAGCATCCCTTCCCTTTGCAAATAGTCCTCTTCCAACAATAATAATGTCAGATCCTGTAGAGACCACATCATCCACG<br>GTTCTATACTGTTGACCCAATGCGTCTCCCTTGTCATCTAAACCCACACCGGGTGTCATAATCAACCAATCGTAACCTTCATCTCTTCC<br>ACCCATGTCTCTTTGAGCAATAAAGCCGATAACAAAATCTTTGTCGCTCTTCGCAATGTCAACAGTACCCTTAGTATATTCTCCAGTAGA<br>TAGGGAGCCCTTGCATGACAATTCTGCTAACATCAAAAGGCCTCTAGGTTCCTTTGTTACTTCTTCTGCCGCCTGCTTCAAACCGCTAA<br>CAATACCTGGGCCCACCACACCGTGTGCATTCGTAATGTCTGCCCATTCTGCTATTCTGTATACACCCGCAGAGTACTGCAATTTGAC<br>TGTATTACCAATGTCAGCAAATTTTCTGTCTTCGAAGAGTAAAAAATTGTACTTGGCGGATAATGCCTTTAGCGGCTTAACTGTGCCCT<br>CCATGGAAAAATCAGTCAAGATATCCACATGTGTTTTTAGTAAACAAATTTTGGGACCTAATGCTTCAACTAACTCCAGTAATTCCTTGG<br>TGGTACGAACATCCAATGAAGCACACAAGTTTGTTTGCTTTTCGTGCATGATATTAAATAGCTTGGCAGCAACAGGACTAGGATGAGTA<br>GCAGCACGTTCCTTATATGTAGCTTTCGACATGATTTATCTTCGTTTCCTGCAGGTTTTTGTTCTGTGCAGTTGGGTTAAGAATACTGG<br>GCAATTTCATGTTTCTTCAACACTACATATGCGTATATATACCAATCTAAGTCTGTGCTCCTTCCTTCGTTCTTCCTTCTGTTCGGAGATT<br>ACCGAATCAAAAAAATTTCAAAGAAACCGAAATCAAAAAAAAGAATAAAAAAAAAATGATGAATTGAATTGAAAAGCTAGCTTATCGATG<br>ATAAGCTGTCAAAGATGAGAATTAATTCCACGGACTATAGACTATACTAGATACTCCGTCTACTGTACGATACACTTCCGCTCAGGTCC<br>TTGTCCTTTAACGAGGCCTTACCACTCTTTTGTTACTCTATTGATCCAGCTCAGCAAAGGCAGTGTGATCTAAGATTCTATCTTCGCGAT<br>GTAGTAAAACTAGCTAGACCGAGAAAGAGACTAGAAATGCAAAAGGCACTTCTACAATGGCTGCCATCATTATTATCCGATGTGACGC<br>TGCAGCTTCTCAATGATATTCGAATACGCTTTGAGGAGATACAGCCTAATATCCGACAAACTGTTTTACAGATTTACGATCGTACTTGTT<br>ACCCATCATTGAATTTTGAACATCCGAACCTGGGAGTTTTCCCTGAAACAGATAGTATATTTGAACCTGTATAATAATATATAGTCTAGC<br>GCTTTACGGAAGACAATGTATGTATTTCGGTTCCTGGAGAAACTATTGCATCTATTGCATAGGTAATCTTGCACGTCGCATCCCCGGTT<br>CATTTTCTGCGTTTCCATCTTGCACTTCAATAGCATATCTTTGTTAACGAAGCATCTGTGCTTCATTTTGTAGAACAAAAATGCAACGCG<br>AGAGCGCTAATTTTTCAAACAAAGAATCTGAGCTGCATTTTTACAGAACAGAAATGCAACGCGAAAGCGCTATTTTACCAACGAAGAAT<br>CTGTGCTTCATTTTTGTAAAACAAAAATGCAACGCGACGAGAGCGCTAATTTTTCAAACAAAGAATCTGAGCTGCATTTTTACAGAACA<br>GAAATGCAACGCGAGAGCGCTATTTTACCAACAAAGAATCTATACTTCTTTTTTGTTCTACAAAAATGCATCCCGAGAGCGCTATTTTTC<br>TAACAAAGCATCTTAGATTACTTTTTTTCTCCTTTGTGCGCTCTATAATGCAGTCTCTTGATAACTTTTTGCACTGTAGGTCCGTTAAGGT<br>TAGAAGAAGGCTACTTTGGTGTCTATTTTCTCTTCCATAAAAAAAGCCTGACTCCACTTCCCGCGTTTACTGATTACTAGCGAAGCTGC<br>GGGTGCATTTTTTCAAGATAAAGGCATCCCCGATTATATTCTATACCGATGTGGATTGCGCATACTTTGTGAACAGAAAGTGATAGCGT<br>TGATGATTCTTCATTGGTCAGAAAATTATGAACGGTTTCTTCTATTTTGTCTCTATATACTACGTATAGGAAATGTTTACATTTTCGTATT<br>GTTTTCGATTCACTCTATGAATAGTTCTTACTACAATTTTTTTGTCTAAAGAGTAATACTAGAGATAAACATAAAAAATGTAGAGGTCGAG<br>TTTAGATGCAAGTTCAAGGAGCGAAAGGTGGATGGGTAGGTTATATAGGGATATAGCACAGAGATATATAGCAAAGAGATACTTTTGA<br>GCAATGTTTGTGGAAGCGGTATTCGCAATGGGAAGCTCCACCCCGGTTGATAATCAGAAAAGCCCCAAAAACAGGAAGATTGTATAAG<br>CAAATATTTAAATTGTAATTCTTTCCTGCGTTATCCCCTGATTCTGTGGATAACCGTATTACCGCCTTTGAGTGAGCTGATACCGCTCGC<br>CGCAGCCGAACGACCGAGCGCAGCGAGTCAGTGAGCGAGGAAGCGGAAGAGGGTCTGACGCTCAGTGGAACGAAAACTCACGTTA<br>AGGGATTTTGGTCATGAGATTATCAAAAAGGATCTTCACCTAGATCCTTTTAAATTAAAAATGAAGTTTTAAATCAATCTAAAGTATATAT<br>GAGTAAACTTGGTCTGACAGTCAGAAGAACTCGTCAAGAAGGCGATAGAAGGCGATGCGCTGCGAATCGGGAGCGGCGATACCGTA<br>AAGCACGAGGAAGCGGTCAGCCCATTCGCCGCCAAGCTCTTCAGCAATATCACGGGTAGCCAACGCTATGTCCTGATAGCGGTCCG	Naseri *et al.*, 2019[7]

	CCACACCCAGCCGGCCACAGTCGATGAATCCAGAAAAGCGGCCATTTTCCACCATGATATTCGGCAAGCAGGCATCGTCATGGGTCA<br>CGACGAGATCCTCGCCGTCGGGCATGCTCGCCTTGAGCCTGGCGAACAGTTCGGCTGGCGCGAGCCCCTGATGCTCTTCGTCCAGA<br>TCATCCTGATCGACAAGACCGGCTTCCATCCGAGTACGTGCTCGCTCGATGCGATGTTTCGCTTGGTGGTCGAATGGGCAGGTAGCC<br>GGATCAAGCGTATGCAGCCGCCGCATTGCATCAGCCATGATGGATACTTTCTCGGCAGGAGCAAGGTGAGATGACAGGAGATCCTGC<br>CCCGGCACTTCGCCCAATAGCAGCCAGTCCCTTCCCGCTTCAGTGACAACGTCGAGCACAGCTGCGCAAGGAACGCCCGTCGTGGC<br>CAGCCACGATAGCCGCGCTGCCTCGTCTTGCAGTTCATTCAGGGCACCGGACAGGTCGGTCTTGACAAAAAGAACCGGGCGCCCCT<br>GCGCTGACAGCCGGAACACGGCGGCATCAGAGCAGCCGATTGTCTGTTGTGCCCAGTCATAGCCGAATAGCCTCTCCACCCAAGCG<br>GCCGGAGAACCTGCGTGCAATCCATCTTGTTCAATCATACTCTTCCTTTTTCAATATTATTGAAGCATTTATCAGGGTTATTGTCTCATG<br>AGCGGATACATATTTGAATGTATTTAGAAAAATAAACAAATAGGGGTTCCGCGCACATTTCCCCGAAAAGTGCCACCTGACGTCGGAC<br>GGATCGCTTGCCTGTAACTTACACGCGCCTCGTGGCGCGCCGTCGACGCGTTTGAAAGTGCGCAGTGACAGCTTTCAGCTGAGTCG<br>AGCACTTTGGAATCATTTTCGCAGCGTCGGGGAAGTAGTCGTAATACATCTTGTCGAAACCCTCACTAGTTTTTTCGGTTGGTTCAAAG<br>CTCAGCGCTTTATAACGCCCTTTTTGCTTGGGCATAATGCCCAAATAATACAGACCATTTTTGACAAACAAAATAGCTCCGTTATTCTTT<br>TCCCGGTTTACATCCCATCCGCGAGCTAATGTCGGCATTTGAAAGTTTAACTTAAACTTCTCGACAGAATACGGTTTTTTTGTAGCGTA<br>ATTACGGGCCTTGTTGTAAAAAGACAAACTCGGCTCCATTTCAAGTTTGATACCAGTCAAACGTGCGCTAAACTCTGGGTCGACCTCAT<br>TAGACTCGTCCACGGCGAACCAGTCTAACAAATGGTATAAGCCCAGTAAGCTATCAAGTTGAGATTTAAGAATCTCTTTTTCCTCTTGC<br>TTCTTTAAAGTGGTGGGCAGAGGTTGATCCAACGCGGCATGGGCATGCGACAGAATCTCCGACGTTTTTTGTTTGAAGGCTTCCGACA<br>ACTCTTTCCCGGCAGCGCTAATAATCTCCTGAAGATTGATGTCCTCATGCTTTAAGCTCCGTTGCACCTTTTCTTTGGCAGATTTTGTG<br>ATTTTGCCCGTCAGTTCGGAGATGCGCCGCTCATACAGGGCGTTGCGCAATGTATCCCAATGATCGCATAATGCTGATGAGATGGTTT<br>CTAACTTTTTATGTGAGATAAAGATATGAGTCAAGTCAATAGAATTCAACTCATTGAACAAAGCCTCAGCAGTTTCCAATACGTTCTCAT<br>TACGAAGCAGGGTCTTATACTTACAAAATGATTGAATAACCTCCTCGTCTGATTTGAACTCTTCTAAGATAAAACTCAGGGTATTCCGAT<br>CACTAAGGATTTGTTTAAACAGCGGAATGAAACGGTGAGGCAGAGACGCGATGATATGCGCAGTCTCATCATTCTTTTGAATCGCTAA<br>TGCAAGCACCTCATTCAGCCCTTTAATTTTTTCAGTCCCTGCTTCCCGGCTAATGCCCCCAAGAAGTTGGTTATAAAGGTCGATCTGCG<br>TCTGAGTCAACAACTGATTATAAAAGGGAAACGAGAAAACTTCTTCAATCGACGTTGAGACGAAGATTCCGATTGCCTTCTTGACATTT<br>TCAAAGTGTTCCCGTAAAGACGGTACCGCCGTAATAAGGCGTGTAAAGATGTGGCAGTTCTCTTTAAATTTGGGAAAATTATCTTGAAC<br>AATGCGATGTGGGATGGCTGTACTAATGTCCTCGGCCGAAAAGACGTTTTTACGGTTCCGGTAGAACCCCGAAAAATAAGTAGTGAAT<br>TTGTCGAAACTACGTAACAACGCATTCTCATGTTCCGTAGTTGTTACCGTACCCAACTGCTTCAGGACCTTACCGTTGAAAAGTTCTGC<br>CTTGAACAGTCCCTTGTAGATCTCTGCATGACGCTTGTTGATCGCATCAGTCAAGTTGTCCGTCCGGCCGATGAAGTAATCATGAATG<br>GCATTGCGGTAGGTAGCCTGCTCCTCAATTAAGGCATTGCGTGTTTCTTCTGTCTTTTCCTTGCGATAGCTGTCGATCGCCGCAGACA<br>AATTCTCCCAGTCAAGCTGAACAAGCTGAAGGCATTGGTCTGCATAAGTTTTGTAAATACGATCAATAATCGGTTTCAGCTCCTTGTAA<br>TGATCATTACGTGCCTTGTCCTCTTCGATAAACCCTTGTTCCTGAATGTGCTTAAGTGTTTTACCCTGGGGAATCAACTCGAAGCGCAA<br>TGTTTTGGAAACTTGATATAAATTCGTGAAACCCTCGAACTGTGTCATTCTAGAGTTTAAACCCTGCAGGGGCCGGCCCCGTAGAAAA<br>GATCAAAGGATCTTCTTGAGATCCTTTTTTTCTGCGCGTAATCTGCTGCTTGCAAACAAAAAAACCACCGCTACCAGCGGTGGTTTGTT<br>TGCCGGATCAAGAGCTACCAACTCTTTTTCCGAAGGTAACTGGCTTCAGCAGAGCGCAGATACCAAATACTGTTCTTCTAGTGTAG	
pL0A0-1-1_N-dCas9	CCGTAGTTAGGCCACCACTTCAAGAACTCTGTAGCACCGCCTACATACCTCGCTCTGCTAATCCTGTTACCAGTGGCTGCTGCCAGTG<br>GCGATAAGTCGTGTCTTACCGGGTTGGACTCAAGACGATAGTTACCGGATAAGGCGCAGCGGTCGGGCTGAACGGGGGGTTCGTGC<br>ACACAGCCCAGCTTGGAGCGAACGACCTACACCGAACTGAGATACCTACAGCGTGAGCTATGAGAAAGCGCCACGCTTCCCGAAGG<br>GAGAAAGGCGGACAGGTATCCGGTAAGCGGCAGGGTCGGAACAGGAGAGCGCACGAGGGAGCTTCCAGGGGGAAACGCCTGGTAT<br>CTTTATAGTCCTGTCGGGTTTCGCCACCTCTGACTTGAGCGTCGATTTTTGTGATGCTCGTCAGGGGGGCGGAGCCTATGGAAAAAC<br>GCCAGCAACGCGGCCTTTTTACGGTTCCTGGCCTTTTGCTGGCCTTTTGCTCATGATATAATTAAATTGAAGCTCTAATTTGTGAGTTT<br>AGTATACATGCATTTACTTATAATACAGTTTTTTAGTTTTGCTGGCCGCATCTTCTCAAATATGCTTCCCAGCCTGCTTTTCTGTAACGTT<br>CACCCTCTACCTTAGCATCCCTTCCCTTTGCAAATAGTCCTCTTCCAACAATAATAATGTCAGATCCTGTAGAGACCACATCATCCACG<br>GTTCTATACTGTTGACCCAATGCGTCTCCCTTGTCATCTAAACCCACACCGGGTGTCATAATCAACCAATCGTAACCTTCATCTCTTCC<br>ACCCATGTCTCTTTGAGCAATAAAGCCGATAACAAAATCTTTGTCGCTCTTCGCAATGTCAACAGTACCCTTAGTATATTCTCCAGTAGA<br>TAGGGAGCCCTTGCATGACAATTCTGCTAACATCAAAAGGCCTCTAGGTTCCTTTGTTACTTCTTCTGCCGCCTGCTTCAAACCGCTAA<br>CAATACCTGGGCCCACCACACCGTGTGCATTCGTAATGTCTGCCCATTCTGCTATTCTGTATACACCCGCAGAGTACTGCAATTTGAC<br>TGTATTACCAATGTCAGCAAATTTTCTGTCTTCGAAGAGTAAAAAATTGTACTTGGCGGATAATGCCTTTAGCGGCTTAACTGTGCCCT<br>CCATGGAAAAATCAGTCAAGATATCCACATGTGTTTTTAGTAAACAAATTTTGGGACCTAATGCTTCAACTAACTCCAGTAATTCCTTGG<br>TGGTACGAACATCCAATGAAGCACACAAGTTTGTTTGCTTTTCGTGCATGATATTAAATAGCTTGGCAGCAACAGGACTAGGATGAGTA<br>GCAGCACGTTCCTTATATGTAGCTTTCGACATGATTTATCTTCGTTTCCTGCAGGTTTTTGTTCTGTGCAGTTGGGTTAAGAATACTGG<br>GCAATTTCATGTTTCTTCAACACTACATATGCGTATATATACCAATCTAAGTCTGTGCTCCTTCCTTCGTTCTTCCTTCTGTTCGGAGATT<br>ACCGAATCAAAAAAATTTCAAAGAAACCGAAATCAAAAAAAAGAATAAAAAAAAAATGATGAATTGAATTGAAAAGCTAGCTTATCGATG<br>ATAAGCTGTCAAAGATGAGAATTAATTCCACGGACTATAGACTATACTAGATACTCCGTCTACTGTACGATACACTTCCGCTCAGGTCC<br>TTGTCCTTTAACGAGGCCTTACCACTCTTTTGTTACTCTATTGATCCAGCTCAGCAAAGGCAGTGTGATCTAAGATTCTATCTTCGCGAT<br>GTAGTAAAACTAGCTAGACCGAGAAAGAGACTAGAAATGCAAAAGGCACTTCTACAATGGCTGCCATCATTATTATCCGATGTGACGC<br>TGCAGCTTCTCAATGATATTCGAATACGCTTTGAGGAGATACAGCCTAATATCCGACAAACTGTTTTACAGATTTACGATCGTACTTGTT<br>ACCCATCATTGAATTTTGAACATCCGAACCTGGGAGTTTTCCCTGAAACAGATAGTATATTTGAACCTGTATAATAATATATAGTCTAGC<br>GCTTTACGGAAGACAATGTATGTATTTCGGTTCCTGGAGAAACTATTGCATCTATTGCATAGGTAATCTTGCACGTCGCATCCCCGGTT	This study

	CATTTTCTGCGTTTCCATCTTGCACTTCAATAGCATATCTTTGTTAACGAAGCATCTGTGCTTCATTTTGTAGAACAAAAATGCAACGCG<br>AGAGCGCTAATTTTTCAAACAAAGAATCTGAGCTGCATTTTTACAGAACAGAAATGCAACGCGAAAGCGCTATTTTACCAACGAAGAAT<br>CTGTGCTTCATTTTTGTAAAACAAAAATGCAACGCGACGAGAGCGCTAATTTTTCAAACAAAGAATCTGAGCTGCATTTTTACAGAACA<br>GAAATGCAACGCGAGAGCGCTATTTTACCAACAAAGAATCTATACTTCTTTTTTGTTCTACAAAAATGCATCCCGAGAGCGCTATTTTTC<br>TAACAAAGCATCTTAGATTACTTTTTTTCTCCTTTGTGCGCTCTATAATGCAGTCTCTTGATAACTTTTTGCACTGTAGGTCCGTTAAGGT<br>TAGAAGAAGGCTACTTTGGTGTCTATTTTCTCTTCCATAAAAAAAGCCTGACTCCACTTCCCGCGTTTACTGATTACTAGCGAAGCTGC<br>GGGTGCATTTTTTCAAGATAAAGGCATCCCCGATTATATTCTATACCGATGTGGATTGCGCATACTTTGTGAACAGAAAGTGATAGCGT<br>TGATGATTCTTCATTGGTCAGAAAATTATGAACGGTTTCTTCTATTTTGTCTCTATATACTACGTATAGGAAATGTTTACATTTTCGTATT<br>GTTTTCGATTCACTCTATGAATAGTTCTTACTACAATTTTTTTGTCTAAAGAGTAATACTAGAGATAAACATAAAAAATGTAGAGGTCGAG<br>TTTAGATGCAAGTTCAAGGAGCGAAAGGTGGATGGGTAGGTTATATAGGGATATAGCACAGAGATATATAGCAAAGAGATACTTTTGA<br>GCAATGTTTGTGGAAGCGGTATTCGCAATGGGAAGCTCCACCCCGGTTGATAATCAGAAAAGCCCCAAAAACAGGAAGATTGTATAAG<br>CAAATATTTAAATTGTAATTCTTTCCTGCGTTATCCCCTGATTCTGTGGATAACCGTATTACCGCCTTTGAGTGAGCTGATACCGCTCGC<br>CGCAGCCGAACGACCGAGCGCAGCGAGTCAGTGAGCGAGGAAGCGGAAGAGGGTCTGACGCTCAGTGGAACGAAAACTCACGTTA<br>AGGGATTTTGGTCATGAGATTATCAAAAAGGATCTTCACCTAGATCCTTTTAAATTAAAAATGAAGTTTTAAATCAATCTAAAGTATATAT<br>GAGTAAACTTGGTCTGACAGTCAGAAGAACTCGTCAAGAAGGCGATAGAAGGCGATGCGCTGCGAATCGGGAGCGGCGATACCGTA<br>AAGCACGAGGAAGCGGTCAGCCCATTCGCCGCCAAGCTCTTCAGCAATATCACGGGTAGCCAACGCTATGTCCTGATAGCGGTCCG<br>CCACACCCAGCCGGCCACAGTCGATGAATCCAGAAAAGCGGCCATTTTCCACCATGATATTCGGCAAGCAGGCATCGTCATGGGTCA<br>CGACGAGATCCTCGCCGTCGGGCATGCTCGCCTTGAGCCTGGCGAACAGTTCGGCTGGCGCGAGCCCCTGATGCTCTTCGTCCAGA<br>TCATCCTGATCGACAAGACCGGCTTCCATCCGAGTACGTGCTCGCTCGATGCGATGTTTCGCTTGGTGGTCGAATGGGCAGGTAGCC<br>GGATCAAGCGTATGCAGCCGCCGCATTGCATCAGCCATGATGGATACTTTCTCGGCAGGAGCAAGGTGAGATGACAGGAGATCCTGC<br>CCCGGCACTTCGCCCAATAGCAGCCAGTCCCTTCCCGCTTCAGTGACAACGTCGAGCACAGCTGCGCAAGGAACGCCCGTCGTGGC<br>CAGCCACGATAGCCGCGCTGCCTCGTCTTGCAGTTCATTCAGGGCACCGGACAGGTCGGTCTTGACAAAAAGAACCGGGCGCCCCT<br>GCGCTGACAGCCGGAACACGGCGGCATCAGAGCAGCCGATTGTCTGTTGTGCCCAGTCATAGCCGAATAGCCTCTCCACCCAAGCG<br>GCCGGAGAACCTGCGTGCAATCCATCTTGTTCAATCATACTCTTCCTTTTTCAATATTATTGAAGCATTTATCAGGGTTATTGTCTCATG<br>AGCGGATACATATTTGAATGTATTTAGAAAAATAAACAAATAGGGGTTCCGCGCACATTTCCCCGAAAAGTGCCACCTGACGTCGGAC<br>GGATCGCTTGCCTGTAACTTACACGCGCCTCGTGGGGCGCGCCGTCGACATGGATAAGAAGTACTCCATTGGTTTAGCAATTGGCAC<br>GAACAGTGTCGGATGGGCCGTGATTACAGATGAATACAAGGTCCCTTCAAAGAAATTCAAAGTGTTAGGTAATACTGACCGGCACAGT<br>ATTAAGAAAAACCTGATCGGGGCACTTCTGTTCGATTCCGGGGAAACTGCAGAGGCAACACGTTTGAAGCGTACCGCCCGGCGCCGT<br>TATACGCGTCGTAAAAATCGCATCTGTTACTTACAAGAAATCTTCTCAAATGAGATGGCGAAGGTAGACGATTCTTTTTTTCATCGGCT<br>GGAGGAATCGTTTTTGGTAGAAGAGGACAAGAAGCATGAACGCCATCCGATTTTCGGGAATATTGTCGATGAAGTTGCCTATCATGAG<br>AAATATCCGACAATTTACCATTTACGGAAAAAACTTGTGGATTCTACGGACAAAGCTGACTTGCGGTTGATCTATCTTGCCCTGGCCCA<br>CATGATTAAGTTCCGTGGGCACTTTCTTATCGAAGGGGATTTGAACCCAGATAACTCGGACGTTGACAAGTTGTTTATTCAATTGGTCC<br>AAACCTATAACCAGCTTTTCGAGGAAAATCCGATCAATGCTAGCGGTGTGGACGCGAAAGCTATCCTGAGTGCCCGGTTATCTAAATC<br>ACGGCGGCTGGAGAACTTGATTGCCCAGTTGCCCGGAGAGAAGAAAAACGGATTATTCGGTAATCTTATCGCCCTTTCTCTGGGGTT<br>GACGCCCAACTTCAAATCTAACTTTGATCTGGCCGAAGACGCTAAATTACAACTTAGTAAAGATACCTACGACGATGACCTGGATAATT<br>TGTTGGCTCAAATCGGGGATCAATATGCTGACCTTTTCCTTGCGGCGAAGAACTTATCGGACGCCATCTTGCTGTCGGATATCCTGCG<br>TGTCAATACGGAGATTACTAAGGCACCACTTTCCGCATCCATGATCAAGCGTTATGACGAGCATCACCAAGACCTTACGTTGCTGAAA<br>GCCTTAGTCCGTCAACAGTTACCTGAAAAATATAAAGAAATTTTTTTTGATCAGTCTAAGAATGGATACGCAGGTTATATCGACGGAGG<br>AGCAAGCCAAGAAGAATTTTACAAATTTATTAAACCGATTCTTGAAAAGATGGATGGTACGGAGGAACTTCTGGTTAAGTTAAATCGCG<br>AAGACTTGTTGCGCAAGCAGCGCACTTTCGACAACGGGAGTATCCCCCACCAAATTCATCTTGGTGAATTACATGCGATCCTGCGGC<br>GGCAAGAAGATTTTTATCCTTTCTTAAAAGATAATCGCGAAAAAATTGAAAAAATCTTAACGTTTCGCATTCCTTATTACGTGGGCCCTC<br>TGGCACGCGGTAATAGCCGGTTTGCCTGGATGACCCGCAAATCAGAAGAGACCATCACCCCGTGGAATTTCGAGGAGGTAGTAGACA<br>AGGGTGCCAGTGCACAATCCTTCATCGAACGCATGACAAATTTTGATAAAAATTTGCCTAATGAGAAGGTCCTGCCGAAACATAGTCTT<br>TTATATGAATACTTTACCGTATACAACGAGTTGACAAAGGTTAAGTACGTGACGGAGGGGATGCGTAAACCTGCATTTTTGTCAGGTGA<br>GCAAAAAAAAGCGATTGTGGATTTATTATTTAAAACTAATCGGAAGGTGACAGTGAAGCAACTTAAGGAAGACTATTTCAAGAAGATCG<br>AATGCTTCGATTCGGTAGAAATCAGTGGTGTTGAAGACCGCTTCAACGCAAGCTTAGGGACTTATCACGATCTTCTTAAGATCATTAAG<br>GACAAAGACTTCCTGGACAACGAGGAAAACGAGGACATTTTGGAAGATATTGTACTTACTTTGACTTTGTTTGAAGACCGGGAGATGA<br>TCGAAGAGCGTCTTAAGACGTACGCGCACCTTTTCGATGACAAAGTAATGAAGCAGCTGAAGCGGCGGCGGTACACCGGATGGGGT<br>CGTTTATCTCGGAAATTGATCAATTCTAGAGTTTAAACAAACCCTGCAGGGGCCGGCCCCGTAGAAAAGATCAAAGGATCTTCTTGAG<br>ATCCTTTTTTTCTGCGCGTAATCTGCTGCTTGCAAACAAAAAAACCACCGCTACCAGCGGTGGTTTGTTTGCCGGATCAAGAGCTACC<br>AACTCTTTTTCCGAAGGTAACTGGCTTCAGCAGAGCGCAGATACCAAATACTGTTCTTCTAGTGTAG	
pL0A0-1-1_dCas9	CCGTAGTTAGGCCACCACTTCAAGAACTCTGTAGCACCGCCTACATACCTCGCTCTGCTAATCCTGTTACCAGTGGCTGCTGCCAGTG<br>GCGATAAGTCGTGTCTTACCGGGTTGGACTCAAGACGATAGTTACCGGATAAGGCGCAGCGGTCGGGCTGAACGGGGGGTTCGTGC<br>ACACAGCCCAGCTTGGAGCGAACGACCTACACCGAACTGAGATACCTACAGCGTGAGCTATGAGAAAGCGCCACGCTTCCCGAAGG<br>GAGAAAGGCGGACAGGTATCCGGTAAGCGGCAGGGTCGGAACAGGAGAGCGCACGAGGGAGCTTCCAGGGGGAAACGCCTGGTAT<br>CTTTATAGTCCTGTCGGGTTTCGCCACCTCTGACTTGAGCGTCGATTTTTGTGATGCTCGTCAGGGGGGCGGAGCCTATGGAAAAAC<br>GCCAGCAACGCGGCCTTTTTACGGTTCCTGGCCTTTTGCTGGCCTTTTGCTCATGATATAATTAAATTGAAGCTCTAATTTGTGAGTTT	This study

	AGTATACATGCATTTACTTATAATACAGTTTTTTAGTTTTGCTGGCCGCATCTTCTCAAATATGCTTCCCAGCCTGCTTTTCTGTAACGTT<br>CACCCTCTACCTTAGCATCCCTTCCCTTTGCAAATAGTCCTCTTCCAACAATAATAATGTCAGATCCTGTAGAGACCACATCATCCACG<br>GTTCTATACTGTTGACCCAATGCGTCTCCCTTGTCATCTAAACCCACACCGGGTGTCATAATCAACCAATCGTAACCTTCATCTCTTCC<br>ACCCATGTCTCTTTGAGCAATAAAGCCGATAACAAAATCTTTGTCGCTCTTCGCAATGTCAACAGTACCCTTAGTATATTCTCCAGTAGA<br>TAGGGAGCCCTTGCATGACAATTCTGCTAACATCAAAAGGCCTCTAGGTTCCTTTGTTACTTCTTCTGCCGCCTGCTTCAAACCGCTAA<br>CAATACCTGGGCCCACCACACCGTGTGCATTCGTAATGTCTGCCCATTCTGCTATTCTGTATACACCCGCAGAGTACTGCAATTTGAC<br>TGTATTACCAATGTCAGCAAATTTTCTGTCTTCGAAGAGTAAAAAATTGTACTTGGCGGATAATGCCTTTAGCGGCTTAACTGTGCCCT<br>CCATGGAAAAATCAGTCAAGATATCCACATGTGTTTTTAGTAAACAAATTTTGGGACCTAATGCTTCAACTAACTCCAGTAATTCCTTGG<br>TGGTACGAACATCCAATGAAGCACACAAGTTTGTTTGCTTTTCGTGCATGATATTAAATAGCTTGGCAGCAACAGGACTAGGATGAGTA<br>GCAGCACGTTCCTTATATGTAGCTTTCGACATGATTTATCTTCGTTTCCTGCAGGTTTTTGTTCTGTGCAGTTGGGTTAAGAATACTGG<br>GCAATTTCATGTTTCTTCAACACTACATATGCGTATATATACCAATCTAAGTCTGTGCTCCTTCCTTCGTTCTTCCTTCTGTTCGGAGATT<br>ACCGAATCAAAAAAATTTCAAAGAAACCGAAATCAAAAAAAAGAATAAAAAAAAAATGATGAATTGAATTGAAAAGCTAGCTTATCGATG<br>ATAAGCTGTCAAAGATGAGAATTAATTCCACGGACTATAGACTATACTAGATACTCCGTCTACTGTACGATACACTTCCGCTCAGGTCC<br>TTGTCCTTTAACGAGGCCTTACCACTCTTTTGTTACTCTATTGATCCAGCTCAGCAAAGGCAGTGTGATCTAAGATTCTATCTTCGCGAT<br>GTAGTAAAACTAGCTAGACCGAGAAAGAGACTAGAAATGCAAAAGGCACTTCTACAATGGCTGCCATCATTATTATCCGATGTGACGC<br>TGCAGCTTCTCAATGATATTCGAATACGCTTTGAGGAGATACAGCCTAATATCCGACAAACTGTTTTACAGATTTACGATCGTACTTGTT<br>ACCCATCATTGAATTTTGAACATCCGAACCTGGGAGTTTTCCCTGAAACAGATAGTATATTTGAACCTGTATAATAATATATAGTCTAGC<br>GCTTTACGGAAGACAATGTATGTATTTCGGTTCCTGGAGAAACTATTGCATCTATTGCATAGGTAATCTTGCACGTCGCATCCCCGGTT<br>CATTTTCTGCGTTTCCATCTTGCACTTCAATAGCATATCTTTGTTAACGAAGCATCTGTGCTTCATTTTGTAGAACAAAAATGCAACGCG<br>AGAGCGCTAATTTTTCAAACAAAGAATCTGAGCTGCATTTTTACAGAACAGAAATGCAACGCGAAAGCGCTATTTTACCAACGAAGAAT<br>CTGTGCTTCATTTTTGTAAAACAAAAATGCAACGCGACGAGAGCGCTAATTTTTCAAACAAAGAATCTGAGCTGCATTTTTACAGAACA<br>GAAATGCAACGCGAGAGCGCTATTTTACCAACAAAGAATCTATACTTCTTTTTTGTTCTACAAAAATGCATCCCGAGAGCGCTATTTTTC<br>TAACAAAGCATCTTAGATTACTTTTTTTCTCCTTTGTGCGCTCTATAATGCAGTCTCTTGATAACTTTTTGCACTGTAGGTCCGTTAAGGT<br>TAGAAGAAGGCTACTTTGGTGTCTATTTTCTCTTCCATAAAAAAAGCCTGACTCCACTTCCCGCGTTTACTGATTACTAGCGAAGCTGC<br>GGGTGCATTTTTTCAAGATAAAGGCATCCCCGATTATATTCTATACCGATGTGGATTGCGCATACTTTGTGAACAGAAAGTGATAGCGT<br>TGATGATTCTTCATTGGTCAGAAAATTATGAACGGTTTCTTCTATTTTGTCTCTATATACTACGTATAGGAAATGTTTACATTTTCGTATT<br>GTTTTCGATTCACTCTATGAATAGTTCTTACTACAATTTTTTTGTCTAAAGAGTAATACTAGAGATAAACATAAAAAATGTAGAGGTCGAG<br>TTTAGATGCAAGTTCAAGGAGCGAAAGGTGGATGGGTAGGTTATATAGGGATATAGCACAGAGATATATAGCAAAGAGATACTTTTGA<br>GCAATGTTTGTGGAAGCGGTATTCGCAATGGGAAGCTCCACCCCGGTTGATAATCAGAAAAGCCCCAAAAACAGGAAGATTGTATAAG<br>CAAATATTTAAATTGTAATTCTTTCCTGCGTTATCCCCTGATTCTGTGGATAACCGTATTACCGCCTTTGAGTGAGCTGATACCGCTCGC<br>CGCAGCCGAACGACCGAGCGCAGCGAGTCAGTGAGCGAGGAAGCGGAAGAGGGTCTGACGCTCAGTGGAACGAAAACTCACGTTA<br>AGGGATTTTGGTCATGAGATTATCAAAAAGGATCTTCACCTAGATCCTTTTAAATTAAAAATGAAGTTTTAAATCAATCTAAAGTATATAT<br>GAGTAAACTTGGTCTGACAGTCAGAAGAACTCGTCAAGAAGGCGATAGAAGGCGATGCGCTGCGAATCGGGAGCGGCGATACCGTA<br>AAGCACGAGGAAGCGGTCAGCCCATTCGCCGCCAAGCTCTTCAGCAATATCACGGGTAGCCAACGCTATGTCCTGATAGCGGTCCG<br>CCACACCCAGCCGGCCACAGTCGATGAATCCAGAAAAGCGGCCATTTTCCACCATGATATTCGGCAAGCAGGCATCGTCATGGGTCA<br>CGACGAGATCCTCGCCGTCGGGCATGCTCGCCTTGAGCCTGGCGAACAGTTCGGCTGGCGCGAGCCCCTGATGCTCTTCGTCCAGA<br>TCATCCTGATCGACAAGACCGGCTTCCATCCGAGTACGTGCTCGCTCGATGCGATGTTTCGCTTGGTGGTCGAATGGGCAGGTAGCC<br>GGATCAAGCGTATGCAGCCGCCGCATTGCATCAGCCATGATGGATACTTTCTCGGCAGGAGCAAGGTGAGATGACAGGAGATCCTGC<br>CCCGGCACTTCGCCCAATAGCAGCCAGTCCCTTCCCGCTTCAGTGACAACGTCGAGCACAGCTGCGCAAGGAACGCCCGTCGTGGC<br>CAGCCACGATAGCCGCGCTGCCTCGTCTTGCAGTTCATTCAGGGCACCGGACAGGTCGGTCTTGACAAAAAGAACCGGGCGCCCCT<br>GCGCTGACAGCCGGAACACGGCGGCATCAGAGCAGCCGATTGTCTGTTGTGCCCAGTCATAGCCGAATAGCCTCTCCACCCAAGCG<br>GCCGGAGAACCTGCGTGCAATCCATCTTGTTCAATCATACTCTTCCTTTTTCAATATTATTGAAGCATTTATCAGGGTTATTGTCTCATG<br>AGCGGATACATATTTGAATGTATTTAGAAAAATAAACAAATAGGGGTTCCGCGCACATTTCCCCGAAAAGTGCCACCTGACGTCGGAC<br>GGATCGCTTGCCTGTAACTTACACGCGCCTCGTGGGGCGCGCCGTCGACATGGATAAGAAGTACTCCATTGGTTTAGCAATTGGCAC<br>GAACAGTGTCGGATGGGCCGTGATTACAGATGAATACAAGGTCCCTTCAAAGAAATTCAAAGTGTTAGGTAATACTGACCGGCACAGT<br>ATTAAGAAAAACCTGATCGGGGCACTTCTGTTCGATTCCGGGGAAACTGCAGAGGCAACACGTTTGAAGCGTACCGCCCGGCGCCGT<br>TATACGCGTCGTAAAAATCGCATCTGTTACTTACAAGAAATCTTCTCAAATGAGATGGCGAAGGTAGACGATTCTTTTTTTCATCGGCT<br>GGAGGAATCGTTTTTGGTAGAAGAGGACAAGAAGCATGAACGCCATCCGATTTTCGGGAATATTGTCGATGAAGTTGCCTATCATGAG<br>AAATATCCGACAATTTACCATTTACGGAAAAAACTTGTGGATTCTACGGACAAAGCTGACTTGCGGTTGATCTATCTTGCCCTGGCCCA<br>CATGATTAAGTTCCGTGGGCACTTTCTTATCGAAGGGGATTTGAACCCAGATAACTCGGACGTTGACAAGTTGTTTATTCAATTGGTCC<br>AAACCTATAACCAGCTTTTCGAGGAAAATCCGATCAATGCTAGCGGTGTGGACGCGAAAGCTATCCTGAGTGCCCGGTTATCTAAATC<br>ACGGCGGCTGGAGAACTTGATTGCCCAGTTGCCCGGAGAGAAGAAAAACGGATTATTCGGTAATCTTATCGCCCTTTCTCTGGGGTT<br>GACGCCCAACTTCAAATCTAACTTTGATCTGGCCGAAGACGCTAAATTACAACTTAGTAAAGATACCTACGACGATGACCTGGATAATT<br>TGTTGGCTCAAATCGGGGATCAATATGCTGACCTTTTCCTTGCGGCGAAGAACTTATCGGACGCCATCTTGCTGTCGGATATCCTGCG<br>TGTCAATACGGAGATTACTAAGGCACCACTTTCCGCATCCATGATCAAGCGTTATGACGAGCATCACCAAGACCTTACGTTGCTGAAA<br>GCCTTAGTCCGTCAACAGTTACCTGAAAAATATAAAGAAATTTTTTTTGATCAGTCTAAGAATGGATACGCAGGTTATATCGACGGAGG<br>AGCAAGCCAAGAAGAATTTTACAAATTTATTAAACCGATTCTTGAAAAGATGGATGGTACGGAGGAACTTCTGGTTAAGTTAAATCGCG	

	AAGACTTGTTGCGCAAGCAGCGCACTTTCGACAACGGGAGTATCCCCCACCAAATTCATCTTGGTGAATTACATGCGATCCTGCGGC<br>GGCAAGAAGATTTTTATCCTTTCTTAAAAGATAATCGCGAAAAAATTGAAAAAATCTTAACGTTTCGCATTCCTTATTACGTGGGCCCTC<br>TGGCACGCGGTAATAGCCGGTTTGCCTGGATGACCCGCAAATCAGAAGAGACCATCACCCCGTGGAATTTCGAGGAGGTAGTAGACA<br>AGGGTGCCAGTGCACAATCCTTCATCGAACGCATGACAAATTTTGATAAAAATTTGCCTAATGAGAAGGTCCTGCCGAAACATAGTCTT<br>TTATATGAATACTTTACCGTATACAACGAGTTGACAAAGGTTAAGTACGTGACGGAGGGGATGCGTAAACCTGCATTTTTGTCAGGTGA<br>GCAAAAAAAAGCGATTGTGGATTTATTATTTAAAACTAATCGGAAGGTGACAGTGAAGCAACTTAAGGAAGACTATTTCAAGAAGATCG<br>AATGCTTCGATTCGGTAGAAATCAGTGGTGTTGAAGACCGCTTCAACGCAAGCTTAGGGACTTATCACGATCTTCTTAAGATCATTAAG<br>GACAAAGACTTCCTGGACAACGAGGAAAACGAGGACATTTTGGAAGATATTGTACTTACTTTGACTTTGTTTGAAGACCGGGAGATGA<br>TCGAAGAGCGTCTTAAGACGTACGCGCACCTTTTCGATGACAAAGTAATGAAGCAGCTGAAGCGGCGGCGGTACACCGGATGGGGT<br>CGTTTATCTCGGAAATTGATCAATGGCATTCGTGACAAGCAAAGCGGCAAGACGATCTTAGATTTCCTTAAGTCCGATGGATTTGCTAA<br>TCGGAACTTTATGCAATTGATTCATGACGATAGTCTTACCTTTAAAGAGGACATCCAAAAGGCACAAGTGTCAGGCCAAGGTGACAGT<br>CTTCACGAACATATTGCTAACTTAGCCGGTAGTCCAGCTATCAAGAAGGGTATCTTACAGACTGTTAAAGTAGTAGATGAACTGGTGAA<br>AGTAATGGGTCGTCACAAGCCTGAGAATATCGTAATTGAAATGGCGCGGGAAAATCAGACAACTCAAAAAGGTCAAAAAAACAGTCGT<br>GAACGTATGAAACGCATTGAAGAGGGCATCAAGGAGTTGGGCAGTCAGATTTTAAAGGAGCATCCAGTGGAGAACACTCAGCTGCAA<br>AATGAGAAACTTTATCTTTATTACCTTCAGAATGGACGCGATATGTACGTCGATCAAGAACTGGATATTAATCGGTTATCCGATTACGAC<br>GTTGATGCCATCGTCCCTCAATCATTTCTGAAAGACGATAGCATCGACAATAAGGTGCTGACACGTAGTGACAAGAACCGTGGGAAGA<br>GCGACAACGTACCGTCGGAGGAGGTCGTTAAGAAGATGAAGAACTATTGGCGGCAATTGCTGAACGCTAAATTGATTACGCAGCGGA<br>AATTTGACAACCTGACCAAGGCAGAGCGTGGGGGCCTGAGTGAACTGGATAAAGCTGGCTTTATCAAACGTCAATTGGTAGAGACAC<br>GCCAAATTACGAAGCATGTCGCGCAGATTTTGGACTCGCGCATGAACACTAAGTACGATGAAAACGACAAGTTAATTCGTGAGGTGAA<br>AGTCATTACTCTTAAGAGTAAACTTGTCTCGGACTTCCGGAAGGACTTTCAATTTTACAAAGTCCGTGAAATTAATAACTATCATCACGC<br>CCACGACGCATATCTTAACGCGGTCGTTGGAACAGCTTTGATTAAGAAATATCCCAAGCTTGAGAGCGAGTTCGTGTACGGTGATTAT<br>AAAGTGTATGACGTACGTAAGATGATCGCGAAGTCCGAACAAGAGATTGGGAAGGCAACTGCTAAGTACTTTTTTTACTCTAATATTAT<br>GAACTTTTTCAAAACCGAGATCACATTGGCTAATGGCGAAATCCGTAAGCGCCCTCTTATTGAGACAAACGGGGAAACTGGGGAGATC<br>GTGTGGGATAAGGGCCGCGATTTTGCAACTGTCCGGAAGGTCCTGTCTATGCCCCAAGTGAACATCGTTAAGAAGACGGAAGTTCAG<br>ACCGGGGGCTTTTCAAAGGAGAGTATTTTACCCAAGCGCAACAGTGACAAGTTGATCGCGCGGAAGAAGGACTGGGATCCCAAAAAG<br>TATGGGGGATTCGACTCACCGACCGTTGCATACTCCGTGCTGGTCGTCGCGAAAGTGGAGAAGGGCAAGAGTAAAAAACTGAAGTCG<br>GTTAAGGAGTTGCTTGGCATCACTATTATGGAACGTAGTAGCTTCGAGAAGAATCCAATCGATTTTTTAGAAGCCAAGGGCTACAAAGA<br>AGTTAAGAAAGACCTTATTATCAAGCTGCCAAAGTACTCACTTTTCGAATTAGAAAACGGACGTAAGCGCATGTTAGCGTCCGCTGGT<br>GAACTGCAAAAAGGAAATGAGCTGGCACTGCCTTCTAAATACGTGAACTTTTTATATTTGGCGTCCCATTACGAAAAGCTTAAGGGGTC<br>GCCCGAAGATAACGAGCAAAAGCAGCTGTTTGTGGAACAACACAAGCATTATCTGGATGAGATCATCGAGCAGATTAGCGAATTCTCA<br>AAGCGGGTGATCTTAGCCGATGCTAACTTGGATAAAGTGCTGTCCGCTTACAACAAACATCGCGATAAGCCCATCCGTGAGCAGGCG<br>GAGAATATCATTCATCTGTTTACATTGACGAATTTGGGGGCCCCCGCTGCATTCAAGTACTTCGATACTACAATCGACCGTAAACGTTA<br>CACTAGTACAAAAGAAGTTTTGGACGCTACCCTGATCCATCAGAGTATCACTGGCCTGTACGAGACACGCATCGACTTGTCCCAATTG<br>GGGGGGGATTAACTCGAGTAAGGATCTCCAGGCATCAAATAAAACGAAAGGCTCAGTCGAAAGACTGGGCCTTTCGTTTTATCTGTTG<br>TTTGTCGGTGAACGCTCTCTACTAGAGTCACACTGGCTCACCTTCGGGTGGGCCTTTCTGCGTTTATAAAACCCTGCAGGGGCCGGC<br>CCCGTAGAAAAGATCAAAGGATCTTCTTGAGATCCTTTTTTTCTGCGCGTAATCTGCTGCTTGCAAACAAAAAAACCACCGCTACCAGC<br>GGTGGTTTGTTTGCCGGATCAAGAGCTACCAACTCTTTTTCCGAAGGTAACTGGCTTCAGCAGAGCGCAGATACCAAATACTGTTCTT<br>CTAGTGTAG	
placI-<br>araC-<br>PBAD-<br>RBS	TTGCCTGTAACTTACACGCGCCTCGTGGGGCGCGCCCCGCCAACACCCGCTGACGCGCCCTGACGGGCTTGTCTGCTCCCGGCATC<br>CGCTTACAGACAAGCTGTGACCGTCTCCGGGAGCTGCATGTGTCAGAGGTTTTCACCGTCATCACCGAAACGCGCGAGGCAGCAGAT<br>CAATTCGCGCGCGAAGGCGAAGCGGCATGCATTTACGTTGACACCATCGAATGGTGCAAAACCTTTCGCGGTATGGCATGATAGCGC<br>CCGGAAGAGAGTCAATTCAGGGTGGTGAATGTGAAACCAGTAACGTTATACGATGTCGCAGAGTATGCCGGTGTCTCTTATCAGACC<br>GTTTCCCGCGTGGTGAACCAGGCCAGCCACGTTTCTGCGAAAACGCGGGAAAAAGTGGAAGCGGCGATGGCGGAGCTGAATTACAT<br>TCCCAACCGCGTGGCACAACAACTGGCGGGCAAACAGTCGTTGCTGATTGGCGTTGCCACCTCCAGTCTGGCCCTGCACGCGCCGT<br>CGCAAATTGTCGCGGCGATTAAATCTCGCGCCGATCAACTGGGTGCCAGCGTGGTGGTGTCGATGGTAGAACGAAGCGGCGTCGAA<br>GCCTGTAAAGCGGCGGTGCACAATCTTCTCGCGCAACGCGTCAGTGGGCTGATCATTAACTATCCGCTGGATGACCAGGATGCCATT<br>GCTGTGGAAGCTGCCTGCACTAATGTTCCGGCGTTATTTCTTGATGTCTCTGACCAGACACCCATCAACAGTATTATTTTCTCCCATGA<br>AGACGGTACGCGACTGGGCGTGGAGCATCTGGTCGCATTGGGTCACCAGCAAATCGCGCTGTTAGCGGGCCCATTAAGTTCTGTCT<br>CGGCGCGTCTGCGTCTGGCTGGCTGGCATAAATATCTCACTCGCAATCAAATTCAGCCGATAGCGGAACGGGAAGGCGACTGGAGT<br>GCCATGTCCGGTTTTCAACAAACCATGCAAATGCTGAATGAGGGCATCGTTCCCACTGCGATGCTGGTTGCCAACGATCAGATGGCG<br>CTGGGCGCAATGCGCGCCATTACCGAGTCCGGGCTGCGCGTTGGTGCGGATATCTCGGTAGTGGGATACGACGATACCGAAGACAG<br>CTCATGTTATATCCCGCCGTCAACCACCATCAAACAGGATTTTCGCCTGCTGGGGCAAACCAGCGTGGACCGCTTGCTGCAACTCTCT<br>CAGGGCCAGGCGGTGAAGGGCAATCAGCTGTTGCCCGTCTCACTGGTGAAAAGAAAAACCACCCTGGCGCCCAATACGCAAACCGC<br>CTCTCCCCGCGCGTTGGCCGATTCATTAATGCAGCTGGCACGACAGGTTTCCCGACTGGAAAGCGGGCAGTGAGCGCAACGCAATT<br>AATGTGAGTTAGCGCGAATTGATCTGGTTTGACAGCTTATCATCGACTGCACGGTGCACCAATGCTTCTGGCGTCAGGCAGCCATCG<br>GAAGCTGTGGTATGGCTGTGCAGGTCGTAAATCACTGCATAATTCGTGTCGCTCAAGGCGCACTCCCGTTCTGGATAATGTTTTTTGC<br>GCCGACATCATAACGGTTCTGGCAAATATTCTGAAATGAGCTGTTGACAATTAATCATCCGGCTCGTATAATGTGTTTATGACAACTTG	ATG<br>Biosynth<br>etics

	ACGGCTACATCATTCACTTTTTCTTCACAACCGGCACGGAACTCGCTCGGGCTGGCCCCGGTGCATTTTTTAAATACCCGCGAGAAAT<br>AGAGTTGATCGTCAAAACCAACATTGCGACCGACGGTGGCGATAGGCATCCGGGTGGTGCTCAAAAGCAGCTTCGCCTGGCTGATAC<br>GTTGGTCCTCGCGCCAGCTTAAGACGCTAATCCCTAACTGCTGGCGGAAAAGATGTGACAGACGCGACGGCGACAAGCAAACATGCT<br>GTGCGACGCTGGCGATATCAAAATTGCTGTCTGCCAGGTGATCGCTGATGTACTGACAAGCCTCGCGTACCCGATTATCCATCGGTG<br>GATGGAGCGACTCGTTAATCGCTTCCATGCGCCGCAGTAACAATTGCTCAAGCAGATTTATCGCCAGCAGCTCCGAATAGCGCCCTT<br>CCCCTTGCCCGGCGTTAATGATTTGCCCAAACAGGTCGCTGAAATGCGGCTGGTGCGCTTCATCCGGGCGAAAGAACCCCGTATTGG<br>CAAATATTGACGGCCAGTTAAGCCATTCATGCCAGTAGGCGCGCGGACGAAAGTAAACCCACTGGTGATACCATTCGCGAGCCTCCG<br>GATGACGACCGTAGTGATGAATCTCTCCTGGCGGGAACAGCAAAATATCACCCGGTCGGCAAACAAATTCTCGTCCCTGATTTTTCAC<br>CACCCCCTGACCGCGAATGGTGAGATTGAGAATATAACCTTTCATTCCCAGCGGTCGGTCGATAAAAAAATCGAGATAACCGTTGGCC<br>TCAATCGGCGTTAAACCCGCCACCAGATGGGCATTAAACGAGTATCCCGGCAGCAGGGGATCATTTTGCGCTTCAGCCATACTTTTCA<br>TACTCCCGCCATTCAGAGAAGAAACCAATTGTCCATATTGCATCAGACATTGCCGTCACTGCGTCTTTTACTGGCTCTTCTCGCTAACC<br>AAACCGGTAACCCCGCTTATTAAAAGCATTCTGTAACAAAGCGGGACCAAAGCCATGACAAAAACGCGTAACAAAAGTGTCTATAATC<br>ACGGCAGAAAAGTCCACATTGATTATTTGCACGGCGTCACACTTTGCTATGCCATAGCATTTTTATCCATAAGATTAGCGGATTCTACC<br>TGACGCTTTTTATCGCAACTCTCTACTGTTTCTCCATACCCGTTTTTTTGGGAATTCAAAAGATCTAAAGAGGAGAAAGGATCTATGGAT<br>AAGAAGTACTCCATTGGTTTAGCAATTGGC	
pL0A-1-1_PBAD_dCas9	CCGTAGTTAGGCCACCACTTCAAGAACTCTGTAGCACCGCCTACATACCTCGCTCTGCTAATCCTGTTACCAGTGGCTGCTGCCAGTG<br>GCGATAAGTCGTGTCTTACCGGGTTGGACTCAAGACGATAGTTACCGGATAAGGCGCAGCGGTCGGGCTGAACGGGGGGTTCGTGC<br>ACACAGCCCAGCTTGGAGCGAACGACCTACACCGAACTGAGATACCTACAGCGTGAGCTATGAGAAAGCGCCACGCTTCCCGAAGG<br>GAGAAAGGCGGACAGGTATCCGGTAAGCGGCAGGGTCGGAACAGGAGAGCGCACGAGGGAGCTTCCAGGGGGAAACGCCTGGTAT<br>CTTTATAGTCCTGTCGGGTTTCGCCACCTCTGACTTGAGCGTCGATTTTTGTGATGCTCGTCAGGGGGGCGGAGCCTATGGAAAAAC<br>GCCAGCAACGCGGCCTTTTTACGGTTCCTGGCCTTTTGCTGGCCTTTTGCTCATGATATAATTAAATTGAAGCTCTAATTTGTGAGTTT<br>AGTATACATGCATTTACTTATAATACAGTTTTTTAGTTTTGCTGGCCGCATCTTCTCAAATATGCTTCCCAGCCTGCTTTTCTGTAACGTT<br>CACCCTCTACCTTAGCATCCCTTCCCTTTGCAAATAGTCCTCTTCCAACAATAATAATGTCAGATCCTGTAGAGACCACATCATCCACG<br>GTTCTATACTGTTGACCCAATGCGTCTCCCTTGTCATCTAAACCCACACCGGGTGTCATAATCAACCAATCGTAACCTTCATCTCTTCC<br>ACCCATGTCTCTTTGAGCAATAAAGCCGATAACAAAATCTTTGTCGCTCTTCGCAATGTCAACAGTACCCTTAGTATATTCTCCAGTAGA<br>TAGGGAGCCCTTGCATGACAATTCTGCTAACATCAAAAGGCCTCTAGGTTCCTTTGTTACTTCTTCTGCCGCCTGCTTCAAACCGCTAA<br>CAATACCTGGGCCCACCACACCGTGTGCATTCGTAATGTCTGCCCATTCTGCTATTCTGTATACACCCGCAGAGTACTGCAATTTGAC<br>TGTATTACCAATGTCAGCAAATTTTCTGTCTTCGAAGAGTAAAAAATTGTACTTGGCGGATAATGCCTTTAGCGGCTTAACTGTGCCCT<br>CCATGGAAAAATCAGTCAAGATATCCACATGTGTTTTTAGTAAACAAATTTTGGGACCTAATGCTTCAACTAACTCCAGTAATTCCTTGG<br>TGGTACGAACATCCAATGAAGCACACAAGTTTGTTTGCTTTTCGTGCATGATATTAAATAGCTTGGCAGCAACAGGACTAGGATGAGTA<br>GCAGCACGTTCCTTATATGTAGCTTTCGACATGATTTATCTTCGTTTCCTGCAGGTTTTTGTTCTGTGCAGTTGGGTTAAGAATACTGG<br>GCAATTTCATGTTTCTTCAACACTACATATGCGTATATATACCAATCTAAGTCTGTGCTCCTTCCTTCGTTCTTCCTTCTGTTCGGAGATT<br>ACCGAATCAAAAAAATTTCAAAGAAACCGAAATCAAAAAAAAGAATAAAAAAAAAAATGATGAATTGAATTGAAAAGCTAGCTTATCGATG<br>ATAAGCTGTCAAAGATGAGAATTAATTCCACGGACTATAGACTATACTAGATACTCCGTCTACTGTACGATACACTTCCGCTCAGGTCC<br>TTGTCCTTTAACGAGGCCTTACCACTCTTTTGTTACTCTATTGATCCAGCTCAGCAAAGGCAGTGTGATCTAAGATTCTATCTTCGCGAT<br>GTAGTAAAACTAGCTAGACCGAGAAAGAGACTAGAAATGCAAAAGGCACTTCTACAATGGCTGCCATCATTATTATCCGATGTGACGC<br>TGCAGCTTCTCAATGATATTCGAATACGCTTTGAGGAGATACAGCCTAATATCCGACAAACTGTTTTACAGATTTACGATCGTACTTGTT<br>ACCCATCATTGAATTTTGAACATCCGAACCTGGGAGTTTTCCCTGAAACAGATAGTATATTTGAACCTGTATAATAATATATAGTCTAGC<br>GCTTTACGGAAGACAATGTATGTATTTCGGTTCCTGGAGAAACTATTGCATCTATTGCATAGGTAATCTTGCACGTCGCATCCCCGGTT<br>CATTTTCTGCGTTTCCATCTTGCACTTCAATAGCATATCTTTGTTAACGAAGCATCTGTGCTTCATTTTGTAGAACAAAAATGCAACGCG<br>AGAGCGCTAATTTTTCAAACAAAGAATCTGAGCTGCATTTTTACAGAACAGAAATGCAACGCGAAAGCGCTATTTTACCAACGAAGAAT<br>CTGTGCTTCATTTTTGTAAAACAAAAATGCAACGCGACGAGAGCGCTAATTTTTCAAACAAAGAATCTGAGCTGCATTTTTACAGAACA<br>GAAATGCAACGCGAGAGCGCTATTTTACCAACAAAGAATCTATACTTCTTTTTTGTTCTACAAAAATGCATCCCGAGAGCGCTATTTTTC<br>TAACAAAGCATCTTAGATTACTTTTTTTCTCCTTTGTGCGCTCTATAATGCAGTCTCTTGATAACTTTTTGCACTGTAGGTCCGTTAAGGT<br>TAGAAGAAGGCTACTTTGGTGTCTATTTTCTCTTCCATAAAAAAAGCCTGACTCCACTTCCCGCGTTTACTGATTACTAGCGAAGCTGC<br>GGGTGCATTTTTTCAAGATAAAGGCATCCCCGATTATATTCTATACCGATGTGGATTGCGCATACTTTGTGAACAGAAAGTGATAGCGT<br>TGATGATTCTTCATTGGTCAGAAAATTATGAACGGTTTCTTCTATTTTGTCTCTATATACTACGTATAGGAAATGTTTACATTTTCGTATT<br>GTTTTCGATTCACTCTATGAATAGTTCTTACTACAATTTTTTTGTCTAAAGAGTAATACTAGAGATAAACATAAAAAATGTAGAGGTCGAG<br>TTTAGATGCAAGTTCAAGGAGCGAAAGGTGGATGGGTAGGTTATATAGGGATATAGCACAGAGATATATAGCAAAGAGATACTTTTGA<br>GCAATGTTTGTGGAAGCGGTATTCGCAATGGGAAGCTCCACCCCGGTTGATAATCAGAAAAGCCCCAAAAACAGGAAGATTGTATAAG<br>CAAATATTTAAATTGTAATTCTTTCCTGCGTTATCCCCTGATTCTGTGGATAACCGTATTACCGCCTTTGAGTGAGCTGATACCGCTCGC<br>CGCAGCCGAACGACCGAGCGCAGCGAGTCAGTGAGCGAGGAAGCGGAAGAGGGTCTGACGCTCAGTGGAACGAAAACTCACGTTA<br>AGGGATTTTGGTCATGAGATTATCAAAAAGGATCTTCACCTAGATCCTTTTAAATTAAAAATGAAGTTTTAAATCAATCTAAAGTATATAT<br>GAGTAAACTTGGTCTGACAGTCAGAAGAACTCGTCAAGAAGGCGATAGAAGGCGATGCGCTGCGAATCGGGAGCGGCGATACCGTA<br>AAGCACGAGGAAGCGGTCAGCCCATTCGCCGCCAAGCTCTTCAGCAATATCACGGGTAGCCAACGCTATGTCCTGATAGCGGTCCG<br>CCACACCCAGCCGGCCACAGTCGATGAATCCAGAAAAGCGGCCATTTTCCACCATGATATTCGGCAAGCAGGCATCGTCATGGGTCA<br>CGACGAGATCCTCGCCGTCGGGCATGCTCGCCTTGAGCCTGGCGAACAGTTCGGCTGGCGCGAGCCCCTGATGCTCTTCGTCCAGA	This study

	TCATCCTGATCGACAAGACCGGCTTCCATCCGAGTACGTGCTCGCTCGATGCGATGTTTCGCTTGGTGGTCGAATGGGCAGGTAGCC<br>GGATCAAGCGTATGCAGCCGCCGCATTGCATCAGCCATGATGGATACTTTCTCGGCAGGAGCAAGGTGAGATGACAGGAGATCCTGC<br>CCCGGCACTTCGCCCAATAGCAGCCAGTCCCTTCCCGCTTCAGTGACAACGTCGAGCACAGCTGCGCAAGGAACGCCCGTCGTGGC<br>CAGCCACGATAGCCGCGCTGCCTCGTCTTGCAGTTCATTCAGGGCACCGGACAGGTCGGTCTTGACAAAAAGAACCGGGCGCCCCT<br>GCGCTGACAGCCGGAACACGGCGGCATCAGAGCAGCCGATTGTCTGTTGTGCCCAGTCATAGCCGAATAGCCTCTCCACCCAAGCG<br>GCCGGAGAACCTGCGTGCAATCCATCTTGTTCAATCATACTCTTCCTTTTTCAATATTATTGAAGCATTTATCAGGGTTATTGTCTCATG<br>AGCGGATACATATTTGAATGTATTTAGAAAAATAAACAAATAGGGGTTCCGCGCACATTTCCCCGAAAAGTGCCACCTGACGTCGGAC<br>GGATCGCTTGCCTGTAACTTACACGCGCCTCGTGGGGCGCGCCCCGCCAACACCCGCTGACGCGCCCTGACGGGCTTGTCTGCTCC<br>CGGCATCCGCTTACAGACAAGCTGTGACCGTCTCCGGGAGCTGCATGTGTCAGAGGTTTTCACCGTCATCACCGAAACGCGCGAGG<br>CAGCAGATCAATTCGCGCGCGAAGGCGAAGCGGCATGCATTTACGTTGACACCATCGAATGGTGCAAAACCTTTCGCGGTATGGCAT<br>GATAGCGCCCGGAAGAGAGTCAATTCAGGGTGGTGAATGTGAAACCAGTAACGTTATACGATGTCGCAGAGTATGCCGGTGTCTCTT<br>ATCAGACCGTTTCCCGCGTGGTGAACCAGGCCAGCCACGTTTCTGCGAAAACGCGGGAAAAAGTGGAAGCGGCGATGGCGGAGCTG<br>AATTACATTCCCAACCGCGTGGCACAACAACTGGCGGGCAAACAGTCGTTGCTGATTGGCGTTGCCACCTCCAGTCTGGCCCTGCAC<br>GCGCCGTCGCAAATTGTCGCGGCGATTAAATCTCGCGCCGATCAACTGGGTGCCAGCGTGGTGGTGTCGATGGTAGAACGAAGCGG<br>CGTCGAAGCCTGTAAAGCGGCGGTGCACAATCTTCTCGCGCAACGCGTCAGTGGGCTGATCATTAACTATCCGCTGGATGACCAGGA<br>TGCCATTGCTGTGGAAGCTGCCTGCACTAATGTTCCGGCGTTATTTCTTGATGTCTCTGACCAGACACCCATCAACAGTATTATTTTCT<br>CCCATGAAGACGGTACGCGACTGGGCGTGGAGCATCTGGTCGCATTGGGTCACCAGCAAATCGCGCTGTTAGCGGGCCCATTAAGT<br>TCTGTCTCGGCGCGTCTGCGTCTGGCTGGCTGGCATAAATATCTCACTCGCAATCAAATTCAGCCGATAGCGGAACGGGAAGGCGAC<br>TGGAGTGCCATGTCCGGTTTTCAACAAACCATGCAAATGCTGAATGAGGGCATCGTTCCCACTGCGATGCTGGTTGCCAACGATCAG<br>ATGGCGCTGGGCGCAATGCGCGCCATTACCGAGTCCGGGCTGCGCGTTGGTGCGGATATCTCGGTAGTGGGATACGACGATACCGA<br>AGACAGCTCATGTTATATCCCGCCGTCAACCACCATCAAACAGGATTTTCGCCTGCTGGGGCAAACCAGCGTGGACCGCTTGCTGCA<br>ACTCTCTCAGGGCCAGGCGGTGAAGGGCAATCAGCTGTTGCCCGTCTCACTGGTGAAAAGAAAAACCACCCTGGCGCCCAATACGC<br>AAACCGCCTCTCCCCGCGCGTTGGCCGATTCATTAATGCAGCTGGCACGACAGGTTTCCCGACTGGAAAGCGGGCAGTGAGCGCAA<br>CGCAATTAATGTGAGTTAGCGCGAATTGATCTGGTTTGACAGCTTATCATCGACTGCACGGTGCACCAATGCTTCTGGCGTCAGGCAG<br>CCATCGGAAGCTGTGGTATGGCTGTGCAGGTCGTAAATCACTGCATAATTCGTGTCGCTCAAGGCGCACTCCCGTTCTGGATAATGTT<br>TTTTGCGCCGACATCATAACGGTTCTGGCAAATATTCTGAAATGAGCTGTTGACAATTAATCATCCGGCTCGTATAATGTGTTTATGAC<br>AACTTGACGGCTACATCATTCACTTTTTCTTCACAACCGGCACGGAACTCGCTCGGGCTGGCCCCGGTGCATTTTTTAAATACCCGCG<br>AGAAATAGAGTTGATCGTCAAAACCAACATTGCGACCGACGGTGGCGATAGGCATCCGGGTGGTGCTCAAAAGCAGCTTCGCCTGGC<br>TGATACGTTGGTCCTCGCGCCAGCTTAAGACGCTAATCCCTAACTGCTGGCGGAAAAGATGTGACAGACGCGACGGCGACAAGCAAA<br>CATGCTGTGCGACGCTGGCGATATCAAAATTGCTGTCTGCCAGGTGATCGCTGATGTACTGACAAGCCTCGCGTACCCGATTATCCAT<br>CGGTGGATGGAGCGACTCGTTAATCGCTTCCATGCGCCGCAGTAACAATTGCTCAAGCAGATTTATCGCCAGCAGCTCCGAATAGCG<br>CCCTTCCCCTTGCCCGGCGTTAATGATTTGCCCAAACAGGTCGCTGAAATGCGGCTGGTGCGCTTCATCCGGGCGAAAGAACCCCGT<br>ATTGGCAAATATTGACGGCCAGTTAAGCCATTCATGCCAGTAGGCGCGCGGACGAAAGTAAACCCACTGGTGATACCATTCGCGAGC<br>CTCCGGATGACGACCGTAGTGATGAATCTCTCCTGGCGGGAACAGCAAAATATCACCCGGTCGGCAAACAAATTCTCGTCCCTGATTT<br>TTCACCACCCCCTGACCGCGAATGGTGAGATTGAGAATATAACCTTTCATTCCCAGCGGTCGGTCGATAAAAAAATCGAGATAACCGT<br>TGGCCTCAATCGGCGTTAAACCCGCCACCAGATGGGCATTAAACGAGTATCCCGGCAGCAGGGGATCATTTTGCGCTTCAGCCATAC<br>TTTTCATACTCCCGCCATTCAGAGAAGAAACCAATTGTCCATATTGCATCAGACATTGCCGTCACTGCGTCTTTTACTGGCTCTTCTCG<br>CTAACCAAACCGGTAACCCCGCTTATTAAAAGCATTCTGTAACAAAGCGGGACCAAAGCCATGACAAAAACGCGTAACAAAAGTGTCT<br>ATAATCACGGCAGAAAAGTCCACATTGATTATTTGCACGGCGTCACACTTTGCTATGCCATAGCATTTTTATCCATAAGATTAGCGGAT<br>TCTACCTGACGCTTTTTATCGCAACTCTCTACTGTTTCTCCATACCCGTTTTTTTGGGAATTCAAAAGATCTAAAGAGGAGAAAGGATCT<br>ATGGATAAGAAGTACTCCATTGGTTTAGCAATTGGCACGAACAGTGTCGGATGGGCCGTGATTACAGATGAATACAAGGTCCCTTCAA<br>AGAAATTCAAAGTGTTAGGTAATACTGACCGGCACAGTATTAAGAAAAACCTGATCGGGGCACTTCTGTTCGATTCCGGGGAAACTGC<br>AGAGGCAACACGTTTGAAGCGTACCGCCCGGCGCCGTTATACGCGTCGTAAAAATCGCATCTGTTACTTACAAGAAATCTTCTCAAAT<br>GAGATGGCGAAGGTAGACGATTCTTTTTTTCATCGGCTGGAGGAATCGTTTTTGGTAGAAGAGGACAAGAAGCATGAACGCCATCCGA<br>TTTTCGGGAATATTGTCGATGAAGTTGCCTATCATGAGAAATATCCGACAATTTACCATTTACGGAAAAAACTTGTGGATTCTACGGAC<br>AAAGCTGACTTGCGGTTGATCTATCTTGCCCTGGCCCACATGATTAAGTTCCGTGGGCACTTTCTTATCGAAGGGGATTTGAACCCAG<br>ATAACTCGGACGTTGACAAGTTGTTTATTCAATTGGTCCAAACCTATAACCAGCTTTTCGAGGAAAATCCGATCAATGCTAGCGGTGTG<br>GACGCGAAAGCTATCCTGAGTGCCCGGTTATCTAAATCACGGCGGCTGGAGAACTTGATTGCCCAGTTGCCCGGAGAGAAGAAAAAC<br>GGATTATTCGGTAATCTTATCGCCCTTTCTCTGGGGTTGACGCCCAACTTCAAATCTAACTTTGATCTGGCCGAAGACGCTAAATTACA<br>ACTTAGTAAAGATACCTACGACGATGACCTGGATAATTTGTTGGCTCAAATCGGGGATCAATATGCTGACCTTTTCCTTGCGGCGAAG<br>AACTTATCGGACGCCATCTTGCTGTCGGATATCCTGCGTGTCAATACGGAGATTACTAAGGCACCACTTTCCGCATCCATGATCAAGC<br>GTTATGACGAGCATCACCAAGACCTTACGTTGCTGAAAGCCTTAGTCCGTCAACAGTTACCTGAAAAATATAAAGAAATTTTTTTTGATC<br>AGTCTAAGAATGGATACGCAGGTTATATCGACGGAGGAGCAAGCCAAGAAGAATTTTACAAATTTATTAAACCGATTCTTGAAAAGATG<br>GATGGTACGGAGGAACTTCTGGTTAAGTTAAATCGCGAAGACTTGTTGCGCAAGCAGCGCACTTTCGACAACGGGAGTATCCCCCAC<br>CAAATTCATCTTGGTGAATTACATGCGATCCTGCGGCGGCAAGAAGATTTTTATCCTTTCTTAAAAGATAATCGCGAAAAAATTGAAAAA<br>ATCTTAACGTTTCGCATTCCTTATTACGTGGGCCCTCTGGCACGCGGTAATAGCCGGTTTGCCTGGATGACCCGCAAATCAGAAGAGA<br>CCATCACCCCGTGGAATTTCGAGGAGGTAGTAGACAAGGGTGCCAGTGCACAATCCTTCATCGAACGCATGACAAATTTTGATAAAAA	

	TTTGCCTAATGAGAAGGTCCTGCCGAAACATAGTCTTTTATATGAATACTTTACCGTATACAACGAGTTGACAAAGGTTAAGTACGTGA<br>CGGAGGGGATGCGTAAACCTGCATTTTTGTCAGGTGAGCAAAAAAAAGCGATTGTGGATTTATTATTTAAAACTAATCGGAAGGTGAC<br>AGTGAAGCAACTTAAGGAAGACTATTTCAAGAAGATCGAATGCTTCGATTCGGTAGAAATCAGTGGTGTTGAAGACCGCTTCAACGCA<br>AGCTTAGGGACTTATCACGATCTTCTTAAGATCATTAAGGACAAAGACTTCCTGGACAACGAGGAAAACGAGGACATTTTGGAAGATAT<br>TGTACTTACTTTGACTTTGTTTGAAGACCGGGAGATGATCGAAGAGCGTCTTAAGACGTACGCGCACCTTTTCGATGACAAAGTAATGA<br>AGCAGCTGAAGCGGCGGCGGTACACCGGATGGGGTCGTTTATCTCGGAAATTGATCAATGGCATTCGTGACAAGCAAAGCGGCAAG<br>ACGATCTTAGATTTCCTTAAGTCCGATGGATTTGCTAATCGGAACTTTATGCAATTGATTCATGACGATAGTCTTACCTTTAAAGAGGAC<br>ATCCAAAAGGCACAAGTGTCAGGCCAAGGTGACAGTCTTCACGAACATATTGCTAACTTAGCCGGTAGTCCAGCTATCAAGAAGGGTA<br>TCTTACAGACTGTTAAAGTAGTAGATGAACTGGTGAAAGTAATGGGTCGTCACAAGCCTGAGAATATCGTAATTGAAATGGCGCGGGA<br>AAATCAGACAACTCAAAAAGGTCAAAAAAACAGTCGTGAACGTATGAAACGCATTGAAGAGGGCATCAAGGAGTTGGGCAGTCAGATT<br>TTAAAGGAGCATCCAGTGGAGAACACTCAGCTGCAAAATGAGAAACTTTATCTTTATTACCTTCAGAATGGACGCGATATGTACGTCGA<br>TCAAGAACTGGATATTAATCGGTTATCCGATTACGACGTTGATGCCATCGTCCCTCAATCATTTCTGAAAGACGATAGCATCGACAATA<br>AGGTGCTGACACGTAGTGACAAGAACCGTGGGAAGAGCGACAACGTACCGTCGGAGGAGGTCGTTAAGAAGATGAAGAACTATTGG<br>CGGCAATTGCTGAACGCTAAATTGATTACGCAGCGGAAATTTGACAACCTGACCAAGGCAGAGCGTGGGGGCCTGAGTGAACTGGAT<br>AAAGCTGGCTTTATCAAACGTCAATTGGTAGAGACACGCCAAATTACGAAGCATGTCGCGCAGATTTTGGACTCGCGCATGAACACTA<br>AGTACGATGAAAACGACAAGTTAATTCGTGAGGTGAAAGTCATTACTCTTAAGAGTAAACTTGTCTCGGACTTCCGGAAGGACTTTCAA<br>TTTTACAAAGTCCGTGAAATTAATAACTATCATCACGCCCACGACGCATATCTTAACGCGGTCGTTGGAACAGCTTTGATTAAGAAATA<br>TCCCAAGCTTGAGAGCGAGTTCGTGTACGGTGATTATAAAGTGTATGACGTACGTAAGATGATCGCGAAGTCCGAACAAGAGATTGG<br>GAAGGCAACTGCTAAGTACTTTTTTTACTCTAATATTATGAACTTTTTCAAAACCGAGATCACATTGGCTAATGGCGAAATCCGTAAGCG<br>CCCTCTTATTGAGACAAACGGGGAAACTGGGGAGATCGTGTGGGATAAGGGCCGCGATTTTGCAACTGTCCGGAAGGTCCTGTCTAT<br>GCCCCAAGTGAACATCGTTAAGAAGACGGAAGTTCAGACCGGGGGCTTTTCAAAGGAGAGTATTTTACCCAAGCGCAACAGTGACAA<br>GTTGATCGCGCGGAAGAAGGACTGGGATCCCAAAAAGTATGGGGGATTCGACTCACCGACCGTTGCATACTCCGTGCTGGTCGTCG<br>CGAAAGTGGAGAAGGGCAAGAGTAAAAAACTGAAGTCGGTTAAGGAGTTGCTTGGCATCACTATTATGGAACGTAGTAGCTTCGAGAA<br>GAATCCAATCGATTTTTTAGAAGCCAAGGGCTACAAAGAAGTTAAGAAAGACCTTATTATCAAGCTGCCAAAGTACTCACTTTTCGAATT<br>AGAAAACGGACGTAAGCGCATGTTAGCGTCCGCTGGTGAACTGCAAAAAGGAAATGAGCTGGCACTGCCTTCTAAATACGTGAACTTT<br>TTATATTTGGCGTCCCATTACGAAAAGCTTAAGGGGTCGCCCGAAGATAACGAGCAAAAGCAGCTGTTTGTGGAACAACACAAGCATT<br>ATCTGGATGAGATCATCGAGCAGATTAGCGAATTCTCAAAGCGGGTGATCTTAGCCGATGCTAACTTGGATAAAGTGCTGTCCGCTTA<br>CAACAAACATCGCGATAAGCCCATCCGTGAGCAGGCGGAGAATATCATTCATCTGTTTACATTGACGAATTTGGGGGCCCCCGCTGC<br>ATTCAAGTACTTCGATACTACAATCGACCGTAAACGTTACACTAGTACAAAAGAAGTTTTGGACGCTACCCTGATCCATCAGAGTATCA<br>CTGGCCTGTACGAGACACGCATCGACTTGTCCCAATTGGGGGGGGATTAACTCGAGTAAGGATCTCCAGGCATCAAATAAAACGAAA<br>GGCTCAGTCGAAAGACTGGGCCTTTCGTTTTATCTGTTGTTTGTCGGTGAACGCTCTCTACTAGAGTCACACTGGCTCACCTTCGGGT<br>GGGCCTTTCTGCGTTTATAAAACCCTGCAGGGGCCGGCCCCGTAGAAAAGATCAAAGGATCTTCTTGAGATCCTTTTTTTCTGCGCGT<br>AATCTGCTGCTTGCAAACAAAAAAACCACCGCTACCAGCGGTGGTTTGTTTGCCGGATCAAGAGCTACCAACTCTTTTTCCGAAGGTA<br>ACTGGCTTCAGCAGAGCGCAGATACCAAATACTGTTCTTCTAGTGTAG	
pOSIP-KO	GAATTCGAGCTCGGTACCCGGGGATCCTCTAGAGGGCTTACTAAAAGCCAGATAACAGTATGCGTATTTGCGCGCTGATTTTTGCGGT<br>ATAAGAATATATACTGATATGTATACCCGAAGTATGTCAAAAAGAGGTATGCTATGCAGTTTAAGGTTTACACCTATAAAAGAGAGAGC<br>CGTTATCGTCTGTTTGTGGATGTACAGAGTGATATTATTGACACGCCCGGGCGACGGATGGTGATCCCCCTGGCCAGTGCACGTCTG<br>CTGTCAGATAAAGTCTCCCGTGAACTTTACCCGGTGGTGCATATCGGGGATGAAAGCTGGCGCATGATGACCACCGATATGGCCAGT<br>GTGCCGGTCTCCGTTATCGGGGAAGAAGTGGCTGATCTCAGCCACCGCGAAAATGACATCAAAAACGCCATTAACCTGATGTTCTGG<br>GGAATATAATAACCCGTAGAAAAGATCAAAAGATCTTCTTGAGATCCTTTTTTTCTGCGCGTAATCTGCTACTTGCAAACAAAAAAACCA<br>CCGCTACCAGCGGTGGTTTGTTTGCCGGATCAAGAGCTACCAACTCTTTTTCCGAAGGTAACTGGCTTTAGCAGAGCGCAGATACCAA<br>ATACTGTCCTTCTAGTGTAGCCGTAGTTAGGCCACCACTTCAAGAACTCTGTAGCACCGCCTACATACCTCGCTCTGCTAATCCTGTTA<br>CCAGTGGCTGCTGCCAGTGGCGATAAGTCGTGTCTTACCGGGTTGGACTCAAGACGATAGTTACCGGATAAGGCGCAGCGGTCGGG<br>CTGAACGGGGGGTTCGTGCACACAGCCCAGCTTGGAGCGAACGACCTACACCGAACTGAGATACCTACAGCGTGAGCTATGAGAAA<br>GCGCCACGCTTCCCGAAGGGAGAAAGGCGGACAGGTATCCGGTAAGCGGCAGGGTCGGAACAGGAGAGCGCACGAGGGAGCTTCC<br>AGGGGGAAACGCCTGGTATCTTTATAGTCCTGTCGGGTTTCGCCACCTCTGACTTGAGCGTCGATTTTTGTGATGCTCGTCAGGGGG<br>GCGGAGCCTATGGAAAAACGCCAGCAACGCGGCCTTTTTACGGTTCCTGGCCTTTTGCTGGCCTTTTGCTCACATTACTAGTAGCGG<br>CCGCTGCAGGCATGCCTCGAGATGCATGGCGCCTAACCTAAACTGACAGGCATCAAATTAAGCAGAAGGCCATCCTGACGGATGGCC<br>TTTTTGCGTTTCGAACAATTGAAAAAACCTCGCGCCTTACCTGTTGAGTAATAGTCAAAAGCCTCCGGTCGGAGGCTTTTGACTTTCTG<br>CTTACTGAATTTCGGTGGTGCCGTTAATTAACCGGTGGGCCCTCATGATAATAATGGTTTCTTAGACGTCCGAAGTTCCTATTCTCTAG<br>AAAGTATAGGAACTTCCCTAGGTCAGCCAAACGTCTCTTCAGGCCACTGACTAGCGATAACTTTCCCCACAACGGAACAACTCTCATT<br>GCATGGGATCATTGGGTACTGTGGGTTTAGTGGTTGTAAAAACACCTGACCGCTATCCCTGATCAGTTTCTTGAAGGTAAACTCATCA<br>CCCCCAAGTCTGGCTATGCAGAAATCACCTGGCTCAACAGCCTGCTCAGGGTCAACGAGAATTAACATTCCGTCAGGAAAGCTTGGC<br>TTGGAGCCTGTTGGTGCGGTCATGGAATTACCTTCAACCTCAAGCCAGAATGCAGAATCACTGGCTTTTTTGGTTGTGCTTACCCATCT<br>CTCCGCATCACCTTTGGTAAAGGTTCTAAGCTTAGGTGAGAACATCCCTGCCTGAACATGAGAAAAAACAGGGTACTCATACTCACTT<br>CTAAGTGACGGCTGCATACTAACCGCTTCATACATCTCGTAGATTTCTCTGGCGATTGAAGGGCTAAATTCTTCAACGCTAACTTTGAG<br>AATTTTTGTAAGCAATGCGGCGTTATAAGCATTTAATGCATTGATGCCATTAAATAAAGCACCAACGCCTGACTGCCCCATCCCCATCT	St-Pierre *et al.*, 2013[11]

	TGTCTGCGACAGATTCCTGGGATAAGCCAAGTTCATTTTTCTTTTTTTCATAAATTGCTTTAAGGCGACGTGCGTCCTCAAGCTGCTCTT<br>GTGTTAATGGTTTCTTTTTTGTGCTCATACGTTAAATCTATCACCGCAAGGGATAAATATCTAACACCGTGCGTGTTGACTATTTTACCT<br>CTGGCGGTGATAATGGTTGCATGTACTAAGGAGGTTGTATACGCGTTTTGTTTAACTTTAAGAAGGAGATATACATATGACCGTCCGTA<br>AAAATCCGGCTGGCGGTTGGATTTGTGAACTCTACCCAAACGGTGCAAAAGGCAAACGTATCAGAAAGAAATTCGCTACTAAAGGCGA<br>GGCTCTGGCGTTTGAGCAGTACACCGTTCAAAACCCGTGGCAGGAAGAAAAGGAAGACAGGCGCACGTTAAAAGAGCTGGTTGATTC<br>ATGGTATAGCGCTCATGGCATTACACTGAAAGATGGTTTGAAACGCCAGTTAGCCATGCACCATGCTTTTGAGTGTATGGGCGAACCA<br>CTCGCACGCGATTTCGATGCGCAGATGTTTTCCCGCTACCGAGAAAAACGGTTAAAAGGTGAGTATGCCCGTTCAAACAGAGTGAAA<br>GAGGTATCGCCTCGCACGCTTAATCTTGAGCTGGCCTACTTCCGGGCGGTGTTCAATGAGCTAAACCGCCTCGGAGAATGGAAGGGT<br>GAAAACCCACTAAAAAATATGCGCCCATTCCGCACAGAAGAAATGGAAATGACTTGGCTAACTCACGACCAAATTTCGCAACTGCTCG<br>GAGAGTGTAACCGACATGACCACCCTGATTTAGAAACCGTGGTAAGAATCTGTCTCGCAACTGGCGCACGGTGGTCTGAGGCCGAGA<br>GTCTGAGAAAAAGCCAACTCGCGAAATACAAAATCACATACACCAACACGAAAGGTAGAAAAAATCGCACCGTCCCAATCAGCAAAGA<br>GCTCTATGAGTCTCTGCCTGATGATAAAAAGGGCCGGTTGTTCAGTGATTGTTATGGCGCGTTCCGGTCAGCTTTGGAAAGAACAGGT<br>ATCGAACTACCGGCAGGACAACTTACCCACGTTTTACGTCACACCTTCGCTAGTCACTTTATGATGAATGGCGGTAATATTTTGGTGTT<br>GCAGCGCGTACTCGGCCATACAGATATAAAAATGACCATGCGATATGCGCACTTTGCACCCGACCATTTAGAGGATGCAGTTAAACTA<br>AATCCTTTAGTTCACATTACTAACAGCAAATAAGTTATGGAGGTTGTATGTCAGTTCGGCTAGCCCATGGGTATGGACAGTTTTCCCTT<br>TGATATGTAACGCACGTTGTGTCTCAAAATCTCTGATGTTACATTGCACAAGATAAAAATATATCATCATGAACAATAAAACTGTCTGCT<br>TACATAAACAGTAATACAAGGGGTGTTATGAGCCATATTCAACGGGAAACGTCTTGCTCCCGTCCGCGCTTAAACTCCAACATGGACG<br>CTGATTTATATGGGTATAAATGGGCTCGCGATAATGTCGGGCAATCAGGTGCGACAATCTATCGCTTGTATGGGAAGCCCGATGCGC<br>CAGAGTTGTTTCTGAAACATGGCAAAGGTAGCGTTGCCAATGATGTTACAGATGAGATGGTCCGTCTCAACTGGCTGACGGAGTTTAT<br>GCCTCTCCCGACCATCAAGCATTTTATCCGTACTCCTGATGATGCGTGGTTACTCACCACCGCGATTCCTGGGAAAACAGCCTTCCAG<br>GTATTAGAAGAATATCCTGATTCAGGTGAAAATATTGTTGATGCGCTGGCCGTGTTCCTGCGCCGGTTACATTCGATTCCTGTTTGTAA<br>TTGTCCTTTTAACAGCGATCGTGTATTTCGTCTTGCTCAGGCGCAATCACGCATGAATAACGGTTTGGTTGATGCGAGTGATTTTGATG<br>ACGAGCGTAATGGCTGGCCTGTTGAACAAGTCTGGAAAGAAATGCACAAGCTCTTGCCATTCTCACCGGATTCAGTCGTCACTCATGG<br>TGATTTCTCACTTGATAACCTTATTTTTGACGAGGGGAAATTAATAGGTTGTATTGATGTTGGACGGGTCGGAATCGCAGACCGTTACC<br>AGGACCTTGCCATTCTTTGGAACTGCCTCGGTGAGTTTTCTCCTTCATTACAGAAACGGCTTTTTCAAAAATATGGTATTGATAATCCTG<br>ATATGAATAAATTGCAGTTTCATTTGATGCTCGATGAGTTTTTCTAATAATACTAGCAGAAATCATCCTTAGCGAAAGCTAAGGATTTTTT<br>TTATCTGATTACCGCCTTTGAGTGAGCGTCGACCTAGTGCGGCCGCAAGATCCGGCCACGATGCGTCCGGCGTAGAGGATCTGAAGA<br>TCAGCAGTTCAACCTGTTGATAGTACGTACTAAGCTCTCATGTTTCACGTACTAAGCTCTCATGTTTAACGTACTAAGCTCTCATGTTTA<br>ACGAACTAAACCCTCATGGCTAACGTACTAAGCTCTCATGGCTAACGTACTAAGCTCTCATGTTTCACGTACTAAGCTCTCATGTTTGA<br>ACAATAAAATTAATATAAATCAGCAACTTAAATAGCCTCTAAGGTTTTAAGTTTTATAAGAAAAAAAAGAATATATAAGGCTTTTAAAGCT<br>TTTAAGGTTTAACGGTTGTGGACAACAAGCCAGGGATGTAACGCACTGAGAAGCCCTTAGAGCCTCTCAAAGCAATTTTGAGTGACAC<br>AGGAACACTTAACGGCTGACATGGGAATTAGGAAGTTCCTATTCCGAAGTTCCTATTCTCTAGAAAGTATAGGAACTTCCATATGCCAT<br>GGGCGATGGTTCTGAGTAACAGATAATAGAATGGCGATAAAGTGGCGGTAGAAATGGCGAATAATAGGTAATTATTGGCAAACAATGG<br>CAATCTATGTCAATGATAAATAACGCATACTATTGATTTTCGGTTGTTCTTGTAGGAACTCATAATCGCTTGGTCGCTGGTTCAAGTCCA<br>GCAGGGGCCACCAAATTTAAGTAGTAAATACATACAGTTAGGCCACTCTCTCGAGTGGCCTTTTTGTTGCCTCGTTTTCGAGTGGCGA<br>TAGAATGGCGGTGGATTTTTTACCGCCACTTTCCTGAGGAGTGCATAGCTAGCTTCTTCGTCTGTTTCTACTGGTATTGGCACAAACCT<br>GATTCCAATTTGAGCAAGGCTATGTGCCATCTCGATACTCGTTCTTAACTCAACAGAAGATGCTTTGTGCATACAGCCCCTCGTTTATT<br>ATTTATCTCCTCAGCCAGCCGCTGTGCTTTCAGTGGATTTCGGATAACAGAAAGGCCGGGAAATACCCAGCCTCGCTTTGTAACGGAG<br>TAGAGACGAAAGTGATTGCGCCTACCCGGATATTATCGTGAGGATGCGTCATCGCCATTAATTCACTGATCAGTGATAGCTGTCAAAC<br>ATGAGAATTGATCCGGCTGCCTCGCGCGTTTCGGTGATGACGGTGAAAACCTCTGACACATGCAGCTCCCGGAGACGGTCACAGCTT<br>GTCTGTAAGCGGATGCCGGGAGCAGACAAGCCCGTCAGGGCGCGTCAGCGGGTGTTGGCGGGTGTCGGGGCGCAGCCATGACCC<br>AGTCACGTAGCGATAGCGGAGTGTATGCTGCACATGACATTAACCTATAAAAATAGGCGTATCACGAGGCCCTTTCGTCTTCAAGAAT<br>TAATTCCCAATTCCCCAGGCATCAAATAAAACGAAAGGCTCAGTCGAAAGACTGGGCCTTTCGTTTTATCTGTTGTTTGTCGGTGAACG<br>CTCTCCTGAGTAGGACAAATCCGCCGGGAGCGGATTTGAACGTTGCGAAGCAACGGCCCGGAGGGTGGCGGGCAGGACGCCCGCC<br>ATAAACTGCCAGGAATTAATTCCCCAGGCATCAAATAAAACGAAAGGCTCAGTCGAAAGACTGGGCCTTTCGTTTTATCTGTTGTTTGT<br>CGGTGAACGCTCTCCTGAGTAGGACAAATCCGCCGGGAGCGGATTTGAACGTTGCGAAGCAACGGCCCGGAGGGTGGCGGGCAGG<br>ACGCCCGCCATAAACTGCCAGGAATTAATTCCCCAGGCATCAAATAAAACGAAAGGCTCAGTCGAAAGACTGGGCCTTTCGTTTTATC<br>TGTTGTTTGTCGGTGAACGCTCTCCTGAGTAGGACAAATCCGCCGGGAGCGGATTTGAACGTTGCGAAGCAACGGCCCGGAGGGTG<br>GCGGGCAGGACGCCCGCCATAAACTGCCAGGAATTGGGGATCG	
pOSIP-<br>KO_<br>PBAD-<br>dCas9	GAATTCGAGCTCGGTACCCGGGGATCCACTTTTCATACTCCCGCCATTCAGAGAAGAAACCAATTGTCCATATTGCATCAGACATTGC<br>CGTCACTGCGTCTTTTACTGGCTCTTCTCGCTAACCAAACCGGTAACCCCGCTTATTAAAAGCATTCTGTAACAAAGCGGGACCAAAG<br>CCATGACAAAAACGCGTAACAAAAGTGTCTATAATCACGGCAGAAAAGTCCACATTGATTATTTGCACGGCGTCACACTTTGCTATGCC<br>ATAGCATTTTTATCCATAAGATTAGCGGATTCTACCTGACGCTTTTTATCGCAACTCTCTACTGTTTCTCCATACCCGTTTTTTTGGGAAT<br>TCAAAAGATCTAAAGAGGAGAAAGGATCTATGGATAAGAAGTACTCCATTGGTTTAGCAATTGGCACGAACAGTGTCGGATGGGCCGT<br>GATTACAGATGAATACAAGGTCCCTTCAAAGAAATTCAAAGTGTTAGGTAATACTGACCGGCACAGTATTAAGAAAAACCTGATCGGG<br>GCACTTCTGTTCGATTCCGGGGAAACTGCAGAGGCAACACGTTTGAAGCGTACCGCCCGGCGCCGTTATACGCGTCGTAAAAATCGC<br>ATCTGTTACTTACAAGAAATCTTCTCAAATGAGATGGCGAAGGTAGACGATTCTTTTTTTCATCGGCTGGAGGAATCGTTTTTGGTAGA	This<br>study

AGAGGACAAGAAGCATGAACGCCATCCGATTTTCGGGAATATTGTCGATGAAGTTGCCTATCATGAGAAATATCCGACAATTTACCATT
TACGGAAAAAACTTGTGGATTCTACGGACAAAGCTGACTTGCGGTTGATCTATCTTGCCCTGGCCCACATGATTAAGTTCCGTGGGCA
CTTTCTTATCGAAGGGGATTTGAACCCAGATAACTCGGACGTTGACAAGTTGTTTATTCAATTGGTCCAAACCTATAACCAGCTTTTCG
AGGAAAATCCGATCAATGCTAGCGGTGTGGACGCGAAAGCTATCCTGAGTGCCCGGTTATCTAAATCACGGCGGCTGGAGAACTTGA
TTGCCCAGTTGCCCGGAGAGAAGAAAAACGGATTATTCGGTAATCTTATCGCCCTTTCTCTGGGGTTGACGCCCAACTTCAAATCTAA
CTTTGATCTGGCCGAAGACGCTAAATTACAACTTAGTAAAGATACCTACGACGATGACCTGGATAATTTGTTGGCTCAAATCGGGGATC
AATATGCTGACCTTTTCCTTGCGGCGAAGAACTTATCGGACGCCATCTTGCTGTCGGATATCCTGCGTGTCAATACGGAGATTACTAA
GGCACCACTTTCCGCATCCATGATCAAGCGTTATGACGAGCATCACCAAGACCTTACGTTGCTGAAAGCCTTAGTCCGTCAACAGTTA
CCTGAAAAATATAAAGAAATTTTTTTTGATCAGTCTAAGAATGGATACGCAGGTTATATCGACGGAGGAGCAAGCCAAGAAGAATTTTA
CAAATTTATTAAACCGATTCTTGAAAAGATGGATGGTACGGAGGAACTTCTGGTTAAGTTAAATCGCGAAGACTTGTTGCGCAAGCAGC
GCACTTTCGACAACGGGAGTATCCCCCACCAAATTCATCTTGGTGAATTACATGCGATCCTGCGGCGGCAAGAAGATTTTTATCCTTT
CTTAAAAGATAATCGCGAAAAAATTGAAAAAATCTTAACGTTTCGCATTCCTTATTACGTGGGCCCTCTGGCACGCGGTAATAGCCGGT
TTGCCTGGATGACCCGCAAATCAGAAGAGACCATCACCCCGTGGAATTTCGAGGAGGTAGTAGACAAGGGTGCCAGTGCACAATCCT
TCATCGAACGCATGACAAATTTTGATAAAAATTTGCCTAATGAGAAGGTCCTGCCGAAACATAGTCTTTTATATGAATACTTTACCGTAT
ACAACGAGTTGACAAAGGTTAAGTACGTGACGGAGGGGATGCGTAAACCTGCATTTTTGTCAGGTGAGCAAAAAAAAGCGATTGTGG
ATTTATTATTTAAAACTAATCGGAAGGTGACAGTGAAGCAACTTAAGGAAGACTATTTCAAGAAGATCGAATGCTTCGATTCGGTAGAA
ATCAGTGGTGTTGAAGACCGCTTCAACGCAAGCTTAGGGACTTATCACGATCTTCTTAAGATCATTAAGGACAAAGACTTCCTGGACAA
CGAGGAAAACGAGGACATTTTGGAAGATATTGTACTTACTTTGACTTTGTTTGAAGACCGGGAGATGATCGAAGAGCGTCTTAAGACG
TACGCGCACCTTTTCGATGACAAAGTAATGAAGCAGCTGAAGCGGCGGCGGTACACCGGATGGGGTCGTTTATCTCGGAAATTGATC
AATGGCATTCGTGACAAGCAAAGCGGCAAGACGATCTTAGATTTCCTTAAGTCCGATGGATTTGCTAATCGGAACTTTATGCAATTGAT
TCATGACGATAGTCTTACCTTTAAAGAGGACATCCAAAAGGCACAAGTGTCAGGCCAAGGTGACAGTCTTCACGAACATATTGCTAAC
TTAGCCGGTAGTCCAGCTATCAAGAAGGGTATCTTACAGACTGTTAAAGTAGTAGATGAACTGGTGAAAGTAATGGGTCGTCACAAGC
CTGAGAATATCGTAATTGAAATGGCGCGGGAAAATCAGACAACTCAAAAAGGTCAAAAAAACAGTCGTGAACGTATGAAACGCATTGA
AGAGGGCATCAAGGAGTTGGGCAGTCAGATTTTAAAGGAGCATCCAGTGGAGAACACTCAGCTGCAAAATGAGAAACTTTATCTTTAT
TACCTTCAGAATGGACGCGATATGTACGTCGATCAAGAACTGGATATTAATCGGTTATCCGATTACGACGTTGATGCCATCGTCCCTCA
ATCATTTCTGAAAGACGATAGCATCGACAATAAGGTGCTGACACGTAGTGACAAGAACCGTGGGAAGAGCGACAACGTACCGTCGGA
GGAGGTCGTTAAGAAGATGAAGAACTATTGGCGGCAATTGCTGAACGCTAAATTGATTACGCAGCGGAAATTTGACAACCTGACCAAG
GCAGAGCGTGGGGGCCTGAGTGAACTGGATAAAGCTGGCTTTATCAAACGTCAATTGGTAGAGACACGCCAAATTACGAAGCATGTC
GCGCAGATTTTGGACTCGCGCATGAACACTAAGTACGATGAAAACGACAAGTTAATTCGTGAGGTGAAAGTCATTACTCTTAAGAGTA
AACTTGTCTCGGACTTCCGGAAGGACTTTCAATTTTACAAAGTCCGTGAAATTAATAACTATCATCACGCCCACGACGCATATCTTAAC
GCGGTCGTTGGAACAGCTTTGATTAAGAAATATCCCAAGCTTGAGAGCGAGTTCGTGTACGGTGATTATAAAGTGTATGACGTACGTA
AGATGATCGCGAAGTCCGAACAAGAGATTGGGAAGGCAACTGCTAAGTACTTTTTTTACTCTAATATTATGAACTTTTTCAAAACCGAG
ATCACATTGGCTAATGGCGAAATCCGTAAGCGCCCTCTTATTGAGACAAACGGGGAAACTGGGGAGATCGTGTGGGATAAGGGCCGC
GATTTTGCAACTGTCCGGAAGGTCCTGTCTATGCCCCAAGTGAACATCGTTAAGAAGACGGAAGTTCAGACCGGGGGCTTTTCAAAG
GAGAGTATTTTACCCAAGCGCAACAGTGACAAGTTGATCGCGCGGAAGAAGGACTGGGATCCCAAAAAGTATGGGGGATTCGACTCA
CCGACCGTTGCATACTCCGTGCTGGTCGTCGCGAAAGTGGAGAAGGGCAAGAGTAAAAAACTGAAGTCGGTTAAGGAGTTGCTTGGC
ATCACTATTATGGAACGTAGTAGCTTCGAGAAGAATCCAATCGATTTTTTAGAAGCCAAGGGCTACAAAGAAGTTAAGAAAGACCTTAT
TATCAAGCTGCCAAAGTACTCACTTTTCGAATTAGAAAACGGACGTAAGCGCATGTTAGCGTCCGCTGGTGAACTGCAAAAAGGAAAT
GAGCTGGCACTGCCTTCTAAATACGTGAACTTTTTATATTTGGCGTCCCATTACGAAAAGCTTAAGGGGTCGCCCGAAGATAACGAGC
AAAAGCAGCTGTTTGTGGAACAACACAAGCATTATCTGGATGAGATCATCGAGCAGATTAGCGAATTCTCAAAGCGGGTGATCTTAGC
CGATGCTAACTTGGATAAAGTGCTGTCCGCTTACAACAAACATCGCGATAAGCCCATCCGTGAGCAGGCGGAGAATATCATTCATCTG
TTTACATTGACGAATTTGGGGGCCCCCGCTGCATTCAAGTACTTCGATACTACAATCGACCGTAAACGTTACACTAGTACAAAAGAAGT
TTTGGACGCTACCCTGATCCATCAGAGTATCACTGGCCTGTACGAGACACGCATCGACTTGTCCCAATTGGGGGGGGATTAACTCGA
GTAAGGATCTCCAGGCATCAAATAAAACGAAAGGCTCAGTCGAAAGACTGGGCCTTTCGTTTTATCTGTTGTTTGTCGGTGAACGCTC
TCTACTAGAGTCACACTGGCTCACCTTCGGGTGGGCCTTTCTGCGTTTATAAAACCCTGCAGGGGCCGGCCCCGTAGAAAACTAGTA
GCGGCCGCTGCAGGCATGCCTCGAGATGCATGGCGCCTAACCTAAACTGACAGGCATCAAATTAAGCAGAAGGCCATCCTGACGGA
TGGCCTTTTTGCGTTTCGAACAATTGAAAAAACCTCGCGCCTTACCTGTTGAGTAATAGTCAAAAGCCTCCGGTCGGAGGCTTTTGACT
TTCTGCTTACTGAATTTCGGTGGTGCCGTTAATTAACCGGTGGGCCCTCATGATAATAATGGTTTCTTAGACGTCCGAAGTTCCTATTC
TCTAGAAAGTATAGGAACTTCCCTAGGTCAGCCAAACGTCTCTTCAGGCCACTGACTAGCGATAACTTTCCCCACAACGGAACAACTC
TCATTGCATGGGATCATTGGGTACTGTGGGTTTAGTGGTTGTAAAAACACCTGACCGCTATCCCTGATCAGTTTCTTGAAGGTAAACTC
ATCACCCCCAAGTCTGGCTATGCAGAAATCACCTGGCTCAACAGCCTGCTCAGGGTCAACGAGAATTAACATTCCGTCAGGAAAGCTT
GGCTTGGAGCCTGTTGGTGCGGTCATGGAATTACCTTCAACCTCAAGCCAGAATGCAGAATCACTGGCTTTTTTGGTTGTGCTTACCC
ATCTCTCCGCATCACCTTTGGTAAAGGTTCTAAGCTTAGGTGAGAACATCCCTGCCTGAACATGAGAAAAAACAGGGTACTCATACTCA
CTTCTAAGTGACGGCTGCATACTAACCGCTTCATACATCTCGTAGATTTCTCTGGCGATTGAAGGGCTAAATTCTTCAACGCTAACTTT
GAGAATTTTTGTAAGCAATGCGGCGTTATAAGCATTTAATGCATTGATGCCATTAAATAAAGCACCAACGCCTGACTGCCCCATCCCCA
TCTTGTCTGCGACAGATTCCTGGGATAAGCCAAGTTCATTTTTCTTTTTTTCATAAATTGCTTTAAGGCGACGTGCGTCCTCAAGCTGCT
CTTGTGTTAATGGTTTCTTTTTTGTGCTCATACGTTAAATCTATCACCGCAAGGGATAAATATCTAACACCGTGCGTGTTGACTATTTTA

	CCTCTGGCGGTGATAATGGTTGCATGTACTAAGGAGGTTGTATACGCGTTTTGTTTAACTTTAAGAAGGAGATATACATATGACCGTCC<br>GTAAAAATCCGGCTGGCGGTTGGATTTGTGAACTCTACCCAAACGGTGCAAAAGGCAAACGTATCAGAAAGAAATTCGCTACTAAAGG<br>CGAGGCTCTGGCGTTTGAGCAGTACACCGTTCAAAACCCGTGGCAGGAAGAAAAGGAAGACAGGCGCACGTTAAAAGAGCTGGTTG<br>ATTCATGGTATAGCGCTCATGGCATTACACTGAAAGATGGTTTGAAACGCCAGTTAGCCATGCACCATGCTTTTGAGTGTATGGGCGA<br>ACCACTCGCACGCGATTTCGATGCGCAGATGTTTTCCCGCTACCGAGAAAAACGGTTAAAAGGTGAGTATGCCCGTTCAAACAGAGT<br>GAAAGAGGTATCGCCTCGCACGCTTAATCTTGAGCTGGCCTACTTCCGGGCGGTGTTCAATGAGCTAAACCGCCTCGGAGAATGGAA<br>GGGTGAAAACCCACTAAAAAATATGCGCCCATTCCGCACAGAAGAAATGGAAATGACTTGGCTAACTCACGACCAAATTTCGCAACTG<br>CTCGGAGAGTGTAACCGACATGACCACCCTGATTTAGAAACCGTGGTAAGAATCTGTCTCGCAACTGGCGCACGGTGGTCTGAGGCC<br>GAGAGTCTGAGAAAAAGCCAACTCGCGAAATACAAAATCACATACACCAACACGAAAGGTAGAAAAAATCGCACCGTCCCAATCAGCA<br>AAGAGCTCTATGAGTCTCTGCCTGATGATAAAAAGGGCCGGTTGTTCAGTGATTGTTATGGCGCGTTCCGGTCAGCTTTGGAAAGAAC<br>AGGTATCGAACTACCGGCAGGACAACTTACCCACGTTTTACGTCACACCTTCGCTAGTCACTTTATGATGAATGGCGGTAATATTTTGG<br>TGTTGCAGCGCGTACTCGGCCATACAGATATAAAAATGACCATGCGATATGCGCACTTTGCACCCGACCATTTAGAGGATGCAGTTAA<br>ACTAAATCCTTTAGTTCACATTACTAACAGCAAATAAGTTATGGAGGTTGTATGTCAGTTCGGCTAGCCCATGGGTATGGACAGTTTTC<br>CCTTTGATATGTAACGCACGTTGTGTCTCAAAATCTCTGATGTTACATTGCACAAGATAAAAATATATCATCATGAACAATAAAACTGTC<br>TGCTTACATAAACAGTAATACAAGGGGTGTTATGAGCCATATTCAACGGGAAACGTCTTGCTCCCGTCCGCGCTTAAACTCCAACATG<br>GACGCTGATTTATATGGGTATAAATGGGCTCGCGATAATGTCGGGCAATCAGGTGCGACAATCTATCGCTTGTATGGGAAGCCCGAT<br>GCGCCAGAGTTGTTTCTGAAACATGGCAAAGGTAGCGTTGCCAATGATGTTACAGATGAGATGGTCCGTCTCAACTGGCTGACGGAG<br>TTTATGCCTCTCCCGACCATCAAGCATTTTATCCGTACTCCTGATGATGCGTGGTTACTCACCACCGCGATTCCTGGGAAAACAGCCTT<br>CCAGGTATTAGAAGAATATCCTGATTCAGGTGAAAATATTGTTGATGCGCTGGCCGTGTTCCTGCGCCGGTTACATTCGATTCCTGTTT<br>GTAATTGTCCTTTTAACAGCGATCGTGTATTTCGTCTTGCTCAGGCGCAATCACGCATGAATAACGGTTTGGTTGATGCGAGTGATTTT<br>GATGACGAGCGTAATGGCTGGCCTGTTGAACAAGTCTGGAAAGAAATGCACAAGCTCTTGCCATTCTCACCGGATTCAGTCGTCACTC<br>ATGGTGATTTCTCACTTGATAACCTTATTTTTGACGAGGGGAAATTAATAGGTTGTATTGATGTTGGACGGGTCGGAATCGCAGACCGT<br>TACCAGGACCTTGCCATTCTTTGGAACTGCCTCGGTGAGTTTTCTCCTTCATTACAGAAACGGCTTTTTCAAAAATATGGTATTGATAAT<br>CCTGATATGAATAAATTGCAGTTTCATTTGATGCTCGATGAGTTTTTCTAATAATACTAGCAGAAATCATCCTTAGCGAAAGCTAAGGAT<br>TTTTTTTATCTGATTACCGCCTTTGAGTGAGCGTCGACCTAGTGCGGCCGCAAGATCCGGCCACGATGCGTCCGGCGTAGAGGATCT<br>GAAGATCAGCAGTTCAACCTGTTGATAGTACGTACTAAGCTCTCATGTTTCACGTACTAAGCTCTCATGTTTAACGTACTAAGCTCTCAT<br>GTTTAACGAACTAAACCCTCATGGCTAACGTACTAAGCTCTCATGGCTAACGTACTAAGCTCTCATGTTTCACGTACTAAGCTCTCATG<br>TTTGAACAATAAAATTAATATAAATCAGCAACTTAAATAGCCTCTAAGGTTTTAAGTTTTATAAGAAAAAAAAGAATATATAAGGCTTTTAA<br>AGCTTTTAAGGTTTAACGGTTGTGGACAACAAGCCAGGGATGTAACGCACTGAGAAGCCCTTAGAGCCTCTCAAAGCAATTTTGAGTG<br>ACACAGGAACACTTAACGGCTGACATGGGAATTAGGAAGTTCCTATTCCGAAGTTCCTATTCTCTAGAAAGTATAGGAACTTCCATATG<br>CCATGGGCGATGGTTCTGAGTAACAGATAATAGAATGGCGATAAAGTGGCGGTAGAAATGGCGAATAATAGGTAATTATTGGCAAACA<br>ATGGCAATCTATGTCAATGATAAATAACGCATACTATTGATTTTCGGTTGTTCTTGTAGGAACTCATAATCGCTTGGTCGCTGGTTCAAG<br>TCCAGCAGGGGCCACCAAATTTAAGTAGTAAATACATACAGTTAGGCCACTCTCTCGAGTGGCCTTTTTGTTGCCTCGTTTTCGAGTG<br>GCGATAGAATGGCGGTGGATTTTTTACCGCCACTTTCCTGAGGAGTGCATAGCTAGCTTCTTCGTCTGTTTCTACTGGTATTGGCACA<br>AACCTGATTCCAATTTGAGCAAGGCTATGTGCCATCTCGATACTCGTTCTTAACTCAACAGAAGATGCTTTGTGCATACAGCCCCTCGT<br>TTATTATTTATCTCCTCAGCCAGCCGCTGTGCTTTCAGTGGATTTCGGATAACAGAAAGGCCGGGAAATACCCAGCCTCGCTTTGTAA<br>CGGAGTAGAGACGAAAGTGATTGCGCCTACCCGGATATTATCGTGAGGATGCGTCATCGCCATTAATTCACTGATCAGTGATAGCTGT<br>CAAACATGAGAATTGATCCGGCTGCCTCGCGCGTTTCGGTGATGACGGTGAAAACCTCTGACACATGCAGCTCCCGGAGACGGTCAC<br>AGCTTGTCTGTAAGCGGATGCCGGGAGCAGACAAGCCCGTCAGGGCGCGTCAGCGGGTGTTGGCGGGTGTCGGGGCGCAGCCATG<br>ACCCAGTCACGTAGCGATAGCGGAGTGTATGCTGCACATGACATTAACCTATAAAAATAGGCGTATCACGAGGCCCTTTCGTCTTCAA<br>GAATTAATTCCCAATTCCCCAGGCATCAAATAAAACGAAAGGCTCAGTCGAAAGACTGGGCCTTTCGTTTTATCTGTTGTTTGTCGGTG<br>AACGCTCTCCTGAGTAGGACAAATCCGCCGGGAGCGGATTTGAACGTTGCGAAGCAACGGCCCGGAGGGTGGCGGGCAGGACGCC<br>CGCCATAAACTGCCAGGAATTAATTCCCCAGGCATCAAATAAAACGAAAGGCTCAGTCGAAAGACTGGGCCTTTCGTTTTATCTGTTGT<br>TTGTCGGTGAACGCTCTCCTGAGTAGGACAAATCCGCCGGGAGCGGATTTGAACGTTGCGAAGCAACGGCCCGGAGGGTGGCGGG<br>CAGGACGCCCGCCATAAACTGCCAGGAATTAATTCCCCAGGCATCAAATAAAACGAAAGGCTCAGTCGAAAGACTGGGCCTTTCGTTT<br>TATCTGTTGTTTGTCGGTGAACGCTCTCCTGAGTAGGACAAATCCGCCGGGAGCGGATTTGAACGTTGCGAAGCAACGGCCCGGAGG<br>GTGGCGGGCAGGACGCCCGCCATAAACTGCCAGGAATTGGGGATCG	
pCK005.<br>6	GACGTCTTAAGTTACGAAATCATCCTGTGGAGCTTAGTAGGTTTAGCAAGATGGCAGCGCCTAAATGTAGAATGATAAAAGGATTAAG<br>AGATTAATTTCCCTAAAAATGATAAAACAAGCGTTTTGAAAGCGCTTGTTTTTTTGGTTTGCAGTCAGAGTAGAATAGAAGTATCAAAAA<br>AAGCACCGACTCGGTGCCACTTTTTCAAGTTGATAACGGACTAGCCTTATTTTAACTTGCTATGCTGTTTTGAATGGTTCCAACAAGATT<br>ATTTTATAACTTTTATAACAAATAATCAAGGAGAAATTCAAAGAAATTTATCAGCCATAAAACAATACTTAATACTATAGAATGATAACAAA<br>ATAAACTACTTTTTAAAAGAATTTTGTGTTATAATCTATTTATTATTAAGTATTGGGTAATATTTTTTGAAGAGATATTTTGAAAAAGAAAA<br>ATTAAAGCATATTAAACTAATTTCGGAGGTCATTAAAACTATTATTGAAATCATCAAACTCATTATGGATTTAATTTAAACTTTTTATTTTA<br>GGAGGCAAAAATGGATAAGAAATACTCAATAGGCTTAGCTATCGGCACAAATAGCGTCGGATGGGCGGTGATCACTGATGAATATAAG<br>GTTCCGTCTAAAAAGTTCAAGGTTCTGGGAAATACAGACCGCCACAGTATCAAAAAAAATCTTATAGGGGCTCTTTTATTTGACAGTGG<br>AGAGACAGCGGAAGCGACTCGTCTCAAACGGACAGCTCGTAGAAGGTATACACGTCGGAAGAATCGTATTTGTTATCTACAGGAGATT<br>TTTTCAAATGAGATGGCGAAAGTAGATGATAGTTTCTTTCATCGACTTGAAGAGTCTTTTTTGGTGGAAGAAGACAAGAAGCATGAACG	Addgene<br>,<br>#153025

	TCATCCTATTTTTGGAAATATAGTAGATGAAGTTGCTTATCATGAGAAATATCCAACTATCTATCATCTGCGAAAAAAATTGGTAGATTCT<br>ACTGATAAAGCGGATTTGCGCTTAATCTATTTGGCCTTAGCGCATATGATTAAGTTTCGTGGTCATTTTTTGATTGAGGGAGATTTAAAT<br>CCTGATAATAGTGATGTGGACAAACTATTTATCCAGTTGGTACAAACCTACAATCAATTATTTGAAGAAAACCCTATTAACGCAAGTGGA<br>GTAGATGCTAAAGCGATTCTTTCTGCACGATTGAGTAAATCAAGACGATTAGAAAATCTCATTGCTCAGCTCCCCGGTGAGAAGAAAAA<br>TGGCTTATTTGGGAATCTCATTGCTTTGTCATTGGGTTTGACCCCTAATTTTAAATCAAATTTTGATTTGGCAGAAGATGCTAAATTACA<br>GCTTTCAAAAGATACTTACGATGATGATTTAGATAATTTATTGGCGCAAATTGGAGATCAATATGCTGATTTGTTTTTGGCAGCTAAGAA<br>TTTATCAGATGCTATTTTACTTTCAGATATCCTAAGAGTAAATACTGAAATAACTAAGGCTCCCCTATCAGCTTCAATGATTAAACGCTA<br>CGATGAACATCATCAAGACTTGACTCTTTTAAAAGCTTTAGTTCGACAACAACTTCCAGAAAAGTATAAAGAAATCTTTTTTGATCAATC<br>AAAAAACGGATATGCAGGTTATATTGATGGGGGAGCTAGCCAAGAAGAATTTTATAAATTTATCAAACCAATTTTAGAAAAAATGGATG<br>GTACTGAGGAATTATTGGTGAAACTAAATCGTGAAGATTTGCTGCGCAAGCAACGGACCTTTGACAACGGCTCTATTCCCCATCAAATT<br>CACTTGGGTGAGCTGCATGCTATTTTGAGAAGACAAGAAGACTTTTATCCATTTTTAAAAGACAATCGTGAGAAGATTGAAAAAATCTT<br>GACTTTTCGAATTCCTTATTATGTTGGTCCATTGGCGCGTGGCAATAGTCGTTTTGCATGGATGACTCGGAAGTCTGAAGAAACAATTA<br>CCCCATGGAATTTTGAAGAAGTTGTCGATAAAGGTGCTTCAGCTCAATCATTTATTGAACGCATGACAAACTTTGATAAAAATCTTCCAA<br>ATGAAAAAGTACTACCAAAACATAGTTTGCTTTATGAGTATTTTACGGTTTATAACGAATTGACAAAGGTCAAATATGTTACTGAAGGAA<br>TGCGAAAACCAGCATTTCTTTCAGGTGAACAGAAGAAAGCCATTGTTGATTTACTCTTCAAAACAAATCGAAAAGTAACCGTTAAGCAA<br>TTAAAAGAAGATTATTTCAAAAAAATAGAATGTTTTGATAGTGTTGAAATTTCAGGAGTTGAAGATAGATTTAATGCTTCATTAGGTACCT<br>ACCATGATTTGCTAAAAATTATTAAAGATAAAGATTTTTTGGATAATGAAGAAAATGAAGATATCTTAGAGGATATTGTTTTAACATTGAC<br>CTTATTTGAAGATAGGGAGATGATTGAGGAAAGACTTAAAACATATGCTCACCTCTTTGATGATAAGGTGATGAAACAGCTTAAACGTC<br>GCCGTTATACTGGTTGGGGACGTTTGTCTCGAAAATTGATTAATGGTATTAGGGATAAGCAATCTGGCAAAACAATATTAGATTTTTTG<br>AAATCAGATGGTTTTGCCAATCGCAATTTTATGCAGCTGATCCATGATGATAGTTTGACATTTAAAGAAGACATTCAAAAAGCACAAGTG<br>TCTGGACAAGGCGATAGTTTACATGAACATATTGCAAATTTAGCTGGTAGCCCTGCTATTAAAAAAGGTATTTTACAGACTGTAAAAGTT<br>GTTGATGAATTGGTCAAAGTAATGGGGCGGCATAAGCCAGAAAATATCGTTATTGAAATGGCACGTGAAAATCAGACAACTCAAAAGG<br>GCCAGAAAAATTCGCGAGAGCGTATGAAACGAATCGAAGAAGGTATCAAAGAATTAGGAAGTCAGATTCTTAAAGAGCATCCTGTTGA<br>AAATACTCAATTGCAAAATGAAAAGCTCTATCTCTATTATCTCCAAAATGGAAGAGACATGTATGTGGACCAAGAATTAGATATTAATCG<br>TTTAAGTGATTATGATGTCGATGCCATTGTTCCACAAAGTTTCCTTAAAGACGATTCAATAGACAATAAGGTCTTAACGCGTTCTGATAA<br>AAATCGTGGTAAATCGGATAACGTTCCAAGTGAAGAAGTAGTCAAAAAGATGAAAAACTATTGGAGACAACTTCTAAACGCCAAGTTAA<br>TCACTCAACGTAAGTTTGATAATTTAACGAAAGCTGAACGTGGAGGTTTGAGTGAACTTGATAAAGCTGGTTTTATCAAACGCCAATTG<br>GTTGAAACTCGCCAAATCACTAAGCATGTGGCACAAATTTTGGATAGTCGCATGAATACTAAATACGATGAAAATGATAAACTTATTCG<br>AGAGGTTAAAGTGATTACCTTAAAATCTAAATTAGTTTCTGACTTCCGAAAAGATTTCCAATTCTATAAAGTACGTGAGATTAACAATTAC<br>CATCATGCCCATGATGCGTATCTAAATGCCGTCGTTGGAACTGCTTTGATTAAGAAATATCCAAAACTTGAATCGGAGTTTGTCTATGG<br>TGATTATAAAGTTTATGATGTTCGTAAAATGATTGCTAAGTCTGAGCAAGAAATAGGCAAAGCAACCGCAAAATATTTCTTTTACTCTAA<br>TATCATGAACTTCTTCAAAACAGAAATTACACTTGCAAATGGAGAGATTCGCAAACGCCCTCTAATCGAAACTAATGGGGAAACTGGAG<br>AAATTGTCTGGGATAAAGGGCGAGATTTTGCCACAGTGCGCAAAGTATTGTCCATGCCCCAAGTCAATATTGTCAAGAAAACAGAAGT<br>ACAGACAGGCGGATTCTCCAAGGAGTCAATTTTACCAAAAAGAAATTCGGACAAGCTTATTGCTCGTAAAAAAGACTGGGATCCAAAA<br>AAATATGGTGGTTTTGATAGTCCAACGGTAGCTTATTCAGTCCTAGTGGTTGCTAAGGTGGAAAAAGGGAAATCGAAGAAGTTAAAATC<br>CGTTAAAGAGTTACTAGGGATCACAATTATGGAAAGAAGTTCCTTTGAAAAAAATCCGATTGACTTTTTAGAAGCTAAAGGATATAAGG<br>AAGTTAAAAAAGACTTAATCATTAAACTACCTAAATATAGTCTTTTTGAGTTAGAAAACGGTCGTAAACGGATGCTGGCTAGTGCCGGA<br>GAATTACAAAAAGGAAATGAGCTGGCTCTGCCAAGCAAATATGTGAATTTTTTATATTTAGCTAGTCATTATGAAAAGTTGAAGGGTAGT<br>CCAGAAGATAACGAACAAAAACAATTGTTTGTGGAGCAGCATAAGCATTATTTAGATGAGATTATTGAGCAAATCAGTGAATTTTCTAAG<br>CGTGTTATTTTAGCAGATGCCAATTTAGATAAAGTTCTTAGTGCATATAACAAACATAGAGACAAACCAATACGTGAACAAGCAGAAAAT<br>ATTATTCATTTATTTACGTTGACGAATCTTGGAGCTCCCGCTGCTTTTAAATATTTTGATACAACAATTGATCGTAAACGATATACGTCTA<br>CAAAAGAAGTTTTAGATGCCACTCTTATCCATCAATCCATCACTGGTCTTTATGAAACACGCATTGATTTGAGTCAGCTAGGAGGTGAC<br>TAACTCGAGTAAGGATCTCCAGGCATCAAATAAAACGAAAGGCTCAGTCGAAAGACTGGGCCTTTCGTTTTATCTGTTGTTTGTCGGT<br>GAACGCTCTCTACTAGAGTCACACTGGCTCACCTTCGGGTGGGCCTTTCTGCGTTTATACCTAGAGCGCCCGGAAGAGAGTCAATTC<br>AGGGTGGTGAATCTGCAGTTTACGGCTAGCTCAGCCCTAGGTATTATGCTAGCGAATTCATTAAAGAGGAGAAAGGTACCATGGGGC<br>CCGCTTCTAACTTTACTCAGTTCGTTCTCGTCGACAATGGCGGAACTGGCGACGTGACTGTCGCCCCAAGCAACTTCGCTAACGGGA<br>TCGCTGAATGGATCAGCTCTAACTCGCGTTCACAGGCTTACAAAGTAACCTGTAGCGTTCGTCAGAGCTCTGCGCAGAATCGCAAATA<br>CACCATCAAAGTCGAGGTGCCTAAAGGCGCCTGGCGTTCGTACTTAAATATGGAACTAACCATTCCAATTTTCGCCACGAATTCCGAC<br>TGCGAGCTTATTGTTAAGGCAATGCAAGGTCTCCTAAAAGATGGAAACCCGATTCCCTCAGCAATCGCAGCAAACTCCGGCATCTACG<br>GTGGCGGAGGTAGCATGTCCCATCAGAAAATTATTCAGGATCTTATCGCATGGATTGACGAGCATATTGACCAGCCGCTTAACATTGA<br>TGTAGTCGCAAAAAAATCAGGCTATTCAAAGTGGTACTTGCAACGAATGTTCCGCACGGTGACGCATCAGACGCTTGGCGATTACATT<br>CGCCAACGCCGCCTGTTACTGGCCGCCGTTGAGTTGCGCACCACCGAGCGTCCGATTTTTGATATCGCAATGGACCTGGGTTATGTC<br>TCGCAGCAGACCTTCTCCCGCGTTTTCGCGCGGCAGTTTGATCGCACTCCCGCGGATTATCGCCACCGCCTGTAAGCGGCCGCCAC<br>GCAAAAAACCCCGCTTCGGCGGGGTTTTTTCGCCCTAGGGATATATTCCGCTTCCTCGCTCACTGACTCGCTACGCTCGGTCGTTCG<br>ACTGCGGCGAGCGGAAATGGCTTACGAACGGGGCGGAGATTTCCTGGAAGATGCCAGGAAGATACTTAACAGGGAAGTGAGAGGGC<br>CGCGGCAAAGCCGTTTTTCCATAGGCTCCGCCCCCCTGACAAGCATCACGAAATCTGACGCTCAAATCAGTGGTGGCGAAACCCGAC<br>AGGACTATAAAGATACCAGGCGTTTCCCCCTGGCGGCTCCCTCGTGCGCTCTCCTGTTCCTGCCTTTCGGTTTACCGGTGTCATTCCG	

	CTGTTATGGCCGCGTTTGTCTCATTCCACGCCTGACACTCAGTTCCGGGTAGGCAGTTCGCTCCAAGCTGGACTGTATGCACGAACC<br>CCCCGTTCAGTCCGACCGCTGCGCCTTATCCGGTAACTATCGTCTTGAGTCCAACCCGGAAAGACATGCAAAAGCACCACTGGCAGC<br>AGCCACTGGTAATTGATTTAGAGGAGTTAGTCTTGAAGTCATGCGCCGGTTAAGGCTAAACTGAAAGGACAAGTTTTGGTGACTGCGC<br>TCCTCCAAGCCAGTTACCTCGGTTCAAAGAGTTGGTAGCTCAGAGAACCTTCGAAAAACCGCCCTGCAAGGCGGTTTTTTCGTTTTCA<br>GAGCAAGAGATTACGCGCAGACCAAAACGATCTCAAGAAGATCATCTTATTAATCAGATAAAATATTTCTAGATTTCAGTGCAATTTATC<br>TCTTCAAATGTAGCACCTGAAGTCAGCCCCATACGATATAAGTTGTTACTAGATTGACAGCTAGCTCAGTCCTAGGTATAATACTAGTA<br>GGACGCCTTTGGTAACCGCGTTTTAGAGCTAGAAATAGCAAGTTAAAATAAGGCTAGTCCGTTATCAACTTGAAAAAGTGGCACATGA<br>GGATCACCCATGTGCTTTTTTTGAAGCTTGGGCCCGAACAAAAACTCATCTCAGAAGAGGATCTGAATAGCGCCGTCGACCATCATCA<br>TCATCATCATTGAGTTTAAACGGTCTCCAGCTTGGCTGTTTTGGCGGATGAGAGAAGATTTTCAGCCTGATACAGATTAAATCAGAACG<br>CAGAAGCGGTCTGATAAAACAGAATTTGCCTGGCGGCAGTAGCGCGGTGGTCCCACCTGACCCCATGCCGAACTCAGAAGTGAAAC<br>GCCGTAGCGCCGATGGTAGTGTGGGGTCTCCCCATGCGAGAGTAGGGAACTGCCAGGCATCAAATAAAACGAAAGGCTCAGTCGAA<br>AGACTGGGCCTTTCGTTTTATCTGTTGTTTGTCGGTGAACTGGATCCTTACTCGAGTCTAGACTGCAGGCTTCCTCGCTCACTGACTC<br>GCTGCGCTCGGTCGTTCGGCTGCGGCGAGCGGTATCAGCTCACTCAAAGGCGGTAATACGGTTATCCACAGAATCAGGGGATAACG<br>CAGGAAAGAACATGTGAGCAAAAGGCCAGCAAAAGGCCAGGAACCGTAAAAAACTAGTGCTTGGATTCTCACCAATAAAAAACGCCC<br>GGCGGCAACCGAGCGTTCTGAACAAATCCAGATGGAGTTCTGAGGTCATTACTGGATCTATCAACAGGAGTCCAAGCGAGCTCGATA<br>TCAAATTACGCCCCGCCCTGCCACTCATCGCAGTACTGTTGTAATTCATTAAGCATTCTGCCGACATGGAAGCCATCACAAACGGCAT<br>GATGAACCTGAATCGCCAGCGGCATCAGCACCTTGTCGCCTTGCGTATAATATTTGCCCATGGTGAAAACGGGGGCGAAGAAGTTGT<br>CCATATTGGCCACGTTTAAATCAAAACTGGTGAAACTCACCCAGGGATTGGCTGAGACGAAAAACATATTCTCAATAAACCCTTTAGGG<br>AAATAGGCCAGGTTTTCACCGTAACACGCCACATCTTGCGAATATATGTGTAGAAACTGCCGGAAATCGTCGTGGTATTCACTCCAGA<br>GCGATGAAAACGTTTCAGTTTGCTCATGGAAAACGGTGTAACAAGGGTGAACACTATCCCATATCACCAGCTCACCGTCTTTCATTGC<br>CATACGAAATTCCGGATGAGCATTCATCAGGCGGGCAAGAATGTGAATAAAGGCCGGATAAAACTTGTGCTTATTTTTCTTTACGGTCT<br>TTAAAAAGGCCGTAATATCCAGCTGAACGGTCTGGTTATAGGTACATTGAGCAACTGACTGAAATGCCTCAAAATGTTCTTTACGATGC<br>CATTGGGATATATCAACGGTGGTATATCCAGTGATTTTTTTCTCCATTTTAGCTTCCTTAGCTCCTGAAAATCTCGATAACTCAAAAAAT<br>ACGCCCGGTAGTGATCTTATTTCATTATGGTGAAAGTTGGAACCTCTTACGTGCCGATCAACGTCTCATTTTCGCCAGATATC	
PUC19	GAGATACCTACAGCGTGAGCTATGAGAAAGCGCCACGCTTCCCGAAGGGAGAAAGGCGGACAGGTATCCGGTAAGCGGCAGGGTC<br>GGAACAGGAGAGCGCACGAGGGAGCTTCCAGGGGGAAACGCCTGGTATCTTTATAGTCCTGTCGGGTTTCGCCACCTCTGACTTGA<br>GCGTCGATTTTTGTGATGCTCGTCAGGGGGGCGGAGCCTATGGAAAAACGCCAGCAACGCGGCCTTTTTACGGTTCCTGGCCTTTTG<br>CTGGCCTTTTGCTCACATGTTCTTTCCTGCGTTATCCCCTGATTCTGTGGATAACCGTATTACCGCCTTTGAGTGAGCTGATACCGCTC<br>GCCGCAGCCGAACGACCGAGCGCAGCGAGTCAGTGAGCGAGGAAGCGGAAGAGCGCCCAATACGCAAACCGCCTCTCCCCGCGCG<br>TTGGCCGATTCATTAATGCAGCTGGCACGACAGGTTTCCCGACTGGAAAGCGGGCAGTGAGCGCAACGCAATTAATGTGAGTTAGCT<br>CACTCATTAGGCACCCCAGGCTTTACACTTTATGCTTCCGGCTCGTATGTTGTGTGGAATTGTGAGCGGATAACAATTTCACACAGGA<br>AACAGCTATGACCATGATTACGCCAAGCTTGCATGCCTGCAGGTCGACTCTAGAGGATCCCCGGGTACCGAGCTCGAATTCACTGGC<br>CGTCGTTTTACAACGTCGTGACTGGGAAAACCCTGGCGTTACCCAACTTAATCGCCTTGCAGCACATCCCCCTTTCGCCAGCTGGCGT<br>AATAGCGAAGAGGCCCGCACCGATCGCCCTTCCCAACAGTTGCGCAGCCTGAATGGCGAATGGCGCCTGATGCGGTATTTTCTCCTT<br>ACGCATCTGTGCGGTATTTCACACCGCATATGGTGCACTCTCAGTACAATCTGCTCTGATGCCGCATAGTTAAGCCAGCCCCGACACC<br>CGCCAACACCCGCTGACGCGCCCTGACGGGCTTGTCTGCTCCCGGCATCCGCTTACAGACAAGCTGTGACCGTCTCCGGGAGCTGC<br>ATGTGTCAGAGGTTTTCACCGTCATCACCGAAACGCGCGAGACGAAAGGGCCTCGTGATACGCCTATTTTTATAGGTTAATGTCATGA<br>TAATAATGGTTTCTTAGACGTCAGGTGGCACTTTTCGGGGAAATGTGCGCGGAACCCCTATTTGTTTATTTTTCTAAATACATTCAAATA<br>TGTATCCGCTCATGAGACAATAACCCTGATAAATGCTTCAATAATATTGAAAAAGGAAGAGTATGAGTATTCAACATTTCCGTGTCGCC<br>CTTATTCCCTTTTTTGCGGCATTTTGCCTTCCTGTTTTTGCTCACCCAGAAACGCTGGTGAAAGTAAAAGATGCTGAAGATCAGTTGGG<br>TGCACGAGTGGGTTACATCGAACTGGATCTCAACAGCGGTAAGATCCTTGAGAGTTTTCGCCCCGAAGAACGTTTTCCAATGATGAGC<br>ACTTTTAAAGTTCTGCTATGTGGCGCGGTATTATCCCGTATTGACGCCGGGCAAGAGCAACTCGGTCGCCGCATACACTATTCTCAGA<br>ATGACTTGGTTGAGTACTCACCAGTCACAGAAAAGCATCTTACGGATGGCATGACAGTAAGAGAATTATGCAGTGCTGCCATAACCAT<br>GAGTGATAACACTGCGGCCAACTTACTTCTGACAACGATCGGAGGACCGAAGGAGCTAACCGCTTTTTTGCACAACATGGGGGATCA<br>TGTAACTCGCCTTGATCGTTGGGAACCGGAGCTGAATGAAGCCATACCAAACGACGAGCGTGACACCACGATGCCTGTAGCAATGGC<br>AACAACGTTGCGCAAACTATTAACTGGCGAACTACTTACTCTAGCTTCCCGGCAACAATTAATAGACTGGATGGAGGCGGATAAAGTT<br>GCAGGACCACTTCTGCGCTCGGCCCTTCCGGCTGGCTGGTTTATTGCTGATAAATCTGGAGCCGGTGAGCGTGGGTCTCGCGGTAT<br>CATTGCAGCACTGGGGCCAGATGGTAAGCCCTCCCGTATCGTAGTTATCTACACGACGGGGAGTCAGGCAACTATGGATGAACGAAA<br>TAGACAGATCGCTGAGATAGGTGCCTCACTGATTAAGCATTGGTAACTGTCAGACCAAGTTTACTCATATATACTTTAGATTGATTTAAA<br>ACTTCATTTTTAATTTAAAAGGATCTAGGTGAAGATCCTTTTTGATAATCTCATGACCAAAATCCCTTAACGTGAGTTTTCGTTCCACTGA<br>GCGTCAGACCCCGTAGAAAAGATCAAAGGATCTTCTTGAGATCCTTTTTTTCTGCGCGTAATCTGCTGCTTGCAAACAAAAAAACCACC<br>GCTACCAGCGGTGGTTTGTTTGCCGGATCAAGAGCTACCAACTCTTTTTCCGAAGGTAACTGGCTTCAGCAGAGCGCAGATACCAAAT<br>ACTGTTCTTCTAGTGTAGCCGTAGTTAGGCCACCACTTCAAGAACTCTGTAGCACCGCCTACATACCTCGCTCTGCTAATCCTGTTACC<br>AGTGGCTGCTGCCAGTGGCGATAAGTCGTGTCTTACCGGGTTGGACTCAAGACGATAGTTACCGGATAAGGCGCAGCGGTCGGGCT<br>GAACGGGGGGTTCGTGCACACAGCCCAGCTTGGAGCGAACGACCTACACCGAACT	NEB

p106	GAGATACCTACAGCGTGAGCTATGAGAAAGCGCCACGCTTCCCGAAGGGAGAAAGGCGGACAGGTATCCGGTAAGCGGCAGGGTC<br>GGAACAGGAGAGCGCACGAGGGAGCTTCCAGGGGGAAACGCCTGGTATCTTTATAGTCCTGTCGGGTTTCGCCACCTCTGACTTGA<br>GCGTCGATTTTTGTGATGCTCGTCAGGGGGGCGGAGCCTATGGAAAAACGCCAGCAACGCGGCCTTTTTACGGTTCCTGGCCTTTTG<br>CTGGCCTTTTGCTCTCAGATAAAATATTTCTAGATTTCAGTGCAATTTATCTCTTCAAATGTAGCACCTGAAGTCAGCCCCATACGATAT<br>AAGTTGTTACTAGATTGACAGCTAGCTCAGTCCTAGGTATAATACTAGTAGGACGCCTTTGGTAACCGCGTTTTAGAGCTAGAAATAGC<br>AAGTTAAAATAAGGCTAGTCCGTTATCAACTTGAAAAAGTGGCACATGAGGATCACCCATGTGCTTTTTTTGAAGCTTGGGCCCGAACA<br>AAAACTCATCTCAGAAGAGGATCTGAATAGCGCCGTCGACCATCATCATCATCATCATTGAGTTTAAACGGTCTCCAGCTTGGCTGTTT<br>TGGCGGATGAGAGAAGATTTTCAGCCTGATACAGATTAAATCAGAACGCAGAAGCGGTCTGATAAAACAGAATTTGCCTGGCGGCAGT<br>AGCGCGGTGGTCCCACCTGACCCCATGCCGAACTCAGAAGTGAAACGCCGTAGCGCCGATGGTAGTGTGGGGTCTCCCCATGCGAG<br>AGTAGGGAACTGCCAGGCATCAAATAAAACGAAAGGCTCAGTCGAAAGACTGGGCCTTTCGTTTTATCTGTTGTTTGTCGGTGAACTG<br>GATCCTTACTCGAGTCTAGACTGCAGGCTTCCTCGCTCACTGACTCGCTGCGCTCGGTCGTTCGGCTGCGGCGAGCGGTATCAGCTC<br>ACTCAAAGGCGGTAATACGGTTATCCACAGAATCAGGGGATAACGCAGGAAAGAACATGTGAGCAAAAGGCCAGCAAAAGGCCAGGA<br>ACCGTAAAAAACTAGTGCTTGGATTCTCACCAATAAAAAACGCCCGGCGGCAACCGAGCGTTCTGAACAAATCCAGATGGAGTTCTGA<br>GGTCATTACTGGATCTATCAACAGGAGTCCAAGCGAGCTCGATATCAAATTACGCCCCGCCCTGCCACTCATCGCAGTACTGTTGTAA<br>TTCATTAAGCATTCTGCCGACATGGAAGCCATCACAAACGGCATGATGAACCTGAATCGCCAGCCGAGACGAAAGGGCCTCGTGATA<br>CGCCTATTTTTATAGGTTAATGTCATGATAATAATGGTTTCTTAGACGTCAGGTGGCACTTTTCGGGGAAATGTGCGCGGAACCCCTAT<br>TTGTTTATTTTTCTAAATACATTCAAATATGTATCCGCTCATGAGACAATAACCCTGATAAATGCTTCAATAATATTGAAAAAGGAAGAGT<br>ATGAGTATTCAACATTTCCGTGTCGCCCTTATTCCCTTTTTTGCGGCATTTTGCCTTCCTGTTTTTGCTCACCCAGAAACGCTGGTGAAA<br>GTAAAAGATGCTGAAGATCAGTTGGGTGCACGAGTGGGTTACATCGAACTGGATCTCAACAGCGGTAAGATCCTTGAGAGTTTTCGCC<br>CCGAAGAACGTTTTCCAATGATGAGCACTTTTAAAGTTCTGCTATGTGGCGCGGTATTATCCCGTATTGACGCCGGGCAAGAGCAACT<br>CGGTCGCCGCATACACTATTCTCAGAATGACTTGGTTGAGTACTCACCAGTCACAGAAAAGCATCTTACGGATGGCATGACAGTAAGA<br>GAATTATGCAGTGCTGCCATAACCATGAGTGATAACACTGCGGCCAACTTACTTCTGACAACGATCGGAGGACCGAAGGAGCTAACC<br>GCTTTTTTGCACAACATGGGGGATCATGTAACTCGCCTTGATCGTTGGGAACCGGAGCTGAATGAAGCCATACCAAACGACGAGCGT<br>GACACCACGATGCCTGTAGCAATGGCAACAACGTTGCGCAAACTATTAACTGGCGAACTACTTACTCTAGCTTCCCGGCAACAATTAA<br>TAGACTGGATGGAGGCGGATAAAGTTGCAGGACCACTTCTGCGCTCGGCCCTTCCGGCTGGCTGGTTTATTGCTGATAAATCTGGAG<br>CCGGTGAGCGTGGGTCTCGCGGTATCATTGCAGCACTGGGGCCAGATGGTAAGCCCTCCCGTATCGTAGTTATCTACACGACGGGG<br>AGTCAGGCAACTATGGATGAACGAAATAGACAGATCGCTGAGATAGGTGCCTCACTGATTAAGCATTGGTAACTGTCAGACCAAGTTT<br>ACTCATATATACTTTAGATTGATTTAAAACTTCATTTTTAATTTAAAAGGATCTAGGTGAAGATCCTTTTTGATAATCTCATGACCAAAATC<br>CCTTAACGTGAGTTTTCGTTCCACTGAGCGTCAGACCCCGTAGAAAAGATCAAAGGATCTTCTTGAGATCCTTTTTTTCTGCGCGTAAT<br>CTGCTGCTTGCAAACAAAAAAACCACCGCTACCAGCGGTGGTTTGTTTGCCGGATCAAGAGCTACCAACTCTTTTTCCGAAGGTAACT<br>GGCTTCAGCAGAGCGCAGATACCAAATACTGTTCTTCTAGTGTAGCCGTAGTTAGGCCACCACTTCAAGAACTCTGTAGCACCGCCTA<br>CATACCTCGCTCTGCTAATCCTGTTACCAGTGGCTGCTGCCAGTGGCGATAAGTCGTGTCTTACCGGGTTGGACTCAAGACGATAGTT<br>ACCGGATAAGGCGCAGCGGTCGGGCTGAACGGGGGGTTCGTGCACACAGCCCAGCTTGGAGCGAACGACCTACACCGAACT	This study
p105	GAGATACCTACAGCGTGAGCTATGAGAAAGCGCCACGCTTCCCGAAGGGAGAAAGGCGGACAGGTATCCGGTAAGCGGCAGGGTC<br>GGAACAGGAGAGCGCACGAGGGAGCTTCCAGGGGGAAACGCCTGGTATCTTTATAGTCCTGTCGGGTTTCGCCACCTCTGACTTGA<br>GCGTCGATTTTTGTGATGCTCGTCAGGGGGGCGGAGCCTATGGAAAAACGCCAGCAACGCGGCCTTTTTACGGTTCCTGGCCTTTTG<br>CTGGCCTTTTGCTCTCAGATAAAATATTTCTAGATTTCAGTGCAATTTATCTCTTCAAATGTAGCACCTGAAGTCAGCCCCATACGATAT<br>AAGTTGTTACTAGATTGACAGCTAGCTCAGTCCTAGGTATAATACTAGTCGGTTACCAAAGGCGTCCTTGTTTTAGAGCTAGAAATAGC<br>AAGTTAAAATAAGGCTAGTCCGTTATCAACTTGAAAAAGTGGCACATGAGGATCACCCATGTGCTTTTTTTGAAGCTTGGGCCCGAACA<br>AAAACTCATCTCAGAAGAGGATCTGAATAGCGCCGTCGACCATCATCATCATCATCATTGAGTTTAAACGGTCTCCAGCTTGGCTGTTT<br>TGGCGGATGAGAGAAGATTTTCAGCCTGATACAGATTAAATCAGAACGCAGAAGCGGTCTGATAAAACAGAATTTGCCTGGCGGCAGT<br>AGCGCGGTGGTCCCACCTGACCCCATGCCGAACTCAGAAGTGAAACGCCGTAGCGCCGATGGTAGTGTGGGGTCTCCCCATGCGAG<br>AGTAGGGAACTGCCAGGCATCAAATAAAACGAAAGGCTCAGTCGAAAGACTGGGCCTTTCGTTTTATCTGTTGTTTGTCGGTGAACTG<br>GATCCTTACTCGAGTCTAGACTGCAGGCTTCCTCGCTCACTGACTCGCTGCGCTCGGTCGTTCGGCTGCGGCGAGCGGTATCAGCTC<br>ACTCAAAGGCGGTAATACGGTTATCCACAGAATCAGGGGATAACGCAGGAAAGAACATGTGAGCAAAAGGCCAGCAAAAGGCCAGGA<br>ACCGTAAAAAACTAGTGCTTGGATTCTCACCAATAAAAAACGCCCGGCGGCAACCGAGCGTTCTGAACAAATCCAGATGGAGTTCTGA<br>GGTCATTACTGGATCTATCAACAGGAGTCCAAGCGAGCTCGATATCAAATTACGCCCCGCCCTGCCACTCATCGCAGTACTGTTGTAA<br>TTCATTAAGCATTCTGCCGACATGGAAGCCATCACAAACGGCATGATGAACCTGAATCGCCAGCCGAGACGAAAGGGCCTCGTGATA<br>CGCCTATTTTTATAGGTTAATGTCATGATAATAATGGTTTCTTAGACGTCAGGTGGCACTTTTCGGGGAAATGTGCGCGGAACCCCTAT<br>TTGTTTATTTTTCTAAATACATTCAAATATGTATCCGCTCATGAGACAATAACCCTGATAAATGCTTCAATAATATTGAAAAAGGAAGAGT<br>ATGAGTATTCAACATTTCCGTGTCGCCCTTATTCCCTTTTTTGCGGCATTTTGCCTTCCTGTTTTTGCTCACCCAGAAACGCTGGTGAAA<br>GTAAAAGATGCTGAAGATCAGTTGGGTGCACGAGTGGGTTACATCGAACTGGATCTCAACAGCGGTAAGATCCTTGAGAGTTTTCGCC<br>CCGAAGAACGTTTTCCAATGATGAGCACTTTTAAAGTTCTGCTATGTGGCGCGGTATTATCCCGTATTGACGCCGGGCAAGAGCAACT<br>CGGTCGCCGCATACACTATTCTCAGAATGACTTGGTTGAGTACTCACCAGTCACAGAAAAGCATCTTACGGATGGCATGACAGTAAGA<br>GAATTATGCAGTGCTGCCATAACCATGAGTGATAACACTGCGGCCAACTTACTTCTGACAACGATCGGAGGACCGAAGGAGCTAACC<br>GCTTTTTTGCACAACATGGGGGATCATGTAACTCGCCTTGATCGTTGGGAACCGGAGCTGAATGAAGCCATACCAAACGACGAGCGT<br>GACACCACGATGCCTGTAGCAATGGCAACAACGTTGCGCAAACTATTAACTGGCGAACTACTTACTCTAGCTTCCCGGCAACAATTAA	This study

	TAGACTGGATGGAGGCGGATAAAGTTGCAGGACCACTTCTGCGCTCGGCCCTTCCGGCTGGCTGGTTTATTGCTGATAAATCTGGAG<br>CCGGTGAGCGTGGGTCTCGCGGTATCATTGCAGCACTGGGGCCAGATGGTAAGCCCTCCCGTATCGTAGTTATCTACACGACGGGG<br>AGTCAGGCAACTATGGATGAACGAAATAGACAGATCGCTGAGATAGGTGCCTCACTGATTAAGCATTGGTAACTGTCAGACCAAGTTT<br>ACTCATATATACTTTAGATTGATTTAAAACTTCATTTTTAATTTAAAAGGATCTAGGTGAAGATCCTTTTTGATAATCTCATGACCAAAATC<br>CCTTAACGTGAGTTTTCGTTCCACTGAGCGTCAGACCCCGTAGAAAAGATCAAAGGATCTTCTTGAGATCCTTTTTTTCTGCGCGTAAT<br>CTGCTGCTTGCAAACAAAAAAACCACCGCTACCAGCGGTGGTTTGTTTGCCGGATCAAGAGCTACCAACTCTTTTTCCGAAGGTAACT<br>GGCTTCAGCAGAGCGCAGATACCAAATACTGTTCTTCTAGTGTAGCCGTAGTTAGGCCACCACTTCAAGAACTCTGTAGCACCGCCTA<br>CATACCTCGCTCTGCTAATCCTGTTACCAGTGGCTGCTGCCAGTGGCGATAAGTCGTGTCTTACCGGGTTGGACTCAAGACGATAGTT<br>ACCGGATAAGGCGCAGCGGTCGGGCTGAACGGGGGGTTCGTGCACACAGCCCAGCTTGGAGCGAACGACCTACACCGAACT	
pJF076Sa	GTCTGACCACTTCGGATTATCCCGTGACAGGTCATTCAGACTGGCTAATGCACCCAGTAAGGCAGCGGTATCATCAACAGGCTTACCC<br>GTCTTACTGTCCCTAGTGCTTGGATTCTCACCAATAAAAAACGCCCGGCGGCAACCGAGCGTTCTGAACAAATCCAGATGGAGTTCTG<br>AGGTCATTACTGGATCTATCAACAGGAGTCCAAGCGAGCTCGTAAACTTGGTCTGACAGTTACCAATGCTTAATCAGTGAGGCACCTA<br>TCTCAGCGATCTGTCTATTTCGTTCATCCATAGTTGCCTGACTCCCCGTCGTGTAGATAACTACGATACGGGAGGGCTTACCATCTGG<br>CCCCAGTGCTGCAATGATACCGCGAGACCCACGCTCACCGGCTCCAGATTTATCAGCAATAAACCAGCCAGCCGGAAGGGCCGAGC<br>GCAGAAGTGGTCCTGCAACTTTATCCGCCTCCATCCAGTCTATTAATTGTTGCCGGGAAGCTAGAGTAAGTAGTTCGCCAGTTAATAG<br>TTTGCGCAACGTTGTTGCCATTGCTACAGGCATCGTGGTGTCACGCTCGTCGTTTGGTATGGCTTCATTCAGCTCCGGTTCCCAACGA<br>TCAAGGCGAGTTACATGATCCCCCATGTTGTGCAAAAAAGCGGTTAGCTCCTTCGGTCCTCCGATCGTTGTCAGAAGTAAGTTGGCCG<br>CAGTGTTATCACTCATGGTTATGGCAGCACTGCATAATTCTCTTACTGTCATGCCATCCGTAAGATGCTTTTCTGTGACTGGTGAGTAC<br>TCAACCAAGTCATTCTGAGAATAGTGTATGCGGCGACCGAGTTGCTCTTGCCCGGCGTCAATACGGGATAATACCGCGCCACATAGC<br>AGAACTTTAAAAGTGCTCATCATTGGAAAACGTTCTTCGGGGCGAAAACTCTCAAGGATCTTACCGCTGTTGAGATCCAGTTCGATGTA<br>ACCCACTCGTGCACCCAACTGATCTTCAGCATCTTTTACTTTCACCAGCGTTTCTGGGTGAGCAAAAACAGGAAGGCAAAATGCCGCA<br>AAAAAGGGAATAAGGGCGACACGGAAATGTTGAATACTCATACTCTTCCTTTTTCAATATTATTGAAGCATTTATCAGGGTTATTGTCTC<br>ATGAGCGGATACATATTTGAATGTATTTAGAAAAATAAACAAATAGGGGTTCCGCGCACATTTCCCCGAAAAGTGCCACCTGTGGCAAT<br>TCCGACGTCGCCTACGGTATCCACCGGAGACCTATGGCAGCCTCCGGCCGCCATAGGACACCTTTGGTTGCCAAGGGTGACCTATG<br>GTGACCATGGGCCACCACGGGCGACCTCAGGTATCCTGCGGTGTCCTGCGGTTACCAAAGGCGTCCTTTGGGTTCCACCGGATACC<br>TCCGGACTTGACAGCTAGCTCAGTCCTAGGGATTGTGCTAGCGAATTCATTAAAGAGGAGAAAGGTACCATGGCGAGTAGCGAAGAC<br>GTTATCAAAGAGTTCATGCGTTTCAAAGTTCGTATGGAAGGTTCCGTTAACGGTCACGAGTTCGAAATCGAAGGTGAAGGTGAAGGTC<br>GTCCGTACGAAGGTACCCAGACCGCTAAACTGAAAGTTACCAAAGGTGGTCCGCTGCCGTTCGCTTGGGACATCCTGTCCCCGCAGT<br>TCCAGTACGGTTCCAAAGCTTACGTTAAACACCCGGCTGACATCCCGGACTACCTGAAACTGTCCTTCCCGGAAGGTTTCAAATGGGA<br>ACGTGTTATGAACTTCGAAGACGGTGGTGTTGTTACCGTTACCCAGGACTCCTCCCTGCAAGACGGTGAGTTCATCTACAAAGTTAAA<br>CTGCGTGGTACCAACTTCCCGTCCGACGGTCCGGTTATGCAGAAAAAAACCATGGGTTGGGAAGCTTCCACCGAACGTATGTACCCG<br>GAAGACGGTGCTCTGAAAGGTGAAATCAAAATGCGTCTGAAACTGAAAGACGGTGGTCACTACGACGCTGAAGTTAAAACCACCTACA<br>TGGCTAAAAAACCGGTTCAGCTGCCGGGTGCTTACAAAACCGACATCAAACTGGACATCACCTCCCACAACGAAGACTACACCATCGT<br>TGAACAGTACGAACGTGCTGAAGGTCGTCACTCCACCGGTGCTTAAGGATCCAAACTCGAGTAAGGATCTCCAGGCATCAAATAAAAC<br>GAAAGGCTCAGTCGAAAGACTGGGCCTTTCGTTTTATCTGTTGTTTGTCGGTGAACGCTCTCTACTAGAGTCACACTGGCTCACCTTC<br>GGGTGGGCCTTTCTGCGTTTATACCTAGGGTACGGGTTTTGCTGCCCGCAAACGGGCTGTTCTGGTGTTGCTAGTTTGTTATCAGAAT<br>CGCAGATCCGGCTTCAGCCGGTTTGCCGGCTGAAAGCGCTATTTCTTCCAGAATTGCCATGATTTTTTCCCCACGGGAGGCGTCACT<br>GGCTCCCGTGTTGTCGGCAGCTTTGATTCGATAAGCAGCATCGCCTGTTTCAGGCTGTCTATGTGTGACTGTTGAGCTGTAACAAGTT<br>GTCTCAGGTGTTCAATTTCATGTTCTAGTTGCTTTGTTTTACTGGTTTCACCTGTTCTATTAGGTGTTACATGCTGTTCATCTGTTACATT<br>GTCGATCTGTTCATGGTGAACAGCTTTGAATGCACCAAAAACTCGTAAAAGCTCTGATGTATCTATCTTTTTTACACCGTTTTCATCTGT<br>GCATATGGACAGTTTTCCCTTTGATATGTAACGGTGAACAGTTGTTCTACTTTTGTTTGTTAGTCTTGATGCTTCACTGATAGATACAAG<br>AGCCATAAGAACCTCAGATCCTTCCGTATTTAGCCAGTATGTTCTCTAGTGTGGTTCGTTGTTTTTGCGTGAGCCATGAGAACGAACCA<br>TTGAGATCATACTTACTTTGCATGTCACTCAAAAATTTTGCCTCAAAACTGGTGAGCTGAATTTTTGCAGTTAAAGCATCGTGTAGTGTT<br>TTTCTTAGTCCGTTATGTAGGTAGGAATCTGATGTAATGGTTGTTGGTATTTTGTCACCATTCATTTTTATCTGGTTGTTCTCAAGTTCG<br>GTTACGAGATCCATTTGTCTATCTAGTTCAACTTGGAAAATCAACGTATCAGTCGGGCGGCCTCGCTTATCAACCACCAATTTCATATT<br>GCTGTAAGTGTTTAAATCTTTACTTATTGGTTTCAAAACCCATTGGTTAAGCCTTTTAAACTCATGGTAGTTATTTTCAAGCATTAACATG<br>AACTTAAATTCATCAAGGCTAATCTCTATATTTGCCTTGTGAGTTTTCTTTTGTGTTAGTTCTTTTAATAACCACTCATAAATCCTCATAGA<br>GTATTTGTTTTCAAAAGACTTAACATGTTCCAGATTATATTTTATGAATTTTTTTAACTGGAAAAGATAAGGCAATATCTCTTCACTAAAAA<br>CTAATTCTAATTTTTCGCTTGAGAACTTGGCATAGTTTGTCCACTGGAAAATCTCAAAGCCTTTAACCAAAGGATTCCTGATTTCCACAG<br>TTCTCGTCATCAGCTCTCTGGTTGCTTTAGCTAATACACCATAAGCATTTTCCCTACTGATGTTCATCATCTGAGCGTATTGGTTATAAG<br>TGAACGATACCGTCCGTTCTTTCCTTGTAGGGTTTTCAATCGTGGGGTTGAGTAGTGCCACACAGCATAAAATTAGCTTGGTTTCATGC<br>TCCGTTAAGTCATAGCGACTAATCGCTAGTTCATTTGCTTTGAAAACAACTAATTCAGACATACATCTCAATTGGTCTAGGTGATTTTAA<br>TCACTATACCAATTGAGATGGGCTAGTCAATGATAATTACTAGTCCTTTTCCCGGGTGATCTGGGTATCTGTAAATTCTGCTAGACCTTT<br>GCTGGAAAACTTGTAAATTCTGCTAGACCCTCTGTAAATTCCGCTAGACCTTTGTGTGTTTTTTTTGTTTATATTCAAGTGGTTATAATTT<br>ATAGAATAAAGAAAGAATAAAAAAAGATAAAAAGAATAGATCCCAGCCCTGTGTATAACTCACTACTTTAGTCAGTTCCGCAGTATTACA<br>AAAGGATGTCGCAAACGCTGTTTGCTCCTCTACAAAACAGACCTTAAAACCCTAAAGGCTTAAGTAGCACCCTCGCAAGCTCGGGCAA<br>ATCGCTGAATATTCCTTTTGTCTCCGACCATCAGGCACCTGAGTCGCTGTCTTTTTCGTGACATTCAGTTCGCTGCGCTCACGGCTCT	Addgene, #113322

	GGCAGTGAATGGGGGTAAATGGCACTACAGGCGCCTTTTATGGATTCATGCAAGGAAACTACCCATAATACAAGAAAAGCCCGTCAC<br>GGGCTTCTCAGGGCGTTTTATGGCGGGTCTGCTATGTGGTGCTATCTGACTTTTTGCTGTTCAGCAGTTCCTGCCCTCTGATTTTCCA	
pJ-105	GAGATACCTACAGCGTGAGCTATGAGAAAGCGCCACGCTTCCCGAAGGGAGAAAGGCGGACAGGTATCCGGTAAGCGGCAGGGTC<br>GGAACAGGAGAGCGCACGAGGGAGCTTCCAGGGGGAAACGCCTGGTATCTTTATAGTCCTGTCGGGTTTCGCCACCTCTGACTTGA<br>GCGTCGATTTTTGTGATGCTCGTCAGGGGGGCGGAGCCTATGGAAAAACGCCAGCAACGCGGCCTTTTTACGGTTCCTGGCCTTTTG<br>CTGGCCTTTTGCTCTCAGATAAAATATTTCTAGATTTCAGTGCAATTTATCTCTTCAAATGTAGCACCTGAAGTCAGCCCCATACGATAT<br>AAGTTGTTACTAGATTGACAGCTAGCTCAGTCCTAGGTATAATACTAGTCGGTTACCAAAGGCGTCCTTGTTTTAGAGCTAGAAATAGC<br>AAGTTAAAATAAGGCTAGTCCGTTATCAACTTGAAAAAGTGGCACATGAGGATCACCCATGTGCTTTTTTTGAAGCTTGGGCCCGAACA<br>AAAACTCATCTCAGAAGAGGATCTGAATAGCGCCGTCGACCATCATCATCATCATCATTGAGTTTAAACGGTCTCCAGCTTGGCTGTTT<br>TGGCGGATGAGAGAAGATTTTCAGCCTGATACAGATTAAATCAGAACGCAGAAGCGGTCTGATAAAACAGAATTTGCCTGGCGGCAGT<br>AGCGCGGTGGTCCCACCTGACCCCATGCCGAACTCAGAAGTGAAACGCCGTAGCGCCGATGGTAGTGTGGGGTCTCCCCATGCGAG<br>AGTAGGGAACTGCCAGGCATCAAATAAAACGAAAGGCTCAGTCGAAAGACTGGGCCTTTCGTTTTATCTGTTGTTTGTCGGTGAACTG<br>GATCCTTACTCGAGTCTAGACTGCAGGCTTCCTCGCTCACTGACTCGCTGCGCTCGGTCGTTCGGCTGCGGCGAGCGGTATCAGCTC<br>ACTCAAAGGCGGTAATACGGTTATCCACAGAATCAGGGGATAACGCAGGAAAGAACATGTGAGCAAAAGGCCAGCAAAAGGCCAGGA<br>ACCGTAAAAAACTAGTGCTTGGATTCTCACCAATAAAAAACGCCCGGCGGCAACCGAGCGTTCTGAACAAATCCAGATGGAGTTCTGA<br>GGTCATTACTGGATCTATCAACAGGAGTCCAAGCGAGCTCGATATCAAATTACGCCCCGCCCTGCCACTCATCGCAGTACTGTTGTAA<br>TTCATTAAGCATTCTGCCGACATGGAAGCCATCACAAACGGCATGATGAACCTGAATCGCCAGCCGAGACGAAAGGGCCTCGTGATA<br>CGCCTATTTTTATAGGTTAATGTCATGATAATAATGGTTTCTTACCTCCCGTGGGGAAAAAATCATGGCAATTCTGGAAGAAATAGCGC<br>TTTCAGCCGGCAAACCGGCTGAAGCCGGATCTGCGATTCTGATAACAAACTAGCAACACCAGAACAGCCCGTTTGCGGGCAGCAAAA<br>CCCGTACCCTAGGTATAAACGCAGAAAGGCCCACCCGAAGGTGAGCCAGTGTGACTCTAGTAGAGAGCGTTCACCGACAAACAACAG<br>ATAAAACGAAAGGCCCAGTCTTTCGACTGAGCCTTTCGTTTTATTTGATGCCTGGAGATCCTTACTCGAGTTTGGATCCTTAAGCACCG<br>GTGGAGTGACGACCTTCAGCACGTTCGTACTGTTCAACGATGGTGTAGTCTTCGTTGTGGGAGGTGATGTCCAGTTTGATGTCGGTTT<br>TGTAAGCACCCGGCAGCTGAACCGGTTTTTTAGCCATGTAGGTGGTTTTAACTTCAGCGTCGTAGTGACCACCGTCTTTCAGTTTCAG<br>ACGCATTTTGATTTCACCTTTCAGAGCACCGTCTTCCGGGTACATACGTTCGGTGGAAGCTTCCCAACCCATGGTTTTTTTCTGCATAA<br>CCGGACCGTCGGACGGGAAGTTGGTACCACGCAGTTTAACTTTGTAGATGAACTCACCGTCTTGCAGGGAGGAGTCCTGGGTAACG<br>GTAACAACACCACCGTCTTCGAAGTTCATAACACGTTCCCATTTGAAACCTTCCGGGAAGGACAGTTTCAGGTAGTCCGGGATGTCAG<br>CCGGGTGTTTAACGTAAGCTTTGGAACCGTACTGGAACTGCGGGGACAGGATGTCCCAAGCGAACGGCAGCGGACCACCTTTGGTA<br>ACTTTCAGTTTAGCGGTCTGGGTACCTTCGTACGGACGACCTTCACCTTCACCTTCGATTTCGAACTCGTGACCGTTAACGGAACCTT<br>CCATACGAACTTTGAAACGCATGAACTCTTTGATAACGTCTTCGCTACTCGCCATGGTACCTTTCTCCTCTTTAATGAATTCGCTAGCA<br>CAATCCCTAGGACTGAGCTAGCTGTCAAGTCCGGAGGTATCCGGTGGAACCCAAAGGACGCCTTTGGTAACCGCAGGACACCGCAG<br>GATACCTGAGGTCGCCCGTGGTGGCCCATGGTCACCATAGGTCACCCTTGGCAACCAAAGGTGTCCTATGGCGGCCGGAGGCTGCC<br>ATAGGTCTCCGGTGGATACCGTAGGCGACGTCATCTAGATGCATTCGCGAGGTACCGAGCTCGAATTCACTGGCCGTCGTTTTACAA<br>CGTCGTGACTGGGAAAACCCTGGCGTTACCCAACTTAATCGCCTTGCAGCACATCCCCCTTTCGCCAGCTGGCGTAATAGCGAAGAG<br>GCCCGCACCGATCGCCCTTCCCAACAGTTGCGCAGCCTGAATGGCGAATGGCGCCTGATGCGGTATTTTCTCCTTACGCATCTGTGC<br>GGTATTTCACACCGCATATGGTGCACTCTCAGTACAATCTGCTCTGATGCCGCATAGTTAAGCCAGCCCCGACACCCGCCAACACCC<br>GCTGACGCGCCCTGACGGGCTTGTCTGCTCCCGGCATCCGCTTACAGACAAGCTGTGACCGTCTCCGGGAGCTGCATGTGTCAGAG<br>GTTTTCACCGTCATCACCGAAACGCGCGAGACGAAAGGGCCTCGTGATACGCCTATTTTTATAGGTTAATGTCATGATAATAATGGTTT<br>CTTAGACGTCAGGTGGCACTTTTCGGGGAAATGTGCGCGGAACCCCTATTTGTTTATTTTTCTAAATACATTCAAATATGTATCCGCTC<br>ATGAGACAATAACCCTGATAAATGCTTCAATAATATTGAAAAAGGAAGAGTATGAGTATTCAACATTTCCGTGTCGCCCTTATTCCCTTT<br>TTTGCGGCATTTTGCCTTCCTGTTTTTGCTCACCCAGAAACGCTGGTGAAAGTAAAAGATGCTGAAGATCAGTTGGGTGCACGAGTGG<br>GTTACATCGAACTGGATCTCAACAGCGGTAAGATCCTTGAGAGTTTTCGCCCCGAAGAACGTTTTCCAATGATGAGCACTTTTAAAGTT<br>CTGCTATGTGGCGCGGTATTATCCCGTATTGACGCCGGGCAAGAGCAACTCGGTCGCCGCATACACTATTCTCAGAATGACTTGGTT<br>GAGTACTCACCAGTCACAGAAAAGCATCTTACGGATGGCATGACAGTAAGAGAATTATGCAGTGCTGCCATAACCATGAGTGATAACA<br>CTGCGGCCAACTTACTTCTGACAACGATCGGAGGACCGAAGGAGCTAACCGCTTTTTTGCACAACATGGGGGATCATGTAACTCGCC<br>TTGATCGTTGGGAACCGGAGCTGAATGAAGCCATACCAAACGACGAGCGTGACACCACGATGCCTGTAGCAATGGCAACAACGTTGC<br>GCAAACTATTAACTGGCGAACTACTTACTCTAGCTTCCCGGCAACAATTAATAGACTGGATGGAGGCGGATAAAGTTGCAGGACCACT<br>TCTGCGCTCGGCCCTTCCGGCTGGCTGGTTTATTGCTGATAAATCTGGAGCCGGTGAGCGTGGGTCTCGCGGTATCATTGCAGCACT<br>GGGGCCAGATGGTAAGCCCTCCCGTATCGTAGTTATCTACACGACGGGGAGTCAGGCAACTATGGATGAACGAAATAGACAGATCGC<br>TGAGATAGGTGCCTCACTGATTAAGCATTGGTAACTGTCAGACCAAGTTTACTCATATATACTTTAGATTGATTTAAAACTTCATTTTTAA<br>TTTAAAAGGATCTAGGTGAAGATCCTTTTTGATAATCTCATGACCAAAATCCCTTAACGTGAGTTTTCGTTCCACTGAGCGTCAGACCC<br>CGTAGAAAAGATCAAAGGATCTTCTTGAGATCCTTTTTTTCTGCGCGTAATCTGCTGCTTGCAAACAAAAAAACCACCGCTACCAGCGG<br>TGGTTTGTTTGCCGGATCAAGAGCTACCAACTCTTTTTCCGAAGGTAACTGGCTTCAGCAGAGCGCAGATACCAAATACTGTTCTTCTA<br>GTGTAGCCGTAGTTAGGCCACCACTTCAAGAACTCTGTAGCACCGCCTACATACCTCGCTCTGCTAATCCTGTTACCAGTGGCTGCTG<br>CCAGTGGCGATAAGTCGTGTCTTACCGGGTTGGACTCAAGACGATAGTTACCGGATAAGGCGCAGCGGTCGGGCTGAACGGGGGGT<br>TCGTGCACACAGCCCAGCTTGGAGCGAACGACCTACACCGAACT	This study

AVA	AAACCACCGCTACCAGCGGTGGTTTGTTTGCCGGATCAAGAGCTACCAACTCTTTTTCCGAAGGTAACTGGCTTCAGCAGAGCGCAG<br>ATACCAAATACTGTTCTTCTAGTGTAGCCGTAGTTAGGCCACCACTTCAAGAACTCTGTAGCACCGCCTACATACCTCGCTCTGCTAAT<br>CCTGTTACCAGTGGCTGCTGCCAGTGGCGATAAGTCGTGTCTTACCGGGTTGGACTCAAGACGATAGTTACCGGATAAGGCGCAGCG<br>GTCGGGCTGAACGGGGGGTTCGTGCACACAGCCCAGCTTGGAGCGAACGACCTACACCGAACTGAGATACCTACAGCGTGAGCTAT<br>GAGAAAGCGCCACGCTTCCCGAAGGGAGAAAGGCGGACAGGTATCCGGTAAGCGGCAGGGTCGGAACAGGAGAGCGCACGAGGG<br>AGCTTCCAGGGGGAAACGCCTGGTATCTTTATAGTCCTGTCGGGTTTCGCCACCTCTGACTTGAGCGTCGATTTTTGTGATGCTCGTC<br>AGGGGGGCGGAGCCTATGGAAAAACGCCAGCAACGCGGCCTTTTTACGGTTCCTGGCCTTTTGCTGGCCTTTTGCTCATGATATAATT<br>AAATTGAAGCTCTAATTTGTGAGTTTAGTATACATGCATTTACTTATAATACAGTTTTTTAGTTTTGCTGGCCGCATCTTCTCAAATATGC<br>TTCCCAGCCTGCTTTTCTGTAACGTTCACCCTCTACCTTAGCATCCCTTCCCTTTGCAAATAGTCCTCTTCCAACAATAATAATGTCAGA<br>TCCTGTAGAGACCACATCATCCACGGTTCTATACTGTTGACCCAATGCGTCTCCCTTGTCATCTAAACCCACACCGGGTGTCATAATCA<br>ACCAATCGTAACCTTCATCTCTTCCACCCATGTCTCTTTGAGCAATAAAGCCGATAACAAAATCTTTGTCGCTCTTCGCAATGTCAACA<br>GTACCCTTAGTATATTCTCCAGTAGATAGGGAGCCCTTGCATGACAATTCTGCTAACATCAAAAGGCCTCTAGGTTCCTTTGTTACTTC<br>TTCTGCCGCCTGCTTCAAACCGCTAACAATACCTGGGCCCACCACACCGTGTGCATTCGTAATGTCTGCCCATTCTGCTATTCTGTATA<br>CACCCGCAGAGTACTGCAATTTGACTGTATTACCAATGTCAGCAAATTTTCTGTCTTCGAAGAGTAAAAAATTGTACTTGGCGGATAAT<br>GCCTTTAGCGGCTTAACTGTGCCCTCCATGGAAAAATCAGTCAAGATATCCACATGTGTTTTTAGTAAACAAATTTTGGGACCTAATGC<br>TTCAACTAACTCCAGTAATTCCTTGGTGGTACGAACATCCAATGAAGCACACAAGTTTGTTTGCTTTTCGTGCATGATATTAAATAGCTT<br>GGCAGCAACAGGACTAGGATGAGTAGCAGCACGTTCCTTATATGTAGCTTTCGACATGATTTATCTTCGTTTCCTGAGGTTTTTGTTCT<br>GTGCAGTTGGGTTAAGAATACTGGGCAATTTCATGTTTCTTCAACACTACATATGCGTATATATACCAATCTAAGTCTGTGCTCCTTCCT<br>TCGTTCTTCCTTCTGTTCGGAGATTACCGAATCAAAAAAATTTCAAAGAAACCGAAATCAAAAAAAAGAATAAAAAAAAAATGATGAATT<br>GAATTGAAAAGCTAGCTTATCGATGATAAGCTGTCAAAGATGAGAATTAATTCCACGGACTATAGACTATACTAGATACTCCGTCTACT<br>GTACGATACACTTCCGCTCAGGTCCTTGTCCTTTAACGAGGCCTTACCACTCTTTTGTTACTCTATTGATCCAGCTCAGCAAAGGCAGT<br>GTGATCTAAGATTCTATCTTCGCGATGTAGTAAAACTAGCTAGACCGAGAAAGAGACTAGAAATGCAAAAGGCACTTCTACAATGGCT<br>GCCATCATTATTATCCGATGTGACGCTGCAGCTTCTCAATGATATTCGAATACGCTTTGAGGAGATACAGCCTAATATCCGACAAACTG<br>TTTTACAGATTTACGATCGTACTTGTTACCCATCATTGAATTTTGAACATCCGAACCTGGGAGTTTTCCCTGAAACAGATAGTATATTTG<br>AACCTGTATAATAATATATAGTCTAGCGCTTTACGGAAGACAATGTATGTATTTCGGTTCCTGGAGAAACTATTGCATCTATTGCATAGG<br>TAATCTTGCACGTCGCATCCCCGGTTCATTTTCTGCGTTTCCATCTTGCACTTCAATAGCATATCTTTGTTAACGAAGCATCTGTGCTTC<br>ATTTTGTAGAACAAAAATGCAACGCGAGAGCGCTAATTTTTCAAACAAAGAATCTGAGCTGCATTTTTACAGAACAGAAATGCAACGCG<br>AAAGCGCTATTTTACCAACGAAGAATCTGTGCTTCATTTTTGTAAAACAAAAATGCAACGCGACGAGAGCGCTAATTTTTCAAACAAAG<br>AATCTGAGCTGCATTTTTACAGAACAGAAATGCAACGCGAGAGCGCTATTTTACCAACAAAGAATCTATACTTCTTTTTTGTTCTACAAA<br>AATGCATCCCGAGAGCGCTATTTTTCTAACAAAGCATCTTAGATTACTTTTTTTCTCCTTTGTGCGCTCTATAATGCAGTCTCTTGATAA<br>CTTTTTGCACTGTAGGTCCGTTAAGGTTAGAAGAAGGCTACTTTGGTGTCTATTTTCTCTTCCATAAAAAAAGCCTGACTCCACTTCCC<br>GCGTTTACTGATTACTAGCGAAGCTGCGGGTGCATTTTTTCAAGATAAAGGCATCCCCGATTATATTCTATACCGATGTGGATTGCGCA<br>TACTTTGTGAACAGAAAGTGATAGCGTTGATGATTCTTCATTGGTCAGAAAATTATGAACGGTTTCTTCTATTTTGTCTCTATATACTAC<br>GTATAGGAAATGTTTACATTTTCGTATTGTTTTCGATTCACTCTATGAATAGTTCTTACTACAATTTTTTTGTCTAAAGAGTAATACTAGA<br>GATAAACATAAAAAATGTAGAGGTCGAGTTTAGATGCAAGTTCAAGGAGCGAAAGGTGGATGGGTAGGTTATATAGGGATATAGCACA<br>GAGATATATAGCAAAGAGATACTTTTGAGCAATGTTTGTGGAAGCGGTATTCGCAATGGGAAGCTCCACCCCGGTTGATAATCAGAAA<br>AGCCCCAAAAACAGGAAGATTGTATAAGCAAATATTTAAATTGTAATTCTTTCCTGCGTTATCCCCTGATTCTGTGGATAACCGTATTAC<br>CGCCTTTGAGTGAGCTGATACCGCTCGCCGCAGCCGAACGACCGAGCGCAGCGAGTCAGTGAGCGAGGAAGCGGAAGAGGGTCTG<br>ACGCTCAGTGGAACGAAAACTCACGTTAAGGGATTTTGGTCATGAGATTATCAAAAAGGATCTTCACCTAGATCCTTTTAAATTAAAAAT<br>GAAGTTTTAAATCAATCTAAAGTATATATGAGTAAACTTGGTCTGACAGTCAGAAGAACTCGTCAAGAAGGCGATAGAAGGCGATGCG<br>CTGCGAATCGGGAGCGGCGATACCGTAAAGCACGAGGAAGCGGTCAGCCCATTCGCCGCCAAGCTCTTCAGCAATATCACGGGTAG<br>CCAACGCTATGTCCTGATAGCGGTCCGCCACACCCAGCCGGCCACAGTCGATGAATCCAGAAAAGCGGCCATTTTCCACCATGATAT<br>TCGGCAAGCAGGCATCGTCATGGGTCACGACGAGATCCTCGCCGTCGGGCATGCTCGCCTTGAGCCTGGCGAACAGTTCGGCTGGC<br>GCGAGCCCCTGATGCTCTTCGTCCAGATCATCCTGATCGACAAGACCGGCTTCCATCCGAGTACGTGCTCGCTCGATGCGATGTTTC<br>GCTTGGTGGTCGAATGGGCAGGTAGCCGGATCAAGCGTATGCAGCCGCCGCATTGCATCAGCCATGATGGATACTTTCTCGGCAGG<br>AGCAAGGTGAGATGACAGGAGATCCTGCCCCGGCACTTCGCCCAATAGCAGCCAGTCCCTTCCCGCTTCAGTGACAACGTCGAGCA<br>CAGCTGCGCAAGGAACGCCCGTCGTGGCCAGCCACGATAGCCGCGCTGCCTCGTCTTGCAGTTCATTCAGGGCACCGGACAGGTCG<br>GTCTTGACAAAAAGAACCGGGCGCCCCTGCGCTGACAGCCGGAACACGGCGGCATCAGAGCAGCCGATTGTCTGTTGTGCCCAGTC<br>ATAGCCGAATAGCCTCTCCACCCAAGCGGCCGGAGAACCTGCGTGCAATCCATCTTGTTCAATCATACTCTTCCTTTTTCAATATTATT<br>GAAGCATTTATCAGGGTTATTGTCTCATGAGCGGATACATATTTGAATGTATTTAGAAAAATAAACAAATAGGGGTTCCGCGCACATTT<br>CCCCGAAAAGTGCCACCTGCGGACGGATCGCTTGCCTGTAACTTACACGCGCCTCGTCCTGAGAAAGCAACCTGACCTACAGGAAAG<br>AGTTACTCAAGAATAAGAATTTTCGTTTTAAAACCTAAGAGTCACTTTAAAATTTGTATACACTTATTTTTTTTATAACTTATTTAATAATAA<br>AAATCATAAATCATAAGAAATTCGCTTATTTAGAAGTGTCAACAACGTATCTACCAACGATTTGACCCTTTTCCATCTTTTCGTAAATTTC<br>TGGCAAGGTAGACAAGCCGACAACCTTGATTGGAGACTTGACCAAACCTCTGGCGAAGAATTGTTAATATTAAGAGCTCTCACTGCCC<br>ACTTTCCAGTCTGGAAACCTGTCTTGCTAGCTGCATGAGTGAATCAGCCAATGCCCGGGGAGAGGCAGTTTGTGTATTGGGTGCCAG<br>GGTGGTTTTTCTCTTCACCAGTGAGACTGGCAACAGCTGATTGCCCTTCACCGCCTGGCCCTGAGAGAGTTGCAGCAAGCGGTCCAC<br>GCTGGTTTGCCCCAGCAGGCGAAAATCCTGTTTGATGGTGGTTAACGGCGGGATATAACATGAGCTGTCTTCGGTATCGTCGTATCC	Naseri *et al.*, 2019[7]

	CACTACCGAGATATCTGCACCAACTCTCAGCCCAGACTCAGTAATGGCTCTCATTGCACCCAGTGCCATCTGATCATTGGCAACCAGC<br>ATTGCAGTGGGAACAATGCCCTCATTCAGCATTTGCATGGTTTGTTGAAACCCAGACATGGCACTCCAGTCCCCTTCTCTTTCAGCTAT<br>TGGCTGAATTTGATTCCTAGTGAGATATTTATGCCAGCCGGCCAGTCTCAGCCTTGCTGAGACAGAACTGAGTGGGCCCGCAAGCAG<br>TGCAATTTGCTGGTGTCCCAATGCAACCAGATGCTCCACACCCAGTCTTGTACCATCTTCATGGGAGAAAATAATACTGTTGATGGGT<br>GTCTGGTCAGAGACATCAAGAAAGAGTGCTGGAACATTAGTGCAGGCAGCTTCCACAGCAATGGCATCCTGGTCATCCAGTGGATAG<br>TTAATGATCAGCCCACTGACTCTTTGTGCCAGAAGATTGTGCACAGCAGCTTTACAGGCTTCAACTCCACTTCTTTCTACCATTGACAC<br>CACCACAGAGGCTCCCAGTTGATCAGCTCTAGATTTAATGGCTGCCACAATTTGAGATGGTGCATGCAGGGCCAGACTGGAGGTGGC<br>AACTCCAATCAGCAAGCTCTGTTTGCCTGCCAGTTGTTGTGCCACTCTGTTGGGAATGTAATTCAGCTCTGCCATGGCTGCTTCCACTT<br>TTTCCCTGGTTTTGGCAGAAACATGGCTGGCCTGGTTCACCACTCTGGAAACAGTCTGATAAGAGACACCGGCATACTCTGCGACATC<br>ATACAATGTTACTGGTTTCATTTTAATAACTAGTAAAACTTAGATTAGATTGCTATGCTTTCTTTCTAATGAGCAAGAAGTAAAAAAAGTT<br>GTAATAGAACAAGAAAAATGAAACTGAAACTTGAGAAATTGAAGACCGTTTATTAACTTAAATATCAATGGGAGGTCATCGAAAGAGAA<br>AAAAATCAAAAAAAAAATTTTCAAGAAAAAGAAACGTGATAAAAATTTTTATTGCCTTTTTCGACGAAGAAAAAGAAACGAGGCGGTCTC<br>TTTTTTCTTTTCCAAACCTTTAGTACGGGTAATTAACGACACCCTAGAGGAAGAAAGAGGGGAAATTTAGTATGCTGTGCTTGGGTGTT<br>TTGAAGTGGTACGGCGATGCGCGGAGTCCGAGAAAATCTGGAAGAGTAAAAAAGGAGTAGAAACATTTTGAAGCTATGGTGTTCGAG<br>TTCAAGAGAAAAAAAAAGAAAAAGCAAAAAGAAAAAAGGAAAGCGCGCCTCGTTCAGAATGACACGTATAGAATGATGCATTACCTTGT<br>CATCTTCAGTATCATACTGTTCGTATACATACTTACTGACATTCATAGGTATACATATATACACATGTATATATATCGTATGCTGCAGCTT<br>TAAATAATCGGTGTCACTACATAAGAACACCTTTGGTGGAGGGAACATCGTTGGTACCATTGGGCGAGGTGGCTTCTCTTATGGCAAC<br>CGCAAGAGCCTTGAACGCACTCTCACTACGGTGATGATCATTCTTGCCTCGCAGACAATCAACGTGGAGGGTAATTCTGCTAGCCTCT<br>GCAAAGCTTTCAAGAAAATGCGGGATCATCTCGCAAGAGAGATCTCCTACTTTCTCCCTTTGCAAACCAAGTTCGACAACTGCGTACG<br>GCCTGTTCGAAAGATCTACCACCGCTCTGGAAAGTGCCTCATCCAAAGGCGCAAATCCTGATCCAAACCTTTTTACTCCACGCGCCAG<br>TAGGGCCTCTTTAAAAGCTTGACCGAGAGCAATCCCGCAGTCTTCAGTGGTGTGATGGTCGTCTATGTGTAAGTCACCAATGCACTCA<br>ACGATTAGCGACCAGCCGGAATGCTTGGCCAGAGCATGTATCATATGGTCCAGAAACCCTATACCTGTGTGGACGTTAATCACTTGCG<br>ATTGTGTGGCCTGTTCTGCTACTGCTTCTGCCTCTTTTTCTGGGAAGATCGAGTGCTCTATCGCTAGGGGACCACCCTTTAAAGAGAT<br>CGCAATCTGAATCTTGGTTTCATTTGTAATACGCTTTACTAGGGCTTTCTGCTCTGTCATCGCGGCCGCCCAAGCTGCCTTTGTGTGCT<br>TAATCACGTCTTTGCCTTCGTTTATCTTGCCTGCTCATTTTTTAGTATATTCTTCGAAGAAATCACATTACTTTATATAATGTATAATTCAT<br>TATGTGATAATGCCAATCGCTAAGAAAAAAAAAGAGTCATCCGCTAGGGGAAAAAAAAAAATGAAAATCATTACCGAGGCATAAAAAAA<br>TATAGAGTGTACTAGAGGAGGCCAAGAGTAATAGAAAAAGAAAATTGCGGGAAAGGACTGTGTTATGACTTCCCTGACTAATGCCGTG<br>TTCAAACGATACCTGGCAGTGACTCCTAGCGCTCACCAAGCTCTTAAAACGGGACTCAGTACAATCTGCTCTGATGCCGCATAGTTAA<br>GCCCTGCAGGGAATTGGCCGGCCGGATCCGGCGCGCCTTCAACATTTCCGTGTCGCCCTTATTCCCTTTTTTGCGGCATTTTGCCTT<br>CCTGTTTTTGCTCACCCAGAAACGCTGGTGAAAGTAAAAGATGCTGAAGATCAGTTGGGTGCACGAGTGGGTTACATCGAACTGGATC<br>TCAACAGCGGTAAGATCCTTGAGAGTTTTCGCCCCGAAGAACGTTTTCCAATGATGAGCACTTTTAAAGTTCTGCTATGTGGCGCGGT<br>ATTATCCCGTATTGACGCCGGGCAAGAGCAACTCGGTCGCCGCATACACTATTCTCAGAATGACTTGGTTGAGTACTCACCAGTCACA<br>GAAAAGCATCTTACGGATGGCATGACAGTAAGAGAATTATGCAGTGCTGCCATAACCATGAGTGATAACACTGCGGCCAACTTACTTC<br>TGACAACGATCGGAGGACCGAAGGAGCTAACCGCTTTTTTGCACAACATGGGGGATCATGTAACTCGCCTTGATCGTTGGGAACCGG<br>AGCTGAATGAAGCCATACCAAACGACGAGCGTGACACCACGATGCCTGTAGCAATGGCAACAACGTTGCGCAAACTATTAACTGGCG<br>AACTACTTACTCTAGCTTCCCGGCAACAATTAATAGACTGGATGGAGGCGGATAAAGTTGCAGGACCACTTCTGCGCTCGGCCCTTCC<br>GGCTGGCTGGTTTATTGCTGATAAATCTGGAGCCGGTGAGCGTGGGTCTCGCGGTATCATTGCAGCACTGGGGCCAGATGGTAAGC<br>CCTCCCGTATCGTAGTTATCTACACGACGGGGAGTCAGGCAACTATGGATGAACGAAATAGACAGATCGCTGAGATAGGTGCCTCAC<br>TGATTAAGCATTGGTAAAATTATTAACGCTTACAATTTCCTGATGCGGTATTTTCTCCTTACGCATCTGTGCGGTATTTCACACCGCATC<br>AGGTGGCACTTTTCGGGGAAATGTGCGCGGAACCCCTATTTGTTTATTTTTCTAAATACATTCAAATATGTATCCGCTGGATCTCATGC<br>TGGAGTTCTTCGCCCACCCTTAATTAACCGTAGAAAAGATCAAAGGATCTTCTTGAGATCCTTTTTTTCTGCGCGTAATCTGCTGCTTG<br>CAAACAAAA	
AVA_TetR_SoxS_a	AAACCACCGCTACCAGCGGTGGTTTGTTTGCCGGATCAAGAGCTACCAACTCTTTTTCCGAAGGTAACTGGCTTCAGCAGAGCGCAG<br>ATACCAAATACTGTTCTTCTAGTGTAGCCGTAGTTAGGCCACCACTTCAAGAACTCTGTAGCACCGCCTACATACCTCGCTCTGCTAAT<br>CCTGTTACCAGTGGCTGCTGCCAGTGGCGATAAGTCGTGTCTTACCGGGTTGGACTCAAGACGATAGTTACCGGATAAGGCGCAGCG<br>GTCGGGCTGAACGGGGGGTTCGTGCACACAGCCCAGCTTGGAGCGAACGACCTACACCGAACTGAGATACCTACAGCGTGAGCTAT<br>GAGAAAGCGCCACGCTTCCCGAAGGGAGAAAGGCGGACAGGTATCCGGTAAGCGGCAGGGTCGGAACAGGAGAGCGCACGAGGG<br>AGCTTCCAGGGGGAAACGCCTGGTATCTTTATAGTCCTGTCGGGTTTCGCCACCTCTGACTTGAGCGTCGATTTTTGTGATGCTCGTC<br>AGGGGGGCGGAGCCTATGGAAAAACGCCAGCAACGCGGCCTTTTTACGGTTCCTGGCCTTTTGCTGGCCTTTTGCTCATGATATAATT<br>AAATTGAAGCTCTAATTTGTGAGTTTAGTATACATGCATTTACTTATAATACAGTTTTTTAGTTTTGCTGGCCGCATCTTCTCAAATATGC<br>TTCCCAGCCTGCTTTTCTGTAACGTTCACCCTCTACCTTAGCATCCCTTCCCTTTGCAAATAGTCCTCTTCCAACAATAATAATGTCAGA<br>TCCTGTAGAGACCACATCATCCACGGTTCTATACTGTTGACCCAATGCGTCTCCCTTGTCATCTAAACCCACACCGGGTGTCATAATCA<br>ACCAATCGTAACCTTCATCTCTTCCACCCATGTCTCTTTGAGCAATAAAGCCGATAACAAAATCTTTGTCGCTCTTCGCAATGTCAACA<br>GTACCCTTAGTATATTCTCCAGTAGATAGGGAGCCCTTGCATGACAATTCTGCTAACATCAAAAGGCCTCTAGGTTCCTTTGTTACTTC<br>TTCTGCCGCCTGCTTCAAACCGCTAACAATACCTGGGCCCACCACACCGTGTGCATTCGTAATGTCTGCCCATTCTGCTATTCTGTATA<br>CACCCGCAGAGTACTGCAATTTGACTGTATTACCAATGTCAGCAAATTTTCTGTCTTCGAAGAGTAAAAAATTGTACTTGGCGGATAAT<br>GCCTTTAGCGGCTTAACTGTGCCCTCCATGGAAAAATCAGTCAAGATATCCACATGTGTTTTTAGTAAACAAATTTTGGGACCTAATGC	This study

	TTCAACTAACTCCAGTAATTCCTTGGTGGTACGAACATCCAATGAAGCACACAAGTTTGTTTGCTTTTCGTGCATGATATTAAATAGCTT<br>GGCAGCAACAGGACTAGGATGAGTAGCAGCACGTTCCTTATATGTAGCTTTCGACATGATTTATCTTCGTTTCCTGAGGTTTTTGTTCT<br>GTGCAGTTGGGTTAAGAATACTGGGCAATTTCATGTTTCTTCAACACTACATATGCGTATATATACCAATCTAAGTCTGTGCTCCTTCCT<br>TCGTTCTTCCTTCTGTTCGGAGATTACCGAATCAAAAAAATTTCAAAGAAACCGAAATCAAAAAAAAGAATAAAAAAAAAATGATGAATT<br>GAATTGAAAAGCTAGCTTATCGATGATAAGCTGTCAAAGATGAGAATTAATTCCACGGACTATAGACTATACTAGATACTCCGTCTACT<br>GTACGATACACTTCCGCTCAGGTCCTTGTCCTTTAACGAGGCCTTACCACTCTTTTGTTACTCTATTGATCCAGCTCAGCAAAGGCAGT<br>GTGATCTAAGATTCTATCTTCGCGATGTAGTAAAACTAGCTAGACCGAGAAAGAGACTAGAAATGCAAAAGGCACTTCTACAATGGCT<br>GCCATCATTATTATCCGATGTGACGCTGCAGCTTCTCAATGATATTCGAATACGCTTTGAGGAGATACAGCCTAATATCCGACAAACTG<br>TTTTACAGATTTACGATCGTACTTGTTACCCATCATTGAATTTTGAACATCCGAACCTGGGAGTTTTCCCTGAAACAGATAGTATATTTG<br>AACCTGTATAATAATATATAGTCTAGCGCTTTACGGAAGACAATGTATGTATTTCGGTTCCTGGAGAAACTATTGCATCTATTGCATAGG<br>TAATCTTGCACGTCGCATCCCCGGTTCATTTTCTGCGTTTCCATCTTGCACTTCAATAGCATATCTTTGTTAACGAAGCATCTGTGCTTC<br>ATTTTGTAGAACAAAAATGCAACGCGAGAGCGCTAATTTTTCAAACAAAGAATCTGAGCTGCATTTTTACAGAACAGAAATGCAACGCG<br>AAAGCGCTATTTTACCAACGAAGAATCTGTGCTTCATTTTTGTAAAACAAAAATGCAACGCGACGAGAGCGCTAATTTTTCAAACAAAG<br>AATCTGAGCTGCATTTTTACAGAACAGAAATGCAACGCGAGAGCGCTATTTTACCAACAAAGAATCTATACTTCTTTTTTGTTCTACAAA<br>AATGCATCCCGAGAGCGCTATTTTTCTAACAAAGCATCTTAGATTACTTTTTTTCTCCTTTGTGCGCTCTATAATGCAGTCTCTTGATAA<br>CTTTTTGCACTGTAGGTCCGTTAAGGTTAGAAGAAGGCTACTTTGGTGTCTATTTTCTCTTCCATAAAAAAAGCCTGACTCCACTTCCC<br>GCGTTTACTGATTACTAGCGAAGCTGCGGGTGCATTTTTTCAAGATAAAGGCATCCCCGATTATATTCTATACCGATGTGGATTGCGCA<br>TACTTTGTGAACAGAAAGTGATAGCGTTGATGATTCTTCATTGGTCAGAAAATTATGAACGGTTTCTTCTATTTTGTCTCTATATACTAC<br>GTATAGGAAATGTTTACATTTTCGTATTGTTTTCGATTCACTCTATGAATAGTTCTTACTACAATTTTTTTGTCTAAAGAGTAATACTAGA<br>GATAAACATAAAAAATGTAGAGGTCGAGTTTAGATGCAAGTTCAAGGAGCGAAAGGTGGATGGGTAGGTTATATAGGGATATAGCACA<br>GAGATATATAGCAAAGAGATACTTTTGAGCAATGTTTGTGGAAGCGGTATTCGCAATGGGAAGCTCCACCCCGGTTGATAATCAGAAA<br>AGCCCCAAAAACAGGAAGATTGTATAAGCAAATATTTAAATTGTAATTCTTTCCTGCGTTATCCCCTGATTCTGTGGATAACCGTATTAC<br>CGCCTTTGAGTGAGCTGATACCGCTCGCCGCAGCCGAACGACCGAGCGCAGCGAGTCAGTGAGCGAGGAAGCGGAAGAGGGTCTG<br>ACGCTCAGTGGAACGAAAACTCACGTTAAGGGATTTTGGTCATGAGATTATCAAAAAGGATCTTCACCTAGATCCTTTTAAATTAAAAAT<br>GAAGTTTTAAATCAATCTAAAGTATATATGAGTAAACTTGGTCTGACAGTCAGAAGAACTCGTCAAGAAGGCGATAGAAGGCGATGCG<br>CTGCGAATCGGGAGCGGCGATACCGTAAAGCACGAGGAAGCGGTCAGCCCATTCGCCGCCAAGCTCTTCAGCAATATCACGGGTAG<br>CCAACGCTATGTCCTGATAGCGGTCCGCCACACCCAGCCGGCCACAGTCGATGAATCCAGAAAAGCGGCCATTTTCCACCATGATAT<br>TCGGCAAGCAGGCATCGTCATGGGTCACGACGAGATCCTCGCCGTCGGGCATGCTCGCCTTGAGCCTGGCGAACAGTTCGGCTGGC<br>GCGAGCCCCTGATGCTCTTCGTCCAGATCATCCTGATCGACAAGACCGGCTTCCATCCGAGTACGTGCTCGCTCGATGCGATGTTTC<br>GCTTGGTGGTCGAATGGGCAGGTAGCCGGATCAAGCGTATGCAGCCGCCGCATTGCATCAGCCATGATGGATACTTTCTCGGCAGG<br>AGCAAGGTGAGATGACAGGAGATCCTGCCCCGGCACTTCGCCCAATAGCAGCCAGTCCCTTCCCGCTTCAGTGACAACGTCGAGCA<br>CAGCTGCGCAAGGAACGCCCGTCGTGGCCAGCCACGATAGCCGCGCTGCCTCGTCTTGCAGTTCATTCAGGGCACCGGACAGGTCG<br>GTCTTGACAAAAAGAACCGGGCGCCCCTGCGCTGACAGCCGGAACACGGCGGCATCAGAGCAGCCGATTGTCTGTTGTGCCCAGTC<br>ATAGCCGAATAGCCTCTCCACCCAAGCGGCCGGAGAACCTGCGTGCAATCCATCTTGTTCAATCATACTCTTCCTTTTTCAATATTATT<br>GAAGCATTTATCAGGGTTATTGTCTCATGAGCGGATACATATTTGAATGTATTTAGAAAAATAAACAAATAGGGGTTCCGCGCACATTT<br>CCCCGAAAAGTGCCACCTGCGGACGGATCGCTTGCCTGTAACTTACACGCGCCTCGTCCTGAGAAAGCAACCTGACCTACAGGAAAG<br>AGTTACTCAAGAATAAGAATTTTCGTTTTAAAACCTAAGAGTCACTTTAAAATTTGTATACACTTATTTTTTTTATAACTTATTTAATAATAA<br>AAATCATAAATCATAAGAAATTCGCTTATTTAGAAGTGTCAACAACGTATCTACCAACGATTTGACCCTTTTCCATCTTTTCGTAAATTTC<br>TGGCAAGGTAGACAAGCCGACAACCTTGATTGGAGACTTGACCAAACCTCTGGCGAAGAATTGTTAATATTAAGAGCTCTCACTGCCC<br>ACTTTCCAGTCTGGAAACCTGTCTTGCTAGCTGCATGAGTGAATCAGCCAATGCCCGGGGAGAGGCAGTTTGTGTATTGGGTGCCAG<br>GGTGGTTTTTCTCTTCACCAGTGAGACTGGCAACAGCTGATTGCCCTTCACCGCCTGGCCCTGAGAGAGTTGCAGCAAGCGGTCCAC<br>GCTGGTTTGCCCCAGCAGGCGAAAATCCTGTTTGATGGTGGTTAACGGCGGGATATAACATGAGCTGTCTTCGGTATCGTCGTATCC<br>CACTACCGAGATATCTGCACCAACTCTCAGCCCAGACTCAGTAATGGCTCTCATTGCACCCAGTGCCATCTGATCATTGGCAACCAGC<br>ATTGCAGTGGGAACAATGCCCTCATTCAGCATTTGCATGGTTTGTTGAAACCCAGACATGGCACTCCAGTCCCCTTCTCTTTCAGCTAT<br>TGGCTGAATTTGATTCCTAGTGAGATATTTATGCCAGCCGGCCAGTCTCAGCCTTGCTGAGACAGAACTGAGTGGGCCCGCAAGCAG<br>TGCAATTTGCTGGTGTCCCAATGCAACCAGATGCTCCACACCCAGTCTTGTACCATCTTCATGGGAGAAAATAATACTGTTGATGGGT<br>GTCTGGTCAGAGACATCAAGAAAGAGTGCTGGAACATTAGTGCAGGCAGCTTCCACAGCAATGGCATCCTGGTCATCCAGTGGATAG<br>TTAATGATCAGCCCACTGACTCTTTGTGCCAGAAGATTGTGCACAGCAGCTTTACAGGCTTCAACTCCACTTCTTTCTACCATTGACAC<br>CACCACAGAGGCTCCCAGTTGATCAGCTCTAGATTTAATGGCTGCCACAATTTGAGATGGTGCATGCAGGGCCAGACTGGAGGTGGC<br>AACTCCAATCAGCAAGCTCTGTTTGCCTGCCAGTTGTTGTGCCACTCTGTTGGGAATGTAATTCAGCTCTGCCATGGCTGCTTCCACTT<br>TTTCCCTGGTTTTGGCAGAAACATGGCTGGCCTGGTTCACCACTCTGGAAACAGTCTGATAAGAGACACCGGCATACTCTGCGACATC<br>ATACAATGTTACTGGTTTCATTTTAATAACTAGTAAAACTTAGATTAGATTGCTATGCTTTCTTTCTAATGAGCAAGAAGTAAAAAAAGTT<br>GTAATAGAACAAGAAAAATGAAACTGAAACTTGAGAAATTGAAGACCGTTTATTAACTTAAATATCAATGGGAGGTCATCGAAAGAGAA<br>AAAAATCAAAAAAAAAATTTTCAAGAAAAAGAAACGTGATAAAAATTTTTATTGCCTTTTTCGACGAAGAAAAAGAAACGAGGCGGTCTC<br>TTTTTTCTTTTCCAAACCTTTAGTACGGGTAATTAACGACACCCTAGAGGAAGAAAGAGGGGAAATTTAGTATGCTGTGCTTGGGTGTT<br>TTGAAGTGGTACGGCGATGCGCGGAGTCCGAGAAAATCTGGAAGAGTAAAAAAGGAGTAGAAACATTTTGAAGCTATGGTGTTCGAG<br>TTCAAGAGAAAAAAAAAGAAAAAGCAAAAAGAAAAAAGGAAAGCGCGCCTCGTTCAGAATGACACGTATAGAATGATGCATTACCTTGT	

	CATCTTCAGTATCATACTGTTCGTATACATACTTACTGACATTCATAGGTATACATATATACACATGTATATATATCGTATGCTGCAGCTT<br>TAAATAATCGGTGTCACTACATAAGAACACCTTTGGTGGAGGGAACATCGTTGGTACCATTGGGCGAGGTGGCTTCTCTTATGGCAAC<br>CGCAAGAGCCTTGAACGCACTCTCACTACGGTGATGATCATTCTTGCCTCGCAGACAATCAACGTGGAGGGTAATTCTGCTAGCCTCT<br>GCAAAGCTTTCAAGAAAATGCGGGATCATCTCGCAAGAGAGATCTCCTACTTTCTCCCTTTGCAAACCAAGTTCGACAACTGCGTACG<br>GCCTGTTCGAAAGATCTACCACCGCTCTGGAAAGTGCCTCATCCAAAGGCGCAAATCCTGATCCAAACCTTTTTACTCCACGCGCCAG<br>TAGGGCCTCTTTAAAAGCTTGACCGAGAGCAATCCCGCAGTCTTCAGTGGTGTGATGGTCGTCTATGTGTAAGTCACCAATGCACTCA<br>ACGATTAGCGACCAGCCGGAATGCTTGGCCAGAGCATGTATCATATGGTCCAGAAACCCTATACCTGTGTGGACGTTAATCACTTGCG<br>ATTGTGTGGCCTGTTCTGCTACTGCTTCTGCCTCTTTTTCTGGGAAGATCGAGTGCTCTATCGCTAGGGGACCACCCTTTAAAGAGAT<br>CGCAATCTGAATCTTGGTTTCATTTGTAATACGCTTTACTAGGGCTTTCTGCTCTGTCATCGCGGCCGCCCAAGCTGCCTTTGTGTGCT<br>TAATCACGTCTTTGCCTTCGTTTATCTTGCCTGCTCATTTTTTAGTATATTCTTCGAAGAAATCACATTACTTTATATAATGTATAATTCAT<br>TATGTGATAATGCCAATCGCTAAGAAAAAAAAAGAGTCATCCGCTAGGGGAAAAAAAAAAATGAAAATCATTACCGAGGCATAAAAAAA<br>TATAGAGTGTACTAGAGGAGGCCAAGAGTAATAGAAAAAGAAAATTGCGGGAAAGGACTGTGTTATGACTTCCCTGACTAATGCCGTG<br>TTCAAACGATACCTGGCAGTGACTCCTAGCGCTCACCAAGCTCTTAAAACGGGACTCAGTACAATCTGCTCTGATGCCGCATAGTTAA<br>GCTTCTCTATCACTGATAGGGAGTGGTAAAATAACTCTATCAATGATAGAGTGTCAACAAAAATTAGGAATTAATGATGTCTAGATTAGA<br>TAAAAGTAAAGTGATTAACAGCGCATTAGAGCTGCTTAATGAGGTCGGAATCGAAGGTTTAACAACCCGTAAACTCGCCCAGAAGCTA<br>GGTGTAGAGCAGCCTACATTGTATTGGCATGTAAAAAATAAGCGGGCTTTGCTCGACGCCTTAGCCATTGAGATGTTAGATAGGCACC<br>ATACTCACTTTTGCCCTTTAGAAGGGGAAAGCTGGCAAGATTTTTTACGTAATAACGCTAAAAGTTTTAGATGTGCTTTACTAAGTCATC<br>GCGATGGAGCAAAAGTACATTTAGGTACACGGCCTACAGAAAAACAGTATGAAACTCTCGAAAATCAATTAGCCTTTTTATGCCAACAA<br>GGTTTTTCACTAGAGAATGCATTATATGCACTCAGCGCTGTGGGGCATTTTACTTTAGGTTGCGTATTGGAAGATCAAGAGCATCAAGT<br>CGCTAAAGAAGAAAGGGAAACACCTACTACTGATAGTATGCCGCCATTATTACGACAAGCTATCGAATTATTTGATCACCAAGGTGCA<br>GAGCCAGCCTTCTTATTCGGCCTTGAATTGATCATATGCGGATTAGAAAAACAACTTAAATGTGAAAGTGGGTCTTAAAAGCAGCATAA<br>CCTTTTTCCGTGATGGTAACTTCACGGTAACCAAGATGTCGAGTTAACCACCCTTTAGATTCATAAAGCGAAAATAATGCGGCTCCAAC<br>GTACCCACCTAAATGGAAACGGCGTTCACTCCAAGCGAAAAAACCCCGCCGAAGCGGGGTTTTTTGCGTTAAAGCCGGTGACGGTAG<br>TCGCTGGGAGTCCGGTCAAATTGACGAGCGAAGACGCGAGAGAAGGTTTGCTGCGACACATAGCCTAAGTCCATGGCAATATCGAAA<br>ATCGGACGTTCTGTTGTCCGCAATTCAACGGCAGCCAACAACAAACGGCGTTGACGGATATAGTCTCCTAATGTTTGATGTGTGACGG<br>TACGAAACATGCGCTGTAAATACCACTTCGAGTAACCACTTTTCTTCGCTACAACATCAATATTAAGAGGCTGATCAATATGCTCATCG<br>ATCCAAGCAATAAGGTCCTGGATGATTTTTTGGTGGCTCATTGAACCACCTCCGCCGTAAATACCACTGTTGGCCGCGATGGCAGATG<br>GAATCGGATTTCCATCTTTCAGAAGACCCTGCATCGCTTTCACAATCAACTCACAGTCGCTATTCGTAGCAAAGATCGGAATAGTCAGT<br>TCCATATTAAGGTACGAACGCCATGCACCTTTAGGGACTTCAACTTTGATCGTGTACTTACGGTTTTGCGCACTCGATTGACGTACAGA<br>ACAAGTAACTTTGTATGCTTGGCTACGGCTGTTGGAGGAAATCCACTCGGCAATGCCGTTAGCGAAGTTGCTAGGGGCAACAGTTACA<br>TCACCAGTACCACCATTATCCACAAGAACGAACTGCGTAAAATTGCTCGCGGGACCCATAGATCCTTTCTCCTCTTTAGATCTTTTGAA<br>TTCCCAAAAAAACGGGTATGGAGAAACAGTAGAGAGTTGCGATAAAAAGCGTCAGGTAGAATCCGCTAATCTTATGGATAAAAATGCT<br>ATGGCATAGCAAAGTGTGACGCCGTGCAAATAATCAATGTGGACTTTTCTGCCGTGATTATAGACACTTTTGTTACGCGTTTTTGTCAT<br>GGCTTTGGTCCCGCTTTGTTACAGAATGCTTTTAATAAGCGGGGTTACCGGTTTGGTTAGCGAGAAGAGCCAGTAAAAGACGCAGTGA<br>CGGCAATGTCTGATGCAATATGGACAATTGGTTTCTTCTCTGAATGGCGGGAGTATGAAAAGTTCAACATTTCCGTGTCGCCCTTATTC<br>CCTTTTTTGCGGCATTTTGCCTTCCTGTTTTTGCTCACCCAGAAACGCTGGTGAAAGTAAAAGATGCTGAAGATCAGTTGGGTGCACG<br>AGTGGGTTACATCGAACTGGATCTCAACAGCGGTAAGATCCTTGAGAGTTTTCGCCCCGAAGAACGTTTTCCAATGATGAGCACTTTT<br>AAAGTTCTGCTATGTGGCGCGGTATTATCCCGTATTGACGCCGGGCAAGAGCAACTCGGTCGCCGCATACACTATTCTCAGAATGACT<br>TGGTTGAGTACTCACCAGTCACAGAAAAGCATCTTACGGATGGCATGACAGTAAGAGAATTATGCAGTGCTGCCATAACCATGAGTGA<br>TAACACTGCGGCCAACTTACTTCTGACAACGATCGGAGGACCGAAGGAGCTAACCGCTTTTTTGCACAACATGGGGGATCATGTAACT<br>CGCCTTGATCGTTGGGAACCGGAGCTGAATGAAGCCATACCAAACGACGAGCGTGACACCACGATGCCTGTAGCAATGGCAACAAC<br>GTTGCGCAAACTATTAACTGGCGAACTACTTACTCTAGCTTCCCGGCAACAATTAATAGACTGGATGGAGGCGGATAAAGTTGCAGGA<br>CCACTTCTGCGCTCGGCCCTTCCGGCTGGCTGGTTTATTGCTGATAAATCTGGAGCCGGTGAGCGTGGGTCTCGCGGTATCATTGCA<br>GCACTGGGGCCAGATGGTAAGCCCTCCCGTATCGTAGTTATCTACACGACGGGGAGTCAGGCAACTATGGATGAACGAAATAGACAG<br>ATCGCTGAGATAGGTGCCTCACTGATTAAGCATTGGTAAAATTATTAACGCTTACAATTTCCTGATGCGGTATTTTCTCCTTACGCATCT<br>GTGCGGTATTTCACACCGCATCAGGTGGCACTTTTCGGGGAAATGTGCGCGGAACCCCTATTTGTTTATTTTTCTAAATACATTCAAAT<br>ATGTATCCGCTGGATCTCATGCTGGAGTTCTTCGCCCACCCTTAATTAACCGTAGAAAAGATCAAAGGATCTTCTTGAGATCCTTTTTTT<br>CTGCGCGTAATCTGCTGCTTGCAAACAAAA	
pSJ105	GAGATACCTACAGCGTGAGCTATGAGAAAGCGCCACGCTTCCCGAAGGGAGAAAGGCGGACAGGTATCCGGTAAGCGGCAGGGTC<br>GGAACAGGAGAGCGCACGAGGGAGCTTCCAGGGGGAAACGCCTGGTATCTTTATAGTCCTGTCGGGTTTCGCCACCTCTGACTTGA<br>GCGTCGATTTTTGTGATGCTCGTCAGGGGGGCGGAGCCTATGGAAAAACGCCAGCAACGCGGCCTTTTTACGGTTCCTGGCCTTTTG<br>CTGGCCTTTTGCTCTCAGATAAAATATTTCTAGATTTCAGTGCAATTTATCTCTTCAAATGTAGCACCTGAAGTCAGCCCCATACGATAT<br>AAGTTGTTACTAGATTGACAGCTAGCTCAGTCCTAGGTATAATACTAGTCGGTTACCAAAGGCGTCCTTGTTTTAGAGCTAGAAATAGC<br>AAGTTAAAATAAGGCTAGTCCGTTATCAACTTGAAAAAGTGGCACATGAGGATCACCCATGTGCTTTTTTTGAAGCTTGGGCCCGAACA<br>AAAACTCATCTCAGAAGAGGATCTGAATAGCGCCGTCGACCATCATCATCATCATCATTGAGTTTAAACGGTCTCCAGCTTGGCTGTTT<br>TGGCGGATGAGAGAAGATTTTCAGCCTGATACAGATTAAATCAGAACGCAGAAGCGGTCTGATAAAACAGAATTTGCCTGGCGGCAGT<br>AGCGCGGTGGTCCCACCTGACCCCATGCCGAACTCAGAAGTGAAACGCCGTAGCGCCGATGGTAGTGTGGGGTCTCCCCATGCGAG	This study

	AGTAGGGAACTGCCAGGCATCAAATAAAACGAAAGGCTCAGTCGAAAGACTGGGCCTTTCGTTTTATCTGTTGTTTGTCGGTGAACTG<br>GATCCTTACTCGAGTCTAGACTGCAGGCTTCCTCGCTCACTGACTCGCTGCGCTCGGTCGTTCGGCTGCGGCGAGCGGTATCAGCTC<br>ACTCAAAGGCGGTAATACGGTTATCCACAGAATCAGGGGATAACGCAGGAAAGAACATGTGAGCAAAAGGCCAGCAAAAGGCCAGGA<br>ACCGTAAAAAACTAGTGCTTGGATTCTCACCAATAAAAAACGCCCGGCGGCAACCGAGCGTTCTGAACAAATCCAGATGGAGTTCTGA<br>GGTCATTACTGGATCTATCAACAGGAGTCCAAGCGAGCTCGATATCAAATTACGCCCCGCCCTGCCACTCATCGCAGTACTGTTGTAA<br>TTCATTAAGCATTCTGCCGACATGGAAGCCATCACAAACGGCATGATGAACCTGAATCGCCAGCCGAGACGAAAGGGCCTCGTGATA<br>CGCCTATTTTTATAGGTTAATGTCATGATAATAATGGTTTCTTACCTCCCGTGGGGAAAAAATCATGGCAATTCTGGAAGAAATAGCGC<br>TTTCAGCCGGCAAACCGGCTGAAGCCGGATCTGCGATTCTGATAACAAACTAGCAACACCAGAACAGCCCGTTTGCGGGCAGCAAAA<br>CCCGTACCCTAGGTATAAACGCAGAAAGGCCCACCCGAAGGTGAGCCAGTGTGACTCTAGTAGAGAGCGTTCACCGACAAACAACAG<br>ATAAAACGAAAGGCCCAGTCTTTCGACTGAGCCTTTCGTTTTATTTGATGCCTGGAGATCCTTACTCGAGTTTGGATCCTTAAGCACCG<br>GTGGAGTGACGACCTTCAGCACGTTCGTACTGTTCAACGATGGTGTAGTCTTCGTTGTGGGAGGTGATGTCCAGTTTGATGTCGGTTT<br>TGTAAGCACCCGGCAGCTGAACCGGTTTTTTAGCCATGTAGGTGGTTTTAACTTCAGCGTCGTAGTGACCACCGTCTTTCAGTTTCAG<br>ACGCATTTTGATTTCACCTTTCAGAGCACCGTCTTCCGGGTACATACGTTCGGTGGAAGCTTCCCAACCCATGGTTTTTTTCTGCATAA<br>CCGGACCGTCGGACGGGAAGTTGGTACCACGCAGTTTAACTTTGTAGATGAACTCACCGTCTTGCAGGGAGGAGTCCTGGGTAACG<br>GTAACAACACCACCGTCTTCGAAGTTCATAACACGTTCCCATTTGAAACCTTCCGGGAAGGACAGTTTCAGGTAGTCCGGGATGTCAG<br>CCGGGTGTTTAACGTAAGCTTTGGAACCGTACTGGAACTGCGGGGACAGGATGTCCCAAGCGAACGGCAGCGGACCACCTTTGGTA<br>ACTTTCAGTTTAGCGGTCTGGGTACCTTCGTACGGACGACCTTCACCTTCACCTTCGATTTCGAACTCGTGACCGTTAACGGAACCTT<br>CCATACGAACTTTGAAACGCATGAACTCTTTGATAACGTCTTCGCTACTCGCCATGGTACCTTTCTCCTCTTTAATGAATTCGCTAGCA<br>CAATCCCTAGGACTGAGCTAGCTGTCAAGTCCGGAGGTATCCGGTGGAACCCAAAGGACGCCTTTGGTAACCGCAGGACACCGCAG<br>GATACCTGAGGTCGCCCGTGGTGGCCCATGGTCACCATAGGTCACCCTTGGCAACCAAAGGTGTCCTATGGCGGCCGGAGGCTGCC<br>ATAGGTCTCCGGTGGATACCGTAGGCGACGTCATCTAGATGCATTCGCGAGGTACCGAGCTCGAATTCACTGGCCGTCGTTTTACAA<br>CGTCGTGACTGGGAAAACCCTGGCGTTACCCAACTTAATCGCCTTGCAGCACATCCCCCTTTCGCCAGCTGGCGTAATAGCGAAGAG<br>GCCCGCACCGATCGCCCTTCCCAACAGTTGCGCAGCCTGAATGGCGAATGGCGCCTGATGCGGTATTTTCTCCTTACGCATCTGTGC<br>GGTATTTCACACCGCATATGGTGCACTCTCAGTACAATCTGCTCTGATGCCGCATAGTTAAGCCAGCCCCGACACCCGCCAACACCC<br>GCTGACGCGCCCTGACGGGCTTGTCTGCTCCCGGCATCCGCTTACAGACAAGCTGTGACCGTCTCCGGGAGCTGCATGTGTCAGAG<br>GTTTTCACCGTCATCACCGAAACGCGCGAGACGAAAGGGCCTCGTGATACGCCTATTTTTATAGGTTAATGTCATGATAATAATGGTTT<br>CTTAGACGTCAGGTGGCACTTTTCGGGGAAATGTGCGCGGAACCCCTATTTGTTTATTTTTCTAAATACATTCAAATATGTATCCGCTC<br>ATGAGACAATAACCCTGATAAATGCTTCAATAATATTGAAAAAGGAAGAGTATGAGTATTCAACATTTCCGTGTCGCCCTTATTCCCTTT<br>TTTGCGGCATTTTGCCTTCCTGTTTTTGCTCACCCAGAAACGCTGGTGAAAGTAAAAGATGCTGAAGATCAGTTGGGTGCACGAGTGG<br>GTTACATCGAACTGGATCTCAACAGCGGTAAGATCCTTGAGAGTTTTCGCCCCGAAGAACGTTTTCCAATGATGAGCACTTTTAAAGTT<br>CTGCTATGTGGCGCGGTATTATCCCGTATTGACGCCGGGCAAGAGCAACTCGGTCGCCGCATACACTATTCTCAGAATGACTTGGTT<br>GAGTACTCACCAGTCACAGAAAAGCATCTTACGGATGGCATGACAGTAAGAGAATTATGCAGTGCTGCCATAACCATGAGTGATAACA<br>CTGCGGCCAACTTACTTCTGACAACGATCGGAGGACCGAAGGAGCTAACCGCTTTTTTGCACAACATGGGGGATCATGTAACTCGCC<br>TTGATCGTTGGGAACCGGAGCTGAATGAAGCCATACCAAACGACGAGCGTGACACCACGATGCCTGTAGCAATGGCAACAACGTTGC<br>GCAAACTATTAACTGGCGAACTACTTACTCTAGCTTCCCGGCAACAATTAATAGACTGGATGGAGGCGGATAAAGTTGCAGGACCACT<br>TCTGCGCTCGGCCCTTCCGGCTGGCTGGTTTATTGCTGATAAATCTGGAGCCGGTGAGCGTGGGTCTCGCGGTATCATTGCAGCACT<br>GGGGCCAGATGGTAAGCCCTCCCGTATCGTAGTTATCTACACGACGGGGAGTCAGGCAACTATGGATGAACGAAATAGACAGATCGC<br>TGAGATAGGTGCCTCACTGATTAAGCATTGGTAACTGTCAGACCAAGTTTACTCATATATACTTTAGATTGATTTAAAACTTCATTTTTAA<br>TTTAAAAGGATCTAGGTGAAGATCCTTTTTGATAATCTCATGACCAAAATCCCTTAACGTGAGTTTTCGTTCCACTGAGCGTCAGACCC<br>CGTAGAAAAGATCAAAGGATCTTCAAGCAGCATAACCTTTTTCCGTGATGGTAACTTCACGGTAACCAAGATGTCGAGTTAACCACCCT<br>TTAGATTCATAAAGCGAAAATAATGCGGCTCCAACGTACCCACCTAAATGGAAACGGCGTTCACTCCAAGCGAAAAAACCCCGCCGAA<br>GCGGGGTTTTTTGCGTTAAAGCCGGTGACGGTAGTCGCTGGGAGTCCGGTCAAATTGACGAGCGAAGACGCGAGAGAAGGTTTGCT<br>GCGACACATAGCCTAAGTCCATGGCAATATCGAAAATCGGACGTTCTGTTGTCCGCAATTCAACGGCAGCCAACAACAAACGGCGTT<br>GACGGATATAGTCTCCTAATGTTTGATGTGTGACGGTACGAAACATGCGCTGTAAATACCACTTCGAGTAACCACTTTTCTTCGCTACA<br>ACATCAATATTAAGAGGCTGATCAATATGCTCATCGATCCAAGCAATAAGGTCCTGGATGATTTTTTGGTGGCTCATTGAACCACCTCC<br>GCCGTAAATACCACTGTTGGCCGCGATGGCAGATGGAATCGGATTTCCATCTTTCAGAAGACCCTGCATCGCTTTCACAATCAACTCA<br>CAGTCGCTATTCGTAGCAAAGATCGGAATAGTCAGTTCCATATTAAGGTACGAACGCCATGCACCTTTAGGGACTTCAACTTTGATCGT<br>GTACTTACGGTTTTGCGCACTCGATTGACGTACAGAACAAGTAACTTTGTATGCTTGGCTACGGCTGTTGGAGGAAATCCACTCGGCA<br>ATGCCGTTAGCGAAGTTGCTAGGGGCAACAGTTACATCACCAGTACCACCATTATCCACAAGAACGAACTGCGTAAAATTGCTCGCGG<br>GACCCATAGATCCTTTCTCCTCTTTAGATCTTTTGAATTCCCAAAAAAACGGGTATGGAGAAACAGTAGAGAGTTGCGATAAAAAGCGT<br>CAGGTAGAATCCGCTAATCTTATGGATAAAAATGCTATGGCATAGCAAAGTGTGACGCCGTGCAAATAATCAATGTGGACTTTTCTGCC<br>GTGATTATAGACACTTTTGTTACGCGTTTTTGTCATGGCTTTGGTCCCGCTTTGTTACAGAATGCTTTTAATAAGCGGGGTTACCGGTTT<br>GGTTAGCGAGAAGAGCCAGTAAAAGACGCAGTGACGGCAATGTCTGATGCAATATGGACAATTGGTTTCTTCTCTGAATGGCGGGAG<br>TATGAAAAGTTGAGATCCTTTTTTTCTGCGCGTAATCTGCTGCTTGCAAACAAAAAAACCACCGCTACCAGCGGTGGTTTGTTTGCCGG<br>ATCAAGAGCTACCAACTCTTTTTCCGAAGGTAACTGGCTTCAGCAGAGCGCAGATACCAAATACTGTTCTTCTAGTGTAGCCGTAGTTA<br>GGCCACCACTTCAAGAACTCTGTAGCACCGCCTACATACCTCGCTCTGCTAATCCTGTTACCAGTGGCTGCTGCCAGTGGCGATAAGT	

	CGTGTCTTACCGGGTTGGACTCAAGACGATAGTTACCGGATAAGGCGCAGCGGTCGGGCTGAACGGGGGGTTCGTGCACACAGCCC<br>AGCTTGGAGCGAACGACCTACACCGAACT	
pS106	GAGATACCTACAGCGTGAGCTATGAGAAAGCGCCACGCTTCCCGAAGGGAGAAAGGCGGACAGGTATCCGGTAAGCGGCAGGGTC<br>GGAACAGGAGAGCGCACGAGGGAGCTTCCAGGGGGAAACGCCTGGTATCTTTATAGTCCTGTCGGGTTTCGCCACCTCTGACTTGA<br>GCGTCGATTTTTGTGATGCTCGTCAGGGGGGCGGAGCCTATGGAAAAACGCCAGCAACGCGGCCTTTTTACGGTTCCTGGCCTTTTG<br>CTGGCCTTTTGCTCTCAGATAAAATATTTCTAGATTTCAGTGCAATTTATCTCTTCAAATGTAGCACCTGAAGTCAGCCCCATACGATAT<br>AAGTTGTTACTAGATTGACAGCTAGCTCAGTCCTAGGTATAATACTAGTAGGACGCCTTTGGTAACCGCGTTTTAGAGCTAGAAATAGC<br>AAGTTAAAATAAGGCTAGTCCGTTATCAACTTGAAAAAGTGGCACATGAGGATCACCCATGTGCTTTTTTTGAAGCTTGGGCCCGAACA<br>AAAACTCATCTCAGAAGAGGATCTGAATAGCGCCGTCGACCATCATCATCATCATCATTGAGTTTAAACGGTCTCCAGCTTGGCTGTTT<br>TGGCGGATGAGAGAAGATTTTCAGCCTGATACAGATTAAATCAGAACGCAGAAGCGGTCTGATAAAACAGAATTTGCCTGGCGGCAGT<br>AGCGCGGTGGTCCCACCTGACCCCATGCCGAACTCAGAAGTGAAACGCCGTAGCGCCGATGGTAGTGTGGGGTCTCCCCATGCGAG<br>AGTAGGGAACTGCCAGGCATCAAATAAAACGAAAGGCTCAGTCGAAAGACTGGGCCTTTCGTTTTATCTGTTGTTTGTCGGTGAACTG<br>GATCCTTACTCGAGTCTAGACTGCAGGCTTCCTCGCTCACTGACTCGCTGCGCTCGGTCGTTCGGCTGCGGCGAGCGGTATCAGCTC<br>ACTCAAAGGCGGTAATACGGTTATCCACAGAATCAGGGGATAACGCAGGAAAGAACATGTGAGCAAAAGGCCAGCAAAAGGCCAGGA<br>ACCGTAAAAAACTAGTGCTTGGATTCTCACCAATAAAAAACGCCCGGCGGCAACCGAGCGTTCTGAACAAATCCAGATGGAGTTCTGA<br>GGTCATTACTGGATCTATCAACAGGAGTCCAAGCGAGCTCGATATCAAATTACGCCCCGCCCTGCCACTCATCGCAGTACTGTTGTAA<br>TTCATTAAGCATTCTGCCGACATGGAAGCCATCACAAACGGCATGATGAACCTGAATCGCCAGCCGAGACGAAAGGGCCTCGTGATA<br>CGCCTATTTTTATAGGTTAATGTCATGATAATAATGGTTTCTTAGACGTCAGGTGGCACTTTTCGGGGAAATGTGCGCGGAACCCCTAT<br>TTGTTTATTTTTCTAAATACATTCAAATATGTATCCGCTCATGAGACAATAACCCTGATAAATGCTTCAATAATATTGAAAAAGGAAGAGT<br>ATGAGTATTCAACATTTCCGTGTCGCCCTTATTCCCTTTTTTGCGGCATTTTGCCTTCCTGTTTTTGCTCACCCAGAAACGCTGGTGAAA<br>GTAAAAGATGCTGAAGATCAGTTGGGTGCACGAGTGGGTTACATCGAACTGGATCTCAACAGCGGTAAGATCCTTGAGAGTTTTCGCC<br>CCGAAGAACGTTTTCCAATGATGAGCACTTTTAAAGTTCTGCTATGTGGCGCGGTATTATCCCGTATTGACGCCGGGCAAGAGCAACT<br>CGGTCGCCGCATACACTATTCTCAGAATGACTTGGTTGAGTACTCACCAGTCACAGAAAAGCATCTTACGGATGGCATGACAGTAAGA<br>GAATTATGCAGTGCTGCCATAACCATGAGTGATAACACTGCGGCCAACTTACTTCTGACAACGATCGGAGGACCGAAGGAGCTAACC<br>GCTTTTTTGCACAACATGGGGGATCATGTAACTCGCCTTGATCGTTGGGAACCGGAGCTGAATGAAGCCATACCAAACGACGAGCGT<br>GACACCACGATGCCTGTAGCAATGGCAACAACGTTGCGCAAACTATTAACTGGCGAACTACTTACTCTAGCTTCCCGGCAACAATTAA<br>TAGACTGGATGGAGGCGGATAAAGTTGCAGGACCACTTCTGCGCTCGGCCCTTCCGGCTGGCTGGTTTATTGCTGATAAATCTGGAG<br>CCGGTGAGCGTGGGTCTCGCGGTATCATTGCAGCACTGGGGCCAGATGGTAAGCCCTCCCGTATCGTAGTTATCTACACGACGGGG<br>AGTCAGGCAACTATGGATGAACGAAATAGACAGATCGCTGAGATAGGTGCCTCACTGATTAAGCATTGGTAACTGTCAGACCAAGTTT<br>ACTCATATATACTTTAGATTGATTTAAAACTTCATTTTTAATTTAAAAGGATCTAGGTGAAGATCCTTTTTGATAATCTCATGACCAAAATC<br>CCTTAACGTGAGTTTTCGTTCCACTGAGCGTCAGACCCCGTAGAAAAGATCAAAGGATCTTCAAGCAGCATAACCTTTTTCCGTGATG<br>GTAACTTCACGGTAACCAAGATGTCGAGTTAACCACCCTTTAGATTCATAAAGCGAAAATAATGCGGCTCCAACGTACCCACCTAAATG<br>GAAACGGCGTTCACTCCAAGCGAAAAAACCCCGCCGAAGCGGGGTTTTTTGCGTTAAAGCCGGTGACGGTAGTCGCTGGGAGTCCG<br>GTCAAATTGACGAGCGAAGACGCGAGAGAAGGTTTGCTGCGACACATAGCCTAAGTCCATGGCAATATCGAAAATCGGACGTTCTGT<br>TGTCCGCAATTCAACGGCAGCCAACAACAAACGGCGTTGACGGATATAGTCTCCTAATGTTTGATGTGTGACGGTACGAAACATGCGC<br>TGTAAATACCACTTCGAGTAACCACTTTTCTTCGCTACAACATCAATATTAAGAGGCTGATCAATATGCTCATCGATCCAAGCAATAAGG<br>TCCTGGATGATTTTTTGGTGGCTCATTGAACCACCTCCGCCGTAAATACCACTGTTGGCCGCGATGGCAGATGGAATCGGATTTCCAT<br>CTTTCAGAAGACCCTGCATCGCTTTCACAATCAACTCACAGTCGCTATTCGTAGCAAAGATCGGAATAGTCAGTTCCATATTAAGGTAC<br>GAACGCCATGCACCTTTAGGGACTTCAACTTTGATCGTGTACTTACGGTTTTGCGCACTCGATTGACGTACAGAACAAGTAACTTTGTA<br>TGCTTGGCTACGGCTGTTGGAGGAAATCCACTCGGCAATGCCGTTAGCGAAGTTGCTAGGGGCAACAGTTACATCACCAGTACCACC<br>ATTATCCACAAGAACGAACTGCGTAAAATTGCTCGCGGGACCCATAGATCCTTTCTCCTCTTTAGATCTTTTGAATTCCCAAAAAAACG<br>GGTATGGAGAAACAGTAGAGAGTTGCGATAAAAAGCGTCAGGTAGAATCCGCTAATCTTATGGATAAAAATGCTATGGCATAGCAAAG<br>TGTGACGCCGTGCAAATAATCAATGTGGACTTTTCTGCCGTGATTATAGACACTTTTGTTACGCGTTTTTGTCATGGCTTTGGTCCCGC<br>TTTGTTACAGAATGCTTTTAATAAGCGGGGTTACCGGTTTGGTTAGCGAGAAGAGCCAGTAAAAGACGCAGTGACGGCAATGTCTGAT<br>GCAATATGGACAATTGGTTTCTTCTCTGAATGGCGGGAGTATGAAAAGTTGAGATCCTTTTTTTCTGCGCGTAATCTGCTGCTTGCAAA<br>CAAAAAAACCACCGCTACCAGCGGTGGTTTGTTTGCCGGATCAAGAGCTACCAACTCTTTTTCCGAAGGTAACTGGCTTCAGCAGAGC<br>GCAGATACCAAATACTGTTCTTCTAGTGTAGCCGTAGTTAGGCCACCACTTCAAGAACTCTGTAGCACCGCCTACATACCTCGCTCTG<br>CTAATCCTGTTACCAGTGGCTGCTGCCAGTGGCGATAAGTCGTGTCTTACCGGGTTGGACTCAAGACGATAGTTACCGGATAAGGCG<br>CAGCGGTCGGGCTGAACGGGGGGTTCGTGCACACAGCCCAGCTTGGAGCGAACGACCTACACCGAACT	This study
pSJ106	GAGATACCTACAGCGTGAGCTATGAGAAAGCGCCACGCTTCCCGAAGGGAGAAAGGCGGACAGGTATCCGGTAAGCGGCAGGGTC<br>GGAACAGGAGAGCGCACGAGGGAGCTTCCAGGGGGAAACGCCTGGTATCTTTATAGTCCTGTCGGGTTTCGCCACCTCTGACTTGA<br>GCGTCGATTTTTGTGATGCTCGTCAGGGGGGCGGAGCCTATGGAAAAACGCCAGCAACGCGGCCTTTTTACGGTTCCTGGCCTTTTG<br>CTGGCCTTTTGCTCTCAGATAAAATATTTCTAGATTTCAGTGCAATTTATCTCTTCAAATGTAGCACCTGAAGTCAGCCCCATACGATAT<br>AAGTTGTTACTAGATTGACAGCTAGCTCAGTCCTAGGTATAATACTAGTAGGACGCCTTTGGTAACCGCGTTTTAGAGCTAGAAATAGC<br>AAGTTAAAATAAGGCTAGTCCGTTATCAACTTGAAAAAGTGGCACATGAGGATCACCCATGTGCTTTTTTTGAAGCTTGGGCCCGAACA<br>AAAACTCATCTCAGAAGAGGATCTGAATAGCGCCGTCGACCATCATCATCATCATCATTGAGTTTAAACGGTCTCCAGCTTGGCTGTTT<br>TGGCGGATGAGAGAAGATTTTCAGCCTGATACAGATTAAATCAGAACGCAGAAGCGGTCTGATAAAACAGAATTTGCCTGGCGGCAGT	This study

	AGCGCGGTGGTCCCACCTGACCCCATGCCGAACTCAGAAGTGAAACGCCGTAGCGCCGATGGTAGTGTGGGGTCTCCCCATGCGAG<br>AGTAGGGAACTGCCAGGCATCAAATAAAACGAAAGGCTCAGTCGAAAGACTGGGCCTTTCGTTTTATCTGTTGTTTGTCGGTGAACTG<br>GATCCTTACTCGAGTCTAGACTGCAGGCTTCCTCGCTCACTGACTCGCTGCGCTCGGTCGTTCGGCTGCGGCGAGCGGTATCAGCTC<br>ACTCAAAGGCGGTAATACGGTTATCCACAGAATCAGGGGATAACGCAGGAAAGAACATGTGAGCAAAAGGCCAGCAAAAGGCCAGGA<br>ACCGTAAAAAACTAGTGCTTGGATTCTCACCAATAAAAAACGCCCGGCGGCAACCGAGCGTTCTGAACAAATCCAGATGGAGTTCTGA<br>GGTCATTACTGGATCTATCAACAGGAGTCCAAGCGAGCTCGATATCAAATTACGCCCCGCCCTGCCACTCATCGCAGTACTGTTGTAA<br>TTCATTAAGCATTCTGCCGACATGGAAGCCATCACAAACGGCATGATGAACCTGAATCGCCAGCCGAGACGAAAGGGCCTCGTGATA<br>CGCCTATTTTTATAGGTTAATGTCATGATAATAATGGTTTCTTACCTCCCGTGGGGAAAAAATCATGGCAATTCTGGAAGAAATAGCGC<br>TTTCAGCCGGCAAACCGGCTGAAGCCGGATCTGCGATTCTGATAACAAACTAGCAACACCAGAACAGCCCGTTTGCGGGCAGCAAAA<br>CCCGTACCCTAGGTATAAACGCAGAAAGGCCCACCCGAAGGTGAGCCAGTGTGACTCTAGTAGAGAGCGTTCACCGACAAACAACAG<br>ATAAAACGAAAGGCCCAGTCTTTCGACTGAGCCTTTCGTTTTATTTGATGCCTGGAGATCCTTACTCGAGTTTGGATCCTTAAGCACCG<br>GTGGAGTGACGACCTTCAGCACGTTCGTACTGTTCAACGATGGTGTAGTCTTCGTTGTGGGAGGTGATGTCCAGTTTGATGTCGGTTT<br>TGTAAGCACCCGGCAGCTGAACCGGTTTTTTAGCCATGTAGGTGGTTTTAACTTCAGCGTCGTAGTGACCACCGTCTTTCAGTTTCAG<br>ACGCATTTTGATTTCACCTTTCAGAGCACCGTCTTCCGGGTACATACGTTCGGTGGAAGCTTCCCAACCCATGGTTTTTTTCTGCATAA<br>CCGGACCGTCGGACGGGAAGTTGGTACCACGCAGTTTAACTTTGTAGATGAACTCACCGTCTTGCAGGGAGGAGTCCTGGGTAACG<br>GTAACAACACCACCGTCTTCGAAGTTCATAACACGTTCCCATTTGAAACCTTCCGGGAAGGACAGTTTCAGGTAGTCCGGGATGTCAG<br>CCGGGTGTTTAACGTAAGCTTTGGAACCGTACTGGAACTGCGGGGACAGGATGTCCCAAGCGAACGGCAGCGGACCACCTTTGGTA<br>ACTTTCAGTTTAGCGGTCTGGGTACCTTCGTACGGACGACCTTCACCTTCACCTTCGATTTCGAACTCGTGACCGTTAACGGAACCTT<br>CCATACGAACTTTGAAACGCATGAACTCTTTGATAACGTCTTCGCTACTCGCCATGGTACCTTTCTCCTCTTTAATGAATTCGCTAGCA<br>CAATCCCTAGGACTGAGCTAGCTGTCAAGTCCGGAGGTATCCGGTGGAACCCAAAGGACGCCTTTGGTAACCGCAGGACACCGCAG<br>GATACCTGAGGTCGCCCGTGGTGGCCCATGGTCACCATAGGTCACCCTTGGCAACCAAAGGTGTCCTATGGCGGCCGGAGGCTGCC<br>ATAGGTCTCCGGTGGATACCGTAGGCGACGTCATCTAGATGCATTCGCGAGGTACCGAGCTCGAATTCACTGGCCGTCGTTTTACAA<br>CGTCGTGACTGGGAAAACCCTGGCGTTACCCAACTTAATCGCCTTGCAGCACATCCCCCTTTCGCCAGCTGGCGTAATAGCGAAGAG<br>GCCCGCACCGATCGCCCTTCCCAACAGTTGCGCAGCCTGAATGGCGAATGGCGCCTGATGCGGTATTTTCTCCTTACGCATCTGTGC<br>GGTATTTCACACCGCATATGGTGCACTCTCAGTACAATCTGCTCTGATGCCGCATAGTTAAGCCAGCCCCGACACCCGCCAACACCC<br>GCTGACGCGCCCTGACGGGCTTGTCTGCTCCCGGCATCCGCTTACAGACAAGCTGTGACCGTCTCCGGGAGCTGCATGTGTCAGAG<br>GTTTTCACCGTCATCACCGAAACGCGCGAGACGAAAGGGCCTCGTGATACGCCTATTTTTATAGGTTAATGTCATGATAATAATGGTTT<br>CTTAGACGTCAGGTGGCACTTTTCGGGGAAATGTGCGCGGAACCCCTATTTGTTTATTTTTCTAAATACATTCAAATATGTATCCGCTC<br>ATGAGACAATAACCCTGATAAATGCTTCAATAATATTGAAAAAGGAAGAGTATGAGTATTCAACATTTCCGTGTCGCCCTTATTCCCTTT<br>TTTGCGGCATTTTGCCTTCCTGTTTTTGCTCACCCAGAAACGCTGGTGAAAGTAAAAGATGCTGAAGATCAGTTGGGTGCACGAGTGG<br>GTTACATCGAACTGGATCTCAACAGCGGTAAGATCCTTGAGAGTTTTCGCCCCGAAGAACGTTTTCCAATGATGAGCACTTTTAAAGTT<br>CTGCTATGTGGCGCGGTATTATCCCGTATTGACGCCGGGCAAGAGCAACTCGGTCGCCGCATACACTATTCTCAGAATGACTTGGTT<br>GAGTACTCACCAGTCACAGAAAAGCATCTTACGGATGGCATGACAGTAAGAGAATTATGCAGTGCTGCCATAACCATGAGTGATAACA<br>CTGCGGCCAACTTACTTCTGACAACGATCGGAGGACCGAAGGAGCTAACCGCTTTTTTGCACAACATGGGGGATCATGTAACTCGCC<br>TTGATCGTTGGGAACCGGAGCTGAATGAAGCCATACCAAACGACGAGCGTGACACCACGATGCCTGTAGCAATGGCAACAACGTTGC<br>GCAAACTATTAACTGGCGAACTACTTACTCTAGCTTCCCGGCAACAATTAATAGACTGGATGGAGGCGGATAAAGTTGCAGGACCACT<br>TCTGCGCTCGGCCCTTCCGGCTGGCTGGTTTATTGCTGATAAATCTGGAGCCGGTGAGCGTGGGTCTCGCGGTATCATTGCAGCACT<br>GGGGCCAGATGGTAAGCCCTCCCGTATCGTAGTTATCTACACGACGGGGAGTCAGGCAACTATGGATGAACGAAATAGACAGATCGC<br>TGAGATAGGTGCCTCACTGATTAAGCATTGGTAACTGTCAGACCAAGTTTACTCATATATACTTTAGATTGATTTAAAACTTCATTTTTAA<br>TTTAAAAGGATCTAGGTGAAGATCCTTTTTGATAATCTCATGACCAAAATCCCTTAACGTGAGTTTTCGTTCCACTGAGCGTCAGACCC<br>CGTAGAAAAGATCAAAGGATCTTCAAGCAGCATAACCTTTTTCCGTGATGGTAACTTCACGGTAACCAAGATGTCGAGTTAACCACCCT<br>TTAGATTCATAAAGCGAAAATAATGCGGCTCCAACGTACCCACCTAAATGGAAACGGCGTTCACTCCAAGCGAAAAAACCCCGCCGAA<br>GCGGGGTTTTTTGCGTTAAAGCCGGTGACGGTAGTCGCTGGGAGTCCGGTCAAATTGACGAGCGAAGACGCGAGAGAAGGTTTGCT<br>GCGACACATAGCCTAAGTCCATGGCAATATCGAAAATCGGACGTTCTGTTGTCCGCAATTCAACGGCAGCCAACAACAAACGGCGTT<br>GACGGATATAGTCTCCTAATGTTTGATGTGTGACGGTACGAAACATGCGCTGTAAATACCACTTCGAGTAACCACTTTTCTTCGCTACA<br>ACATCAATATTAAGAGGCTGATCAATATGCTCATCGATCCAAGCAATAAGGTCCTGGATGATTTTTTGGTGGCTCATTGAACCACCTCC<br>GCCGTAAATACCACTGTTGGCCGCGATGGCAGATGGAATCGGATTTCCATCTTTCAGAAGACCCTGCATCGCTTTCACAATCAACTCA<br>CAGTCGCTATTCGTAGCAAAGATCGGAATAGTCAGTTCCATATTAAGGTACGAACGCCATGCACCTTTAGGGACTTCAACTTTGATCGT<br>GTACTTACGGTTTTGCGCACTCGATTGACGTACAGAACAAGTAACTTTGTATGCTTGGCTACGGCTGTTGGAGGAAATCCACTCGGCA<br>ATGCCGTTAGCGAAGTTGCTAGGGGCAACAGTTACATCACCAGTACCACCATTATCCACAAGAACGAACTGCGTAAAATTGCTCGCGG<br>GACCCATAGATCCTTTCTCCTCTTTAGATCTTTTGAATTCCCAAAAAAACGGGTATGGAGAAACAGTAGAGAGTTGCGATAAAAAGCGT<br>CAGGTAGAATCCGCTAATCTTATGGATAAAAATGCTATGGCATAGCAAAGTGTGACGCCGTGCAAATAATCAATGTGGACTTTTCTGCC<br>GTGATTATAGACACTTTTGTTACGCGTTTTTGTCATGGCTTTGGTCCCGCTTTGTTACAGAATGCTTTTAATAAGCGGGGTTACCGGTTT<br>GGTTAGCGAGAAGAGCCAGTAAAAGACGCAGTGACGGCAATGTCTGATGCAATATGGACAATTGGTTTCTTCTCTGAATGGCGGGAG<br>TATGAAAAGTTGAGATCCTTTTTTTCTGCGCGTAATCTGCTGCTTGCAAACAAAAAAACCACCGCTACCAGCGGTGGTTTGTTTGCCGG<br>ATCAAGAGCTACCAACTCTTTTTCCGAAGGTAACTGGCTTCAGCAGAGCGCAGATACCAAATACTGTTCTTCTAGTGTAGCCGTAGTTA<br>GGCCACCACTTCAAGAACTCTGTAGCACCGCCTACATACCTCGCTCTGCTAATCCTGTTACCAGTGGCTGCTGCCAGTGGCGATAAGT	

	CGTGTCTTACCGGGTTGGACTCAAGACGATAGTTACCGGATAAGGCGCAGCGGTCGGGCTGAACGGGGGGTTCGTGCACACAGCCC<br>AGCTTGGAGCGAACGACCTACACCGAACT	
pUC57_<br>106_2x	TCGCGCGTTTCGGTGATGACGGTGAAAACCTCTGACACATGCAGCTCCCGGAGACGGTCACAGCTTGTCTGTAAGCGGATGCCGGG<br>AGCAGACAAGCCCGTCAGGGCGCGTCAGCGGGTGTTGGCGGGTGTCGGGGCTGGCTTAACTATGCGGCATCAGAGCAGATTGTACT<br>GAGAGTGCACCATATGCGGTGTGAAATACCGCACAGATGCGTAAGGAGAAAATACCGCATCAGGCGCCATTCGCCATTCAGGCTGCG<br>CAACTGTTGGGAAGGGCGATCGGTGCGGGCCTCTTCGCTATTACGCCAGCTGGCGAAAGGGGGATGTGCTGCAAGGCGATTAAGTT<br>GGGTAACGCCAGGGTTTTCCCAGTCACGACGTTGTAAAACGACGGCCAGTGAATTCGAGCTCGGTACCTCGCGAATGCATCTAGATG<br>ACGTCGCCTACGGTATCCACCGGAGACCTATGGCAGCCTCCGGCCGCCATAGGACACCTTTGGTTGCCAAGGGTGACCTATGGTGA<br>CCATGGGCCACCACCGGTGTCCTGCGGTTACCAAAGGCGTCCTGCGGTTACCAAAGGCGTCCTTTGGGTTCCACCGGATACCTCCG<br>GACTTGACAGCTAGCTCAGTCCTAGGGATTGTGCTAGCGAATTCATTAAAGAGGAGAAAGGTACCATGGCGAGTAGCGAAGACGTTA<br>TCAAAGAGTTCATGCGTTTCAAAGTTCGTATGGAAGGTTCCGTTAACGGTCACGAGTTCGAAATCGAAGGTGAAGGTGAAGGTCGTCC<br>GTACGAAGGTACCCAGACCGCTAAACTGAAAGTTACCAAAGGTGGTCCGCTGCCGTTCGCTTGGGACATCCTGTCCCCGCAGTTCCA<br>GTACGGTTCCAAAGCTTACGTTAAACACCCGGCTGACATCCCGGACTACCTGAAACTGTCCTTCCCGGAAGGTTTCAAATGGGAACGT<br>GTTATGAACTTCGAAGACGGTGGTGTTGTTACCGTTACCCAGGACTCCTCCCTGCAAGACGGTGAGTTCATCTACAAAGTTAAACTGC<br>GTGGTACCAACTTCCCGTCCGACGGTCCGGTTATGCAGAAAAAAACCATGGGTTGGGAAGCTTCCACCGAACGTATGTACCCGGAAG<br>ACGGTGCTCTGAAAGGTGAAATCAAAATGCGTCTGAAACTGAAAGACGGTGGTCACTACGACGCTGAAGTTAAAACCACCTACATGGC<br>TAAAAAACCGGTTCAGCTGCCGGGTGCTTACAAAACCGACATCAAACTGGACATCACCTCCCACAACGAAGACTACACCATCGTTGAA<br>CAGTACGAACGTGCTGAAGGTCGTCACTCCACCGGTGCTTAAGGATCCAAACTCGAGTAAGGATCTCCAGGCATCAAATAAAACGAAA<br>GGCTCAGTCGAAAGACTGGGCCTTTCGTTTTATCTGTTGTTTGTCGGTGAACGCTCTCTACTAGAGTCACACTGGCTCACCTTCGGGT<br>GGGCCTTTCTGCGTTTATACCTAGGATCGGATCCCGGGCCCGTCGACTGCAGAGGCCTGCATGCAAGCTTGGCGTAATCATGGTCAT<br>AGCTGTTTCCTGTGTGAAATTGTTATCCGCTCACAATTCCACACAACATACGAGCCGGAAGCATAAAGTGTAAAGCCTGGGGTGCCTA<br>ATGAGTGAGCTAACTCACATTAATTGCGTTGCGCTCACTGCCCGCTTTCCAGTCGGGAAACCTGTCGTGCCAGCTGCATTAATGAATC<br>GGCCAACGCGCGGGGAGAGGCGGTTTGCGTATTGGGCGCTCTTCCGCTTCCTCGCTCACTGACTCGCTGCGCTCGGTCGTTCGGCT<br>GCGGCGAGCGGTATCAGCTCACTCAAAGGCGGTAATACGGTTATCCACAGAATCAGGGGATAACGCAGGAAAGAACATGTGAGCAAA<br>AGGCCAGCAAAAGGCCAGGAACCGTAAAAAGGCCGCGTTGCTGGCGTTTTTCCATAGGCTCCGCCCCCCTGACGAGCATCACAAAAA<br>TCGACGCTCAAGTCAGAGGTGGCGAAACCCGACAGGACTATAAAGATACCAGGCGTTTCCCCCTGGAAGCTCCCTCGTGCGCTCTCC<br>TGTTCCGACCCTGCCGCTTACCGGATACCTGTCCGCCTTTCTCCCTTCGGGAAGCGTGGCGCTTTCTCATAGCTCACGCTGTAGGTAT<br>CTCAGTTCGGTGTAGGTCGTTCGCTCCAAGCTGGGCTGTGTGCACGAACCCCCCGTTCAGCCCGACCGCTGCGCCTTATCCGGTAA<br>CTATCGTCTTGAGTCCAACCCGGTAAGACACGACTTATCGCCACTGGCAGCAGCCACTGGTAACAGGATTAGCAGAGCGAGGTATGT<br>AGGCGGTGCTACAGAGTTCTTGAAGTGGTGGCCTAACTACGGCTACACTAGAAGAACAGTATTTGGTATCTGCGCTCTGCTGAAGCC<br>AGTTACCTTCGGAAAAAGAGTTGGTAGCTCTTGATCCGGCAAACAAACCACCGCTGGTAGCGGTGGTTTTTTTGTTTGCAAGCAGCAG<br>ATTACGCGCAGAAAAAAAGGATCTCAAGAAGATCCTTTGATCTTTTCTACGGGGTCTGACGCTCAGTGGAACGAAAACTCACGTTAAG<br>GGATTTTGGTCATGAGATTATCAAAAAGGATCTTCACCTAGATCCTTTTAAATTAAAAATGAAGTTTTAAATCAATCTAAAGTATATATGA<br>GTAAACTTGGTCTGACAGTTACCAATGCTTAATCAGTGAGGCACCTATCTCAGCGATCTGTCTATTTCGTTCATCCATAGTTGCCTGAC<br>TCCCCGTCGTGTAGATAACTACGATACGGGAGGGCTTACCATCTGGCCCCAGTGCTGCAATGATACCGCGAGACTCACGCTCACCGG<br>CTCCAGATTTATCAGCAATAAACCAGCCAGCCGGAAGGGCCGAGCGCAGAAGTGGTCCTGCAACTTTATCCGCCTCCATCCAGTCTA<br>TTAATTGTTGCCGGGAAGCTAGAGTAAGTAGTTCGCCAGTTAATAGTTTGCGCAACGTTGTTGCCATTGCTACAGGCATCGTGGTGTC<br>ACGCTCGTCGTTTGGTATGGCTTCATTCAGCTCCGGTTCCCAACGATCAAGGCGAGTTACATGATCCCCCATGTTGTGCAAAAAAGCG<br>GTTAGCTCCTTCGGTCCTCCGATCGTTGTCAGAAGTAAGTTGGCCGCAGTGTTATCACTCATGGTTATGGCAGCACTGCATAATTCTC<br>TTACTGTCATGCCATCCGTAAGATGCTTTTCTGTGACTGGTGAGTACTCAACCAAGTCATTCTGAGAATAGTGTATGCGGCGACCGAG<br>TTGCTCTTGCCCGGCGTCAATACGGGATAATACCGCGCCACATAGCAGAACTTTAAAAGTGCTCATCATTGGAAAACGTTCTTCGGGG<br>CGAAAACTCTCAAGGATCTTACCGCTGTTGAGATCCAGTTCGATGTAACCCACTCGTGCACCCAACTGATCTTCAGCATCTTTTACTTT<br>CACCAGCGTTTCTGGGTGAGCAAAAACAGGAAGGCAAAATGCCGCAAAAAAGGGAATAAGGGCGACACGGAAATGTTGAATACTCAT<br>ACTCTTCCTTTTTCAATATTATTGAAGCATTTATCAGGGTTATTGTCTCATGAGCGGATACATATTTGAATGTATTTAGAAAAATAAACAA<br>ATAGGGGTTCCGCGCACATTTCCCCGAAAAGTGCCACCTGACGTCTAAGAAACCATTATTATCATGACATTAACCTATAAAAATAGGCG<br>TATCACGAGGCCCTTTCGTC	BioCat
pSJ106_<br>2x	GAGATACCTACAGCGTGAGCTATGAGAAAGCGCCACGCTTCCCGAAGGGAGAAAGGCGGACAGGTATCCGGTAAGCGGCAGGGTC<br>GGAACAGGAGAGCGCACGAGGGAGCTTCCAGGGGGAAACGCCTGGTATCTTTATAGTCCTGTCGGGTTTCGCCACCTCTGACTTGA<br>GCGTCGATTTTTGTGATGCTCGTCAGGGGGGCGGAGCCTATGGAAAAACGCCAGCAACGCGGCCTTTTTACGGTTCCTGGCCTTTTG<br>CTGGCCTTTTGCTCTCAGATAAAATATTTCTAGATTTCAGTGCAATTTATCTCTTCAAATGTAGCACCTGAAGTCAGCCCCATACGATAT<br>AAGTTGTTACTAGATTGACAGCTAGCTCAGTCCTAGGTATAATACTAGTAGGACGCCTTTGGTAACCGCGTTTTAGAGCTAGAAATAGC<br>AAGTTAAAATAAGGCTAGTCCGTTATCAACTTGAAAAAGTGGCACATGAGGATCACCCATGTGCTTTTTTTGAAGCTTGGGCCCGAACA<br>AAAACTCATCTCAGAAGAGGATCTGAATAGCGCCGTCGACCATCATCATCATCATCATTGAGTTTAAACGGTCTCCAGCTTGGCTGTTT<br>TGGCGGATGAGAGAAGATTTTCAGCCTGATACAGATTAAATCAGAACGCAGAAGCGGTCTGATAAAACAGAATTTGCCTGGCGGCAGT<br>AGCGCGGTGGTCCCACCTGACCCCATGCCGAACTCAGAAGTGAAACGCCGTAGCGCCGATGGTAGTGTGGGGTCTCCCCATGCGAG<br>AGTAGGGAACTGCCAGGCATCAAATAAAACGAAAGGCTCAGTCGAAAGACTGGGCCTTTCGTTTTATCTGTTGTTTGTCGGTGAACTG<br>GATCCTTACTCGAGTCTAGACTGCAGGCTTCCTCGCTCACTGACTCGCTGCGCTCGGTCGTTCGGCTGCGGCGAGCGGTATCAGCTC	This<br>study

	ACTCAAAGGCGGTAATACGGTTATCCACAGAATCAGGGGATAACGCAGGAAAGAACATGTGAGCAAAAGGCCAGCAAAAGGCCAGGA<br>ACCGTAAAAAACTAGTGCTTGGATTCTCACCAATAAAAAACGCCCGGCGGCAACCGAGCGTTCTGAACAAATCCAGATGGAGTTCTGA<br>GGTCATTACTGGATCTATCAACAGGAGTCCAAGCGAGCTCGATATCAAATTACGCCCCGCCCTGCCACTCATCGCAGTACTGTTGTAA<br>TTCATTAAGCATTCTGCCGACATGGAAGCCATCACAAACGGCATGATGAACCTGAATCGCCAGCCGAGACGAAAGGGCCTCGTGATA<br>CGCCTATTTTTATAGGTTAATGTCATGATAATAATGGTTTCTTACCTAGGTATAAACGCAGAAAGGCCCACCCGAAGGTGAGCCAGTGT<br>GACTCTAGTAGAGAGCGTTCACCGACAAACAACAGATAAAACGAAAGGCCCAGTCTTTCGACTGAGCCTTTCGTTTTATTTGATGCCT<br>GGAGATCCTTACTCGAGTTTGGATCCTTAAGCACCGGTGGAGTGACGACCTTCAGCACGTTCGTACTGTTCAACGATGGTGTAGTCTT<br>CGTTGTGGGAGGTGATGTCCAGTTTGATGTCGGTTTTGTAAGCACCCGGCAGCTGAACCGGTTTTTTAGCCATGTAGGTGGTTTTAAC<br>TTCAGCGTCGTAGTGACCACCGTCTTTCAGTTTCAGACGCATTTTGATTTCACCTTTCAGAGCACCGTCTTCCGGGTACATACGTTCGG<br>TGGAAGCTTCCCAACCCATGGTTTTTTTCTGCATAACCGGACCGTCGGACGGGAAGTTGGTACCACGCAGTTTAACTTTGTAGATGAA<br>CTCACCGTCTTGCAGGGAGGAGTCCTGGGTAACGGTAACAACACCACCGTCTTCGAAGTTCATAACACGTTCCCATTTGAAACCTTCC<br>GGGAAGGACAGTTTCAGGTAGTCCGGGATGTCAGCCGGGTGTTTAACGTAAGCTTTGGAACCGTACTGGAACTGCGGGGACAGGAT<br>GTCCCAAGCGAACGGCAGCGGACCACCTTTGGTAACTTTCAGTTTAGCGGTCTGGGTACCTTCGTACGGACGACCTTCACCTTCACC<br>TTCGATTTCGAACTCGTGACCGTTAACGGAACCTTCCATACGAACTTTGAAACGCATGAACTCTTTGATAACGTCTTCGCTACTCGCCA<br>TGGTACCTTTCTCCTCTTTAATGAATTCGCTAGCACAATCCCTAGGACTGAGCTAGCTGTCAAGTCCGGAGGTATCCGGTGGAACCCA<br>AAGGACGCCTTTGGTAACCGCAGGACGCCTTTGGTAACCGCAGGACACCGGTGGTGGCCCATGGTCACCATAGGTCACCCTTGGCA<br>ACCAAAGGTGTCCTATGGCGGCCGGAGGCTGCCATAGGTCTCCGGTGGATACCGTAGGCGACGTCATCTAGATGCATTCGCGAGGT<br>ACCGAGCTCGAATTCACTGGCCGTCGTTTTACAACGTCGTGACTGGGAAAACCCTGGCGTTACCCAACTTAATCGCCTTGCAGCACAT<br>CCCCCTTTCGCCAGCTGGCGTAATAGCGAAGAGGCCCGCACCGATCGCCCTTCCCAACAGTTGCGCAGCCTGAATGGCGAATGGCG<br>CCTGATGCGGTATTTTCTCCTTACGCATCTGTGCGGTATTTCACACCGCATATGGTGCACTCTCAGTACAATCTGCTCTGATGCCGCAT<br>AGTTAAGCCAGCCCCGACACCCGCCAACACCCGCTGACGCGCCCTGACGGGCTTGTCTGCTCCCGGCATCCGCTTACAGACAAGCT<br>GTGACCGTCTCCGGGAGCTGCATGTGTCAGAGGTTTTCACCGTCATCACCGAAACGCGCGAGACGAAAGGGCCTCGTGATACGCCT<br>ATTTTTATAGGTTAATGTCATGATAATAATGGTTTCTTAGACGTCAGGTGGCACTTTTCGGGGAAATGTGCGCGGAACCCCTATTTGTT<br>TATTTTTCTAAATACATTCAAATATGTATCCGCTCATGAGACAATAACCCTGATAAATGCTTCAATAATATTGAAAAAGGAAGAGTATGA<br>GTATTCAACATTTCCGTGTCGCCCTTATTCCCTTTTTTGCGGCATTTTGCCTTCCTGTTTTTGCTCACCCAGAAACGCTGGTGAAAGTAA<br>AAGATGCTGAAGATCAGTTGGGTGCACGAGTGGGTTACATCGAACTGGATCTCAACAGCGGTAAGATCCTTGAGAGTTTTCGCCCCG<br>AAGAACGTTTTCCAATGATGAGCACTTTTAAAGTTCTGCTATGTGGCGCGGTATTATCCCGTATTGACGCCGGGCAAGAGCAACTCGG<br>TCGCCGCATACACTATTCTCAGAATGACTTGGTTGAGTACTCACCAGTCACAGAAAAGCATCTTACGGATGGCATGACAGTAAGAGAA<br>TTATGCAGTGCTGCCATAACCATGAGTGATAACACTGCGGCCAACTTACTTCTGACAACGATCGGAGGACCGAAGGAGCTAACCGCTT<br>TTTTGCACAACATGGGGGATCATGTAACTCGCCTTGATCGTTGGGAACCGGAGCTGAATGAAGCCATACCAAACGACGAGCGTGACA<br>CCACGATGCCTGTAGCAATGGCAACAACGTTGCGCAAACTATTAACTGGCGAACTACTTACTCTAGCTTCCCGGCAACAATTAATAGA<br>CTGGATGGAGGCGGATAAAGTTGCAGGACCACTTCTGCGCTCGGCCCTTCCGGCTGGCTGGTTTATTGCTGATAAATCTGGAGCCGG<br>TGAGCGTGGGTCTCGCGGTATCATTGCAGCACTGGGGCCAGATGGTAAGCCCTCCCGTATCGTAGTTATCTACACGACGGGGAGTCA<br>GGCAACTATGGATGAACGAAATAGACAGATCGCTGAGATAGGTGCCTCACTGATTAAGCATTGGTAACTGTCAGACCAAGTTTACTCA<br>TATATACTTTAGATTGATTTAAAACTTCATTTTTAATTTAAAAGGATCTAGGTGAAGATCCTTTTTGATAATCTCATGACCAAAATCCCTTA<br>ACGTGAGTTTTCGTTCCACTGAGCGTCAGACCCCGTAGAAAAGATCAAAGGATCTTCAAGCAGCATAACCTTTTTCCGTGATGGTAAC<br>TTCACGGTAACCAAGATGTCGAGTTAACCACCCTTTAGATTCATAAAGCGAAAATAATGCGGCTCCAACGTACCCACCTAAATGGAAAC<br>GGCGTTCACTCCAAGCGAAAAAACCCCGCCGAAGCGGGGTTTTTTGCGTTAAAGCCGGTGACGGTAGTCGCTGGGAGTCCGGTCAA<br>ATTGACGAGCGAAGACGCGAGAGAAGGTTTGCTGCGACACATAGCCTAAGTCCATGGCAATATCGAAAATCGGACGTTCTGTTGTCC<br>GCAATTCAACGGCAGCCAACAACAAACGGCGTTGACGGATATAGTCTCCTAATGTTTGATGTGTGACGGTACGAAACATGCGCTGTAA<br>ATACCACTTCGAGTAACCACTTTTCTTCGCTACAACATCAATATTAAGAGGCTGATCAATATGCTCATCGATCCAAGCAATAAGGTCCT<br>GGATGATTTTTTGGTGGCTCATTGAACCACCTCCGCCGTAAATACCACTGTTGGCCGCGATGGCAGATGGAATCGGATTTCCATCTTT<br>CAGAAGACCCTGCATCGCTTTCACAATCAACTCACAGTCGCTATTCGTAGCAAAGATCGGAATAGTCAGTTCCATATTAAGGTACGAAC<br>GCCATGCACCTTTAGGGACTTCAACTTTGATCGTGTACTTACGGTTTTGCGCACTCGATTGACGTACAGAACAAGTAACTTTGTATGCT<br>TGGCTACGGCTGTTGGAGGAAATCCACTCGGCAATGCCGTTAGCGAAGTTGCTAGGGGCAACAGTTACATCACCAGTACCACCATTA<br>TCCACAAGAACGAACTGCGTAAAATTGCTCGCGGGACCCATAGATCCTTTCTCCTCTTTAGATCTTTTGAATTCCCAAAAAAACGGGTA<br>TGGAGAAACAGTAGAGAGTTGCGATAAAAAGCGTCAGGTAGAATCCGCTAATCTTATGGATAAAAATGCTATGGCATAGCAAAGTGTG<br>ACGCCGTGCAAATAATCAATGTGGACTTTTCTGCCGTGATTATAGACACTTTTGTTACGCGTTTTTGTCATGGCTTTGGTCCCGCTTTG<br>TTACAGAATGCTTTTAATAAGCGGGGTTACCGGTTTGGTTAGCGAGAAGAGCCAGTAAAAGACGCAGTGACGGCAATGTCTGATGCAA<br>TATGGACAATTGGTTTCTTCTCTGAATGGCGGGAGTATGAAAAGTTGAGATCCTTTTTTTCTGCGCGTAATCTGCTGCTTGCAAACAAA<br>AAAACCACCGCTACCAGCGGTGGTTTGTTTGCCGGATCAAGAGCTACCAACTCTTTTTCCGAAGGTAACTGGCTTCAGCAGAGCGCA<br>GATACCAAATACTGTTCTTCTAGTGTAGCCGTAGTTAGGCCACCACTTCAAGAACTCTGTAGCACCGCCTACATACCTCGCTCTGCTAA<br>TCCTGTTACCAGTGGCTGCTGCCAGTGGCGATAAGTCGTGTCTTACCGGGTTGGACTCAAGACGATAGTTACCGGATAAGGCGCAGC<br>GGTCGGGCTGAACGGGGGGTTCGTGCACACAGCCCAGCTTGGAGCGAACGACCTACACCGAACT	
p107	GAGATACCTACAGCGTGAGCTATGAGAAAGCGCCACGCTTCCCGAAGGGAGAAAGGCGGACAGGTATCCGGTAAGCGGCAGGGTC<br>GGAACAGGAGAGCGCACGAGGGAGCTTCCAGGGGGAAACGCCTGGTATCTTTATAGTCCTGTCGGGTTTCGCCACCTCTGACTTGA<br>GCGTCGATTTTTGTGATGCTCGTCAGGGGGGCGGAGCCTATGGAAAAACGCCAGCAACGCGGCCTTTTTACGGTTCCTGGCCTTTTG	This study

	CTGGCCTTTTGCTCTCAGATAAAATATTTCTAGATTTCAGTGCAATTTATCTCTTCAAATGTAGCACCTGAAGTCAGCCCCATACGATAT<br>AAGTTGTTACTAGATTGACAGCTAGCTCAGTCCTAGGTATAATACTAGTCGGTGTCCTGCGGTTACCAAGTTTTAGAGCTAGAAATAGC<br>AAGTTAAAATAAGGCTAGTCCGTTATCAACTTGAAAAAGTGGCACATGAGGATCACCCATGTGCTTTTTTTGAAGCTTGGGCCCGAACA<br>AAAACTCATCTCAGAAGAGGATCTGAATAGCGCCGTCGACCATCATCATCATCATCATTGAGTTTAAACGGTCTCCAGCTTGGCTGTTT<br>TGGCGGATGAGAGAAGATTTTCAGCCTGATACAGATTAAATCAGAACGCAGAAGCGGTCTGATAAAACAGAATTTGCCTGGCGGCAGT<br>AGCGCGGTGGTCCCACCTGACCCCATGCCGAACTCAGAAGTGAAACGCCGTAGCGCCGATGGTAGTGTGGGGTCTCCCCATGCGAG<br>AGTAGGGAACTGCCAGGCATCAAATAAAACGAAAGGCTCAGTCGAAAGACTGGGCCTTTCGTTTTATCTGTTGTTTGTCGGTGAACTG<br>GATCCTTACTCGAGTCTAGACTGCAGGCTTCCTCGCTCACTGACTCGCTGCGCTCGGTCGTTCGGCTGCGGCGAGCGGTATCAGCTC<br>ACTCAAAGGCGGTAATACGGTTATCCACAGAATCAGGGGATAACGCAGGAAAGAACATGTGAGCAAAAGGCCAGCAAAAGGCCAGGA<br>ACCGTAAAAAACTAGTGCTTGGATTCTCACCAATAAAAAACGCCCGGCGGCAACCGAGCGTTCTGAACAAATCCAGATGGAGTTCTGA<br>GGTCATTACTGGATCTATCAACAGGAGTCCAAGCGAGCTCGATATCAAATTACGCCCCGCCCTGCCACTCATCGCAGTACTGTTGTAA<br>TTCATTAAGCATTCTGCCGACATGGAAGCCATCACAAACGGCATGATGAACCTGAATCGCCAGCCGAGACGAAAGGGCCTCGTGATA<br>CGCCTATTTTTATAGGTTAATGTCATGATAATAATGGTTTCTTAGACGTCAGGTGGCACTTTTCGGGGAAATGTGCGCGGAACCCCTAT<br>TTGTTTATTTTTCTAAATACATTCAAATATGTATCCGCTCATGAGACAATAACCCTGATAAATGCTTCAATAATATTGAAAAAGGAAGAGT<br>ATGAGTATTCAACATTTCCGTGTCGCCCTTATTCCCTTTTTTGCGGCATTTTGCCTTCCTGTTTTTGCTCACCCAGAAACGCTGGTGAAA<br>GTAAAAGATGCTGAAGATCAGTTGGGTGCACGAGTGGGTTACATCGAACTGGATCTCAACAGCGGTAAGATCCTTGAGAGTTTTCGCC<br>CCGAAGAACGTTTTCCAATGATGAGCACTTTTAAAGTTCTGCTATGTGGCGCGGTATTATCCCGTATTGACGCCGGGCAAGAGCAACT<br>CGGTCGCCGCATACACTATTCTCAGAATGACTTGGTTGAGTACTCACCAGTCACAGAAAAGCATCTTACGGATGGCATGACAGTAAGA<br>GAATTATGCAGTGCTGCCATAACCATGAGTGATAACACTGCGGCCAACTTACTTCTGACAACGATCGGAGGACCGAAGGAGCTAACC<br>GCTTTTTTGCACAACATGGGGGATCATGTAACTCGCCTTGATCGTTGGGAACCGGAGCTGAATGAAGCCATACCAAACGACGAGCGT<br>GACACCACGATGCCTGTAGCAATGGCAACAACGTTGCGCAAACTATTAACTGGCGAACTACTTACTCTAGCTTCCCGGCAACAATTAA<br>TAGACTGGATGGAGGCGGATAAAGTTGCAGGACCACTTCTGCGCTCGGCCCTTCCGGCTGGCTGGTTTATTGCTGATAAATCTGGAG<br>CCGGTGAGCGTGGGTCTCGCGGTATCATTGCAGCACTGGGGCCAGATGGTAAGCCCTCCCGTATCGTAGTTATCTACACGACGGGG<br>AGTCAGGCAACTATGGATGAACGAAATAGACAGATCGCTGAGATAGGTGCCTCACTGATTAAGCATTGGTAACTGTCAGACCAAGTTT<br>ACTCATATATACTTTAGATTGATTTAAAACTTCATTTTTAATTTAAAAGGATCTAGGTGAAGATCCTTTTTGATAATCTCATGACCAAAATC<br>CCTTAACGTGAGTTTTCGTTCCACTGAGCGTCAGACCCCGTAGAAAAGATCAAAGGATCTTCTTGAGATCCTTTTTTTCTGCGCGTAAT<br>CTGCTGCTTGCAAACAAAAAAACCACCGCTACCAGCGGTGGTTTGTTTGCCGGATCAAGAGCTACCAACTCTTTTTCCGAAGGTAACT<br>GGCTTCAGCAGAGCGCAGATACCAAATACTGTTCTTCTAGTGTAGCCGTAGTTAGGCCACCACTTCAAGAACTCTGTAGCACCGCCTA<br>CATACCTCGCTCTGCTAATCCTGTTACCAGTGGCTGCTGCCAGTGGCGATAAGTCGTGTCTTACCGGGTTGGACTCAAGACGATAGTT<br>ACCGGATAAGGCGCAGCGGTCGGGCTGAACGGGGGGTTCGTGCACACAGCCCAGCTTGGAGCGAACGACCTACACCGAACT	
pS107	GAGATACCTACAGCGTGAGCTATGAGAAAGCGCCACGCTTCCCGAAGGGAGAAAGGCGGACAGGTATCCGGTAAGCGGCAGGGTC<br>GGAACAGGAGAGCGCACGAGGGAGCTTCCAGGGGGAAACGCCTGGTATCTTTATAGTCCTGTCGGGTTTCGCCACCTCTGACTTGA<br>GCGTCGATTTTTGTGATGCTCGTCAGGGGGGCGGAGCCTATGGAAAAACGCCAGCAACGCGGCCTTTTTACGGTTCCTGGCCTTTTG<br>CTGGCCTTTTGCTCTCAGATAAAATATTTCTAGATTTCAGTGCAATTTATCTCTTCAAATGTAGCACCTGAAGTCAGCCCCATACGATAT<br>AAGTTGTTACTAGATTGACAGCTAGCTCAGTCCTAGGTATAATACTAGTCGGTGTCCTGCGGTTACCAAGTTTTAGAGCTAGAAATAGC<br>AAGTTAAAATAAGGCTAGTCCGTTATCAACTTGAAAAAGTGGCACATGAGGATCACCCATGTGCTTTTTTTGAAGCTTGGGCCCGAACA<br>AAAACTCATCTCAGAAGAGGATCTGAATAGCGCCGTCGACCATCATCATCATCATCATTGAGTTTAAACGGTCTCCAGCTTGGCTGTTT<br>TGGCGGATGAGAGAAGATTTTCAGCCTGATACAGATTAAATCAGAACGCAGAAGCGGTCTGATAAAACAGAATTTGCCTGGCGGCAGT<br>AGCGCGGTGGTCCCACCTGACCCCATGCCGAACTCAGAAGTGAAACGCCGTAGCGCCGATGGTAGTGTGGGGTCTCCCCATGCGAG<br>AGTAGGGAACTGCCAGGCATCAAATAAAACGAAAGGCTCAGTCGAAAGACTGGGCCTTTCGTTTTATCTGTTGTTTGTCGGTGAACTG<br>GATCCTTACTCGAGTCTAGACTGCAGGCTTCCTCGCTCACTGACTCGCTGCGCTCGGTCGTTCGGCTGCGGCGAGCGGTATCAGCTC<br>ACTCAAAGGCGGTAATACGGTTATCCACAGAATCAGGGGATAACGCAGGAAAGAACATGTGAGCAAAAGGCCAGCAAAAGGCCAGGA<br>ACCGTAAAAAACTAGTGCTTGGATTCTCACCAATAAAAAACGCCCGGCGGCAACCGAGCGTTCTGAACAAATCCAGATGGAGTTCTGA<br>GGTCATTACTGGATCTATCAACAGGAGTCCAAGCGAGCTCGATATCAAATTACGCCCCGCCCTGCCACTCATCGCAGTACTGTTGTAA<br>TTCATTAAGCATTCTGCCGACATGGAAGCCATCACAAACGGCATGATGAACCTGAATCGCCAGCCGAGACGAAAGGGCCTCGTGATA<br>CGCCTATTTTTATAGGTTAATGTCATGATAATAATGGTTTCTTAGACGTCAGGTGGCACTTTTCGGGGAAATGTGCGCGGAACCCCTAT<br>TTGTTTATTTTTCTAAATACATTCAAATATGTATCCGCTCATGAGACAATAACCCTGATAAATGCTTCAATAATATTGAAAAAGGAAGAGT<br>ATGAGTATTCAACATTTCCGTGTCGCCCTTATTCCCTTTTTTGCGGCATTTTGCCTTCCTGTTTTTGCTCACCCAGAAACGCTGGTGAAA<br>GTAAAAGATGCTGAAGATCAGTTGGGTGCACGAGTGGGTTACATCGAACTGGATCTCAACAGCGGTAAGATCCTTGAGAGTTTTCGCC<br>CCGAAGAACGTTTTCCAATGATGAGCACTTTTAAAGTTCTGCTATGTGGCGCGGTATTATCCCGTATTGACGCCGGGCAAGAGCAACT<br>CGGTCGCCGCATACACTATTCTCAGAATGACTTGGTTGAGTACTCACCAGTCACAGAAAAGCATCTTACGGATGGCATGACAGTAAGA<br>GAATTATGCAGTGCTGCCATAACCATGAGTGATAACACTGCGGCCAACTTACTTCTGACAACGATCGGAGGACCGAAGGAGCTAACC<br>GCTTTTTTGCACAACATGGGGGATCATGTAACTCGCCTTGATCGTTGGGAACCGGAGCTGAATGAAGCCATACCAAACGACGAGCGT<br>GACACCACGATGCCTGTAGCAATGGCAACAACGTTGCGCAAACTATTAACTGGCGAACTACTTACTCTAGCTTCCCGGCAACAATTAA<br>TAGACTGGATGGAGGCGGATAAAGTTGCAGGACCACTTCTGCGCTCGGCCCTTCCGGCTGGCTGGTTTATTGCTGATAAATCTGGAG<br>CCGGTGAGCGTGGGTCTCGCGGTATCATTGCAGCACTGGGGCCAGATGGTAAGCCCTCCCGTATCGTAGTTATCTACACGACGGGG<br>AGTCAGGCAACTATGGATGAACGAAATAGACAGATCGCTGAGATAGGTGCCTCACTGATTAAGCATTGGTAACTGTCAGACCAAGTTT	This study

	ACTCATATATACTTTAGATTGATTTAAAACTTCATTTTTAATTTAAAAGGATCTAGGTGAAGATCCTTTTTGATAATCTCATGACCAAAATC<br>CCTTAACGTGAGTTTTCGTTCCACTGAGCGTCAGACCCCGTAGAAAAGATCAAAGGATCTTCAAGCAGCATAACCTTTTTCCGTGATG<br>GTAACTTCACGGTAACCAAGATGTCGAGTTAACCACCCTTTAGATTCATAAAGCGAAAATAATGCGGCTCCAACGTACCCACCTAAATG<br>GAAACGGCGTTCACTCCAAGCGAAAAAACCCCGCCGAAGCGGGGTTTTTTGCGTTAAAGCCGGTGACGGTAGTCGCTGGGAGTCCG<br>GTCAAATTGACGAGCGAAGACGCGAGAGAAGGTTTGCTGCGACACATAGCCTAAGTCCATGGCAATATCGAAAATCGGACGTTCTGT<br>TGTCCGCAATTCAACGGCAGCCAACAACAAACGGCGTTGACGGATATAGTCTCCTAATGTTTGATGTGTGACGGTACGAAACATGCGC<br>TGTAAATACCACTTCGAGTAACCACTTTTCTTCGCTACAACATCAATATTAAGAGGCTGATCAATATGCTCATCGATCCAAGCAATAAGG<br>TCCTGGATGATTTTTTGGTGGCTCATTGAACCACCTCCGCCGTAAATACCACTGTTGGCCGCGATGGCAGATGGAATCGGATTTCCAT<br>CTTTCAGAAGACCCTGCATCGCTTTCACAATCAACTCACAGTCGCTATTCGTAGCAAAGATCGGAATAGTCAGTTCCATATTAAGGTAC<br>GAACGCCATGCACCTTTAGGGACTTCAACTTTGATCGTGTACTTACGGTTTTGCGCACTCGATTGACGTACAGAACAAGTAACTTTGTA<br>TGCTTGGCTACGGCTGTTGGAGGAAATCCACTCGGCAATGCCGTTAGCGAAGTTGCTAGGGGCAACAGTTACATCACCAGTACCACC<br>ATTATCCACAAGAACGAACTGCGTAAAATTGCTCGCGGGACCCATAGATCCTTTCTCCTCTTTAGATCTTTTGAATTCCCAAAAAAACG<br>GGTATGGAGAAACAGTAGAGAGTTGCGATAAAAAGCGTCAGGTAGAATCCGCTAATCTTATGGATAAAAATGCTATGGCATAGCAAAG<br>TGTGACGCCGTGCAAATAATCAATGTGGACTTTTCTGCCGTGATTATAGACACTTTTGTTACGCGTTTTTGTCATGGCTTTGGTCCCGC<br>TTTGTTACAGAATGCTTTTAATAAGCGGGGTTACCGGTTTGGTTAGCGAGAAGAGCCAGTAAAAGACGCAGTGACGGCAATGTCTGAT<br>GCAATATGGACAATTGGTTTCTTCTCTGAATGGCGGGAGTATGAAAAGTTGAGATCCTTTTTTTCTGCGCGTAATCTGCTGCTTGCAAA<br>CAAAAAAACCACCGCTACCAGCGGTGGTTTGTTTGCCGGATCAAGAGCTACCAACTCTTTTTCCGAAGGTAACTGGCTTCAGCAGAGC<br>GCAGATACCAAATACTGTTCTTCTAGTGTAGCCGTAGTTAGGCCACCACTTCAAGAACTCTGTAGCACCGCCTACATACCTCGCTCTG<br>CTAATCCTGTTACCAGTGGCTGCTGCCAGTGGCGATAAGTCGTGTCTTACCGGGTTGGACTCAAGACGATAGTTACCGGATAAGGCG<br>CAGCGGTCGGGCTGAACGGGGGGTTCGTGCACACAGCCCAGCTTGGAGCGAACGACCTACACCGAACT	
pSJ107	GAGATACCTACAGCGTGAGCTATGAGAAAGCGCCACGCTTCCCGAAGGGAGAAAGGCGGACAGGTATCCGGTAAGCGGCAGGGTC<br>GGAACAGGAGAGCGCACGAGGGAGCTTCCAGGGGGAAACGCCTGGTATCTTTATAGTCCTGTCGGGTTTCGCCACCTCTGACTTGA<br>GCGTCGATTTTTGTGATGCTCGTCAGGGGGGCGGAGCCTATGGAAAAACGCCAGCAACGCGGCCTTTTTACGGTTCCTGGCCTTTTG<br>CTGGCCTTTTGCTCTCAGATAAAATATTTCTAGATTTCAGTGCAATTTATCTCTTCAAATGTAGCACCTGAAGTCAGCCCCATACGATAT<br>AAGTTGTTACTAGATTGACAGCTAGCTCAGTCCTAGGTATAATACTAGTCGGTGTCCTGCGGTTACCAAGTTTTAGAGCTAGAAATAGC<br>AAGTTAAAATAAGGCTAGTCCGTTATCAACTTGAAAAAGTGGCACATGAGGATCACCCATGTGCTTTTTTTGAAGCTTGGGCCCGAACA<br>AAAACTCATCTCAGAAGAGGATCTGAATAGCGCCGTCGACCATCATCATCATCATCATTGAGTTTAAACGGTCTCCAGCTTGGCTGTTT<br>TGGCGGATGAGAGAAGATTTTCAGCCTGATACAGATTAAATCAGAACGCAGAAGCGGTCTGATAAAACAGAATTTGCCTGGCGGCAGT<br>AGCGCGGTGGTCCCACCTGACCCCATGCCGAACTCAGAAGTGAAACGCCGTAGCGCCGATGGTAGTGTGGGGTCTCCCCATGCGAG<br>AGTAGGGAACTGCCAGGCATCAAATAAAACGAAAGGCTCAGTCGAAAGACTGGGCCTTTCGTTTTATCTGTTGTTTGTCGGTGAACTG<br>GATCCTTACTCGAGTCTAGACTGCAGGCTTCCTCGCTCACTGACTCGCTGCGCTCGGTCGTTCGGCTGCGGCGAGCGGTATCAGCTC<br>ACTCAAAGGCGGTAATACGGTTATCCACAGAATCAGGGGATAACGCAGGAAAGAACATGTGAGCAAAAGGCCAGCAAAAGGCCAGGA<br>ACCGTAAAAAACTAGTGCTTGGATTCTCACCAATAAAAAACGCCCGGCGGCAACCGAGCGTTCTGAACAAATCCAGATGGAGTTCTGA<br>GGTCATTACTGGATCTATCAACAGGAGTCCAAGCGAGCTCGATATCAAATTACGCCCCGCCCTGCCACTCATCGCAGTACTGTTGTAA<br>TTCATTAAGCATTCTGCCGACATGGAAGCCATCACAAACGGCATGATGAACCTGAATCGCCAGCCGAGACGAAAGGGCCTCGTGATA<br>CGCCTATTTTTATAGGTTAATGTCATGATAATAATGGTTTCTTACCTCCCGTGGGAAAAAATCATGGCAATTCTGGAAGAAATAGCGC<br>TTTCAGCCGGCAAACCGGCTGAAGCCGGATCTGCGATTCTGATAACAAACTAGCAACACCAGAACAGCCCGTTTGCGGGCAGCAAAA<br>CCCGTACCCTAGGTATAAACGCAGAAAGGCCCACCCGAAGGTGAGCCAGTGTGACTCTAGTAGAGAGCGTTCACCGACAAACAACAG<br>ATAAAACGAAAGGCCCAGTCTTTCGACTGAGCCTTTCGTTTTATTTGATGCCTGGAGATCCTTACTCGAGTTTGGATCCTTAAGCACCG<br>GTGGAGTGACGACCTTCAGCACGTTCGTACTGTTCAACGATGGTGTAGTCTTCGTTGTGGGAGGTGATGTCCAGTTTGATGTCGGTTT<br>TGTAAGCACCCGGCAGCTGAACCGGTTTTTTAGCCATGTAGGTGGTTTTAACTTCAGCGTCGTAGTGACCACCGTCTTTCAGTTTCAG<br>ACGCATTTTGATTTCACCTTTCAGAGCACCGTCTTCCGGGTACATACGTTCGGTGGAAGCTTCCCAACCCATGGTTTTTTTCTGCATAA<br>CCGGACCGTCGGACGGGAAGTTGGTACCACGCAGTTTAACTTTGTAGATGAACTCACCGTCTTGCAGGGAGGAGTCCTGGGTAACG<br>GTAACAACACCACCGTCTTCGAAGTTCATAACACGTTCCCATTTGAAACCTTCCGGGAAGGACAGTTTCAGGTAGTCCGGGATGTCAG<br>CCGGGTGTTTAACGTAAGCTTTGGAACCGTACTGGAACTGCGGGGACAGGATGTCCCAAGCGAACGGCAGCGGACCACCTTTGGTA<br>ACTTTCAGTTTAGCGGTCTGGGTACCTTCGTACGGACGACCTTCACCTTCACCTTCGATTTCGAACTCGTGACCGTTAACGGAACCTT<br>CCATACGAACTTTGAAACGCATGAACTCTTTGATAACGTCTTCGCTACTCGCCATGGTACCTTTCTCCTCTTTAATGAATTCGCTAGCA<br>CAATCCCTAGGACTGAGCTAGCTGTCAAGTCCGGAGGTATCCGGTGGAACCCAAAGGACGCCTTTGGTAACCGCAGGACACCGCAG<br>GATACCTGAGGTCGCCCGTGGTGGCCCATGGTCACCATAGGTCACCCTTGGCAACCAAAGGTGTCCTATGGCGGCCGGAGGCTGCC<br>ATAGGTCTCCGGTGGATACCGTAGGCGACGTCATCTAGATGCATTCGCGAGGTACCGAGCTCGAATTCACTGGCCGTCGTTTTACAA<br>CGTCGTGACTGGGAAAACCCTGGCGTTACCCAACTTAATCGCCTTGCAGCACATCCCCCTTTCGCCAGCTGGCGTAATAGCGAAGAG<br>GCCCGCACCGATCGCCCTTCCCAACAGTTGCGCAGCCTGAATGGCGAATGGCGCCTGATGCGGTATTTTCTCCTTACGCATCTGTGC<br>GGTATTTCACACCGCATATGGTGCACTCTCAGTACAATCTGCTCTGATGCCGCATAGTTAAGCCAGCCCCGACACCCGCCAACACCC<br>GCTGACGCGCCCTGACGGGCTTGTCTGCTCCCGGCATCCGCTTACAGACAAGCTGTGACCGTCTCCGGGAGCTGCATGTGTCAGAG<br>GTTTTCACCGTCATCACCGAAACGCGCGAGACGAAAGGGCCTCGTGATACGCCTATTTTTATAGGTTAATGTCATGATAATAATGGTTT<br>CTTAGACGTCAGGTGGCACTTTTCGGGGAAATGTGCGCGGAACCCCTATTTGTTTATTTTTCTAAATACATTCAAATATGTATCCGCTC<br>ATGAGACAATAACCCTGATAAATGCTTCAATAATATTGAAAAAGGAAGAGTATGAGTATTCAACATTTCCGTGTCGCCCTTATTCCCTTT	This study

	TTTGCGGCATTTTGCCTTCCTGTTTTTGCTCACCCAGAAACGCTGGTGAAAGTAAAAGATGCTGAAGATCAGTTGGGTGCACGAGTGG<br>GTTACATCGAACTGGATCTCAACAGCGGTAAGATCCTTGAGAGTTTTCGCCCCGAAGAACGTTTTCCAATGATGAGCACTTTTAAAGTT<br>CTGCTATGTGGCGCGGTATTATCCCGTATTGACGCCGGGCAAGAGCAACTCGGTCGCCGCATACACTATTCTCAGAATGACTTGGTT<br>GAGTACTCACCAGTCACAGAAAAGCATCTTACGGATGGCATGACAGTAAGAGAATTATGCAGTGCTGCCATAACCATGAGTGATAACA<br>CTGCGGCCAACTTACTTCTGACAACGATCGGAGGACCGAAGGAGCTAACCGCTTTTTTGCACAACATGGGGGATCATGTAACTCGCC<br>TTGATCGTTGGGAACCGGAGCTGAATGAAGCCATACCAAACGACGAGCGTGACACCACGATGCCTGTAGCAATGGCAACAACGTTGC<br>GCAAACTATTAACTGGCGAACTACTTACTCTAGCTTCCCGGCAACAATTAATAGACTGGATGGAGGCGGATAAAGTTGCAGGACCACT<br>TCTGCGCTCGGCCCTTCCGGCTGGCTGGTTTATTGCTGATAAATCTGGAGCCGGTGAGCGTGGGTCTCGCGGTATCATTGCAGCACT<br>GGGGCCAGATGGTAAGCCCTCCCGTATCGTAGTTATCTACACGACGGGGAGTCAGGCAACTATGGATGAACGAAATAGACAGATCGC<br>TGAGATAGGTGCCTCACTGATTAAGCATTGGTAACTGTCAGACCAAGTTTACTCATATATACTTTAGATTGATTTAAAACTTCATTTTTAA<br>TTTAAAAGGATCTAGGTGAAGATCCTTTTTGATAATCTCATGACCAAAATCCCTTAACGTGAGTTTTCGTTCCACTGAGCGTCAGACCC<br>CGTAGAAAAGATCAAAGGATCTTCAAGCAGCATAACCTTTTTCCGTGATGGTAACTTCACGGTAACCAAGATGTCGAGTTAACCACCCT<br>TTAGATTCATAAAGCGAAAATAATGCGGCTCCAACGTACCCACCTAAATGGAAACGGCGTTCACTCCAAGCGAAAAAACCCCGCCGAA<br>GCGGGGTTTTTTGCGTTAAAGCCGGTGACGGTAGTCGCTGGGAGTCCGGTCAAATTGACGAGCGAAGACGCGAGAGAAGGTTTGCT<br>GCGACACATAGCCTAAGTCCATGGCAATATCGAAAATCGGACGTTCTGTTGTCCGCAATTCAACGGCAGCCAACAACAAACGGCGTT<br>GACGGATATAGTCTCCTAATGTTTGATGTGTGACGGTACGAAACATGCGCTGTAAATACCACTTCGAGTAACCACTTTTCTTCGCTACA<br>ACATCAATATTAAGAGGCTGATCAATATGCTCATCGATCCAAGCAATAAGGTCCTGGATGATTTTTTGGTGGCTCATTGAACCACCTCC<br>GCCGTAAATACCACTGTTGGCCGCGATGGCAGATGGAATCGGATTTCCATCTTTCAGAAGACCCTGCATCGCTTTCACAATCAACTCA<br>CAGTCGCTATTCGTAGCAAAGATCGGAATAGTCAGTTCCATATTAAGGTACGAACGCCATGCACCTTTAGGGACTTCAACTTTGATCGT<br>GTACTTACGGTTTTGCGCACTCGATTGACGTACAGAACAAGTAACTTTGTATGCTTGGCTACGGCTGTTGGAGGAAATCCACTCGGCA<br>ATGCCGTTAGCGAAGTTGCTAGGGGCAACAGTTACATCACCAGTACCACCATTATCCACAAGAACGAACTGCGTAAAATTGCTCGCGG<br>GACCCATAGATCCTTTCTCCTCTTTAGATCTTTTGAATTCCCAAAAAAACGGGTATGGAGAAACAGTAGAGAGTTGCGATAAAAAGCGT<br>CAGGTAGAATCCGCTAATCTTATGGATAAAAATGCTATGGCATAGCAAAGTGTGACGCCGTGCAAATAATCAATGTGGACTTTTCTGCC<br>GTGATTATAGACACTTTTGTTACGCGTTTTTGTCATGGCTTTGGTCCCGCTTTGTTACAGAATGCTTTTAATAAGCGGGGTTACCGGTTT<br>GGTTAGCGAGAAGAGCCAGTAAAAGACGCAGTGACGGCAATGTCTGATGCAATATGGACAATTGGTTTCTTCTCTGAATGGCGGGAG<br>TATGAAAAGTTGAGATCCTTTTTTTCTGCGCGTAATCTGCTGCTTGCAAACAAAAAAACCACCGCTACCAGCGGTGGTTTGTTTGCCGG<br>ATCAAGAGCTACCAACTCTTTTTCCGAAGGTAACTGGCTTCAGCAGAGCGCAGATACCAAATACTGTTCTTCTAGTGTAGCCGTAGTTA<br>GGCCACCACTTCAAGAACTCTGTAGCACCGCCTACATACCTCGCTCTGCTAATCCTGTTACCAGTGGCTGCTGCCAGTGGCGATAAGT<br>CGTGTCTTACCGGGTTGGACTCAAGACGATAGTTACCGGATAAGGCGCAGCGGTCGGGCTGAACGGGGGGTTCGTGCACACAGCCC<br>AGCTTGGAGCGAACGACCTACACCGAACT	
pUC57_<br>107_2x	TCGCGCGTTTCGGTGATGACGGTGAAAACCTCTGACACATGCAGCTCCCGGAGACGGTCACAGCTTGTCTGTAAGCGGATGCCGGG<br>AGCAGACAAGCCCGTCAGGGCGCGTCAGCGGGTGTTGGCGGGTGTCGGGGCTGGCTTAACTATGCGGCATCAGAGCAGATTGTACT<br>GAGAGTGCACCATATGCGGTGTGAAATACCGCACAGATGCGTAAGGAGAAAATACCGCATCAGGCGCCATTCGCCATTCAGGCTGCG<br>CAACTGTTGGGAAGGGCGATCGGTGCGGGCCTCTTCGCTATTACGCCAGCTGGCGAAAGGGGGATGTGCTGCAAGGCGATTAAGTT<br>GGGTAACGCCAGGGTTTTCCCAGTCACGACGTTGTAAAACGACGGCCAGTGAATTCGAGCTCGGTACCTCGCGAATGCATCTAGATG<br>ACGTCGCCTACGGTATCCACCGGAGACCTATGGCAGCCTCCGGCCGCCATAGGACACCTTTGGTTGCCAAGGGTGACCTATGGTGA<br>CCATGGGCCACCACCGGTGTCCTGCGGTTACCAACGGTGTCCTGCGGTTACCAAAGGCGTCCTGCGGTTACCAAAGGCGTCCTTTG<br>GGTTCCACCGGATACCTCCGGACTTGACAGCTAGCTCAGTCCTAGGGATTGTGCTAGCGAATTCATTAAAGAGGAGAAAGGTACCAT<br>GGCGAGTAGCGAAGACGTTATCAAAGAGTTCATGCGTTTCAAAGTTCGTATGGAAGGTTCCGTTAACGGTCACGAGTTCGAAATCGAA<br>GGTGAAGGTGAAGGTCGTCCGTACGAAGGTACCCAGACCGCTAAACTGAAAGTTACCAAAGGTGGTCCGCTGCCGTTCGCTTGGGA<br>CATCCTGTCCCCGCAGTTCCAGTACGGTTCCAAAGCTTACGTTAAACACCCGGCTGACATCCCGGACTACCTGAAACTGTCCTTCCCG<br>GAAGGTTTCAAATGGGAACGTGTTATGAACTTCGAAGACGGTGGTGTTGTTACCGTTACCCAGGACTCCTCCCTGCAAGACGGTGAG<br>TTCATCTACAAAGTTAAACTGCGTGGTACCAACTTCCCGTCCGACGGTCCGGTTATGCAGAAAAAAACCATGGGTTGGGAAGCTTCCA<br>CCGAACGTATGTACCCGGAAGACGGTGCTCTGAAAGGTGAAATCAAAATGCGTCTGAAACTGAAAGACGGTGGTCACTACGACGCTG<br>AAGTTAAAACCACCTACATGGCTAAAAAACCGGTTCAGCTGCCGGGTGCTTACAAAACCGACATCAAACTGGACATCACCTCCCACAA<br>CGAAGACTACACCATCGTTGAACAGTACGAACGTGCTGAAGGTCGTCACTCCACCGGTGCTTAAGGATCCAAACTCGAGTAAGGATC<br>TCCAGGCATCAAATAAAACGAAAGGCTCAGTCGAAAGACTGGGCCTTTCGTTTTATCTGTTGTTTGTCGGTGAACGCTCTCTACTAGA<br>GTCACACTGGCTCACCTTCGGGTGGGCCTTTCTGCGTTTATACCTAGGATCGGATCCCGGGCCCGTCGACTGCAGAGGCCTGCATG<br>CAAGCTTGGCGTAATCATGGTCATAGCTGTTTCCTGTGTGAAATTGTTATCCGCTCACAATTCCACACAACATACGAGCCGGAAGCATA<br>AAGTGTAAAGCCTGGGGTGCCTAATGAGTGAGCTAACTCACATTAATTGCGTTGCGCTCACTGCCCGCTTTCCAGTCGGGAAACCTGT<br>CGTGCCAGCTGCATTAATGAATCGGCCAACGCGCGGGGAGAGGCGGTTTGCGTATTGGGCGCTCTTCCGCTTCCTCGCTCACTGAC<br>TCGCTGCGCTCGGTCGTTCGGCTGCGGCGAGCGGTATCAGCTCACTCAAAGGCGGTAATACGGTTATCCACAGAATCAGGGGATAA<br>CGCAGGAAAGAACATGTGAGCAAAAGGCCAGCAAAAGGCCAGGAACCGTAAAAAGGCCGCGTTGCTGGCGTTTTTCCATAGGCTCC<br>GCCCCCCTGACGAGCATCACAAAAATCGACGCTCAAGTCAGAGGTGGCGAAACCCGACAGGACTATAAAGATACCAGGCGTTTCCCC<br>CTGGAAGCTCCCTCGTGCGCTCTCCTGTTCCGACCCTGCCGCTTACCGGATACCTGTCCGCCTTTCTCCCTTCGGGAAGCGTGGCGC<br>TTTCTCATAGCTCACGCTGTAGGTATCTCAGTTCGGTGTAGGTCGTTCGCTCCAAGCTGGGCTGTGTGCACGAACCCCCCGTTCAGC<br>CCGACCGCTGCGCCTTATCCGGTAACTATCGTCTTGAGTCCAACCCGGTAAGACACGACTTATCGCCACTGGCAGCAGCCACTGGTA	BioCat

	ACAGGATTAGCAGAGCGAGGTATGTAGGCGGTGCTACAGAGTTCTTGAAGTGGTGGCCTAACTACGGCTACACTAGAAGAACAGTAT<br>TTGGTATCTGCGCTCTGCTGAAGCCAGTTACCTTCGGAAAAAGAGTTGGTAGCTCTTGATCCGGCAAACAAACCACCGCTGGTAGCG<br>GTGGTTTTTTTGTTTGCAAGCAGCAGATTACGCGCAGAAAAAAAGGATCTCAAGAAGATCCTTTGATCTTTTCTACGGGGTCTGACGCT<br>CAGTGGAACGAAAACTCACGTTAAGGGATTTTGGTCATGAGATTATCAAAAAGGATCTTCACCTAGATCCTTTTAAATTAAAAATGAAGT<br>TTTAAATCAATCTAAAGTATATATGAGTAAACTTGGTCTGACAGTTACCAATGCTTAATCAGTGAGGCACCTATCTCAGCGATCTGTCTA<br>TTTCGTTCATCCATAGTTGCCTGACTCCCCGTCGTGTAGATAACTACGATACGGGAGGGCTTACCATCTGGCCCCAGTGCTGCAATGA<br>TACCGCGAGACTCACGCTCACCGGCTCCAGATTTATCAGCAATAAACCAGCCAGCCGGAAGGGCCGAGCGCAGAAGTGGTCCTGCA<br>ACTTTATCCGCCTCCATCCAGTCTATTAATTGTTGCCGGGAAGCTAGAGTAAGTAGTTCGCCAGTTAATAGTTTGCGCAACGTTGTTGC<br>CATTGCTACAGGCATCGTGGTGTCACGCTCGTCGTTTGGTATGGCTTCATTCAGCTCCGGTTCCCAACGATCAAGGCGAGTTACATGA<br>TCCCCCATGTTGTGCAAAAAAGCGGTTAGCTCCTTCGGTCCTCCGATCGTTGTCAGAAGTAAGTTGGCCGCAGTGTTATCACTCATGG<br>TTATGGCAGCACTGCATAATTCTCTTACTGTCATGCCATCCGTAAGATGCTTTTCTGTGACTGGTGAGTACTCAACCAAGTCATTCTGA<br>GAATAGTGTATGCGGCGACCGAGTTGCTCTTGCCCGGCGTCAATACGGGATAATACCGCGCCACATAGCAGAACTTTAAAAGTGCTC<br>ATCATTGGAAAACGTTCTTCGGGGCGAAAACTCTCAAGGATCTTACCGCTGTTGAGATCCAGTTCGATGTAACCCACTCGTGCACCCA<br>ACTGATCTTCAGCATCTTTTACTTTCACCAGCGTTTCTGGGTGAGCAAAAACAGGAAGGCAAAATGCCGCAAAAAAGGGAATAAGGGC<br>GACACGGAAATGTTGAATACTCATACTCTTCCTTTTTCAATATTATTGAAGCATTTATCAGGGTTATTGTCTCATGAGCGGATACATATTT<br>GAATGTATTTAGAAAAATAAACAAATAGGGGTTCCGCGCACATTTCCCCGAAAAGTGCCACCTGACGTCTAAGAAACCATTATTATCAT<br>GACATTAACCTATAAAAATAGGCGTATCACGAGGCCCTTTCGTC	
pSJ107_<br>2x	GAGATACCTACAGCGTGAGCTATGAGAAAGCGCCACGCTTCCCGAAGGGAGAAAGGCGGACAGGTATCCGGTAAGCGGCAGGGTC<br>GGAACAGGAGAGCGCACGAGGGAGCTTCCAGGGGGAAACGCCTGGTATCTTTATAGTCCTGTCGGGTTTCGCCACCTCTGACTTGA<br>GCGTCGATTTTTGTGATGCTCGTCAGGGGGGCGGAGCCTATGGAAAAACGCCAGCAACGCGGCCTTTTTACGGTTCCTGGCCTTTTG<br>CTGGCCTTTTGCTCTCAGATAAAATATTTCTAGATTTCAGTGCAATTTATCTCTTCAAATGTAGCACCTGAAGTCAGCCCCATACGATAT<br>AAGTTGTTACTAGATTGACAGCTAGCTCAGTCCTAGGTATAATACTAGTTGGTAACCGCAGGACACCGCGTTTTAGAGCTAGAAATAG<br>CAAGTTAAAATAAGGCTAGTCCGTTATCAACTTGAAAAAGTGGCACATGAGGATCACCCATGTGCTTTTTTTGAAGCTTGGGCCCGAAC<br>AAAAACTCATCTCAGAAGAGGATCTGAATAGCGCCGTCGACCATCATCATCATCATCATTGAGTTTAAACGGTCTCCAGCTTGGCTGTT<br>TTGGCGGATGAGAGAAGATTTTCAGCCTGATACAGATTAAATCAGAACGCAGAAGCGGTCTGATAAAACAGAATTTGCCTGGCGGCAG<br>TAGCGCGGTGGTCCCACCTGACCCCATGCCGAACTCAGAAGTGAAACGCCGTAGCGCCGATGGTAGTGTGGGGTCTCCCCATGCGA<br>GAGTAGGGAACTGCCAGGCATCAAATAAAACGAAAGGCTCAGTCGAAAGACTGGGCCTTTCGTTTTATCTGTTGTTTGTCGGTGAACT<br>GGATCCTTACTCGAGTCTAGACTGCAGGCTTCCTCGCTCACTGACTCGCTGCGCTCGGTCGTTCGGCTGCGGCGAGCGGTATCAGCT<br>CACTCAAAGGCGGTAATACGGTTATCCACAGAATCAGGGGATAACGCAGGAAAGAACATGTGAGCAAAAGGCCAGCAAAAGGCCAGG<br>AACCGTAAAAAACTAGTGCTTGGATTCTCACCAATAAAAAACGCCCGGCGGCAACCGAGCGTTCTGAACAAATCCAGATGGAGTTCTG<br>AGGTCATTACTGGATCTATCAACAGGAGTCCAAGCGAGCTCGATATCAAATTACGCCCCGCCCTGCCACTCATCGCAGTACTGTTGTA<br>ATTCATTAAGCATTCTGCCGACATGGAAGCCATCACAAACGGCATGATGAACCTGAATCGCCAGCCGAGACGAAAGGGCCTCGTGAT<br>ACGCCTATTTTTATAGGTTAATGTCATGATAATAATGGTTTCTTACCTAGGTATAAACGCAGAAAGGCCCACCCGAAGGTGAGCCAGTG<br>TGACTCTAGTAGAGAGCGTTCACCGACAAACAACAGATAAAACGAAAGGCCCAGTCTTTCGACTGAGCCTTTCGTTTTATTTGATGCCT<br>GGAGATCCTTACTCGAGTTTGGATCCTTAAGCACCGGTGGAGTGACGACCTTCAGCACGTTCGTACTGTTCAACGATGGTGTAGTCTT<br>CGTTGTGGGAGGTGATGTCCAGTTTGATGTCGGTTTTGTAAGCACCCGGCAGCTGAACCGGTTTTTTAGCCATGTAGGTGGTTTTAAC<br>TTCAGCGTCGTAGTGACCACCGTCTTTCAGTTTCAGACGCATTTTGATTTCACCTTTCAGAGCACCGTCTTCCGGGTACATACGTTCGG<br>TGGAAGCTTCCCAACCCATGGTTTTTTTCTGCATAACCGGACCGTCGGACGGGAAGTTGGTACCACGCAGTTTAACTTTGTAGATGAA<br>CTCACCGTCTTGCAGGGAGGAGTCCTGGGTAACGGTAACAACACCACCGTCTTCGAAGTTCATAACACGTTCCCATTTGAAACCTTCC<br>GGGAAGGACAGTTTCAGGTAGTCCGGGATGTCAGCCGGGTGTTTAACGTAAGCTTTGGAACCGTACTGGAACTGCGGGGACAGGAT<br>GTCCCAAGCGAACGGCAGCGGACCACCTTTGGTAACTTTCAGTTTAGCGGTCTGGGTACCTTCGTACGGACGACCTTCACCTTCACC<br>TTCGATTTCGAACTCGTGACCGTTAACGGAACCTTCCATACGAACTTTGAAACGCATGAACTCTTTGATAACGTCTTCGCTACTCGCCA<br>TGGTACCTTTCTCCTCTTTAATGAATTCGCTAGCACAATCCCTAGGACTGAGCTAGCTGTCAAGTCCGGAGGTATCCGGTGGAACCCA<br>AAGGACGCCTTTGGTAACCGCAGGACACCGTTGGTAACCGCAGGACACCGGTGGTGGCCCATGGTCACCATAGGTCACCCTTGGCA<br>ACCAAAGGTGTCCTATGGCGGCCGGAGGCTGCCATAGGTCTCCGGTGGATACCGTAGGCGACGTCATCTAGATGCATTCGCGAGGT<br>ACCGAGCTCGAATTCACTGGCCGTCGTTTTACAACGTCGTGACTGGGAAAACCCTGGCGTTACCCAACTTAATCGCCTTGCAGCACAT<br>CCCCCTTTCGCCAGCTGGCGTAATAGCGAAGAGGCCCGCACCGATCGCCCTTCCCAACAGTTGCGCAGCCTGAATGGCGAATGGCG<br>CCTGATGCGGTATTTTCTCCTTACGCATCTGTGCGGTATTTCACACCGCATATGGTGCACTCTCAGTACAATCTGCTCTGATGCCGCAT<br>AGTTAAGCCAGCCCCGACACCCGCCAACACCCGCTGACGCGCCCTGACGGGCTTGTCTGCTCCCGGCATCCGCTTACAGACAAGCT<br>GTGACCGTCTCCGGGAGCTGCATGTGTCAGAGGTTTTCACCGTCATCACCGAAACGCGCGAGACGAAAGGGCCTCGTGATACGCCT<br>ATTTTTATAGGTTAATGTCATGATAATAATGGTTTCTTAGACGTCAGGTGGCACTTTTCGGGGAAATGTGCGCGGAACCCCTATTTGTT<br>TATTTTTCTAAATACATTCAAATATGTATCCGCTCATGAGACAATAACCCTGATAAATGCTTCAATAATATTGAAAAAGGAAGAGTATGA<br>GTATTCAACATTTCCGTGTCGCCCTTATTCCCTTTTTTGCGGCATTTTGCCTTCCTGTTTTTGCTCACCCAGAAACGCTGGTGAAAGTAA<br>AAGATGCTGAAGATCAGTTGGGTGCACGAGTGGGTTACATCGAACTGGATCTCAACAGCGGTAAGATCCTTGAGAGTTTTCGCCCCG<br>AAGAACGTTTTCCAATGATGAGCACTTTTAAAGTTCTGCTATGTGGCGCGGTATTATCCCGTATTGACGCCGGGCAAGAGCAACTCGG<br>TCGCCGCATACACTATTCTCAGAATGACTTGGTTGAGTACTCACCAGTCACAGAAAAGCATCTTACGGATGGCATGACAGTAAGAGAA<br>TTATGCAGTGCTGCCATAACCATGAGTGATAACACTGCGGCCAACTTACTTCTGACAACGATCGGAGGACCGAAGGAGCTAACCGCTT	This<br>study

	TTTTGCACAACATGGGGGATCATGTAACTCGCCTTGATCGTTGGGAACCGGAGCTGAATGAAGCCATACCAAACGACGAGCGTGACA<br>CCACGATGCCTGTAGCAATGGCAACAACGTTGCGCAAACTATTAACTGGCGAACTACTTACTCTAGCTTCCCGGCAACAATTAATAGA<br>CTGGATGGAGGCGGATAAAGTTGCAGGACCACTTCTGCGCTCGGCCCTTCCGGCTGGCTGGTTTATTGCTGATAAATCTGGAGCCGG<br>TGAGCGTGGGTCTCGCGGTATCATTGCAGCACTGGGGCCAGATGGTAAGCCCTCCCGTATCGTAGTTATCTACACGACGGGGAGTCA<br>GGCAACTATGGATGAACGAAATAGACAGATCGCTGAGATAGGTGCCTCACTGATTAAGCATTGGTAACTGTCAGACCAAGTTTACTCA<br>TATATACTTTAGATTGATTTAAAACTTCATTTTTAATTTAAAAGGATCTAGGTGAAGATCCTTTTTGATAATCTCATGACCAAAATCCCTTA<br>ACGTGAGTTTTCGTTCCACTGAGCGTCAGACCCCGTAGAAAAGATCAAAGGATCTTCAAGCAGCATAACCTTTTTCCGTGATGGTAAC<br>TTCACGGTAACCAAGATGTCGAGTTAACCACCCTTTAGATTCATAAAGCGAAAATAATGCGGCTCCAACGTACCCACCTAAATGGAAAC<br>GGCGTTCACTCCAAGCGAAAAAACCCCGCCGAAGCGGGGTTTTTTGCGTTAAAGCCGGTGACGGTAGTCGCTGGGAGTCCGGTCAA<br>ATTGACGAGCGAAGACGCGAGAGAAGGTTTGCTGCGACACATAGCCTAAGTCCATGGCAATATCGAAAATCGGACGTTCTGTTGTCC<br>GCAATTCAACGGCAGCCAACAACAAACGGCGTTGACGGATATAGTCTCCTAATGTTTGATGTGTGACGGTACGAAACATGCGCTGTAA<br>ATACCACTTCGAGTAACCACTTTTCTTCGCTACAACATCAATATTAAGAGGCTGATCAATATGCTCATCGATCCAAGCAATAAGGTCCT<br>GGATGATTTTTTGGTGGCTCATTGAACCACCTCCGCCGTAAATACCACTGTTGGCCGCGATGGCAGATGGAATCGGATTTCCATCTTT<br>CAGAAGACCCTGCATCGCTTTCACAATCAACTCACAGTCGCTATTCGTAGCAAAGATCGGAATAGTCAGTTCCATATTAAGGTACGAAC<br>GCCATGCACCTTTAGGGACTTCAACTTTGATCGTGTACTTACGGTTTTGCGCACTCGATTGACGTACAGAACAAGTAACTTTGTATGCT<br>TGGCTACGGCTGTTGGAGGAAATCCACTCGGCAATGCCGTTAGCGAAGTTGCTAGGGGCAACAGTTACATCACCAGTACCACCATTA<br>TCCACAAGAACGAACTGCGTAAAATTGCTCGCGGGACCCATAGATCCTTTCTCCTCTTTAGATCTTTTGAATTCCCAAAAAAACGGGTA<br>TGGAGAAACAGTAGAGAGTTGCGATAAAAAGCGTCAGGTAGAATCCGCTAATCTTATGGATAAAAATGCTATGGCATAGCAAAGTGTG<br>ACGCCGTGCAAATAATCAATGTGGACTTTTCTGCCGTGATTATAGACACTTTTGTTACGCGTTTTTGTCATGGCTTTGGTCCCGCTTTG<br>TTACAGAATGCTTTTAATAAGCGGGGTTACCGGTTTGGTTAGCGAGAAGAGCCAGTAAAAGACGCAGTGACGGCAATGTCTGATGCAA<br>TATGGACAATTGGTTTCTTCTCTGAATGGCGGGAGTATGAAAAGTTGAGATCCTTTTTTTCTGCGCGTAATCTGCTGCTTGCAAACAAA<br>AAAACCACCGCTACCAGCGGTGGTTTGTTTGCCGGATCAAGAGCTACCAACTCTTTTTCCGAAGGTAACTGGCTTCAGCAGAGCGCA<br>GATACCAAATACTGTTCTTCTAGTGTAGCCGTAGTTAGGCCACCACTTCAAGAACTCTGTAGCACCGCCTACATACCTCGCTCTGCTAA<br>TCCTGTTACCAGTGGCTGCTGCCAGTGGCGATAAGTCGTGTCTTACCGGGTTGGACTCAAGACGATAGTTACCGGATAAGGCGCAGC<br>GGTCGGGCTGAACGGGGGGTTCGTGCACACAGCCCAGCTTGGAGCGAACGACCTACACCGAACT	
p108	GAGATACCTACAGCGTGAGCTATGAGAAAGCGCCACGCTTCCCGAAGGGAGAAAGGCGGACAGGTATCCGGTAAGCGGCAGGGTC<br>GGAACAGGAGAGCGCACGAGGGAGCTTCCAGGGGGAAACGCCTGGTATCTTTATAGTCCTGTCGGGTTTCGCCACCTCTGACTTGA<br>GCGTCGATTTTTGTGATGCTCGTCAGGGGGGCGGAGCCTATGGAAAAACGCCAGCAACGCGGCCTTTTTACGGTTCCTGGCCTTTTG<br>CTGGCCTTTTGCTCTCAGATAAAATATTTCTAGATTTCAGTGCAATTTATCTCTTCAAATGTAGCACCTGAAGTCAGCCCCATACGATAT<br>AAGTTGTTACTAGATTGACAGCTAGCTCAGTCCTAGGTATAATACTAGTTGGTAACCGCAGGACACCGCGTTTTAGAGCTAGAAATAG<br>CAAGTTAAAATAAGGCTAGTCCGTTATCAACTTGAAAAAGTGGCACATGAGGATCACCCATGTGCTTTTTTTGAAGCTTGGGCCCGAAC<br>AAAAACTCATCTCAGAAGAGGATCTGAATAGCGCCGTCGACCATCATCATCATCATCATTGAGTTTAAACGGTCTCCAGCTTGGCTGTT<br>TTGGCGGATGAGAGAAGATTTTCAGCCTGATACAGATTAAATCAGAACGCAGAAGCGGTCTGATAAAACAGAATTTGCCTGGCGGCAG<br>TAGCGCGGTGGTCCCACCTGACCCCATGCCGAACTCAGAAGTGAAACGCCGTAGCGCCGATGGTAGTGTGGGGTCTCCCCATGCGA<br>GAGTAGGGAACTGCCAGGCATCAAATAAAACGAAAGGCTCAGTCGAAAGACTGGGCCTTTCGTTTTATCTGTTGTTTGTCGGTGAACT<br>GGATCCTTACTCGAGTCTAGACTGCAGGCTTCCTCGCTCACTGACTCGCTGCGCTCGGTCGTTCGGCTGCGGCGAGCGGTATCAGCT<br>CACTCAAAGGCGGTAATACGGTTATCCACAGAATCAGGGGATAACGCAGGAAAGAACATGTGAGCAAAAGGCCAGCAAAAGGCCAGG<br>AACCGTAAAAAACTAGTGCTTGGATTCTCACCAATAAAAAACGCCCGGCGGCAACCGAGCGTTCTGAACAAATCCAGATGGAGTTCTG<br>AGGTCATTACTGGATCTATCAACAGGAGTCCAAGCGAGCTCGATATCAAATTACGCCCCGCCCTGCCACTCATCGCAGTACTGTTGTA<br>ATTCATTAAGCATTCTGCCGACATGGAAGCCATCACAAACGGCATGATGAACCTGAATCGCCAGCCGAGACGAAAGGGCCTCGTGAT<br>ACGCCTATTTTTATAGGTTAATGTCATGATAATAATGGTTTCTTAGACGTCAGGTGGCACTTTTCGGGGAAATGTGCGCGGAACCCCTA<br>TTTGTTTATTTTTCTAAATACATTCAAATATGTATCCGCTCATGAGACAATAACCCTGATAAATGCTTCAATAATATTGAAAAAGGAAGAG<br>TATGAGTATTCAACATTTCCGTGTCGCCCTTATTCCCTTTTTTGCGGCATTTTGCCTTCCTGTTTTTGCTCACCCAGAAACGCTGGTGAA<br>AGTAAAAGATGCTGAAGATCAGTTGGGTGCACGAGTGGGTTACATCGAACTGGATCTCAACAGCGGTAAGATCCTTGAGAGTTTTCGC<br>CCCGAAGAACGTTTTCCAATGATGAGCACTTTTAAAGTTCTGCTATGTGGCGCGGTATTATCCCGTATTGACGCCGGGCAAGAGCAAC<br>TCGGTCGCCGCATACACTATTCTCAGAATGACTTGGTTGAGTACTCACCAGTCACAGAAAAGCATCTTACGGATGGCATGACAGTAAG<br>AGAATTATGCAGTGCTGCCATAACCATGAGTGATAACACTGCGGCCAACTTACTTCTGACAACGATCGGAGGACCGAAGGAGCTAAC<br>CGCTTTTTTGCACAACATGGGGGATCATGTAACTCGCCTTGATCGTTGGGAACCGGAGCTGAATGAAGCCATACCAAACGACGAGCG<br>TGACACCACGATGCCTGTAGCAATGGCAACAACGTTGCGCAAACTATTAACTGGCGAACTACTTACTCTAGCTTCCCGGCAACAATTA<br>ATAGACTGGATGGAGGCGGATAAAGTTGCAGGACCACTTCTGCGCTCGGCCCTTCCGGCTGGCTGGTTTATTGCTGATAAATCTGGA<br>GCCGGTGAGCGTGGGTCTCGCGGTATCATTGCAGCACTGGGGCCAGATGGTAAGCCCTCCCGTATCGTAGTTATCTACACGACGGG<br>GAGTCAGGCAACTATGGATGAACGAAATAGACAGATCGCTGAGATAGGTGCCTCACTGATTAAGCATTGGTAACTGTCAGACCAAGTT<br>TACTCATATATACTTTAGATTGATTTAAAACTTCATTTTTAATTTAAAAGGATCTAGGTGAAGATCCTTTTTGATAATCTCATGACCAAAAT<br>CCCTTAACGTGAGTTTTCGTTCCACTGAGCGTCAGACCCCGTAGAAAAGATCAAAGGATCTTCTTGAGATCCTTTTTTTCTGCGCGTAA<br>TCTGCTGCTTGCAAACAAAAAAACCACCGCTACCAGCGGTGGTTTGTTTGCCGGATCAAGAGCTACCAACTCTTTTTCCGAAGGTAAC<br>TGGCTTCAGCAGAGCGCAGATACCAAATACTGTTCTTCTAGTGTAGCCGTAGTTAGGCCACCACTTCAAGAACTCTGTAGCACCGCCT	This study

	ACATACCTCGCTCTGCTAATCCTGTTACCAGTGGCTGCTGCCAGTGGCGATAAGTCGTGTCTTACCGGGTTGGACTCAAGACGATAGT<br>TACCGGATAAGGCGCAGCGGTCGGGCTGAACGGGGGGTTCGTGCACACAGCCCAGCTTGGAGCGAACGACCTACACCGAACT	
pS108	GAGATACCTACAGCGTGAGCTATGAGAAAGCGCCACGCTTCCCGAAGGGAGAAAGGCGGACAGGTATCCGGTAAGCGGCAGGGTC<br>GGAACAGGAGAGCGCACGAGGGAGCTTCCAGGGGGAAACGCCTGGTATCTTTATAGTCCTGTCGGGTTTCGCCACCTCTGACTTGA<br>GCGTCGATTTTTGTGATGCTCGTCAGGGGGGCGGAGCCTATGGAAAAACGCCAGCAACGCGGCCTTTTTACGGTTCCTGGCCTTTTG<br>CTGGCCTTTTGCTCTCAGATAAAATATTTCTAGATTTCAGTGCAATTTATCTCTTCAAATGTAGCACCTGAAGTCAGCCCCATACGATAT<br>AAGTTGTTACTAGATTGACAGCTAGCTCAGTCCTAGGTATAATACTAGTTGGTAACCGCAGGACACCGCGTTTTAGAGCTAGAAATAG<br>CAAGTTAAAATAAGGCTAGTCCGTTATCAACTTGAAAAAGTGGCACATGAGGATCACCCATGTGCTTTTTTTGAAGCTTGGGCCCGAAC<br>AAAAACTCATCTCAGAAGAGGATCTGAATAGCGCCGTCGACCATCATCATCATCATCATTGAGTTTAAACGGTCTCCAGCTTGGCTGTT<br>TTGGCGGATGAGAGAAGATTTTCAGCCTGATACAGATTAAATCAGAACGCAGAAGCGGTCTGATAAAACAGAATTTGCCTGGCGGCAG<br>TAGCGCGGTGGTCCCACCTGACCCCATGCCGAACTCAGAAGTGAAACGCCGTAGCGCCGATGGTAGTGTGGGGTCTCCCCATGCGA<br>GAGTAGGGAACTGCCAGGCATCAAATAAAACGAAAGGCTCAGTCGAAAGACTGGGCCTTTCGTTTTATCTGTTGTTTGTCGGTGAACT<br>GGATCCTTACTCGAGTCTAGACTGCAGGCTTCCTCGCTCACTGACTCGCTGCGCTCGGTCGTTCGGCTGCGGCGAGCGGTATCAGCT<br>CACTCAAAGGCGGTAATACGGTTATCCACAGAATCAGGGGATAACGCAGGAAAGAACATGTGAGCAAAAGGCCAGCAAAAGGCCAGG<br>AACCGTAAAAAACTAGTGCTTGGATTCTCACCAATAAAAAACGCCCGGCGGCAACCGAGCGTTCTGAACAAATCCAGATGGAGTTCTG<br>AGGTCATTACTGGATCTATCAACAGGAGTCCAAGCGAGCTCGATATCAAATTACGCCCCGCCCTGCCACTCATCGCAGTACTGTTGTA<br>ATTCATTAAGCATTCTGCCGACATGGAAGCCATCACAAACGGCATGATGAACCTGAATCGCCAGCCGAGACGAAAGGGCCTCGTGAT<br>ACGCCTATTTTTATAGGTTAATGTCATGATAATAATGGTTTCTTAGACGTCAGGTGGCACTTTTCGGGGAAATGTGCGCGGAACCCCTA<br>TTTGTTTATTTTTCTAAATACATTCAAATATGTATCCGCTCATGAGACAATAACCCTGATAAATGCTTCAATAATATTGAAAAAGGAAGAG<br>TATGAGTATTCAACATTTCCGTGTCGCCCTTATTCCCTTTTTTGCGGCATTTTGCCTTCCTGTTTTTGCTCACCCAGAAACGCTGGTGAA<br>AGTAAAAGATGCTGAAGATCAGTTGGGTGCACGAGTGGGTTACATCGAACTGGATCTCAACAGCGGTAAGATCCTTGAGAGTTTTCGC<br>CCCGAAGAACGTTTTCCAATGATGAGCACTTTTAAAGTTCTGCTATGTGGCGCGGTATTATCCCGTATTGACGCCGGGCAAGAGCAAC<br>TCGGTCGCCGCATACACTATTCTCAGAATGACTTGGTTGAGTACTCACCAGTCACAGAAAAGCATCTTACGGATGGCATGACAGTAAG<br>AGAATTATGCAGTGCTGCCATAACCATGAGTGATAACACTGCGGCCAACTTACTTCTGACAACGATCGGAGGACCGAAGGAGCTAAC<br>CGCTTTTTTGCACAACATGGGGGATCATGTAACTCGCCTTGATCGTTGGGAACCGGAGCTGAATGAAGCCATACCAAACGACGAGCG<br>TGACACCACGATGCCTGTAGCAATGGCAACAACGTTGCGCAAACTATTAACTGGCGAACTACTTACTCTAGCTTCCCGGCAACAATTA<br>ATAGACTGGATGGAGGCGGATAAAGTTGCAGGACCACTTCTGCGCTCGGCCCTTCCGGCTGGCTGGTTTATTGCTGATAAATCTGGA<br>GCCGGTGAGCGTGGGTCTCGCGGTATCATTGCAGCACTGGGGCCAGATGGTAAGCCCTCCCGTATCGTAGTTATCTACACGACGGG<br>GAGTCAGGCAACTATGGATGAACGAAATAGACAGATCGCTGAGATAGGTGCCTCACTGATTAAGCATTGGTAACTGTCAGACCAAGTT<br>TACTCATATATACTTTAGATTGATTTAAAACTTCATTTTTAATTTAAAAGGATCTAGGTGAAGATCCTTTTTGATAATCTCATGACCAAAAT<br>CCCTTAACGTGAGTTTTCGTTCCACTGAGCGTCAGACCCCGTAGAAAAGATCAAAGGATCTTCAAGCAGCATAACCTTTTTCCGTGAT<br>GGTAACTTCACGGTAACCAAGATGTCGAGTTAACCACCCTTTAGATTCATAAAGCGAAAATAATGCGGCTCCAACGTACCCACCTAAAT<br>GGAAACGGCGTTCACTCCAAGCGAAAAAACCCCGCCGAAGCGGGGTTTTTTGCGTTAAAGCCGGTGACGGTAGTCGCTGGGAGTCC<br>GGTCAAATTGACGAGCGAAGACGCGAGAGAAGGTTTGCTGCGACACATAGCCTAAGTCCATGGCAATATCGAAAATCGGACGTTCTG<br>TTGTCCGCAATTCAACGGCAGCCAACAACAAACGGCGTTGACGGATATAGTCTCCTAATGTTTGATGTGTGACGGTACGAAACATGCG<br>CTGTAAATACCACTTCGAGTAACCACTTTTCTTCGCTACAACATCAATATTAAGAGGCTGATCAATATGCTCATCGATCCAAGCAATAAG<br>GTCCTGGATGATTTTTTGGTGGCTCATTGAACCACCTCCGCCGTAAATACCACTGTTGGCCGCGATGGCAGATGGAATCGGATTTCCA<br>TCTTTCAGAAGACCCTGCATCGCTTTCACAATCAACTCACAGTCGCTATTCGTAGCAAAGATCGGAATAGTCAGTTCCATATTAAGGTA<br>CGAACGCCATGCACCTTTAGGGACTTCAACTTTGATCGTGTACTTACGGTTTTGCGCACTCGATTGACGTACAGAACAAGTAACTTTGT<br>ATGCTTGGCTACGGCTGTTGGAGGAAATCCACTCGGCAATGCCGTTAGCGAAGTTGCTAGGGGCAACAGTTACATCACCAGTACCAC<br>CATTATCCACAAGAACGAACTGCGTAAAATTGCTCGCGGGACCCATAGATCCTTTCTCCTCTTTAGATCTTTTGAATTCCCAAAAAAAC<br>GGGTATGGAGAAACAGTAGAGAGTTGCGATAAAAAGCGTCAGGTAGAATCCGCTAATCTTATGGATAAAAATGCTATGGCATAGCAAA<br>GTGTGACGCCGTGCAAATAATCAATGTGGACTTTTCTGCCGTGATTATAGACACTTTTGTTACGCGTTTTTGTCATGGCTTTGGTCCCG<br>CTTTGTTACAGAATGCTTTTAATAAGCGGGGTTACCGGTTTGGTTAGCGAGAAGAGCCAGTAAAAGACGCAGTGACGGCAATGTCTGA<br>TGCAATATGGACAATTGGTTTCTTCTCTGAATGGCGGGAGTATGAAAAGTTGAGATCCTTTTTTTCTGCGCGTAATCTGCTGCTTGCAA<br>ACAAAAAAACCACCGCTACCAGCGGTGGTTTGTTTGCCGGATCAAGAGCTACCAACTCTTTTTCCGAAGGTAACTGGCTTCAGCAGAG<br>CGCAGATACCAAATACTGTTCTTCTAGTGTAGCCGTAGTTAGGCCACCACTTCAAGAACTCTGTAGCACCGCCTACATACCTCGCTCT<br>GCTAATCCTGTTACCAGTGGCTGCTGCCAGTGGCGATAAGTCGTGTCTTACCGGGTTGGACTCAAGACGATAGTTACCGGATAAGGC<br>GCAGCGGTCGGGCTGAACGGGGGGTTCGTGCACACAGCCCAGCTTGGAGCGAACGACCTACACCGAACT	This study
pSJ108	GAGATACCTACAGCGTGAGCTATGAGAAAGCGCCACGCTTCCCGAAGGGAGAAAGGCGGACAGGTATCCGGTAAGCGGCAGGGTC<br>GGAACAGGAGAGCGCACGAGGGAGCTTCCAGGGGGAAACGCCTGGTATCTTTATAGTCCTGTCGGGTTTCGCCACCTCTGACTTGA<br>GCGTCGATTTTTGTGATGCTCGTCAGGGGGGCGGAGCCTATGGAAAAACGCCAGCAACGCGGCCTTTTTACGGTTCCTGGCCTTTTG<br>CTGGCCTTTTGCTCTCAGATAAAATATTTCTAGATTTCAGTGCAATTTATCTCTTCAAATGTAGCACCTGAAGTCAGCCCCATACGATAT<br>AAGTTGTTACTAGATTGACAGCTAGCTCAGTCCTAGGTATAATACTAGTTGGTAACCGCAGGACACCGCGTTTTAGAGCTAGAAATAG<br>CAAGTTAAAATAAGGCTAGTCCGTTATCAACTTGAAAAAGTGGCACATGAGGATCACCCATGTGCTTTTTTTGAAGCTTGGGCCCGAAC<br>AAAAACTCATCTCAGAAGAGGATCTGAATAGCGCCGTCGACCATCATCATCATCATCATTGAGTTTAAACGGTCTCCAGCTTGGCTGTT<br>TTGGCGGATGAGAGAAGATTTTCAGCCTGATACAGATTAAATCAGAACGCAGAAGCGGTCTGATAAAACAGAATTTGCCTGGCGGCAG	This study

TAGCGCGGTGGTCCCACCTGACCCCATGCCGAACTCAGAAGTGAAACGCCGTAGCGCCGATGGTAGTGTGGGGTCTCCCCATGCGA
GAGTAGGGAACTGCCAGGCATCAAATAAAACGAAAGGCTCAGTCGAAAGACTGGGCCTTTCGTTTTATCTGTTGTTTGTCGGTGAACT
GGATCCTTACTCGAGTCTAGACTGCAGGCTTCCTCGCTCACTGACTCGCTGCGCTCGGTCGTTCGGCTGCGGCGAGCGGTATCAGCT
CACTCAAAGGCGGTAATACGGTTATCCACAGAATCAGGGGATAACGCAGGAAAGAACATGTGAGCAAAAGGCCAGCAAAAGGCCAGG
AACCGTAAAAAACTAGTGCTTGGATTCTCACCAATAAAAAACGCCCGGCGGCAACCGAGCGTTCTGAACAAATCCAGATGGAGTTCTG
AGGTCATTACTGGATCTATCAACAGGAGTCCAAGCGAGCTCGATATCAAATTACGCCCCGCCCTGCCACTCATCGCAGTACTGTTGTA
ATTCATTAAGCATTCTGCCGACATGGAAGCCATCACAAACGGCATGATGAACCTGAATCGCCAGCCGAGACGAAAGGGCCTCGTGAT
ACGCCTATTTTTATAGGTTAATGTCATGATAATAATGGTTTCTTACCTCCCGTGGGGAAAAAATCATGGCAATTCTGGAAGAAATAGCG
CTTTCAGCCGGCAAACCGGCTGAAGCCGGATCTGCGATTCTGATAACAAACTAGCAACACCAGAACAGCCCGTTTGCGGGCAGCAAA
ACCCGTACCCTAGGTATAAACGCAGAAAGGCCCACCCGAAGGTGAGCCAGTGTGACTCTAGTAGAGAGCGTTCACCGACAAACAACA
GATAAAACGAAAGGCCCAGTCTTTCGACTGAGCCTTTCGTTTTATTTGATGCCTGGAGATCCTTACTCGAGTTTGGATCCTTAAGCACC
GGTGGAGTGACGACCTTCAGCACGTTCGTACTGTTCAACGATGGTGTAGTCTTCGTTGTGGGAGGTGATGTCCAGTTTGATGTCGGTT
TTGTAAGCACCCGGCAGCTGAACCGGTTTTTTAGCCATGTAGGTGGTTTTAACTTCAGCGTCGTAGTGACCACCGTCTTTCAGTTTCA
GACGCATTTTGATTTCACCTTTCAGAGCACCGTCTTCCGGGTACATACGTTCGGTGGAAGCTTCCCAACCCATGGTTTTTTTCTGCATA
ACCGGACCGTCGGACGGGAAGTTGGTACCACGCAGTTTAACTTTGTAGATGAACTCACCGTCTTGCAGGGAGGAGTCCTGGGTAACG
GTAACAACACCACCGTCTTCGAAGTTCATAACACGTTCCCATTTGAAACCTTCCGGGAAGGACAGTTTCAGGTAGTCCGGGATGTCAG
CCGGGTGTTTAACGTAAGCTTTGGAACCGTACTGGAACTGCGGGGACAGGATGTCCCAAGCGAACGGCAGCGGACCACCTTTGGTA
ACTTTCAGTTTAGCGGTCTGGGTACCTTCGTACGGACGACCTTCACCTTCACCTTCGATTTCGAACTCGTGACCGTTAACGGAACCTT
CCATACGAACTTTGAAACGCATGAACTCTTTGATAACGTCTTCGCTACTCGCCATGGTACCTTTCTCCTCTTTAATGAATTCGCTAGCA
CAATCCCTAGGACTGAGCTAGCTGTCAAGTCCGGAGGTATCCGGTGGAACCCAAAGGACGCCTTTGGTAACCGCAGGACACCGCAG
GATACCTGAGGTCGCCCGTGGTGGCCCATGGTCACCATAGGTCACCCTTGGCAACCAAAGGTGTCCTATGGCGGCCGGAGGCTGCC
ATAGGTCTCCGGTGGATACCGTAGGCGACGTCATCTAGATGCATTCGCGAGGTACCGAGCTCGAATTCACTGGCCGTCGTTTTACAA
CGTCGTGACTGGGAAAACCCTGGCGTTACCCAACTTAATCGCCTTGCAGCACATCCCCCTTTCGCCAGCTGGCGTAATAGCGAAGAG
GCCCGCACCGATCGCCCTTCCCAACAGTTGCGCAGCCTGAATGGCGAATGGCGCCTGATGCGGTATTTTCTCCTTACGCATCTGTGC
GGTATTTCACACCGCATATGGTGCACTCTCAGTACAATCTGCTCTGATGCCGCATAGTTAAGCCAGCCCCGACACCCGCCAACACCC
GCTGACGCGCCCTGACGGGCTTGTCTGCTCCCGGCATCCGCTTACAGACAAGCTGTGACCGTCTCCGGGAGCTGCATGTGTCAGAG
GTTTTCACCGTCATCACCGAAACGCGCGAGACGAAAGGGCCTCGTGATACGCCTATTTTTATAGGTTAATGTCATGATAATAATGGTTT
CTTAGACGTCAGGTGGCACTTTTCGGGGAAATGTGCGCGGAACCCCTATTTGTTTATTTTTCTAAATACATTCAAATATGTATCCGCTC
ATGAGACAATAACCCTGATAAATGCTTCAATAATATTGAAAAAGGAAGAGTATGAGTATTCAACATTTCCGTGTCGCCCTTATTCCCTTT
TTTGCGGCATTTTGCCTTCCTGTTTTTGCTCACCCAGAAACGCTGGTGAAAGTAAAAGATGCTGAAGATCAGTTGGGTGCACGAGTGG
GTTACATCGAACTGGATCTCAACAGCGGTAAGATCCTTGAGAGTTTTCGCCCCGAAGAACGTTTTCCAATGATGAGCACTTTTAAAGTT
CTGCTATGTGGCGCGGTATTATCCCGTATTGACGCCGGGCAAGAGCAACTCGGTCGCCGCATACACTATTCTCAGAATGACTTGGTT
GAGTACTCACCAGTCACAGAAAAGCATCTTACGGATGGCATGACAGTAAGAGAATTATGCAGTGCTGCCATAACCATGAGTGATAACA
CTGCGGCCAACTTACTTCTGACAACGATCGGAGGACCGAAGGAGCTAACCGCTTTTTTGCACAACATGGGGGATCATGTAACTCGCC
TTGATCGTTGGGAACCGGAGCTGAATGAAGCCATACCAAACGACGAGCGTGACACCACGATGCCTGTAGCAATGGCAACAACGTTGC
GCAAACTATTAACTGGCGAACTACTTACTCTAGCTTCCCGGCAACAATTAATAGACTGGATGGAGGCGGATAAAGTTGCAGGACCACT
TCTGCGCTCGGCCCTTCCGGCTGGCTGGTTTATTGCTGATAAATCTGGAGCCGGTGAGCGTGGGTCTCGCGGTATCATTGCAGCACT
GGGGCCAGATGGTAAGCCCTCCCGTATCGTAGTTATCTACACGACGGGGAGTCAGGCAACTATGGATGAACGAAATAGACAGATCGC
TGAGATAGGTGCCTCACTGATTAAGCATTGGTAACTGTCAGACCAAGTTTACTCATATATACTTTAGATTGATTTAAAACTTCATTTTTAA
TTTAAAAGGATCTAGGTGAAGATCCTTTTTGATAATCTCATGACCAAAATCCCTTAACGTGAGTTTTCGTTCCACTGAGCGTCAGACCC
CGTAGAAAAGATCAAAGGATCTTCAAGCAGCATAACCTTTTTCCGTGATGGTAACTTCACGGTAACCAAGATGTCGAGTTAACCACCCT
TTAGATTCATAAAGCGAAAATAATGCGGCTCCAACGTACCCACCTAAATGGAAACGGCGTTCACTCCAAGCGAAAAAACCCCGCCGAA
GCGGGGTTTTTTGCGTTAAAGCCGGTGACGGTAGTCGCTGGGAGTCCGGTCAAATTGACGAGCGAAGACGCGAGAGAAGGTTTGCT
GCGACACATAGCCTAAGTCCATGGCAATATCGAAAATCGGACGTTCTGTTGTCCGCAATTCAACGGCAGCCAACAACAAACGGCGTT
GACGGATATAGTCTCCTAATGTTTGATGTGTGACGGTACGAAACATGCGCTGTAAATACCACTTCGAGTAACCACTTTTCTTCGCTACA
ACATCAATATTAAGAGGCTGATCAATATGCTCATCGATCCAAGCAATAAGGTCCTGGATGATTTTTTGGTGGCTCATTGAACCACCTCC
GCCGTAAATACCACTGTTGGCCGCGATGGCAGATGGAATCGGATTTCCATCTTTCAGAAGACCCTGCATCGCTTTCACAATCAACTCA
CAGTCGCTATTCGTAGCAAAGATCGGAATAGTCAGTTCCATATTAAGGTACGAACGCCATGCACCTTTAGGGACTTCAACTTTGATCGT
GTACTTACGGTTTTGCGCACTCGATTGACGTACAGAACAAGTAACTTTGTATGCTTGGCTACGGCTGTTGGAGGAAATCCACTCGGCA
ATGCCGTTAGCGAAGTTGCTAGGGGCAACAGTTACATCACCAGTACCACCATTATCCACAAGAACGAACTGCGTAAAATTGCTCGCGG
GACCCATAGATCCTTTCTCCTCTTTAGATCTTTTGAATTCCCAAAAAAACGGGTATGGAGAAACAGTAGAGAGTTGCGATAAAAAGCGT
CAGGTAGAATCCGCTAATCTTATGGATAAAAATGCTATGGCATAGCAAAGTGTGACGCCGTGCAAATAATCAATGTGGACTTTTCTGCC
GTGATTATAGACACTTTTGTTACGCGTTTTTGTCATGGCTTTGGTCCCGCTTTGTTACAGAATGCTTTTAATAAGCGGGGTTACCGGTTT
GGTTAGCGAGAAGAGCCAGTAAAAGACGCAGTGACGGCAATGTCTGATGCAATATGGACAATTGGTTTCTTCTCTGAATGGCGGGAG
TATGAAAAGTTGAGATCCTTTTTTTCTGCGCGTAATCTGCTGCTTGCAAACAAAAAAACCACCGCTACCAGCGGTGGTTTGTTTGCCGG
ATCAAGAGCTACCAACTCTTTTTCCGAAGGTAACTGGCTTCAGCAGAGCGCAGATACCAAATACTGTTCTTCTAGTGTAGCCGTAGTTA
GGCCACCACTTCAAGAACTCTGTAGCACCGCCTACATACCTCGCTCTGCTAATCCTGTTACCAGTGGCTGCTGCCAGTGGCGATAAGT

	CGTGTCTTACCGGGTTGGACTCAAGACGATAGTTACCGGATAAGGCGCAGCGGTCGGGCTGAACGGGGGGTTCGTGCACACAGCCC<br>AGCTTGGAGCGAACGACCTACACCGAACT	
p111	GAGATACCTACAGCGTGAGCTATGAGAAAGCGCCACGCTTCCCGAAGGGAGAAAGGCGGACAGGTATCCGGTAAGCGGCAGGGTC<br>GGAACAGGAGAGCGCACGAGGGAGCTTCCAGGGGGAAACGCCTGGTATCTTTATAGTCCTGTCGGGTTTCGCCACCTCTGACTTGA<br>GCGTCGATTTTTGTGATGCTCGTCAGGGGGGCGGAGCCTATGGAAAAACGCCAGCAACGCGGCCTTTTTACGGTTCCTGGCCTTTTG<br>CTGGCCTTTTGCTCTCAGATAAAATATTTCTAGATTTCAGTGCAATTTATCTCTTCAAATGTAGCACCTGAAGTCAGCCCCATACGATAT<br>AAGTTGTTACTAGATTGACAGCTAGCTCAGTCCTAGGTATAATACTAGTGGGCGACCTCAGGTATCCTGGTTTTAGAGCTAGAAATAGC<br>AAGTTAAAATAAGGCTAGTCCGTTATCAACTTGAAAAAGTGGCACATGAGGATCACCCATGTGCTTTTTTTGAAGCTTGGGCCCGAACA<br>AAAACTCATCTCAGAAGAGGATCTGAATAGCGCCGTCGACCATCATCATCATCATCATTGAGTTTAAACGGTCTCCAGCTTGGCTGTTT<br>TGGCGGATGAGAGAAGATTTTCAGCCTGATACAGATTAAATCAGAACGCAGAAGCGGTCTGATAAAACAGAATTTGCCTGGCGGCAGT<br>AGCGCGGTGGTCCCACCTGACCCCATGCCGAACTCAGAAGTGAAACGCCGTAGCGCCGATGGTAGTGTGGGGTCTCCCCATGCGAG<br>AGTAGGGAACTGCCAGGCATCAAATAAAACGAAAGGCTCAGTCGAAAGACTGGGCCTTTCGTTTTATCTGTTGTTTGTCGGTGAACTG<br>GATCCTTACTCGAGTCTAGACTGCAGGCTTCCTCGCTCACTGACTCGCTGCGCTCGGTCGTTCGGCTGCGGCGAGCGGTATCAGCTC<br>ACTCAAAGGCGGTAATACGGTTATCCACAGAATCAGGGGATAACGCAGGAAAGAACATGTGAGCAAAAGGCCAGCAAAAGGCCAGGA<br>ACCGTAAAAAACTAGTGCTTGGATTCTCACCAATAAAAAACGCCCGGCGGCAACCGAGCGTTCTGAACAAATCCAGATGGAGTTCTGA<br>GGTCATTACTGGATCTATCAACAGGAGTCCAAGCGAGCTCGATATCAAATTACGCCCCGCCCTGCCACTCATCGCAGTACTGTTGTAA<br>TTCATTAAGCATTCTGCCGACATGGAAGCCATCACAAACGGCATGATGAACCTGAATCGCCAGCCGAGACGAAAGGGCCTCGTGATA<br>CGCCTATTTTTATAGGTTAATGTCATGATAATAATGGTTTCTTAGACGTCAGGTGGCACTTTTCGGGGAAATGTGCGCGGAACCCCTAT<br>TTGTTTATTTTTCTAAATACATTCAAATATGTATCCGCTCATGAGACAATAACCCTGATAAATGCTTCAATAATATTGAAAAAGGAAGAGT<br>ATGAGTATTCAACATTTCCGTGTCGCCCTTATTCCCTTTTTTGCGGCATTTTGCCTTCCTGTTTTTGCTCACCCAGAAACGCTGGTGAAA<br>GTAAAAGATGCTGAAGATCAGTTGGGTGCACGAGTGGGTTACATCGAACTGGATCTCAACAGCGGTAAGATCCTTGAGAGTTTTCGCC<br>CCGAAGAACGTTTTCCAATGATGAGCACTTTTAAAGTTCTGCTATGTGGCGCGGTATTATCCCGTATTGACGCCGGGCAAGAGCAACT<br>CGGTCGCCGCATACACTATTCTCAGAATGACTTGGTTGAGTACTCACCAGTCACAGAAAAGCATCTTACGGATGGCATGACAGTAAGA<br>GAATTATGCAGTGCTGCCATAACCATGAGTGATAACACTGCGGCCAACTTACTTCTGACAACGATCGGAGGACCGAAGGAGCTAACC<br>GCTTTTTTGCACAACATGGGGGATCATGTAACTCGCCTTGATCGTTGGGAACCGGAGCTGAATGAAGCCATACCAAACGACGAGCGT<br>GACACCACGATGCCTGTAGCAATGGCAACAACGTTGCGCAAACTATTAACTGGCGAACTACTTACTCTAGCTTCCCGGCAACAATTAA<br>TAGACTGGATGGAGGCGGATAAAGTTGCAGGACCACTTCTGCGCTCGGCCCTTCCGGCTGGCTGGTTTATTGCTGATAAATCTGGAG<br>CCGGTGAGCGTGGGTCTCGCGGTATCATTGCAGCACTGGGGCCAGATGGTAAGCCCTCCCGTATCGTAGTTATCTACACGACGGGG<br>AGTCAGGCAACTATGGATGAACGAAATAGACAGATCGCTGAGATAGGTGCCTCACTGATTAAGCATTGGTAACTGTCAGACCAAGTTT<br>ACTCATATATACTTTAGATTGATTTAAAACTTCATTTTTAATTTAAAAGGATCTAGGTGAAGATCCTTTTTGATAATCTCATGACCAAAATC<br>CCTTAACGTGAGTTTTCGTTCCACTGAGCGTCAGACCCCGTAGAAAAGATCAAAGGATCTTCTTGAGATCCTTTTTTTCTGCGCGTAAT<br>CTGCTGCTTGCAAACAAAAAAACCACCGCTACCAGCGGTGGTTTGTTTGCCGGATCAAGAGCTACCAACTCTTTTTCCGAAGGTAACT<br>GGCTTCAGCAGAGCGCAGATACCAAATACTGTTCTTCTAGTGTAGCCGTAGTTAGGCCACCACTTCAAGAACTCTGTAGCACCGCCTA<br>CATACCTCGCTCTGCTAATCCTGTTACCAGTGGCTGCTGCCAGTGGCGATAAGTCGTGTCTTACCGGGTTGGACTCAAGACGATAGTT<br>ACCGGATAAGGCGCAGCGGTCGGGCTGAACGGGGGGTTCGTGCACACAGCCCAGCTTGGAGCGAACGACCTACACCGAACT	This study
pS111	GAGATACCTACAGCGTGAGCTATGAGAAAGCGCCACGCTTCCCGAAGGGAGAAAGGCGGACAGGTATCCGGTAAGCGGCAGGGTC<br>GGAACAGGAGAGCGCACGAGGGAGCTTCCAGGGGGAAACGCCTGGTATCTTTATAGTCCTGTCGGGTTTCGCCACCTCTGACTTGA<br>GCGTCGATTTTTGTGATGCTCGTCAGGGGGGCGGAGCCTATGGAAAAACGCCAGCAACGCGGCCTTTTTACGGTTCCTGGCCTTTTG<br>CTGGCCTTTTGCTCTCAGATAAAATATTTCTAGATTTCAGTGCAATTTATCTCTTCAAATGTAGCACCTGAAGTCAGCCCCATACGATAT<br>AAGTTGTTACTAGATTGACAGCTAGCTCAGTCCTAGGTATAATACTAGTGGGCGACCTCAGGTATCCTGGTTTTAGAGCTAGAAATAGC<br>AAGTTAAAATAAGGCTAGTCCGTTATCAACTTGAAAAAGTGGCACATGAGGATCACCCATGTGCTTTTTTTGAAGCTTGGGCCCGAACA<br>AAAACTCATCTCAGAAGAGGATCTGAATAGCGCCGTCGACCATCATCATCATCATCATTGAGTTTAAACGGTCTCCAGCTTGGCTGTTT<br>TGGCGGATGAGAGAAGATTTTCAGCCTGATACAGATTAAATCAGAACGCAGAAGCGGTCTGATAAAACAGAATTTGCCTGGCGGCAGT<br>AGCGCGGTGGTCCCACCTGACCCCATGCCGAACTCAGAAGTGAAACGCCGTAGCGCCGATGGTAGTGTGGGGTCTCCCCATGCGAG<br>AGTAGGGAACTGCCAGGCATCAAATAAAACGAAAGGCTCAGTCGAAAGACTGGGCCTTTCGTTTTATCTGTTGTTTGTCGGTGAACTG<br>GATCCTTACTCGAGTCTAGACTGCAGGCTTCCTCGCTCACTGACTCGCTGCGCTCGGTCGTTCGGCTGCGGCGAGCGGTATCAGCTC<br>ACTCAAAGGCGGTAATACGGTTATCCACAGAATCAGGGGATAACGCAGGAAAGAACATGTGAGCAAAAGGCCAGCAAAAGGCCAGGA<br>ACCGTAAAAAACTAGTGCTTGGATTCTCACCAATAAAAAACGCCCGGCGGCAACCGAGCGTTCTGAACAAATCCAGATGGAGTTCTGA<br>GGTCATTACTGGATCTATCAACAGGAGTCCAAGCGAGCTCGATATCAAATTACGCCCCGCCCTGCCACTCATCGCAGTACTGTTGTAA<br>TTCATTAAGCATTCTGCCGACATGGAAGCCATCACAAACGGCATGATGAACCTGAATCGCCAGCCGAGACGAAAGGGCCTCGTGATA<br>CGCCTATTTTTATAGGTTAATGTCATGATAATAATGGTTTCTTAGACGTCAGGTGGCACTTTTCGGGGAAATGTGCGCGGAACCCCTAT<br>TTGTTTATTTTTCTAAATACATTCAAATATGTATCCGCTCATGAGACAATAACCCTGATAAATGCTTCAATAATATTGAAAAAGGAAGAGT<br>ATGAGTATTCAACATTTCCGTGTCGCCCTTATTCCCTTTTTTGCGGCATTTTGCCTTCCTGTTTTTGCTCACCCAGAAACGCTGGTGAAA<br>GTAAAAGATGCTGAAGATCAGTTGGGTGCACGAGTGGGTTACATCGAACTGGATCTCAACAGCGGTAAGATCCTTGAGAGTTTTCGCC<br>CCGAAGAACGTTTTCCAATGATGAGCACTTTTAAAGTTCTGCTATGTGGCGCGGTATTATCCCGTATTGACGCCGGGCAAGAGCAACT<br>CGGTCGCCGCATACACTATTCTCAGAATGACTTGGTTGAGTACTCACCAGTCACAGAAAAGCATCTTACGGATGGCATGACAGTAAGA<br>GAATTATGCAGTGCTGCCATAACCATGAGTGATAACACTGCGGCCAACTTACTTCTGACAACGATCGGAGGACCGAAGGAGCTAACC	This study

	GCTTTTTTGCACAACATGGGGGATCATGTAACTCGCCTTGATCGTTGGGAACCGGAGCTGAATGAAGCCATACCAAACGACGAGCGT<br>GACACCACGATGCCTGTAGCAATGGCAACAACGTTGCGCAAACTATTAACTGGCGAACTACTTACTCTAGCTTCCCGGCAACAATTAA<br>TAGACTGGATGGAGGCGGATAAAGTTGCAGGACCACTTCTGCGCTCGGCCCTTCCGGCTGGCTGGTTTATTGCTGATAAATCTGGAG<br>CCGGTGAGCGTGGGTCTCGCGGTATCATTGCAGCACTGGGGCCAGATGGTAAGCCCTCCCGTATCGTAGTTATCTACACGACGGGG<br>AGTCAGGCAACTATGGATGAACGAAATAGACAGATCGCTGAGATAGGTGCCTCACTGATTAAGCATTGGTAACTGTCAGACCAAGTTT<br>ACTCATATATACTTTAGATTGATTTAAAACTTCATTTTTAATTTAAAAGGATCTAGGTGAAGATCCTTTTTGATAATCTCATGACCAAAATC<br>CCTTAACGTGAGTTTTCGTTCCACTGAGCGTCAGACCCCGTAGAAAAGATCAAAGGATCTTCAAGCAGCATAACCTTTTTCCGTGATG<br>GTAACTTCACGGTAACCAAGATGTCGAGTTAACCACCCTTTAGATTCATAAAGCGAAAATAATGCGGCTCCAACGTACCCACCTAAATG<br>GAAACGGCGTTCACTCCAAGCGAAAAAACCCCGCCGAAGCGGGGTTTTTTGCGTTAAAGCCGGTGACGGTAGTCGCTGGGAGTCCG<br>GTCAAATTGACGAGCGAAGACGCGAGAGAAGGTTTGCTGCGACACATAGCCTAAGTCCATGGCAATATCGAAAATCGGACGTTCTGT<br>TGTCCGCAATTCAACGGCAGCCAACAACAAACGGCGTTGACGGATATAGTCTCCTAATGTTTGATGTGTGACGGTACGAAACATGCGC<br>TGTAAATACCACTTCGAGTAACCACTTTTCTTCGCTACAACATCAATATTAAGAGGCTGATCAATATGCTCATCGATCCAAGCAATAAGG<br>TCCTGGATGATTTTTTGGTGGCTCATTGAACCACCTCCGCCGTAAATACCACTGTTGGCCGCGATGGCAGATGGAATCGGATTTCCAT<br>CTTTCAGAAGACCCTGCATCGCTTTCACAATCAACTCACAGTCGCTATTCGTAGCAAAGATCGGAATAGTCAGTTCCATATTAAGGTAC<br>GAACGCCATGCACCTTTAGGGACTTCAACTTTGATCGTGTACTTACGGTTTTGCGCACTCGATTGACGTACAGAACAAGTAACTTTGTA<br>TGCTTGGCTACGGCTGTTGGAGGAAATCCACTCGGCAATGCCGTTAGCGAAGTTGCTAGGGGCAACAGTTACATCACCAGTACCACC<br>ATTATCCACAAGAACGAACTGCGTAAAATTGCTCGCGGGACCCATAGATCCTTTCTCCTCTTTAGATCTTTTGAATTCCCAAAAAAACG<br>GGTATGGAGAAACAGTAGAGAGTTGCGATAAAAAGCGTCAGGTAGAATCCGCTAATCTTATGGATAAAAATGCTATGGCATAGCAAAG<br>TGTGACGCCGTGCAAATAATCAATGTGGACTTTTCTGCCGTGATTATAGACACTTTTGTTACGCGTTTTTGTCATGGCTTTGGTCCCGC<br>TTTGTTACAGAATGCTTTTAATAAGCGGGGTTACCGGTTTGGTTAGCGAGAAGAGCCAGTAAAAGACGCAGTGACGGCAATGTCTGAT<br>GCAATATGGACAATTGGTTTCTTCTCTGAATGGCGGGAGTATGAAAAGTTGAGATCCTTTTTTTCTGCGCGTAATCTGCTGCTTGCAAA<br>CAAAAAAACCACCGCTACCAGCGGTGGTTTGTTTGCCGGATCAAGAGCTACCAACTCTTTTTCCGAAGGTAACTGGCTTCAGCAGAGC<br>GCAGATACCAAATACTGTTCTTCTAGTGTAGCCGTAGTTAGGCCACCACTTCAAGAACTCTGTAGCACCGCCTACATACCTCGCTCTG<br>CTAATCCTGTTACCAGTGGCTGCTGCCAGTGGCGATAAGTCGTGTCTTACCGGGTTGGACTCAAGACGATAGTTACCGGATAAGGCG<br>CAGCGGTCGGGCTGAACGGGGGGTTCGTGCACACAGCCCAGCTTGGAGCGAACGACCTACACCGAACT	
pSJ111	GAGATACCTACAGCGTGAGCTATGAGAAAGCGCCACGCTTCCCGAAGGGAGAAAGGCGGACAGGTATCCGGTAAGCGGCAGGGTC<br>GGAACAGGAGAGCGCACGAGGGAGCTTCCAGGGGGAAACGCCTGGTATCTTTATAGTCCTGTCGGGTTTCGCCACCTCTGACTTGA<br>GCGTCGATTTTTGTGATGCTCGTCAGGGGGGCGGAGCCTATGGAAAAACGCCAGCAACGCGGCCTTTTTACGGTTCCTGGCCTTTTG<br>CTGGCCTTTTGCTCTCAGATAAAATATTTCTAGATTTCAGTGCAATTTATCTCTTCAAATGTAGCACCTGAAGTCAGCCCCATACGATAT<br>AAGTTGTTACTAGATTGACAGCTAGCTCAGTCCTAGGTATAATACTAGTGGGCGACCTCAGGTATCCTGGTTTTAGAGCTAGAAATAGC<br>AAGTTAAAATAAGGCTAGTCCGTTATCAACTTGAAAAAGTGGCACATGAGGATCACCCATGTGCTTTTTTTGAAGCTTGGGCCCGAACA<br>AAAACTCATCTCAGAAGAGGATCTGAATAGCGCCGTCGACCATCATCATCATCATCATTGAGTTTAAACGGTCTCCAGCTTGGCTGTTT<br>TGGCGGATGAGAGAAGATTTTCAGCCTGATACAGATTAAATCAGAACGCAGAAGCGGTCTGATAAAACAGAATTTGCCTGGCGGCAGT<br>AGCGCGGTGGTCCCACCTGACCCCATGCCGAACTCAGAAGTGAAACGCCGTAGCGCCGATGGTAGTGTGGGGTCTCCCCATGCGAG<br>AGTAGGGAACTGCCAGGCATCAAATAAAACGAAAGGCTCAGTCGAAAGACTGGGCCTTTCGTTTTATCTGTTGTTTGTCGGTGAACTG<br>GATCCTTACTCGAGTCTAGACTGCAGGCTTCCTCGCTCACTGACTCGCTGCGCTCGGTCGTTCGGCTGCGGCGAGCGGTATCAGCTC<br>ACTCAAAGGCGGTAATACGGTTATCCACAGAATCAGGGGATAACGCAGGAAAGAACATGTGAGCAAAAGGCCAGCAAAAGGCCAGGA<br>ACCGTAAAAAACTAGTGCTTGGATTCTCACCAATAAAAAACGCCCGGCGGCAACCGAGCGTTCTGAACAAATCCAGATGGAGTTCTGA<br>GGTCATTACTGGATCTATCAACAGGAGTCCAAGCGAGCTCGATATCAAATTACGCCCCGCCCTGCCACTCATCGCAGTACTGTTGTAA<br>TTCATTAAGCATTCTGCCGACATGGAAGCCATCACAAACGGCATGATGAACCTGAATCGCCAGCCGAGACGAAAGGGCCTCGTGATA<br>CGCCTATTTTTATAGGTTAATGTCATGATAATAATGGTTTCTTACCTCCCGTGGGGAAAAAATCATGGCAATTCTGGAAGAAATAGCGC<br>TTTCAGCCGGCAAACCGGCTGAAGCCGGATCTGCGATTCTGATAACAAACTAGCAACACCAGAACAGCCCGTTTGCGGGCAGCAAAA<br>CCCGTACCCTAGGTATAAACGCAGAAAGGCCCACCCGAAGGTGAGCCAGTGTGACTCTAGTAGAGAGCGTTCACCGACAAACAACAG<br>ATAAAACGAAAGGCCCAGTCTTTCGACTGAGCCTTTCGTTTTATTTGATGCCTGGAGATCCTTACTCGAGTTTGGATCCTTAAGCACCG<br>GTGGAGTGACGACCTTCAGCACGTTCGTACTGTTCAACGATGGTGTAGTCTTCGTTGTGGGAGGTGATGTCCAGTTTGATGTCGGTTT<br>TGTAAGCACCCGGCAGCTGAACCGGTTTTTTAGCCATGTAGGTGGTTTTAACTTCAGCGTCGTAGTGACCACCGTCTTTCAGTTTCAG<br>ACGCATTTTGATTTCACCTTTCAGAGCACCGTCTTCCGGGTACATACGTTCGGTGGAAGCTTCCCAACCCATGGTTTTTTTCTGCATAA<br>CCGGACCGTCGGACGGGAAGTTGGTACCACGCAGTTTAACTTTGTAGATGAACTCACCGTCTTGCAGGGAGGAGTCCTGGGTAACG<br>GTAACAACACCACCGTCTTCGAAGTTCATAACACGTTCCCATTTGAAACCTTCCGGGAAGGACAGTTTCAGGTAGTCCGGGATGTCAG<br>CCGGGTGTTTAACGTAAGCTTTGGAACCGTACTGGAACTGCGGGGACAGGATGTCCCAAGCGAACGGCAGCGGACCACCTTTGGTA<br>ACTTTCAGTTTAGCGGTCTGGGTACCTTCGTACGGACGACCTTCACCTTCACCTTCGATTTCGAACTCGTGACCGTTAACGGAACCTT<br>CCATACGAACTTTGAAACGCATGAACTCTTTGATAACGTCTTCGCTACTCGCCATGGTACCTTTCTCCTCTTTAATGAATTCGCTAGCA<br>CAATCCCTAGGACTGAGCTAGCTGTCAAGTCCGGAGGTATCCGGTGGAACCCAAAGGACGCCTTTGGTAACCGCAGGACACCGCAG<br>GATACCTGAGGTCGCCCGTGGTGGCCCATGGTCACCATAGGTCACCCTTGGCAACCAAAGGTGTCCTATGGCGGCCGGAGGCTGCC<br>ATAGGTCTCCGGTGGATACCGTAGGCGACGTCATCTAGATGCATTCGCGAGGTACCGAGCTCGAATTCACTGGCCGTCGTTTTACAA<br>CGTCGTGACTGGGAAAACCCTGGCGTTACCCAACTTAATCGCCTTGCAGCACATCCCCCTTTCGCCAGCTGGCGTAATAGCGAAGAG<br>GCCCGCACCGATCGCCCTTCCCAACAGTTGCGCAGCCTGAATGGCGAATGGCGCCTGATGCGGTATTTTCTCCTTACGCATCTGTGC	This study

	GGTATTTCACACCGCATATGGTGCACTCTCAGTACAATCTGCTCTGATGCCGCATAGTTAAGCCAGCCCCGACACCCGCCAACACCC<br>GCTGACGCGCCCTGACGGGCTTGTCTGCTCCCGGCATCCGCTTACAGACAAGCTGTGACCGTCTCCGGGAGCTGCATGTGTCAGAG<br>GTTTTCACCGTCATCACCGAAACGCGCGAGACGAAAGGGCCTCGTGATACGCCTATTTTTATAGGTTAATGTCATGATAATAATGGTTT<br>CTTAGACGTCAGGTGGCACTTTTCGGGGAAATGTGCGCGGAACCCCTATTTGTTTATTTTTCTAAATACATTCAAATATGTATCCGCTC<br>ATGAGACAATAACCCTGATAAATGCTTCAATAATATTGAAAAAGGAAGAGTATGAGTATTCAACATTTCCGTGTCGCCCTTATTCCCTTT<br>TTTGCGGCATTTTGCCTTCCTGTTTTTGCTCACCCAGAAACGCTGGTGAAAGTAAAAGATGCTGAAGATCAGTTGGGTGCACGAGTGG<br>GTTACATCGAACTGGATCTCAACAGCGGTAAGATCCTTGAGAGTTTTCGCCCCGAAGAACGTTTTCCAATGATGAGCACTTTTAAAGTT<br>CTGCTATGTGGCGCGGTATTATCCCGTATTGACGCCGGGCAAGAGCAACTCGGTCGCCGCATACACTATTCTCAGAATGACTTGGTT<br>GAGTACTCACCAGTCACAGAAAAGCATCTTACGGATGGCATGACAGTAAGAGAATTATGCAGTGCTGCCATAACCATGAGTGATAACA<br>CTGCGGCCAACTTACTTCTGACAACGATCGGAGGACCGAAGGAGCTAACCGCTTTTTTGCACAACATGGGGGATCATGTAACTCGCC<br>TTGATCGTTGGGAACCGGAGCTGAATGAAGCCATACCAAACGACGAGCGTGACACCACGATGCCTGTAGCAATGGCAACAACGTTGC<br>GCAAACTATTAACTGGCGAACTACTTACTCTAGCTTCCCGGCAACAATTAATAGACTGGATGGAGGCGGATAAAGTTGCAGGACCACT<br>TCTGCGCTCGGCCCTTCCGGCTGGCTGGTTTATTGCTGATAAATCTGGAGCCGGTGAGCGTGGGTCTCGCGGTATCATTGCAGCACT<br>GGGGCCAGATGGTAAGCCCTCCCGTATCGTAGTTATCTACACGACGGGGAGTCAGGCAACTATGGATGAACGAAATAGACAGATCGC<br>TGAGATAGGTGCCTCACTGATTAAGCATTGGTAACTGTCAGACCAAGTTTACTCATATATACTTTAGATTGATTTAAAACTTCATTTTTAA<br>TTTAAAAGGATCTAGGTGAAGATCCTTTTTGATAATCTCATGACCAAAATCCCTTAACGTGAGTTTTCGTTCCACTGAGCGTCAGACCC<br>CGTAGAAAAGATCAAAGGATCTTCAAGCAGCATAACCTTTTTCCGTGATGGTAACTTCACGGTAACCAAGATGTCGAGTTAACCACCCT<br>TTAGATTCATAAAGCGAAAATAATGCGGCTCCAACGTACCCACCTAAATGGAAACGGCGTTCACTCCAAGCGAAAAAACCCCGCCGAA<br>GCGGGGTTTTTTGCGTTAAAGCCGGTGACGGTAGTCGCTGGGAGTCCGGTCAAATTGACGAGCGAAGACGCGAGAGAAGGTTTGCT<br>GCGACACATAGCCTAAGTCCATGGCAATATCGAAAATCGGACGTTCTGTTGTCCGCAATTCAACGGCAGCCAACAACAAACGGCGTT<br>GACGGATATAGTCTCCTAATGTTTGATGTGTGACGGTACGAAACATGCGCTGTAAATACCACTTCGAGTAACCACTTTTCTTCGCTACA<br>ACATCAATATTAAGAGGCTGATCAATATGCTCATCGATCCAAGCAATAAGGTCCTGGATGATTTTTTGGTGGCTCATTGAACCACCTCC<br>GCCGTAAATACCACTGTTGGCCGCGATGGCAGATGGAATCGGATTTCCATCTTTCAGAAGACCCTGCATCGCTTTCACAATCAACTCA<br>CAGTCGCTATTCGTAGCAAAGATCGGAATAGTCAGTTCCATATTAAGGTACGAACGCCATGCACCTTTAGGGACTTCAACTTTGATCGT<br>GTACTTACGGTTTTGCGCACTCGATTGACGTACAGAACAAGTAACTTTGTATGCTTGGCTACGGCTGTTGGAGGAAATCCACTCGGCA<br>ATGCCGTTAGCGAAGTTGCTAGGGGCAACAGTTACATCACCAGTACCACCATTATCCACAAGAACGAACTGCGTAAAATTGCTCGCGG<br>GACCCATAGATCCTTTCTCCTCTTTAGATCTTTTGAATTCCCAAAAAAACGGGTATGGAGAAACAGTAGAGAGTTGCGATAAAAAGCGT<br>CAGGTAGAATCCGCTAATCTTATGGATAAAAATGCTATGGCATAGCAAAGTGTGACGCCGTGCAAATAATCAATGTGGACTTTTCTGCC<br>GTGATTATAGACACTTTTGTTACGCGTTTTTGTCATGGCTTTGGTCCCGCTTTGTTACAGAATGCTTTTAATAAGCGGGGTTACCGGTTT<br>GGTTAGCGAGAAGAGCCAGTAAAAGACGCAGTGACGGCAATGTCTGATGCAATATGGACAATTGGTTTCTTCTCTGAATGGCGGGAG<br>TATGAAAAGTTGAGATCCTTTTTTTCTGCGCGTAATCTGCTGCTTGCAAACAAAAAAACCACCGCTACCAGCGGTGGTTTGTTTGCCGG<br>ATCAAGAGCTACCAACTCTTTTTCCGAAGGTAACTGGCTTCAGCAGAGCGCAGATACCAAATACTGTTCTTCTAGTGTAGCCGTAGTTA<br>GGCCACCACTTCAAGAACTCTGTAGCACCGCCTACATACCTCGCTCTGCTAATCCTGTTACCAGTGGCTGCTGCCAGTGGCGATAAGT<br>CGTGTCTTACCGGGTTGGACTCAAGACGATAGTTACCGGATAAGGCGCAGCGGTCGGGCTGAACGGGGGGTTCGTGCACACAGCCC<br>AGCTTGGAGCGAACGACCTACACCGAACT	
pJUB1	AAACCACCGCTACCAGCGGTGGTTTGTTTGCCGGATCAAGAGCTACCAACTCTTTTTCCGAAGGTAACTGGCTTCAGCAGAGCGCAG<br>ATACCAAATACTGTTCTTCTAGTGTAGCCGTAGTTAGGCCACCACTTCAAGAACTCTGTAGCACCGCCTACATACCTCGCTCTGCTAAT<br>CCTGTTACCAGTGGCTGCTGCCAGTGGCGATAAGTCGTGTCTTACCGGGTTGGACTCAAGACGATAGTTACCGGATAAGGCGCAGCG<br>GTCGGGCTGAACGGGGGGTTCGTGCACACAGCCCAGCTTGGAGCGAACGACCTACACCGAACTGAGATACCTACAGCGTGAGCTAT<br>GAGAAAGCGCCACGCTTCCCGAAGGGAGAAAGGCGGACAGGTATCCGGTAAGCGGCAGGGTCGGAACAGGAGAGCGCACGAGGG<br>AGCTTCCAGGGGGAAACGCCTGGTATCTTTATAGTCCTGTCGGGTTTCGCCACCTCTGACTTGAGCGTCGATTTTTGTGATGCTCGTC<br>AGGGGGGCGGAGCCTATGGAAAAACGCCAGCAACGCGGCCTTTTTACGGTTCCTGGCCTTTTGCTGGCCTTTTGCTCATGATATAATT<br>AAATTGAAGCTCTAATTTGTGAGTTTAGTATACATGCATTTACTTATAATACAGTTTTTTAGTTTTGCTGGCCGCATCTTCTCAAATATGC<br>TTCCCAGCCTGCTTTTCTGTAACGTTCACCCTCTACCTTAGCATCCCTTCCCTTTGCAAATAGTCCTCTTCCAACAATAATAATGTCAGA<br>TCCTGTAGAGACCACATCATCCACGGTTCTATACTGTTGACCCAATGCGTCTCCCTTGTCATCTAAACCCACACCGGGTGTCATAATCA<br>ACCAATCGTAACCTTCATCTCTTCCACCCATGTCTCTTTGAGCAATAAAGCCGATAACAAAATCTTTGTCGCTCTTCGCAATGTCAACA<br>GTACCCTTAGTATATTCTCCAGTAGATAGGGAGCCCTTGCATGACAATTCTGCTAACATCAAAAGGCCTCTAGGTTCCTTTGTTACTTC<br>TTCTGCCGCCTGCTTCAAACCGCTAACAATACCTGGGCCCACCACACCGTGTGCATTCGTAATGTCTGCCCATTCTGCTATTCTGTATA<br>CACCCGCAGAGTACTGCAATTTGACTGTATTACCAATGTCAGCAAATTTTCTGTCTTCGAAGAGTAAAAAATTGTACTTGGCGGATAAT<br>GCCTTTAGCGGCTTAACTGTGCCCTCCATGGAAAAATCAGTCAAGATATCCACATGTGTTTTTAGTAAACAAATTTTGGGACCTAATGC<br>TTCAACTAACTCCAGTAATTCCTTGGTGGTACGAACATCCAATGAAGCACACAAGTTTGTTTGCTTTTCGTGCATGATATTAAATAGCTT<br>GGCAGCAACAGGACTAGGATGAGTAGCAGCACGTTCCTTATATGTAGCTTTCGACATGATTTATCTTCGTTTCCTGAGGTTTTTGTTCT<br>GTGCAGTTGGGTTAAGAATACTGGGCAATTTCATGTTTCTTCAACACTACATATGCGTATATATACCAATCTAAGTCTGTGCTCCTTCCT<br>TCGTTCTTCCTTCTGTTCGGAGATTACCGAATCAAAAAAATTTCAAAGAAACCGAAATCAAAAAAAAGAATAAAAAAAAAATGATGAATT<br>GAATTGAAAAGCTAGCTTATCGATGATAAGCTGTCAAAGATGAGAATTAATTCCACGGACTATAGACTATACTAGATACTCCGTCTACT<br>GTACGATACACTTCCGCTCAGGTCCTTGTCCTTTAACGAGGCCTTACCACTCTTTTGTTACTCTATTGATCCAGCTCAGCAAAGGCAGT<br>GTGATCTAAGATTCTATCTTCGCGATGTAGTAAAACTAGCTAGACCGAGAAAGAGACTAGAAATGCAAAAGGCACTTCTACAATGGCT	This study

	GCCATCATTATTATCCGATGTGACGCTGCAGCTTCTCAATGATATTCGAATACGCTTTGAGGAGATACAGCCTAATATCCGACAAACTG<br>TTTTACAGATTTACGATCGTACTTGTTACCCATCATTGAATTTTGAACATCCGAACCTGGGAGTTTTCCCTGAAACAGATAGTATATTTG<br>AACCTGTATAATAATATATAGTCTAGCGCTTTACGGAAGACAATGTATGTATTTCGGTTCCTGGAGAAACTATTGCATCTATTGCATAGG<br>TAATCTTGCACGTCGCATCCCCGGTTCATTTTCTGCGTTTCCATCTTGCACTTCAATAGCATATCTTTGTTAACGAAGCATCTGTGCTTC<br>ATTTTGTAGAACAAAAATGCAACGCGAGAGCGCTAATTTTTCAAACAAAGAATCTGAGCTGCATTTTTACAGAACAGAAATGCAACGCG<br>AAAGCGCTATTTTACCAACGAAGAATCTGTGCTTCATTTTTGTAAAACAAAAATGCAACGCGACGAGAGCGCTAATTTTTCAAACAAAG<br>AATCTGAGCTGCATTTTTACAGAACAGAAATGCAACGCGAGAGCGCTATTTTACCAACAAAGAATCTATACTTCTTTTTTGTTCTACAAA<br>AATGCATCCCGAGAGCGCTATTTTTCTAACAAAGCATCTTAGATTACTTTTTTTCTCCTTTGTGCGCTCTATAATGCAGTCTCTTGATAA<br>CTTTTTGCACTGTAGGTCCGTTAAGGTTAGAAGAAGGCTACTTTGGTGTCTATTTTCTCTTCCATAAAAAAAGCCTGACTCCACTTCCC<br>GCGTTTACTGATTACTAGCGAAGCTGCGGGTGCATTTTTTCAAGATAAAGGCATCCCCGATTATATTCTATACCGATGTGGATTGCGCA<br>TACTTTGTGAACAGAAAGTGATAGCGTTGATGATTCTTCATTGGTCAGAAAATTATGAACGGTTTCTTCTATTTTGTCTCTATATACTAC<br>GTATAGGAAATGTTTACATTTTCGTATTGTTTTCGATTCACTCTATGAATAGTTCTTACTACAATTTTTTTGTCTAAAGAGTAATACTAGA<br>GATAAACATAAAAAATGTAGAGGTCGAGTTTAGATGCAAGTTCAAGGAGCGAAAGGTGGATGGGTAGGTTATATAGGGATATAGCACA<br>GAGATATATAGCAAAGAGATACTTTTGAGCAATGTTTGTGGAAGCGGTATTCGCAATGGGAAGCTCCACCCCGGTTGATAATCAGAAA<br>AGCCCCAAAAACAGGAAGATTGTATAAGCAAATATTTAAATTGTAATTCTTTCCTGCGTTATCCCCTGATTCTGTGGATAACCGTATTAC<br>CGCCTTTGAGTGAGCTGATACCGCTCGCCGCAGCCGAACGACCGAGCGCAGCGAGTCAGTGAGCGAGGAAGCGGAAGAGGGTCTG<br>ACGCTCAGTGGAACGAAAACTCACGTTAAGGGATTTTGGTCATGAGATTATCAAAAAGGATCTTCACCTAGATCCTTTTAAATTAAAAAT<br>GAAGTTTTAAATCAATCTAAAGTATATATGAGTAAACTTGGTCTGACAGTCAGAAGAACTCGTCAAGAAGGCGATAGAAGGCGATGCG<br>CTGCGAATCGGGAGCGGCGATACCGTAAAGCACGAGGAAGCGGTCAGCCCATTCGCCGCCAAGCTCTTCAGCAATATCACGGGTAG<br>CCAACGCTATGTCCTGATAGCGGTCCGCCACACCCAGCCGGCCACAGTCGATGAATCCAGAAAAGCGGCCATTTTCCACCATGATAT<br>TCGGCAAGCAGGCATCGTCATGGGTCACGACGAGATCCTCGCCGTCGGGCATGCTCGCCTTGAGCCTGGCGAACAGTTCGGCTGGC<br>GCGAGCCCCTGATGCTCTTCGTCCAGATCATCCTGATCGACAAGACCGGCTTCCATCCGAGTACGTGCTCGCTCGATGCGATGTTTC<br>GCTTGGTGGTCGAATGGGCAGGTAGCCGGATCAAGCGTATGCAGCCGCCGCATTGCATCAGCCATGATGGATACTTTCTCGGCAGG<br>AGCAAGGTGAGATGACAGGAGATCCTGCCCCGGCACTTCGCCCAATAGCAGCCAGTCCCTTCCCGCTTCAGTGACAACGTCGAGCA<br>CAGCTGCGCAAGGAACGCCCGTCGTGGCCAGCCACGATAGCCGCGCTGCCTCGTCTTGCAGTTCATTCAGGGCACCGGACAGGTCG<br>GTCTTGACAAAAAGAACCGGGCGCCCCTGCGCTGACAGCCGGAACACGGCGGCATCAGAGCAGCCGATTGTCTGTTGTGCCCAGTC<br>ATAGCCGAATAGCCTCTCCACCCAAGCGGCCGGAGAACCTGCGTGCAATCCATCTTGTTCAATCATACTCTTCCTTTTTCAATATTATT<br>GAAGCATTTATCAGGGTTATTGTCTCATGAGCGGATACATATTTGAATGTATTTAGAAAAATAAACAAATAGGGGTTCCGCGCACATTT<br>CCCCGAAAAGTGCCACCTGCGGACGGATCGCTTGCCTGTAACTTACACGCGCCTCGTCCTGAGAAAGCAACCTGACCTACAGGAAAG<br>AGTTACTCAAGAATAAGAATTTTCGTTTTAAAACCTAAGAGTCACTTTAAAATTTGTATACACTTATTTTTTTTATAACTTATTTAATAATAA<br>AAATCATAAATCATAAGAAATTCGCTTATTTAGAAGTGTCAACAACGTATCTACCAACGATTTGACCCTTTTCCATCTTTTCGTAAATTTC<br>TGGCAAGGTAGACAAGCCGACAACCTTGATTGGAGACTTGACCAAACCTCTGGCGAAGAATTGTTAATATTAAGAGCTCTCACTGCCC<br>ACTTTCCAGTCTGGAAACCTGTCTTGCTAGCTGCATGAGTGAATCAGCCAATGCCCGGGGAGAGGCAGTTTGTGTATTGGGTGCCAG<br>GGTGGTTTTTCTCTTCACCAGTGAGACTGGCAACAGCTGATTGCCCTTCACCGCCTGGCCCTGAGAGAGTTGCAGCAAGCGGTCCAC<br>GCTGGTTTGCCCCAGCAGGCGAAAATCCTGTTTGATGGTGGTTAACGGCGGGATATAACATGAGCTGTCTTCGGTATCGTCGTATCC<br>CACTACCGAGATATCTGCACCAACTCTCAGCCCAGACTCAGTAATGGCTCTCATTGCACCCAGTGCCATCTGATCATTGGCAACCAGC<br>ATTGCAGTGGGAACAATGCCCTCATTCAGCATTTGCATGGTTTGTTGAAACCCAGACATGGCACTCCAGTCCCCTTCTCTTTCAGCTAT<br>TGGCTGAATTTGATTCCTAGTGAGATATTTATGCCAGCCGGCCAGTCTCAGCCTTGCTGAGACAGAACTGAGTGGGCCCGCAAGCAG<br>TGCAATTTGCTGGTGTCCCAATGCAACCAGATGCTCCACACCCAGTCTTGTACCATCTTCATGGGAGAAAATAATACTGTTGATGGGT<br>GTCTGGTCAGAGACATCAAGAAAGAGTGCTGGAACATTAGTGCAGGCAGCTTCCACAGCAATGGCATCCTGGTCATCCAGTGGATAG<br>TTAATGATCAGCCCACTGACTCTTTGTGCCAGAAGATTGTGCACAGCAGCTTTACAGGCTTCAACTCCACTTCTTTCTACCATTGACAC<br>CACCACAGAGGCTCCCAGTTGATCAGCTCTAGATTTAATGGCTGCCACAATTTGAGATGGTGCATGCAGGGCCAGACTGGAGGTGGC<br>AACTCCAATCAGCAAGCTCTGTTTGCCTGCCAGTTGTTGTGCCACTCTGTTGGGAATGTAATTCAGCTCTGCCATGGCTGCTTCCACTT<br>TTTCCCTGGTTTTGGCAGAAACATGGCTGGCCTGGTTCACCACTCTGGAAACAGTCTGATAAGAGACACCGGCATACTCTGCGACATC<br>ATACAATGTTACTGGTTTCATTTTAATAACTAGTGCGAAAAAACCCCGCCGAAGCGGGGTTTTTTGCGTTAAAGCCGGTGACGGTAGT<br>CGCTGGGAGTCCGGTCAAATTGACGAGCGAAGACGCGAGAGAAGGTTTGCTGCGACACATAGCCTAAGTCCATGGCAATATCGAAAA<br>TCGGACGTTCTGTTGTCCGCAATTCAACGGCAGCCAACAACAAACGGCGTTGACGGATATAGTCTCCTAATGTTTGATGTGTGACGGT<br>ACGAAACATGCGCTGTAAATACCACTTCGAGTAACCACTTTTCTTCGCTACAACATCAATATTAAGAGGCTGATCAATATGCTCATCGA<br>TCCAAGCAATAAGGTCCTGGATGATTTTTTGGTGGCTCATTGAACCACCTCCGCCGGGTTTAGTGTTGCCATCTATAACAGATCTAAGT<br>TCATCCCAACTTGCTGAGTCTCCTATGAAATCTCCACCGTTGCCGTTCCAGAAACTCATCAGGAGGTTATTATCATTACCAGTATATGT<br>CGGTTGATTAAAACCAACTATATGTTGGTTCCAATAAGGATTCTGTGGCTGTGGAAGCGGCGGCTCGTGGGACATGGAATCTACTGTA<br>CGGTGGCTCGTGTGGTCGGAATCTAAGCTGCTGGTCTTAGAACAAGTGTCGGTTAAAGTGATAACCGGTTTTCGGTTTGGTGGTAAGA<br>TGGTTGGGTTTCTTTGAGATGTGACTCGTTTGAAGATTCTGCAAAGTGTCCATACCTCTGCTTGTTGAGCTGGAGAGTCGGTTTTCGTG<br>GTGGAGGGGGAGGCGGAATTCATGCATCATCCAATCGGTTTTGGTGCCTTTACCGGCTGAACCAAGATAGTAAACCAGAGATTTCTTGA<br>GACCAACACAGTCAAGATTGGAGTAAACCGGTTTATCAATACCAGTGGCTTTCCAGAAACCTGAACCGGTCACTCGGTTTGGTCGAAC<br>GCTATTCCTGTATTTCCTACCTCTCATGCAGAAGAAGTACCACTCCTTTTCTCCGACGCTGCTCACTCTTGGAAGATCCCAAGGATCGT<br>ACTTATAGATATCGATCTGTTTGATAAGTTCGAGTTTGATGGTTTTGTTCTCTACTTTTCTTCGAAGATAGTATCCTAAAAGCTCTTCATC	

	CGTCGGATGAAACCTGAACCCAGGAAGTGGTGCTTCGTTTTCTTCTTCATGATCCTTACCTAAGTTACCTTCGCCACTCATGTCGACAG<br>ATCCTTTCTCCTCTTTAGATCTTTTGAATTCCCAAAAAAACGGGTATGGAGAAACAGTAGAGAGTTGCGATAAAAAGCGTCAGGTAGAA<br>TCCGCTAATCTTATGGATAAAAATGCTATGGCATAGCAAAGTGTGACGCCGTGCAAATAATCAATGTGGACTTTTCTGCCGTGATTATA<br>GACACTTTTGTTACGCGTTTTTGTCATGGCTTTGGTCCCGCTTTGTTACAGAATGCTTTTAATAAGCGGGGTTACCGGTTTGGTTAGCG<br>AGAAGAGCCAGTAAAAGACGCAGTGACGGCAATGTCTGATGCAATATGGACAATTGGTTTCTTCTCTGAATGGCGGGAGTATGAAAAG<br>TTCAACATTTCCGTGTCGCCCTTATTCCCTTTTTTGCGGCATTTTGCCTTCCTGTTTTTGCTCACCCAGAAACGCTGGTGAAAGTAAAA<br>GATGCTGAAGATCAGTTGGGTGCACGAGTGGGTTACATCGAACTGGATCTCAACAGCGGTAAGATCCTTGAGAGTTTTCGCCCCGAA<br>GAACGTTTTCCAATGATGAGCACTTTTAAAGTTCTGCTATGTGGCGCGGTATTATCCCGTATTGACGCCGGGCAAGAGCAACTCGGTC<br>GCCGCATACACTATTCTCAGAATGACTTGGTTGAGTACTCACCAGTCACAGAAAAGCATCTTACGGATGGCATGACAGTAAGAGAATT<br>ATGCAGTGCTGCCATAACCATGAGTGATAACACTGCGGCCAACTTACTTCTGACAACGATCGGAGGACCGAAGGAGCTAACCGCTTTT<br>TTGCACAACATGGGGGATCATGTAACTCGCCTTGATCGTTGGGAACCGGAGCTGAATGAAGCCATACCAAACGACGAGCGTGACACC<br>ACGATGCCTGTAGCAATGGCAACAACGTTGCGCAAACTATTAACTGGCGAACTACTTACTCTAGCTTCCCGGCAACAATTAATAGACT<br>GGATGGAGGCGGATAAAGTTGCAGGACCACTTCTGCGCTCGGCCCTTCCGGCTGGCTGGTTTATTGCTGATAAATCTGGAGCCGGTG<br>AGCGTGGGTCTCGCGGTATCATTGCAGCACTGGGGCCAGATGGTAAGCCCTCCCGTATCGTAGTTATCTACACGACGGGGAGTCAG<br>GCAACTATGGATGAACGAAATAGACAGATCGCTGAGATAGGTGCCTCACTGATTAAGCATTGGTAAAATTATTAACGCTTACAATTTCC<br>TGATGCGGTATTTTCTCCTTACGCATCTGTGCGGTATTTCACACCGCATCAGGTGGCACTTTTCGGGGAAATGTGCGCGGAACCCCTA<br>TTTGTTTATTTTTCTAAATACATTCAAATATGTATCCGCTGGATCTCATGCTGGAGTTCTTCGCCCACCCTTAATTAACCGTAGAAAAGA<br>TCAAAGGATCTTCTTGAGATCCTTTTTTTCTGCGCGTAATCTGCTGCTTGCAAACAAAA	
pJUB1DBD	CTCTACTGTTTCTCCATACCCGTTTTTTTGGGAATTCAAAAGATCTAAAGAGGAGAAAGGATCTGTCGACATGCCACTTCCTGGGTTCA<br>GG	This study
pANAC102	AAACCACCGCTACCAGCGGTGGTTTGTTTGCCGGATCAAGAGCTACCAACTCTTTTTCCGAAGGTAACTGGCTTCAGCAGAGCGCAG<br>ATACCAAATACTGTTCTTCTAGTGTAGCCGTAGTTAGGCCACCACTTCAAGAACTCTGTAGCACCGCCTACATACCTCGCTCTGCTAAT<br>CCTGTTACCAGTGGCTGCTGCCAGTGGCGATAAGTCGTGTCTTACCGGGTTGGACTCAAGACGATAGTTACCGGATAAGGCGCAGCG<br>GTCGGGCTGAACGGGGGGTTCGTGCACACAGCCCAGCTTGGAGCGAACGACCTACACCGAACTGAGATACCTACAGCGTGAGCTAT<br>GAGAAAGCGCCACGCTTCCCGAAGGGAGAAAGGCGGACAGGTATCCGGTAAGCGGCAGGGTCGGAACAGGAGAGCGCACGAGGG<br>AGCTTCCAGGGGGAAACGCCTGGTATCTTTATAGTCCTGTCGGGTTTCGCCACCTCTGACTTGAGCGTCGATTTTTGTGATGCTCGTC<br>AGGGGGGCGGAGCCTATGGAAAAACGCCAGCAACGCGGCCTTTTTACGGTTCCTGGCCTTTTGCTGGCCTTTTGCTCATGATATAATT<br>AAATTGAAGCTCTAATTTGTGAGTTTAGTATACATGCATTTACTTATAATACAGTTTTTTAGTTTTGCTGGCCGCATCTTCTCAAATATGC<br>TTCCCAGCCTGCTTTTCTGTAACGTTCACCCTCTACCTTAGCATCCCTTCCCTTTGCAAATAGTCCTCTTCCAACAATAATAATGTCAGA<br>TCCTGTAGAGACCACATCATCCACGGTTCTATACTGTTGACCCAATGCGTCTCCCTTGTCATCTAAACCCACACCGGGTGTCATAATCA<br>ACCAATCGTAACCTTCATCTCTTCCACCCATGTCTCTTTGAGCAATAAAGCCGATAACAAAATCTTTGTCGCTCTTCGCAATGTCAACA<br>GTACCCTTAGTATATTCTCCAGTAGATAGGGAGCCCTTGCATGACAATTCTGCTAACATCAAAAGGCCTCTAGGTTCCTTTGTTACTTC<br>TTCTGCCGCCTGCTTCAAACCGCTAACAATACCTGGGCCCACCACACCGTGTGCATTCGTAATGTCTGCCCATTCTGCTATTCTGTATA<br>CACCCGCAGAGTACTGCAATTTGACTGTATTACCAATGTCAGCAAATTTTCTGTCTTCGAAGAGTAAAAAATTGTACTTGGCGGATAAT<br>GCCTTTAGCGGCTTAACTGTGCCCTCCATGGAAAAATCAGTCAAGATATCCACATGTGTTTTTAGTAAACAAATTTTGGGACCTAATGC<br>TTCAACTAACTCCAGTAATTCCTTGGTGGTACGAACATCCAATGAAGCACACAAGTTTGTTTGCTTTTCGTGCATGATATTAAATAGCTT<br>GGCAGCAACAGGACTAGGATGAGTAGCAGCACGTTCCTTATATGTAGCTTTCGACATGATTTATCTTCGTTTCCTGAGGTTTTTGTTCT<br>GTGCAGTTGGGTTAAGAATACTGGGCAATTTCATGTTTCTTCAACACTACATATGCGTATATATACCAATCTAAGTCTGTGCTCCTTCCT<br>TCGTTCTTCCTTCTGTTCGGAGATTACCGAATCAAAAAAATTTCAAAGAAACCGAAATCAAAAAAAAGAATAAAAAAAAAATGATGAATT<br>GAATTGAAAAGCTAGCTTATCGATGATAAGCTGTCAAAGATGAGAATTAATTCCACGGACTATAGACTATACTAGATACTCCGTCTACT<br>GTACGATACACTTCCGCTCAGGTCCTTGTCCTTTAACGAGGCCTTACCACTCTTTTGTTACTCTATTGATCCAGCTCAGCAAAGGCAGT<br>GTGATCTAAGATTCTATCTTCGCGATGTAGTAAAACTAGCTAGACCGAGAAAGAGACTAGAAATGCAAAAGGCACTTCTACAATGGCT<br>GCCATCATTATTATCCGATGTGACGCTGCAGCTTCTCAATGATATTCGAATACGCTTTGAGGAGATACAGCCTAATATCCGACAAACTG<br>TTTTACAGATTTACGATCGTACTTGTTACCCATCATTGAATTTTGAACATCCGAACCTGGGAGTTTTCCCTGAAACAGATAGTATATTTG<br>AACCTGTATAATAATATATAGTCTAGCGCTTTACGGAAGACAATGTATGTATTTCGGTTCCTGGAGAAACTATTGCATCTATTGCATAGG<br>TAATCTTGCACGTCGCATCCCCGGTTCATTTTCTGCGTTTCCATCTTGCACTTCAATAGCATATCTTTGTTAACGAAGCATCTGTGCTTC<br>ATTTTGTAGAACAAAAATGCAACGCGAGAGCGCTAATTTTTCAAACAAAGAATCTGAGCTGCATTTTTACAGAACAGAAATGCAACGCG<br>AAAGCGCTATTTTACCAACGAAGAATCTGTGCTTCATTTTTGTAAAACAAAAATGCAACGCGACGAGAGCGCTAATTTTTCAAACAAAG<br>AATCTGAGCTGCATTTTTACAGAACAGAAATGCAACGCGAGAGCGCTATTTTACCAACAAAGAATCTATACTTCTTTTTTGTTCTACAAA<br>AATGCATCCCGAGAGCGCTATTTTTCTAACAAAGCATCTTAGATTACTTTTTTTCTCCTTTGTGCGCTCTATAATGCAGTCTCTTGATAA<br>CTTTTTGCACTGTAGGTCCGTTAAGGTTAGAAGAAGGCTACTTTGGTGTCTATTTTCTCTTCCATAAAAAAAGCCTGACTCCACTTCCC<br>GCGTTTACTGATTACTAGCGAAGCTGCGGGTGCATTTTTTCAAGATAAAGGCATCCCCGATTATATTCTATACCGATGTGGATTGCGCA<br>TACTTTGTGAACAGAAAGTGATAGCGTTGATGATTCTTCATTGGTCAGAAAATTATGAACGGTTTCTTCTATTTTGTCTCTATATACTAC<br>GTATAGGAAATGTTTACATTTTCGTATTGTTTTCGATTCACTCTATGAATAGTTCTTACTACAATTTTTTTGTCTAAAGAGTAATACTAGA<br>GATAAACATAAAAAATGTAGAGGTCGAGTTTAGATGCAAGTTCAAGGAGCGAAAGGTGGATGGGTAGGTTATATAGGGATATAGCACA<br>GAGATATATAGCAAAGAGATACTTTTGAGCAATGTTTGTGGAAGCGGTATTCGCAATGGGAAGCTCCACCCCGGTTGATAATCAGAAA	This study

AGCCCCAAAAACAGGAAGATTGTATAAGCAAATATTTAAATTGTAATTCTTTCCTGCGTTATCCCCTGATTCTGTGGATAACCGTATTAC
CGCCTTTGAGTGAGCTGATACCGCTCGCCGCAGCCGAACGACCGAGCGCAGCGAGTCAGTGAGCGAGGAAGCGGAAGAGGGTCTG
ACGCTCAGTGGAACGAAAACTCACGTTAAGGGATTTTGGTCATGAGATTATCAAAAAGGATCTTCACCTAGATCCTTTTAAATTAAAAAT
GAAGTTTTAAATCAATCTAAAGTATATATGAGTAAACTTGGTCTGACAGTCAGAAGAACTCGTCAAGAAGGCGATAGAAGGCGATGCG
CTGCGAATCGGGAGCGGCGATACCGTAAAGCACGAGGAAGCGGTCAGCCCATTCGCCGCCAAGCTCTTCAGCAATATCACGGGTAG
CCAACGCTATGTCCTGATAGCGGTCCGCCACACCCAGCCGGCCACAGTCGATGAATCCAGAAAAGCGGCCATTTTCCACCATGATAT
TCGGCAAGCAGGCATCGTCATGGGTCACGACGAGATCCTCGCCGTCGGGCATGCTCGCCTTGAGCCTGGCGAACAGTTCGGCTGGC
GCGAGCCCCTGATGCTCTTCGTCCAGATCATCCTGATCGACAAGACCGGCTTCCATCCGAGTACGTGCTCGCTCGATGCGATGTTTC
GCTTGGTGGTCGAATGGGCAGGTAGCCGGATCAAGCGTATGCAGCCGCCGCATTGCATCAGCCATGATGGATACTTTCTCGGCAGG
AGCAAGGTGAGATGACAGGAGATCCTGCCCCGGCACTTCGCCCAATAGCAGCCAGTCCCTTCCCGCTTCAGTGACAACGTCGAGCA
CAGCTGCGCAAGGAACGCCCGTCGTGGCCAGCCACGATAGCCGCGCTGCCTCGTCTTGCAGTTCATTCAGGGCACCGGACAGGTCG
GTCTTGACAAAAAGAACCGGGCGCCCCTGCGCTGACAGCCGGAACACGGCGGCATCAGAGCAGCCGATTGTCTGTTGTGCCCAGTC
ATAGCCGAATAGCCTCTCCACCCAAGCGGCCGGAGAACCTGCGTGCAATCCATCTTGTTCAATCATACTCTTCCTTTTTCAATATTATT
GAAGCATTTATCAGGGTTATTGTCTCATGAGCGGATACATATTTGAATGTATTTAGAAAAATAAACAAATAGGGGTTCCGCGCACATTT
CCCCGAAAAGTGCCACCTGCGGACGGATCGCTTGCCTGTAACTTACACGCGCCTCGTCCTGAGAAAGCAACCTGACCTACAGGAAAG
AGTTACTCAAGAATAAGAATTTTCGTTTTAAAACCTAAGAGTCACTTTAAAATTTGTATACACTTATTTTTTTTATAACTTATTTAATAATAA
AAATCATAAATCATAAGAAATTCGCTTATTTAGAAGTGTCAACAACGTATCTACCAACGATTTGACCCTTTTCCATCTTTTCGTAAATTTC
TGGCAAGGTAGACAAGCCGACAACCTTGATTGGAGACTTGACCAAACCTCTGGCGAAGAATTGTTAATATTAAGAGCTCTCACTGCCC
ACTTTCCAGTCTGGAAACCTGTCTTGCTAGCTGCATGAGTGAATCAGCCAATGCCCGGGGAGAGGCAGTTTGTGTATTGGGTGCCAG
GGTGGTTTTTCTCTTCACCAGTGAGACTGGCAACAGCTGATTGCCCTTCACCGCCTGGCCCTGAGAGAGTTGCAGCAAGCGGTCCAC
GCTGGTTTGCCCCAGCAGGCGAAAATCCTGTTTGATGGTGGTTAACGGCGGGATATAACATGAGCTGTCTTCGGTATCGTCGTATCC
CACTACCGAGATATCTGCACCAACTCTCAGCCCAGACTCAGTAATGGCTCTCATTGCACCCAGTGCCATCTGATCATTGGCAACCAGC
ATTGCAGTGGGAACAATGCCCTCATTCAGCATTTGCATGGTTTGTTGAAACCCAGACATGGCACTCCAGTCCCCTTCTCTTTCAGCTAT
TGGCTGAATTTGATTCCTAGTGAGATATTTATGCCAGCCGGCCAGTCTCAGCCTTGCTGAGACAGAACTGAGTGGGCCCGCAAGCAG
TGCAATTTGCTGGTGTCCCAATGCAACCAGATGCTCCACACCCAGTCTTGTACCATCTTCATGGGAGAAAATAATACTGTTGATGGGT
GTCTGGTCAGAGACATCAAGAAAGAGTGCTGGAACATTAGTGCAGGCAGCTTCCACAGCAATGGCATCCTGGTCATCCAGTGGATAG
TTAATGATCAGCCCACTGACTCTTTGTGCCAGAAGATTGTGCACAGCAGCTTTACAGGCTTCAACTCCACTTCTTTCTACCATTGACAC
CACCACAGAGGCTCCCAGTTGATCAGCTCTAGATTTAATGGCTGCCACAATTTGAGATGGTGCATGCAGGGCCAGACTGGAGGTGGC
AACTCCAATCAGCAAGCTCTGTTTGCCTGCCAGTTGTTGTGCCACTCTGTTGGGAATGTAATTCAGCTCTGCCATGGCTGCTTCCACTT
TTTCCCTGGTTTTGGCAGAAACATGGCTGGCCTGGTTCACCACTCTGGAAACAGTCTGATAAGAGACACCGGCATACTCTGCGACATC
ATACAATGTTACTGGTTTCATTTTAATAACTAGTGCGAAAAAACCCCGCCGAAGCGGGGTTTTTTGCGTTAAAGCCGGTGACGGTAGT
CGCTGGGAGTCCGGTCAAATTGACGAGCGAAGACGCGAGAGAAGGTTTGCTGCGACACATAGCCTAAGTCCATGGCAATATCGAAAA
TCGGACGTTCTGTTGTCCGCAATTCAACGGCAGCCAACAACAAACGGCGTTGACGGATATAGTCTCCTAATGTTTGATGTGTGACGGT
ACGAAACATGCGCTGTAAATACCACTTCGAGTAACCACTTTTCTTCGCTACAACATCAATATTAAGAGGCTGATCAATATGCTCATCGA
TCCAAGCAATAAGGTCCTGGATGATTTTTTGGTGGCTCATTGAACCACCTCCGCCCCCTTGAGGAGCAAAATTCCAATTCAAGAACGG
TTTCTGCTCGAACGGGTCCTGAAACGAATCGACGAAATCGGAGGACTGGTACTGAAACTGAGGAACAAAGGCGTCATTTTGCAACAAA
CCAATGGAACTAAACATGGACGTGTCATCATTAAATGCCTCTAACGCGTCTTCCAGATTAATCCATTTGGGCTCACTCTCAACCTCCCA
GTTATCAGAACACGTGACGTCCGGTGAAATCACGTGACTTGAGCATCTTGAGTCCGACGTACTCATCTTTTCCGTCGGTTTCTCAGCC
GCCGCCGGTAAATACTTCTCCATTGTTCCTTTCTTATTGTATATCCGACACAAAACCCAATCATCAAGTCTTAAGTTGTTCTTCTTGTTG
GTAGAAGCAGATCGATCGACATTAGCGAGACGATACTCGTGCATAATCCAATTCGTTTTAATCCCTTTCGGAGCTTTTCCTGCGTAGAA
GACGAGTGCTTTCTTAATCCCTAACGTCTTCGGTTTTCCGATCGGTTTATCAGCTCCAGTCGCTTTCCAATAACCGGTTCCAGCTGCCC
GGTTTGGTCTCGACCCGTTTGGGTATTTCCGGTCTCTATGCGAGAAGAAGTACCATTCTTTCTCACCGTACAACGCCATTTCTGGAAG
CTCCCATGGATTGAATTTGTACAAGTCAATCTCTGCGATAACCGGAACGTTAATCGGTTCTGACGCACATCTCCGGCAAAGATAGAAC
TTGACAAGCTCTTCGTCCGTCGGATGAAATCGGAATCCCGCCGGCAAATTCAACTCCGCCTTCATTTTTGGAAAAGTACCTTGTTGTTT
TCTTGTGGATAAGATTCGATTTTGAGTTTTTATAAACCTTCGAATCGGAGGATCTTTGTGGTTTATCTCAAAGATTGAAATCGAGGAGAA
GAGAGCAAAGTCCATGTCGACAGATCCTTTCTCCTCTTTAGATCTTTTGAATTCCCAAAAAAACGGGTATGGAGAAACAGTAGAGAGTT
GCGATAAAAAGCGTCAGGTAGAATCCGCTAATCTTATGGATAAAAATGCTATGGCATAGCAAAGTGTGACGCCGTGCAAATAATCAAT
GTGGACTTTTCTGCCGTGATTATAGACACTTTTGTTACGCGTTTTTGTCATGGCTTTGGTCCCGCTTTGTTACAGAATGCTTTTAATAAG
CGGGGTTACCGGTTTGGTTAGCGAGAAGAGCCAGTAAAAGACGCAGTGACGGCAATGTCTGATGCAATATGGACAATTGGTTTCTTCT
CTGAATGGCGGGAGTATGAAAAGTTCAACATTTCCGTGTCGCCCTTATTCCCTTTTTTGCGGCATTTTGCCTTCCTGTTTTTGCTCACC
CAGAAACGCTGGTGAAAGTAAAAGATGCTGAAGATCAGTTGGGTGCACGAGTGGGTTACATCGAACTGGATCTCAACAGCGGTAAGA
TCCTTGAGAGTTTTCGCCCCGAAGAACGTTTTCCAATGATGAGCACTTTTAAAGTTCTGCTATGTGGCGCGGTATTATCCCGTATTGAC
GCCGGGCAAGAGCAACTCGGTCGCCGCATACACTATTCTCAGAATGACTTGGTTGAGTACTCACCAGTCACAGAAAAGCATCTTACG
GATGGCATGACAGTAAGAGAATTATGCAGTGCTGCCATAACCATGAGTGATAACACTGCGGCCAACTTACTTCTGACAACGATCGGAG
GACCGAAGGAGCTAACCGCTTTTTTGCACAACATGGGGGATCATGTAACTCGCCTTGATCGTTGGGAACCGGAGCTGAATGAAGCCA
TACCAAACGACGAGCGTGACACCACGATGCCTGTAGCAATGGCAACAACGTTGCGCAAACTATTAACTGGCGAACTACTTACTCTAGC
TTCCCGGCAACAATTAATAGACTGGATGGAGGCGGATAAAGTTGCAGGACCACTTCTGCGCTCGGCCCTTCCGGCTGGCTGGTTTAT

	TGCTGATAAATCTGGAGCCGGTGAGCGTGGGTCTCGCGGTATCATTGCAGCACTGGGGCCAGATGGTAAGCCCTCCCGTATCGTAGT<br>TATCTACACGACGGGGAGTCAGGCAACTATGGATGAACGAAATAGACAGATCGCTGAGATAGGTGCCTCACTGATTAAGCATTGGTAA<br>AATTATTAACGCTTACAATTTCCTGATGCGGTATTTTCTCCTTACGCATCTGTGCGGTATTTCACACCGCATCAGGTGGCACTTTTCGG<br>GGAAATGTGCGCGGAACCCCTATTTGTTTATTTTTCTAAATACATTCAAATATGTATCCGCTGGATCTCATGCTGGAGTTCTTCGCCCA<br>CCCTTAATTAACCGTAGAAAAGATCAAAGGATCTTCTTGAGATCCTTTTTTTCTGCGCGTAATCTGCTGCTTGCAAACAAAA	
pGRF9	AAACCACCGCTACCAGCGGTGGTTTGTTTGCCGGATCAAGAGCTACCAACTCTTTTTCCGAAGGTAACTGGCTTCAGCAGAGCGCAG<br>ATACCAAATACTGTTCTTCTAGTGTAGCCGTAGTTAGGCCACCACTTCAAGAACTCTGTAGCACCGCCTACATACCTCGCTCTGCTAAT<br>CCTGTTACCAGTGGCTGCTGCCAGTGGCGATAAGTCGTGTCTTACCGGGTTGGACTCAAGACGATAGTTACCGGATAAGGCGCAGCG<br>GTCGGGCTGAACGGGGGGTTCGTGCACACAGCCCAGCTTGGAGCGAACGACCTACACCGAACTGAGATACCTACAGCGTGAGCTAT<br>GAGAAAGCGCCACGCTTCCCGAAGGGAGAAAGGCGGACAGGTATCCGGTAAGCGGCAGGGTCGGAACAGGAGAGCGCACGAGGG<br>AGCTTCCAGGGGGAAACGCCTGGTATCTTTATAGTCCTGTCGGGTTTCGCCACCTCTGACTTGAGCGTCGATTTTTGTGATGCTCGTC<br>AGGGGGGCGGAGCCTATGGAAAAACGCCAGCAACGCGGCCTTTTTACGGTTCCTGGCCTTTTGCTGGCCTTTTGCTCATGATATAATT<br>AAATTGAAGCTCTAATTTGTGAGTTTAGTATACATGCATTTACTTATAATACAGTTTTTTAGTTTTGCTGGCCGCATCTTCTCAAATATGC<br>TTCCCAGCCTGCTTTTCTGTAACGTTCACCCTCTACCTTAGCATCCCTTCCCTTTGCAAATAGTCCTCTTCCAACAATAATAATGTCAGA<br>TCCTGTAGAGACCACATCATCCACGGTTCTATACTGTTGACCCAATGCGTCTCCCTTGTCATCTAAACCCACACCGGGTGTCATAATCA<br>ACCAATCGTAACCTTCATCTCTTCCACCCATGTCTCTTTGAGCAATAAAGCCGATAACAAAATCTTTGTCGCTCTTCGCAATGTCAACA<br>GTACCCTTAGTATATTCTCCAGTAGATAGGGAGCCCTTGCATGACAATTCTGCTAACATCAAAAGGCCTCTAGGTTCCTTTGTTACTTC<br>TTCTGCCGCCTGCTTCAAACCGCTAACAATACCTGGGCCCACCACACCGTGTGCATTCGTAATGTCTGCCCATTCTGCTATTCTGTATA<br>CACCCGCAGAGTACTGCAATTTGACTGTATTACCAATGTCAGCAAATTTTCTGTCTTCGAAGAGTAAAAAATTGTACTTGGCGGATAAT<br>GCCTTTAGCGGCTTAACTGTGCCCTCCATGGAAAAATCAGTCAAGATATCCACATGTGTTTTTAGTAAACAAATTTTGGGACCTAATGC<br>TTCAACTAACTCCAGTAATTCCTTGGTGGTACGAACATCCAATGAAGCACACAAGTTTGTTTGCTTTTCGTGCATGATATTAAATAGCTT<br>GGCAGCAACAGGACTAGGATGAGTAGCAGCACGTTCCTTATATGTAGCTTTCGACATGATTTATCTTCGTTTCCTGAGGTTTTTGTTCT<br>GTGCAGTTGGGTTAAGAATACTGGGCAATTTCATGTTTCTTCAACACTACATATGCGTATATATACCAATCTAAGTCTGTGCTCCTTCCT<br>TCGTTCTTCCTTCTGTTCGGAGATTACCGAATCAAAAAAATTTCAAAGAAACCGAAATCAAAAAAAAGAATAAAAAAAAAATGATGAATT<br>GAATTGAAAAGCTAGCTTATCGATGATAAGCTGTCAAAGATGAGAATTAATTCCACGGACTATAGACTATACTAGATACTCCGTCTACT<br>GTACGATACACTTCCGCTCAGGTCCTTGTCCTTTAACGAGGCCTTACCACTCTTTTGTTACTCTATTGATCCAGCTCAGCAAAGGCAGT<br>GTGATCTAAGATTCTATCTTCGCGATGTAGTAAAACTAGCTAGACCGAGAAAGAGACTAGAAATGCAAAAGGCACTTCTACAATGGCT<br>GCCATCATTATTATCCGATGTGACGCTGCAGCTTCTCAATGATATTCGAATACGCTTTGAGGAGATACAGCCTAATATCCGACAAACTG<br>TTTTACAGATTTACGATCGTACTTGTTACCCATCATTGAATTTTGAACATCCGAACCTGGGAGTTTTCCCTGAAACAGATAGTATATTTG<br>AACCTGTATAATAATATATAGTCTAGCGCTTTACGGAAGACAATGTATGTATTTCGGTTCCTGGAGAAACTATTGCATCTATTGCATAGG<br>TAATCTTGCACGTCGCATCCCCGGTTCATTTTCTGCGTTTCCATCTTGCACTTCAATAGCATATCTTTGTTAACGAAGCATCTGTGCTTC<br>ATTTTGTAGAACAAAAATGCAACGCGAGAGCGCTAATTTTTCAAACAAAGAATCTGAGCTGCATTTTTACAGAACAGAAATGCAACGCG<br>AAAGCGCTATTTTACCAACGAAGAATCTGTGCTTCATTTTTGTAAAACAAAAATGCAACGCGACGAGAGCGCTAATTTTTCAAACAAAG<br>AATCTGAGCTGCATTTTTACAGAACAGAAATGCAACGCGAGAGCGCTATTTTACCAACAAAGAATCTATACTTCTTTTTTGTTCTACAAA<br>AATGCATCCCGAGAGCGCTATTTTTCTAACAAAGCATCTTAGATTACTTTTTTTCTCCTTTGTGCGCTCTATAATGCAGTCTCTTGATAA<br>CTTTTTGCACTGTAGGTCCGTTAAGGTTAGAAGAAGGCTACTTTGGTGTCTATTTTCTCTTCCATAAAAAAAGCCTGACTCCACTTCCC<br>GCGTTTACTGATTACTAGCGAAGCTGCGGGTGCATTTTTTCAAGATAAAGGCATCCCCGATTATATTCTATACCGATGTGGATTGCGCA<br>TACTTTGTGAACAGAAAGTGATAGCGTTGATGATTCTTCATTGGTCAGAAAATTATGAACGGTTTCTTCTATTTTGTCTCTATATACTAC<br>GTATAGGAAATGTTTACATTTTCGTATTGTTTTCGATTCACTCTATGAATAGTTCTTACTACAATTTTTTTGTCTAAAGAGTAATACTAGA<br>GATAAACATAAAAAATGTAGAGGTCGAGTTTAGATGCAAGTTCAAGGAGCGAAAGGTGGATGGGTAGGTTATATAGGGATATAGCACA<br>GAGATATATAGCAAAGAGATACTTTTGAGCAATGTTTGTGGAAGCGGTATTCGCAATGGGAAGCTCCACCCCGGTTGATAATCAGAAA<br>AGCCCCAAAAACAGGAAGATTGTATAAGCAAATATTTAAATTGTAATTCTTTCCTGCGTTATCCCCTGATTCTGTGGATAACCGTATTAC<br>CGCCTTTGAGTGAGCTGATACCGCTCGCCGCAGCCGAACGACCGAGCGCAGCGAGTCAGTGAGCGAGGAAGCGGAAGAGGGTCTG<br>ACGCTCAGTGGAACGAAAACTCACGTTAAGGGATTTTGGTCATGAGATTATCAAAAAGGATCTTCACCTAGATCCTTTTAAATTAAAAAT<br>GAAGTTTTAAATCAATCTAAAGTATATATGAGTAAACTTGGTCTGACAGTCAGAAGAACTCGTCAAGAAGGCGATAGAAGGCGATGCG<br>CTGCGAATCGGGAGCGGCGATACCGTAAAGCACGAGGAAGCGGTCAGCCCATTCGCCGCCAAGCTCTTCAGCAATATCACGGGTAG<br>CCAACGCTATGTCCTGATAGCGGTCCGCCACACCCAGCCGGCCACAGTCGATGAATCCAGAAAAGCGGCCATTTTCCACCATGATAT<br>TCGGCAAGCAGGCATCGTCATGGGTCACGACGAGATCCTCGCCGTCGGGCATGCTCGCCTTGAGCCTGGCGAACAGTTCGGCTGGC<br>GCGAGCCCCTGATGCTCTTCGTCCAGATCATCCTGATCGACAAGACCGGCTTCCATCCGAGTACGTGCTCGCTCGATGCGATGTTTC<br>GCTTGGTGGTCGAATGGGCAGGTAGCCGGATCAAGCGTATGCAGCCGCCGCATTGCATCAGCCATGATGGATACTTTCTCGGCAGG<br>AGCAAGGTGAGATGACAGGAGATCCTGCCCCGGCACTTCGCCCAATAGCAGCCAGTCCCTTCCCGCTTCAGTGACAACGTCGAGCA<br>CAGCTGCGCAAGGAACGCCCGTCGTGGCCAGCCACGATAGCCGCGCTGCCTCGTCTTGCAGTTCATTCAGGGCACCGGACAGGTCG<br>GTCTTGACAAAAAGAACCGGGCGCCCCTGCGCTGACAGCCGGAACACGGCGGCATCAGAGCAGCCGATTGTCTGTTGTGCCCAGTC<br>ATAGCCGAATAGCCTCTCCACCCAAGCGGCCGGAGAACCTGCGTGCAATCCATCTTGTTCAATCATACTCTTCCTTTTTCAATATTATT<br>GAAGCATTTATCAGGGTTATTGTCTCATGAGCGGATACATATTTGAATGTATTTAGAAAAATAAACAAATAGGGGTTCCGCGCACATTT<br>CCCCGAAAAGTGCCACCTGCGGACGGATCGCTTGCCTGTAACTTACACGCGCCTCGTCCTGAGAAAGCAACCTGACCTACAGGAAAG<br>AGTTACTCAAGAATAAGAATTTTCGTTTTAAAACCTAAGAGTCACTTTAAAATTTGTATACACTTATTTTTTTTATAACTTATTTAATAATAA	This study

	AAATCATAAATCATAAGAAATTCGCTTATTTAGAAGTGTCAACAACGTATCTACCAACGATTTGACCCTTTTCCATCTTTTCGTAAATTTC<br>TGGCAAGGTAGACAAGCCGACAACCTTGATTGGAGACTTGACCAAACCTCTGGCGAAGAATTGTTAATATTAAGAGCTCTCACTGCCC<br>ACTTTCCAGTCTGGAAACCTGTCTTGCTAGCTGCATGAGTGAATCAGCCAATGCCCGGGGAGAGGCAGTTTGTGTATTGGGTGCCAG<br>GGTGGTTTTTCTCTTCACCAGTGAGACTGGCAACAGCTGATTGCCCTTCACCGCCTGGCCCTGAGAGAGTTGCAGCAAGCGGTCCAC<br>GCTGGTTTGCCCCAGCAGGCGAAAATCCTGTTTGATGGTGGTTAACGGCGGGATATAACATGAGCTGTCTTCGGTATCGTCGTATCC<br>CACTACCGAGATATCTGCACCAACTCTCAGCCCAGACTCAGTAATGGCTCTCATTGCACCCAGTGCCATCTGATCATTGGCAACCAGC<br>ATTGCAGTGGGAACAATGCCCTCATTCAGCATTTGCATGGTTTGTTGAAACCCAGACATGGCACTCCAGTCCCCTTCTCTTTCAGCTAT<br>TGGCTGAATTTGATTCCTAGTGAGATATTTATGCCAGCCGGCCAGTCTCAGCCTTGCTGAGACAGAACTGAGTGGGCCCGCAAGCAG<br>TGCAATTTGCTGGTGTCCCAATGCAACCAGATGCTCCACACCCAGTCTTGTACCATCTTCATGGGAGAAAATAATACTGTTGATGGGT<br>GTCTGGTCAGAGACATCAAGAAAGAGTGCTGGAACATTAGTGCAGGCAGCTTCCACAGCAATGGCATCCTGGTCATCCAGTGGATAG<br>TTAATGATCAGCCCACTGACTCTTTGTGCCAGAAGATTGTGCACAGCAGCTTTACAGGCTTCAACTCCACTTCTTTCTACCATTGACAC<br>CACCACAGAGGCTCCCAGTTGATCAGCTCTAGATTTAATGGCTGCCACAATTTGAGATGGTGCATGCAGGGCCAGACTGGAGGTGGC<br>AACTCCAATCAGCAAGCTCTGTTTGCCTGCCAGTTGTTGTGCCACTCTGTTGGGAATGTAATTCAGCTCTGCCATGGCTGCTTCCACTT<br>TTTCCCTGGTTTTGGCAGAAACATGGCTGGCCTGGTTCACCACTCTGGAAACAGTCTGATAAGAGACACCGGCATACTCTGCGACATC<br>ATACAATGTTACTGGTTTCATTTTAATAACTAGTGCGAAAAAACCCCGCCGAAGCGGGGTTTTTTGCGTTAAAGCCGGTGACGGTAGT<br>CGCTGGGAGTCCGGTCAAATTGACGAGCGAAGACGCGAGAGAAGGTTTGCTGCGACACATAGCCTAAGTCCATGGCAATATCGAAAA<br>TCGGACGTTCTGTTGTCCGCAATTCAACGGCAGCCAACAACAAACGGCGTTGACGGATATAGTCTCCTAATGTTTGATGTGTGACGGT<br>ACGAAACATGCGCTGTAAATACCACTTCGAGTAACCACTTTTCTTCGCTACAACATCAATATTAAGAGGCTGATCAATATGCTCATCGA<br>TCCAAGCAATAAGGTCCTGGATGATTTTTTGGTGGCTCATTGAACCACCTCCGCCAACACCTGGTGAAAACAAAGACAAGTACTCCTC<br>ATCGTCGTCTTCACCCCTTAAAGCTGTGTCAGTAATGGTAGTGTCGGTACTGCAACTGCTAGTGCTCTTCTCATCAGAAACTCGGGGC<br>AGTGTAATTCCCATGACTGAAACAGAATGCTTCTGGTCATCTCCATCTTTGCAAGGCACAACCGATCTAACAGCTGTTTCCACGGTTAA<br>CGGGCTAAACCCGGCATTCTCATGTGAGTTAGTAGTATCAACTGGATGCTTCTTCTTCATACCTCTATGCATGTGCTTATCGCAGTACT<br>TCTGACCAGACAAAACATCTTTGCTGCATCTCCACTTCTTCCCATCTGTTCTTCGGCACCTTCCTGGTTCATTGTCTGTTTCAACTCCTT<br>GTAAACGAAAACAACCACGTTCTTGAAGAACAGTGTCTGGAGAAAAGTGGATACCATGAAACAAACCACCATTAACATTCATATTCTTC<br>CATTCTCTGCTTCTTGCAATTATATCATTCTTCCTGTTGCTTGAGAATTTTGCAGCTTCTAAGCATTTATCAGATACCTCAGGGAAGAAC<br>TTTTGAACCGAAGGTTTGCTCTCATCACCTTGAAGAGGAACAACTCCAACACACATACTCGCCTCTTTTTCATTAATAAAGTCATCATAA<br>CTCATCTCCATGTTCTTTCTGCCGTGACTCACACTTTTGTTTGTGAATTCAGAGATCACTAAAGACGGACGAATGACATTTTCACAGGA<br>TCTGGCACTAGGCAGTGAAGCAATGGTAACTACCTGCGCATTACTAGAACCCGAGATTGTCCCATGAAGATGACTCTGACTCTCGTTA<br>TACCTATCCAAACCATAAGTGTTGTCGTATTTGGTTGATGATGAAGCAACCTCAGAAGAAGATTCCACAAGCTTTCTTGAACGTTTACG<br>ACCTCTATGCATGTGCCGTTCACAGTACTTCTCGAATAGAAGGACCGTGTTGCTACAGCGCCATTTCTTCCCATCTGTTCTCCTGCACC<br>TAGTTGGTTCAGTTTCCAACGTGTCGATATGGGTTACTCCCTTGTTACTCAACAGAGAAGAAGAGTGATAGTTGTAATTGGAGGAAGAA<br>GAGAGAGCAAGACTGTTCCAAATAGGCACCACGAGATGATGAGGCACACGGAGACCAGCCTCTATGTATCTATACACCAAAGCTTGC<br>ATCCGAAACTCCATTAACTGCGCCGCCTTCATCCACGGCCACTTATTCCTCATCCTCTCCTCCTCAACCTCCTCCTGCTCCATTTTAGG<br>GCTCTGCATGTCGACAGATCCTTTCTCCTCTTTAGATCTTTTGAATTCCCAAAAAAACGGGTATGGAGAAACAGTAGAGAGTTGCGATA<br>AAAAGCGTCAGGTAGAATCCGCTAATCTTATGGATAAAAATGCTATGGCATAGCAAAGTGTGACGCCGTGCAAATAATCAATGTGGAC<br>TTTTCTGCCGTGATTATAGACACTTTTGTTACGCGTTTTTGTCATGGCTTTGGTCCCGCTTTGTTACAGAATGCTTTTAATAAGCGGGGT<br>TACCGGTTTGGTTAGCGAGAAGAGCCAGTAAAAGACGCAGTGACGGCAATGTCTGATGCAATATGGACAATTGGTTTCTTCTCTGAAT<br>GGCGGGAGTATGAAAAGTTCAACATTTCCGTGTCGCCCTTATTCCCTTTTTTGCGGCATTTTGCCTTCCTGTTTTTGCTCACCCAGAAA<br>CGCTGGTGAAAGTAAAAGATGCTGAAGATCAGTTGGGTGCACGAGTGGGTTACATCGAACTGGATCTCAACAGCGGTAAGATCCTTG<br>AGAGTTTTCGCCCCGAAGAACGTTTTCCAATGATGAGCACTTTTAAAGTTCTGCTATGTGGCGCGGTATTATCCCGTATTGACGCCGG<br>GCAAGAGCAACTCGGTCGCCGCATACACTATTCTCAGAATGACTTGGTTGAGTACTCACCAGTCACAGAAAAGCATCTTACGGATGGC<br>ATGACAGTAAGAGAATTATGCAGTGCTGCCATAACCATGAGTGATAACACTGCGGCCAACTTACTTCTGACAACGATCGGAGGACCGA<br>AGGAGCTAACCGCTTTTTTGCACAACATGGGGGATCATGTAACTCGCCTTGATCGTTGGGAACCGGAGCTGAATGAAGCCATACCAAA<br>CGACGAGCGTGACACCACGATGCCTGTAGCAATGGCAACAACGTTGCGCAAACTATTAACTGGCGAACTACTTACTCTAGCTTCCCG<br>GCAACAATTAATAGACTGGATGGAGGCGGATAAAGTTGCAGGACCACTTCTGCGCTCGGCCCTTCCGGCTGGCTGGTTTATTGCTGA<br>TAAATCTGGAGCCGGTGAGCGTGGGTCTCGCGGTATCATTGCAGCACTGGGGCCAGATGGTAAGCCCTCCCGTATCGTAGTTATCTA<br>CACGACGGGGAGTCAGGCAACTATGGATGAACGAAATAGACAGATCGCTGAGATAGGTGCCTCACTGATTAAGCATTGGTAAAATTAT<br>TAACGCTTACAATTTCCTGATGCGGTATTTTCTCCTTACGCATCTGTGCGGTATTTCACACCGCATCAGGTGGCACTTTTCGGGGAAAT<br>GTGCGCGGAACCCCTATTTGTTTATTTTTCTAAATACATTCAAATATGTATCCGCTGGATCTCATGCTGGAGTTCTTCGCCCACCCTTAA<br>TTAACCGTAGAAAAGATCAAAGGATCTTCTTGAGATCCTTTTTTTCTGCGCGTAATCTGCTGCTTGCAAACAAAA	
pUC57-BsaI-free_ JUB2x	TCGCGCGTTTCGGTGATGACGGTGAAAACCTCTGACACATGCAGCTCCCGGAGACGGTCACAGCTTGTCTGTAAGCGGATGCCGGG<br>AGCAGACAAGCCCGTCAGGGCGCGTCAGCGGGTGTTGGCGGGTGTCGGGGCTGGCTTAACTATGCGGCATCAGAGCAGATTGTACT<br>GAGAGTGCACCATATGCGGTGTGAAATACCGCACAGATGCGTAAGGAGAAAATACCGCATCAGGCGCCATTCGCCATTCAGGCTGCG<br>CAACTGTTGGGAAGGGCGATCGGTGCGGGCCTCTTCGCTATTACGCCAGCTGGCGAAAGGGGGATGTGCTGCAAGGCGATTAAGTT<br>GGGTAACGCCAGGGTTTTCCCAGTCACGACGTTGTAAAACGACGGCCAGTGAATTCGAGCTCGGTACCTCGCGAATGCATCTAGACT<br>CGAGGATCCCGGGTACCTGCAGCTAGCGTCGACTGTGGTCTCTACAGGAATTCTTTCTTCTGTCGCCGTCTTGTCAACGGCACCAGT<br>TATATATTTCTTCTGTCGCCGTCTTGTCAACGGCACCAGTTATATATTGGGTTCCACCGGATACCTCCGGACTTGACAGCTAGCTCAGT	This study

	CCTAGGGATTGTGCTAGCGAATTCATTAAAGAGGAGAAAGGTACCATGTCTAAAGGTGAAGAACTGTTCACCGGTGTTGTTCCGATCC<br>TGGTTGAACTGGATGGTGATGTTAACGGCCACAAATTCTCTGTTCGTGGTGAAGGTGAAGGTGATGCAACCAACGGTAAACTGACCCT<br>GAAATTCATCTGCACTACCGGTAAACTGCCGGTTCCATGGAAGCTTGGCGTAATCATGGTCATAGCTGTTTCCTGTGTGAAATTGTTAT<br>CCGCTCACAATTCCACACAACATACGAGCCGGAAGCATAAAGTGTAAAGCCTGGGGTGCCTAATGAGTGAGCTAACTCACATTAATTG<br>CGTTGCGCTCACTGCCCGCTTTCCAGTCGGGAAACCTGTCGTGCCAGCTGCATTAATGAATCGGCCAACGCGCGGGGAGAGGCGGT<br>TTGCGTATTGGGCGCTCTTCCGCTTCCTCGCTCACTGACTCGCTGCGCTCGGTCGTTCGGCTGCGGCGAGCGGTATCAGCTCACTCA<br>AAGGCGGTAATACGGTTATCCACAGAATCAGGGGATAACGCAGGAAAGAACATGTGAGCAAAAGGCCAGCAAAAGGCCAGGAACCG<br>TAAAAAGGCCGCGTTGCTGGCGTTTTTCCATAGGCTCCGCCCCCCTGACGAGCATCACAAAAATCGACGCTCAAGTCAGAGGTGGCG<br>AAACCCGACAGGACTATAAAGATACCAGGCGTTTCCCCCTGGAAGCTCCCTCGTGCGCTCTCCTGTTCCGACCCTGCCGCTTACCGG<br>ATACCTGTCCGCCTTTCTCCCTTCGGGAAGCGTGGCGCTTTCTCATAGCTCACGCTGTAGGTATCTCAGTTCGGTGTAGGTCGTTCGC<br>TCCAAGCTGGGCTGTGTGCACGAACCCCCCGTTCAGCCCGACCGCTGCGCCTTATCCGGTAACTATCGTCTTGAGTCCAACCCGGTA<br>AGACACGACTTATCGCCACTGGCAGCAGCCACTGGTAACAGGATTAGCAGAGCGAGGTATGTAGGCGGTGCTACAGAGTTCTTGAAG<br>TGGTGGCCTAACTACGGCTACACTAGAAGAACAGTATTTGGTATCTGCGCTCTGCTGAAGCCAGTTACCTTCGGAAAAAGAGTTGGTA<br>GCTCTTGATCCGGCAAACAAACCACCGCTGGTAGCGGTGGTTTTTTTGTTTGCAAGCAGCAGATTACGCGCAGAAAAAAAGGATCTCA<br>AGAAGATCCTTTGATCTTTTCTACGGGGTCTGACGCTCAGTGGAACGAAAACTCACGTTAAGGGATTTTGGTCATGAGATTATCAAAAA<br>GGATCTTCACCTAGATCCTTTTAAATTAAAAATGAAGTTTTAAATCAATCTAAAGTATATATGAGTAAACTTGGTCTGACAGTTACCAATG<br>CTTAATCAGTGAGGCACCTATCTCAGCGATCTGTCTATTTCGTTCATCCATAGTTGCCTGACTCCCCGTCGTGTAGATAACTACGATAC<br>GGGAGGGCTTACCATCTGGCCCCAGTGCTGCAATGATACCGCGAGACTCACGCTCACCGGCTCCAGATTTATCAGCAATAAACCAGC<br>CAGCCGGAAGGGCCGAGCGCAGAAGTGGTCCTGCAACTTTATCCGCCTCCATCCAGTCTATTAATTGTTGCCGGGAAGCTAGAGTAA<br>GTAGTTCGCCAGTTAATAGTTTGCGCAACGTTGTTGCCATTGCTACAGGCATCGTGGTGTCACGCTCGTCGTTTGGTATGGCTTCATT<br>CAGCTCCGGTTCCCAACGATCAAGGCGAGTTACATGATCCCCCATGTTGTGCAAAAAAGCGGTTAGCTCCTTCGGTCCTCCGATCGTT<br>GTCAGAAGTAAGTTGGCCGCAGTGTTATCACTCATGGTTATGGCAGCACTGCATAATTCTCTTACTGTCATGCCATCCGTAAGATGCTT<br>TTCTGTGACTGGTGAGTACTCAACCAAGTCATTCTGAGAATAGTGTATGCGGCGACCGAGTTGCTCTTGCCCGGCGTCAATACGGGAT<br>AATACCGCGCCACATAGCAGAACTTTAAAAGTGCTCATCATTGGAAAACGTTCTTCGGGGCGAAAACTCTCAAGGATCTTACCGCTGT<br>TGAGATCCAGTTCGATGTAACCCACTCGTGCACCCAACTGATCTTCAGCATCTTTTACTTTCACCAGCGTTTCTGGGTGAGCAAAAACA<br>GGAAGGCAAAATGCCGCAAAAAAGGGAATAAGGGCGACACGGAAATGTTGAATACTCATACTCTTCCTTTTTCAATATTATTGAAGCAT<br>TTATCAGGGTTATTGTCTCATGAGCGGATACATATTTGAATGTATTTAGAAAAATAAACAAATAGGGGTTCCGCGCACATTTCCCCGAA<br>AAGTGCCACCTGACGTCTAAGAAACCATTATTATCATGACATTAACCTATAAAAATAGGCGTATCACGAGGCCCTTTCGTC	
pKH70-PrpsM-sfGFP	CTCGAGGATCCCGGGTACCTGCAGCTAGCGTCGACGAAAGGCTACGGCCGTTAATTGGTCGCCTGAGAAGTTACGGAGAGTAAAAAT<br>GAAAGTTCGTGCTTCCGTCAAGAAATTATGCCGTAACTGCAAAATCGTTAAGCGTGATGGTGTCATCCGTGTGATTTGCAGTGCCGAG<br>CCGAAGCATAAACAGCGCCAAGGCTGATTTTTTCGCATATTTTTCTTGCAAAGTTGGGTTGAGCTGGCTAGATTAGCCAGCCAATCTTT<br>TGTATGTCTGTACGTTTCCATTTGAGTATCCTGAAAACGGGCTTTTCAGCATGGTACGTACATATTAAATAGTAGGAGTGCATAGTGGC<br>CCGTATAGCAGGCATTAACATTCCTGATCAGAAACACGCCGTGATCGCGTTAACTTCGATCTACGGTGTCGGCAAGACCCGTTCTAAA<br>GCCATCCTGGCTGGAATTCATAAGGAGGAAAAACATATGTCTAAAGGTGAAGAACTGTTCACCGGTGTTGTTCCGATCCTGGTTGAAC<br>TGGATGGTGATGTTAACGGCCACAAATTCTCTGTTCGTGGTGAAGGTGAAGGTGATGCAACCAACGGTAAACTGACCCTGAAATTCAT<br>CTGCACTACCGGTAAACTGCCGGTTCCATGGCCGACTCTGGTGACTACCCTGACCTATGGTGTTCAGTGTTTTTCTCGTTACCCGGAT<br>CACATGAAGCAGCATGATTTCTTCAAATCTGCAATGCCGGAAGGTTATGTACAGGAGCGCACCATTTCTTTCAAAGACGATGGCACCT<br>ACAAAACCCGTGCAGAGGTTAAATTTGAAGGTGATACTCTGGTGAACCGTATTGAACTGAAAGGCATTGATTTCAAAGAGGACGGCAA<br>CATCCTGGGCCACAAACTGGAATATAACTTCAACTCCCATAACGTTTACATCACCGCAGACAAACAGAAGAACGGTATCAAAGCTAACT<br>TCAAAATTCGCCATAACGTTGAAGACGGTAGCGTACAGCTGGCGGACCACTACCAGCAGAACACTCCGATCGGTGATGGTCCGGTTC<br>TGCTGCCGGATAACCACTACCTGTCCACCCAGTCTGTTCTGTCCAAAGACCCGAACGAAAAGCGCGACCACATGGTGCTGCTGGAGT<br>TCGTTACTGCAGCAGGTATCACGCACGGCATGGATGAGCTCTACAAATGATAAGCGGCCGCTCTAGAGGCATCAAATAAAACGAAAG<br>GCTCAGTCGAAAGACTGGGCCTTTCGTTTTATCTGTTGTTTGTCGGTGAACGCTCTCCTGAGTAGGACAAATCCGCCGCCCTAGACCT<br>AGGCGGAGATATTCCGCTTCCTCGCTCACTGACTCGCTCCGCTCGGTCGTTCGGCTGCGGCGAGCGGTACCGGCTTACTGGCGGGG<br>CGGAAATTTCCTGGAAGATGCCAGGAAGAAACTTAACAGGGAAGCGATAAGGCCACGGCAGAGCCGTTTTTCCATAGGCTCCGCCCC<br>CCTGACAAGCATCACGAAATCTGACGCTCAAATCAGTGGTGGCGAAACCCGACAGGACTTAAAGATACCAGGCGTTTCCCCCTGGTG<br>GCTCCCTCGTGCGCTCTCCTGTTCCTGCCTTTCGGTTTACCGGTGTCACTCCGCTGTTATGGCCGCGTTTATCTCATTCCACGCCTGA<br>CACTCGGTTCCGGGAAGGCAGTTCGCTCCAAGCTGGACTGTATGCACGAACCCCCCGTTCAGTCCGACTGCTGCGCCTGTTCCGGT<br>AACTATCGACTTGAGTCCAAGCCGGAAAGACACGACAAAGCGCCACTGGCAGCAGCCACTGGTAAGAGGGTGTACAAGAGATTACTG<br>ATGCAGAGTTCTTGAAGTCAGAGCCAAACTACGGCTAAACTGGAAGGACGAATTTGGTGGCAGCGGTCTTGTACACCCTGTTACCAC<br>GGTTCAGAAGTTCCCCAACTTACTGAACCTTCGAAAAACCACCTCCCCAGGTGGTTTTTTCGTTTTCAGAGCAAGAGATTACGACGCA<br>ACGGAAAGGATCTCAAGAGGTTCATCTTACTAGTGCTTGGATTCTCACCAATAAAAAACGCCCGGCGGCAACCGAGCGTTCTGAACAA<br>ATCCAGATGGAGTTCTGAGGTCATTACTGGATCTATCAACAGGAGTCCAAGCGAGCTCGTAAACTTGGTCTGACAGTTACCAATGCTT<br>AATCAGTGAGGCACCTATCTCAGCGATCTGTCTATTTCGTTCATCCATAGTTGCCTGACTCCCCGTCGTGTAGATAACTACGATACGG<br>GAGGGCTTACCATCTGGCCCCAGTGCTGCAATGATACCGCGAGACCCACGCTCACCGGCTCCAGATTTATCAGCAATAAACCAGCCA<br>GCCGGAAGGGCCGAGCGCAGAAGTGGTCCTGCAACTTTATCCGCCTCCATCCAGTCTATTAATTGTTGCCGGGAAGCTAGAGTAAGT<br>AGTTCGCCAGTTAATAGTTTGCGCAACGTTGTTGCCATTGCTACAGGCATCGTGGTGTCACGCTCGTCGTTTGGTATGGCTTCATTCA	Lab collection

	GCTCCGGTTCCCAACGATCAAGGCGAGTTACATGATCCCCCATGTTGTGCAAAAAAGCGGTTAGCTCCTTCGGTCCTCCGATCGTTGT<br>CAGAAGTAAGTTGGCCGCAGTGTTATCACTCATGGTTATGGCAGCACTGCATAATTCTCTTACTGTCATGCCATCCGTAAGATGCTTTT<br>CTGTGACTGGTGAGTACTCAACCAAGTCATTCTGAGAATAGTGTATGCGGCGACCGAGTTGCTCTTGCCCGGCGTCAATACGGGATA<br>ATACCGCGCCACATAGCAGAACTTTAAAAGTGCTCATCATTGGAAAACGTTCTTCGGGGCGAAAACTCTCAAGGATCTTACCGCTGTT<br>GAGATCCAGTTCGATGTAACCCACTCGTGCACCCAACTGATCTTCAGCATCTTTTACTTTCACCAGCGTTTCTGGGTGAGCAAAAACA<br>GGAAGGCAAAATGCCGCAAAAAAGGGAATAAGGGCGACACGGAAATGTTGAATACTCATACTCTTCCTTTTTCAATATTATTGAAGCAT<br>TTATCAGGGTTATTGTCTCATGAGCGGATACATATTTGAATGTATTTAGAAAAATAAACAAATAGGGGTTCCGCGCACATTTCCCCGAA<br>AAGTGCCACCTGACGTCTAAGAAACCATTATTATCATGACATTAACCTATAAAAATAGGCGTATCACGAGGCCCTTTCGTCTTCAC	
pJUB2X	ATCACGAGGCCCTTTCGTCTTCACCTCGAGGATCCCGGGTACCTGCAGCTAGCGTCGACTGTGGTCTCTACAGGAATTCTTTCTTCTG<br>TCGCCGTCTTGTCAACGGCACCAGTTATATATTTCTTCTGTCGCCGTCTTGTCAACGGCACCAGTTATATATTGGTGTTCCACCGGATA<br>CCTCCGGACTTGACAGCTAGCTCAGTCCTAGGGATTGTGCTAGCGAATTCATTAAAGAGGAGAAAGGTACCATGTCTAAAGGTGAAGA<br>ACTGTTCACCGGTGTTGTTCCGATCCTGGTTGAACTGGATGGTGATGTTAACGGCCACAAATTCTCTGTTCGTGGTGAAGGTGAAGGT<br>GATGCAACCAACGGTAAACTGACCCTGAAATTCATCTGCACTACCGGTAAACTGCCGGTTCCATGGCCGACTCTGGTGACTACCCTGA<br>CCTATGGTGTTCAGTGTTTTTCTCGTTACCCGGATCACATGAAGCAGCATGATTTCTTCAAATCTGCAATGCCGGAAGGTTATGTACAG<br>GAGCGCACCATTTCTTTCAAAGACGATGGCACCTACAAAACCCGTGCAGAGGTTAAATTTGAAGGTGATACTCTGGTGAACCGTATTG<br>AACTGAAAGGCATTGATTTCAAAGAGGACGGCAACATCCTGGGCCACAAACTGGAATATAACTTCAACTCCCATAACGTTTACATCACC<br>GCAGACAAACAGAAGAACGGTATCAAAGCTAACTTCAAAATTCGCCATAACGTTGAAGACGGTAGCGTACAGCTGGCGGACCACTAC<br>CAGCAGAACACTCCGATCGGTGATGGTCCGGTTCTGCTGCCGGATAACCACTACCTGTCCACCCAGTCTGTTCTGTCCAAAGACCCG<br>AACGAAAAGCGCGACCACATGGTGCTGCTGGAGTTCGTTACTGCAGCAGGTATCACGCACGGCATGGATGAGCTCTACAAATGATAA<br>GCGGCCGCTCTAGAGGCATCAAATAAAACGAAAGGCTCAGTCGAAAGACTGGGCCTTTCGTTTTATCTGTTGTTTGTCGGTGAACGCT<br>CTCCTGAGTAGGACAAATCCGCCGCCCTAGACCTAGGCGGAGATATTCCGCTTCCTCGCTCACTGACTCGCTCCGCTCGGTCGTTCG<br>GCTGCGGCGAGCGGTACCGGCTTACTGGCGGGGCGGAAATTTCCTGGAAGATGCCAGGAAGAAACTTAACAGGGAAGCGATAAGGC<br>CACGGCAGAGCCGTTTTTCCATAGGCTCCGCCCCCCTGACAAGCATCACGAAATCTGACGCTCAAATCAGTGGTGGCGAAACCCGAC<br>AGGACTTAAAGATACCAGGCGTTTCCCCCTGGTGGCTCCCTCGTGCGCTCTCCTGTTCCTGCCTTTCGGTTTACCGGTGTCACTCCG<br>CTGTTATGGCCGCGTTTATCTCATTCCACGCCTGACACTCGGTTCCGGGAAGGCAGTTCGCTCCAAGCTGGACTGTATGCACGAACC<br>CCCCGTTCAGTCCGACTGCTGCGCCTGTTCCGGTAACTATCGACTTGAGTCCAAGCCGGAAAGACACGACAAAGCGCCACTGGCAG<br>CAGCCACTGGTAAGAGGGTGTACAAGAGATTACTGATGCAGAGTTCTTGAAGTCAGAGCCAAACTACGGCTAAACTGGAAGGACGAA<br>TTTGGTGGCAGCGGTCTTGTACACCCTGTTACCACGGTTCAGAAGTTCCCCAACTTACTGAACCTTCGAAAAACCACCTCCCCAGGTG<br>GTTTTTTCGTTTTCAGAGCAAGAGATTACGACGCAACGGAAAGGATCTCAAGAGGTTCATCTTACTAGTGCTTGGATTCTCACCAATAA<br>AAAACGCCCGGCGGCAACCGAGCGTTCTGAACAAATCCAGATGGAGTTCTGAGGTCATTACTGGATCTATCAACAGGAGTCCAAGCG<br>AGCTCGTAAACTTGGTCTGACAGTTACCAATGCTTAATCAGTGAGGCACCTATCTCAGCGATCTGTCTATTTCGTTCATCCATAGTTGC<br>CTGACTCCCCGTCGTGTAGATAACTACGATACGGGAGGGCTTACCATCTGGCCCCAGTGCTGCAATGATACCGCGAGACCCACGCTC<br>ACCGGCTCCAGATTTATCAGCAATAAACCAGCCAGCCGGAAGGGCCGAGCGCAGAAGTGGTCCTGCAACTTTATCCGCCTCCATCCA<br>GTCTATTAATTGTTGCCGGGAAGCTAGAGTAAGTAGTTCGCCAGTTAATAGTTTGCGCAACGTTGTTGCCATTGCTACAGGCATCGTG<br>GTGTCACGCTCGTCGTTTGGTATGGCTTCATTCAGCTCCGGTTCCCAACGATCAAGGCGAGTTACATGATCCCCCATGTTGTGCAAAA<br>AAGCGGTTAGCTCCTTCGGTCCTCCGATCGTTGTCAGAAGTAAGTTGGCCGCAGTGTTATCACTCATGGTTATGGCAGCACTGCATAA<br>TTCTCTTACTGTCATGCCATCCGTAAGATGCTTTTCTGTGACTGGTGAGTACTCAACCAAGTCATTCTGAGAATAGTGTATGCGGCGAC<br>CGAGTTGCTCTTGCCCGGCGTCAATACGGGATAATACCGCGCCACATAGCAGAACTTTAAAAGTGCTCATCATTGGAAAACGTTCTTC<br>GGGGCGAAAACTCTCAAGGATCTTACCGCTGTTGAGATCCAGTTCGATGTAACCCACTCGTGCACCCAACTGATCTTCAGCATCTTTT<br>ACTTTCACCAGCGTTTCTGGGTGAGCAAAAACAGGAAGGCAAAATGCCGCAAAAAAGGGAATAAGGGCGACACGGAAATGTTGAATA<br>CTCATACTCTTCCTTTTTCAATATTATTGAAGCATTTATCAGGGTTATTGTCTCATGAGCGGATACATATTTGAATGTATTTAGAAAAATA<br>AACAAATAGGGGTTCCGCGCACATTTCCCCGAAAAGTGCCACCTGACGTCTAAGAAACCATTATTATCATGACATTAACCTATAAAAAT<br>AGGCGT	This study
pJUB0X	ATCACGAGGCCCTTTCGTCTTCACCTCGAGGATCCCGGGTACCTGCAGCTAGCTGTTCCACCGGATACCTCCGGACTTGACAGCTAG<br>CTCAGTCCTAGGGATTGTGCTAGCGAATTCATTAAAGAGGAGAAAGGTACCATGTCTAAAGGTGAAGAACTGTTCACCGGTGTTGTTC<br>CGATCCTGGTTGAACTGGATGGTGATGTTAACGGCCACAAATTCTCTGTTCGTGGTGAAGGTGAAGGTGATGCAACCAACGGTAAACT<br>GACCCTGAAATTCATCTGCACTACCGGTAAACTGCCGGTTCCATGGCCGACTCTGGTGACTACCCTGACCTATGGTGTTCAGTGTTTT<br>TCTCGTTACCCGGATCACATGAAGCAGCATGATTTCTTCAAATCTGCAATGCCGGAAGGTTATGTACAGGAGCGCACCATTTCTTTCAA<br>AGACGATGGCACCTACAAAACCCGTGCAGAGGTTAAATTTGAAGGTGATACTCTGGTGAACCGTATTGAACTGAAAGGCATTGATTTC<br>AAAGAGGACGGCAACATCCTGGGCCACAAACTGGAATATAACTTCAACTCCCATAACGTTTACATCACCGCAGACAAACAGAAGAACG<br>GTATCAAAGCTAACTTCAAAATTCGCCATAACGTTGAAGACGGTAGCGTACAGCTGGCGGACCACTACCAGCAGAACACTCCGATCG<br>GTGATGGTCCGGTTCTGCTGCCGGATAACCACTACCTGTCCACCCAGTCTGTTCTGTCCAAAGACCCGAACGAAAAGCGCGACCACA<br>TGGTGCTGCTGGAGTTCGTTACTGCAGCAGGTATCACGCACGGCATGGATGAGCTCTACAAATGATAAGCGGCCGCTCTAGAGGCAT<br>CAAATAAAACGAAAGGCTCAGTCGAAAGACTGGGCCTTTCGTTTTATCTGTTGTTTGTCGGTGAACGCTCTCCTGAGTAGGACAAATC<br>CGCCGCCCTAGACCTAGGCGGAGATATTCCGCTTCCTCGCTCACTGACTCGCTCCGCTCGGTCGTTCGGCTGCGGCGAGCGGTACC<br>GGCTTACTGGCGGGGCGGAAATTTCCTGGAAGATGCCAGGAAGAAACTTAACAGGGAAGCGATAAGGCCACGGCAGAGCCGTTTTT<br>CCATAGGCTCCGCCCCCCTGACAAGCATCACGAAATCTGACGCTCAAATCAGTGGTGGCGAAACCCGACAGGACTTAAAGATACCAG	This study

	GCGTTTCCCCCTGGTGGCTCCCTCGTGCGCTCTCCTGTTCCTGCCTTTCGGTTTACCGGTGTCACTCCGCTGTTATGGCCGCGTTTAT<br>CTCATTCCACGCCTGACACTCGGTTCCGGGAAGGCAGTTCGCTCCAAGCTGGACTGTATGCACGAACCCCCCGTTCAGTCCGACTGC<br>TGCGCCTGTTCCGGTAACTATCGACTTGAGTCCAAGCCGGAAAGACACGACAAAGCGCCACTGGCAGCAGCCACTGGTAAGAGGGT<br>GTACAAGAGATTACTGATGCAGAGTTCTTGAAGTCAGAGCCAAACTACGGCTAAACTGGAAGGACGAATTTGGTGGCAGCGGTCTTGT<br>ACACCCTGTTACCACGGTTCAGAAGTTCCCCAACTTACTGAACCTTCGAAAAACCACCTCCCCAGGTGGTTTTTTCGTTTTCAGAGCAA<br>GAGATTACGACGCAACGGAAAGGATCTCAAGAGGTTCATCTTACTAGTGCTTGGATTCTCACCAATAAAAAACGCCCGGCGGCAACC<br>GAGCGTTCTGAACAAATCCAGATGGAGTTCTGAGGTCATTACTGGATCTATCAACAGGAGTCCAAGCGAGCTCGTAAACTTGGTCTGA<br>CAGTTACCAATGCTTAATCAGTGAGGCACCTATCTCAGCGATCTGTCTATTTCGTTCATCCATAGTTGCCTGACTCCCCGTCGTGTAGA<br>TAACTACGATACGGGAGGGCTTACCATCTGGCCCCAGTGCTGCAATGATACCGCGAGACCCACGCTCACCGGCTCCAGATTTATCAG<br>CAATAAACCAGCCAGCCGGAAGGGCCGAGCGCAGAAGTGGTCCTGCAACTTTATCCGCCTCCATCCAGTCTATTAATTGTTGCCGGG<br>AAGCTAGAGTAAGTAGTTCGCCAGTTAATAGTTTGCGCAACGTTGTTGCCATTGCTACAGGCATCGTGGTGTCACGCTCGTCGTTTGG<br>TATGGCTTCATTCAGCTCCGGTTCCCAACGATCAAGGCGAGTTACATGATCCCCCATGTTGTGCAAAAAAGCGGTTAGCTCCTTCGGT<br>CCTCCGATCGTTGTCAGAAGTAAGTTGGCCGCAGTGTTATCACTCATGGTTATGGCAGCACTGCATAATTCTCTTACTGTCATGCCATC<br>CGTAAGATGCTTTTCTGTGACTGGTGAGTACTCAACCAAGTCATTCTGAGAATAGTGTATGCGGCGACCGAGTTGCTCTTGCCCGGCG<br>TCAATACGGGATAATACCGCGCCACATAGCAGAACTTTAAAAGTGCTCATCATTGGAAAACGTTCTTCGGGGCGAAAACTCTCAAGGA<br>TCTTACCGCTGTTGAGATCCAGTTCGATGTAACCCACTCGTGCACCCAACTGATCTTCAGCATCTTTTACTTTCACCAGCGTTTCTGGG<br>TGAGCAAAAACAGGAAGGCAAAATGCCGCAAAAAAGGGAATAAGGGCGACACGGAAATGTTGAATACTCATACTCTTCCTTTTTCAAT<br>ATTATTGAAGCATTTATCAGGGTTATTGTCTCATGAGCGGATACATATTTGAATGTATTTAGAAAAATAAACAAATAGGGGTTCCGCGCA<br>CATTTCCCCGAAAAGTGCCACCTGACGTCTAAGAAACCATTATTATCATGACATTAACCTATAAAAATAGGCGT	
pJUB4X	ATCACGAGGCCCTTTCGTCTTCACCTCGAGGATCCCGGGTACCTGCAGCTAGCGTCGACTGTGGTCTCTACAGGAATTCTTTCTTCTG<br>TCGCCGTCTTGTCAACGGCACCAGTTATATATTTCTTCTGTCGCCGTCTTGTCAACGGCACCAGTTATATATTGGTTTCTTCTGTCGCC<br>GTCTTGTCAACGGCACCAGTTATATATTTCTTCTGTCGCCGTCTTGTCAACGGCACCAGTTATATATTGGGTTCCACCGGATACCTCCG<br>GACTTGACAGCTAGCTCAGTCCTAGGGATTGTGCTAGCGAATTCATTAAAGAGGAGAAAGGTACCATGTCTAAAGGTGAAGAACTGTT<br>CACCGGTGTTGTTCCGATCCTGGTTGAACTGGATGGTGATGTTAACGGCCACAAATTCTCTGTTCGTGGTGAAGGTGAAGGTGATGCA<br>ACCAACGGTAAACTGACCCTGAAATTCATCTGCACTACCGGTAAACTGCCGGTTCCATGGCCGACTCTGGTGACTACCCTGACCTATG<br>GTGTTCAGTGTTTTTCTCGTTACCCGGATCACATGAAGCAGCATGATTTCTTCAAATCTGCAATGCCGGAAGGTTATGTACAGGAGCG<br>CACCATTTCTTTCAAAGACGATGGCACCTACAAAACCCGTGCAGAGGTTAAATTTGAAGGTGATACTCTGGTGAACCGTATTGAACTGA<br>AAGGCATTGATTTCAAAGAGGACGGCAACATCCTGGGCCACAAACTGGAATATAACTTCAACTCCCATAACGTTTACATCACCGCAGA<br>CAAACAGAAGAACGGTATCAAAGCTAACTTCAAAATTCGCCATAACGTTGAAGACGGTAGCGTACAGCTGGCGGACCACTACCAGCA<br>GAACACTCCGATCGGTGATGGTCCGGTTCTGCTGCCGGATAACCACTACCTGTCCACCCAGTCTGTTCTGTCCAAAGACCCGAACGA<br>AAAGCGCGACCACATGGTGCTGCTGGAGTTCGTTACTGCAGCAGGTATCACGCACGGCATGGATGAGCTCTACAAATGATAAGCGGC<br>CGCTCTAGAGGCATCAAATAAAACGAAAGGCTCAGTCGAAAGACTGGGCCTTTCGTTTTATCTGTTGTTTGTCGGTGAACGCTCTCCT<br>GAGTAGGACAAATCCGCCGCCCTAGACCTAGGCGGAGATATTCCGCTTCCTCGCTCACTGACTCGCTCCGCTCGGTCGTTCGGCTGC<br>GGCGAGCGGTACCGGCTTACTGGCGGGGCGGAAATTTCCTGGAAGATGCCAGGAAGAAACTTAACAGGGAAGCGATAAGGCCACGG<br>CAGAGCCGTTTTTCCATAGGCTCCGCCCCCCTGACAAGCATCACGAAATCTGACGCTCAAATCAGTGGTGGCGAAACCCGACAGGAC<br>TTAAAGATACCAGGCGTTTCCCCCTGGTGGCTCCCTCGTGCGCTCTCCTGTTCCTGCCTTTCGGTTTACCGGTGTCACTCCGCTGTTA<br>TGGCCGCGTTTATCTCATTCCACGCCTGACACTCGGTTCCGGGAAGGCAGTTCGCTCCAAGCTGGACTGTATGCACGAACCCCCCGT<br>TCAGTCCGACTGCTGCGCCTGTTCCGGTAACTATCGACTTGAGTCCAAGCCGGAAAGACACGACAAAGCGCCACTGGCAGCAGCCA<br>CTGGTAAGAGGGTGTACAAGAGATTACTGATGCAGAGTTCTTGAAGTCAGAGCCAAACTACGGCTAAACTGGAAGGACGAATTTGGT<br>GGCAGCGGTCTTGTACACCCTGTTACCACGGTTCAGAAGTTCCCCAACTTACTGAACCTTCGAAAAACCACCTCCCCAGGTGGTTTTT<br>TCGTTTTCAGAGCAAGAGATTACGACGCAACGGAAAGGATCTCAAGAGGTTCATCTTACTAGTGCTTGGATTCTCACCAATAAAAAACG<br>CCCGGCGGCAACCGAGCGTTCTGAACAAATCCAGATGGAGTTCTGAGGTCATTACTGGATCTATCAACAGGAGTCCAAGCGAGCTCG<br>TAAACTTGGTCTGACAGTTACCAATGCTTAATCAGTGAGGCACCTATCTCAGCGATCTGTCTATTTCGTTCATCCATAGTTGCCTGACT<br>CCCCGTCGTGTAGATAACTACGATACGGGAGGGCTTACCATCTGGCCCCAGTGCTGCAATGATACCGCGAGACCCACGCTCACCGG<br>CTCCAGATTTATCAGCAATAAACCAGCCAGCCGGAAGGGCCGAGCGCAGAAGTGGTCCTGCAACTTTATCCGCCTCCATCCAGTCTA<br>TTAATTGTTGCCGGGAAGCTAGAGTAAGTAGTTCGCCAGTTAATAGTTTGCGCAACGTTGTTGCCATTGCTACAGGCATCGTGGTGTC<br>ACGCTCGTCGTTTGGTATGGCTTCATTCAGCTCCGGTTCCCAACGATCAAGGCGAGTTACATGATCCCCCATGTTGTGCAAAAAAGCG<br>GTTAGCTCCTTCGGTCCTCCGATCGTTGTCAGAAGTAAGTTGGCCGCAGTGTTATCACTCATGGTTATGGCAGCACTGCATAATTCTC<br>TTACTGTCATGCCATCCGTAAGATGCTTTTCTGTGACTGGTGAGTACTCAACCAAGTCATTCTGAGAATAGTGTATGCGGCGACCGAG<br>TTGCTCTTGCCCGGCGTCAATACGGGATAATACCGCGCCACATAGCAGAACTTTAAAAGTGCTCATCATTGGAAAACGTTCTTCGGGG<br>CGAAAACTCTCAAGGATCTTACCGCTGTTGAGATCCAGTTCGATGTAACCCACTCGTGCACCCAACTGATCTTCAGCATCTTTTACTTT<br>CACCAGCGTTTCTGGGTGAGCAAAAACAGGAAGGCAAAATGCCGCAAAAAAGGGAATAAGGGCGACACGGAAATGTTGAATACTCAT<br>ACTCTTCCTTTTTCAATATTATTGAAGCATTTATCAGGGTTATTGTCTCATGAGCGGATACATATTTGAATGTATTTAGAAAAATAAACAA<br>ATAGGGGTTCCGCGCACATTTCCCCGAAAAGTGCCACCTGACGTCTAAGAAACCATTATTATCATGACATTAACCTATAAAAATAGGCG<br>T	This study

pANAC2X	ATCACGAGGCCCTTTCGTCTTCACCTCGAGGATCCCGGGTACCTGCAGCTAGCGTCGACTGTGGTCTCTACAGGAATTCCGTAGTAA<br>TCAAACCCATTACGCAACTAAGCGTAGTAATCAAACCCATTACGCAACTAAGTCTAGTTCCACCGGATACCTCCGGACTTGACAGCTA<br>GCTCAGTCCTAGGGATTGTGCTAGCGAATTCATTAAAGAGGAGAAAGGTACCATGTCTAAAGGTGAAGAACTGTTCACCGGTGTTGTT<br>CCGATCCTGGTTGAACTGGATGGTGATGTTAACGGCCACAAATTCTCTGTTCGTGGTGAAGGTGAAGGTGATGCAACCAACGGTAAA<br>CTGACCCTGAAATTCATCTGCACTACCGGTAAACTGCCGGTTCCATGGCCGACTCTGGTGACTACCCTGACCTATGGTGTTCAGTGTT<br>TTTCTCGTTACCCGGATCACATGAAGCAGCATGATTTCTTCAAATCTGCAATGCCGGAAGGTTATGTACAGGAGCGCACCATTTCTTTC<br>AAAGACGATGGCACCTACAAAACCCGTGCAGAGGTTAAATTTGAAGGTGATACTCTGGTGAACCGTATTGAACTGAAAGGCATTGATT<br>TCAAAGAGGACGGCAACATCCTGGGCCACAAACTGGAATATAACTTCAACTCCCATAACGTTTACATCACCGCAGACAAACAGAAGAA<br>CGGTATCAAAGCTAACTTCAAAATTCGCCATAACGTTGAAGACGGTAGCGTACAGCTGGCGGACCACTACCAGCAGAACACTCCGAT<br>CGGTGATGGTCCGGTTCTGCTGCCGGATAACCACTACCTGTCCACCCAGTCTGTTCTGTCCAAAGACCCGAACGAAAAGCGCGACCA<br>CATGGTGCTGCTGGAGTTCGTTACTGCAGCAGGTATCACGCACGGCATGGATGAGCTCTACAAATGATAAGCGGCCGCTCTAGAGGC<br>ATCAAATAAAACGAAAGGCTCAGTCGAAAGACTGGGCCTTTCGTTTTATCTGTTGTTTGTCGGTGAACGCTCTCCTGAGTAGGACAAAT<br>CCGCCGCCCTAGACCTAGGCGGAGATATTCCGCTTCCTCGCTCACTGACTCGCTCCGCTCGGTCGTTCGGCTGCGGCGAGCGGTAC<br>CGGCTTACTGGCGGGGCGGAAATTTCCTGGAAGATGCCAGGAAGAAACTTAACAGGGAAGCGATAAGGCCACGGCAGAGCCGTTTT<br>TCCATAGGCTCCGCCCCCCTGACAAGCATCACGAAATCTGACGCTCAAATCAGTGGTGGCGAAACCCGACAGGACTTAAAGATACCA<br>GGCGTTTCCCCCTGGTGGCTCCCTCGTGCGCTCTCCTGTTCCTGCCTTTCGGTTTACCGGTGTCACTCCGCTGTTATGGCCGCGTTT<br>ATCTCATTCCACGCCTGACACTCGGTTCCGGGAAGGCAGTTCGCTCCAAGCTGGACTGTATGCACGAACCCCCCGTTCAGTCCGACT<br>GCTGCGCCTGTTCCGGTAACTATCGACTTGAGTCCAAGCCGGAAAGACACGACAAAGCGCCACTGGCAGCAGCCACTGGTAAGAGG<br>GTGTACAAGAGATTACTGATGCAGAGTTCTTGAAGTCAGAGCCAAACTACGGCTAAACTGGAAGGACGAATTTGGTGGCAGCGGTCTT<br>GTACACCCTGTTACCACGGTTCAGAAGTTCCCCAACTTACTGAACCTTCGAAAAACCACCTCCCCAGGTGGTTTTTTCGTTTTCAGAGC<br>AAGAGATTACGACGCAACGGAAAGGATCTCAAGAGGTTCATCTTACTAGTGCTTGGATTCTCACCAATAAAAAACGCCCGGCGGCAAC<br>CGAGCGTTCTGAACAAATCCAGATGGAGTTCTGAGGTCATTACTGGATCTATCAACAGGAGTCCAAGCGAGCTCGTAAACTTGGTCTG<br>ACAGTTACCAATGCTTAATCAGTGAGGCACCTATCTCAGCGATCTGTCTATTTCGTTCATCCATAGTTGCCTGACTCCCCGTCGTGTAG<br>ATAACTACGATACGGGAGGGCTTACCATCTGGCCCCAGTGCTGCAATGATACCGCGAGACCCACGCTCACCGGCTCCAGATTTATCA<br>GCAATAAACCAGCCAGCCGGAAGGGCCGAGCGCAGAAGTGGTCCTGCAACTTTATCCGCCTCCATCCAGTCTATTAATTGTTGCCGG<br>GAAGCTAGAGTAAGTAGTTCGCCAGTTAATAGTTTGCGCAACGTTGTTGCCATTGCTACAGGCATCGTGGTGTCACGCTCGTCGTTTG<br>GTATGGCTTCATTCAGCTCCGGTTCCCAACGATCAAGGCGAGTTACATGATCCCCCATGTTGTGCAAAAAAGCGGTTAGCTCCTTCGG<br>TCCTCCGATCGTTGTCAGAAGTAAGTTGGCCGCAGTGTTATCACTCATGGTTATGGCAGCACTGCATAATTCTCTTACTGTCATGCCAT<br>CCGTAAGATGCTTTTCTGTGACTGGTGAGTACTCAACCAAGTCATTCTGAGAATAGTGTATGCGGCGACCGAGTTGCTCTTGCCCGGC<br>GTCAATACGGGATAATACCGCGCCACATAGCAGAACTTTAAAAGTGCTCATCATTGGAAAACGTTCTTCGGGGCGAAAACTCTCAAGG<br>ATCTTACCGCTGTTGAGATCCAGTTCGATGTAACCCACTCGTGCACCCAACTGATCTTCAGCATCTTTTACTTTCACCAGCGTTTCTGG<br>GTGAGCAAAAACAGGAAGGCAAAATGCCGCAAAAAAGGGAATAAGGGCGACACGGAAATGTTGAATACTCATACTCTTCCTTTTTCAA<br>TATTATTGAAGCATTTATCAGGGTTATTGTCTCATGAGCGGATACATATTTGAATGTATTTAGAAAAATAAACAAATAGGGGTTCCGCGC<br>ACATTTCCCCGAAAAGTGCCACCTGACGTCTAAGAAACCATTATTATCATGACATTAACCTATAAAAATAGGCGT	This study
pANAC4X	ATCACGAGGCCCTTTCGTCTTCACCTCGAGGATCCCGGGTACCTGCAGCTAGCGTCGACTGTGGTCTCTACAGGAATTCCGTAGTAA<br>TCAAACCCATTACGCAACTAAGCGTAGTAATCAAACCCATTACGCAACTAACGTAGTAATCAAACCCATTACGCAACTAAGCGTAGTAA<br>TCAAACCCATTACGCAACTAAGTCTAGTTCCACCGGATACCTCCGGACTTGACAGCTAGCTCAGTCCTAGGGATTGTGCTAGCGAATT<br>CATTAAAGAGGAGAAAGGTACCATGTCTAAAGGTGAAGAACTGTTCACCGGTGTTGTTCCGATCCTGGTTGAACTGGATGGTGATGTT<br>AACGGCCACAAATTCTCTGTTCGTGGTGAAGGTGAAGGTGATGCAACCAACGGTAAACTGACCCTGAAATTCATCTGCACTACCGGTA<br>AACTGCCGGTTCCATGGCCGACTCTGGTGACTACCCTGACCTATGGTGTTCAGTGTTTTTCTCGTTACCCGGATCACATGAAGCAGCA<br>TGATTTCTTCAAATCTGCAATGCCGGAAGGTTATGTACAGGAGCGCACCATTTCTTTCAAAGACGATGGCACCTACAAAACCCGTGCA<br>GAGGTTAAATTTGAAGGTGATACTCTGGTGAACCGTATTGAACTGAAAGGCATTGATTTCAAAGAGGACGGCAACATCCTGGGCCACA<br>AACTGGAATATAACTTCAACTCCCATAACGTTTACATCACCGCAGACAAACAGAAGAACGGTATCAAAGCTAACTTCAAAATTCGCCAT<br>AACGTTGAAGACGGTAGCGTACAGCTGGCGGACCACTACCAGCAGAACACTCCGATCGGTGATGGTCCGGTTCTGCTGCCGGATAA<br>CCACTACCTGTCCACCCAGTCTGTTCTGTCCAAAGACCCGAACGAAAAGCGCGACCACATGGTGCTGCTGGAGTTCGTTACTGCAGC<br>AGGTATCACGCACGGCATGGATGAGCTCTACAAATGATAAGCGGCCGCTCTAGAGGCATCAAATAAAACGAAAGGCTCAGTCGAAAG<br>ACTGGGCCTTTCGTTTTATCTGTTGTTTGTCGGTGAACGCTCTCCTGAGTAGGACAAATCCGCCGCCCTAGACCTAGGCGGAGATATT<br>CCGCTTCCTCGCTCACTGACTCGCTCCGCTCGGTCGTTCGGCTGCGGCGAGCGGTACCGGCTTACTGGCGGGGCGGAAATTTCCTG<br>GAAGATGCCAGGAAGAAACTTAACAGGGAAGCGATAAGGCCACGGCAGAGCCGTTTTTCCATAGGCTCCGCCCCCCTGACAAGCATC<br>ACGAAATCTGACGCTCAAATCAGTGGTGGCGAAACCCGACAGGACTTAAAGATACCAGGCGTTTCCCCCTGGTGGCTCCCTCGTGCG<br>CTCTCCTGTTCCTGCCTTTCGGTTTACCGGTGTCACTCCGCTGTTATGGCCGCGTTTATCTCATTCCACGCCTGACACTCGGTTCCGG<br>GAAGGCAGTTCGCTCCAAGCTGGACTGTATGCACGAACCCCCCGTTCAGTCCGACTGCTGCGCCTGTTCCGGTAACTATCGACTTGA<br>GTCCAAGCCGGAAAGACACGACAAAGCGCCACTGGCAGCAGCCACTGGTAAGAGGGTGTACAAGAGATTACTGATGCAGAGTTCTTG<br>AAGTCAGAGCCAAACTACGGCTAAACTGGAAGGACGAATTTGGTGGCAGCGGTCTTGTACACCCTGTTACCACGGTTCAGAAGTTCC<br>CCAACTTACTGAACCTTCGAAAAACCACCTCCCCAGGTGGTTTTTTCGTTTTCAGAGCAAGAGATTACGACGCAACGGAAAGGATCTC<br>AAGAGGTTCATCTTACTAGTGCTTGGATTCTCACCAATAAAAAACGCCCGGCGGCAACCGAGCGTTCTGAACAAATCCAGATGGAGTT<br>CTGAGGTCATTACTGGATCTATCAACAGGAGTCCAAGCGAGCTCGTAAACTTGGTCTGACAGTTACCAATGCTTAATCAGTGAGGCAC	This study

	CTATCTCAGCGATCTGTCTATTTCGTTCATCCATAGTTGCCTGACTCCCCGTCGTGTAGATAACTACGATACGGGAGGGCTTACCATCT<br>GGCCCCAGTGCTGCAATGATACCGCGAGACCCACGCTCACCGGCTCCAGATTTATCAGCAATAAACCAGCCAGCCGGAAGGGCCGA<br>GCGCAGAAGTGGTCCTGCAACTTTATCCGCCTCCATCCAGTCTATTAATTGTTGCCGGGAAGCTAGAGTAAGTAGTTCGCCAGTTAAT<br>AGTTTGCGCAACGTTGTTGCCATTGCTACAGGCATCGTGGTGTCACGCTCGTCGTTTGGTATGGCTTCATTCAGCTCCGGTTCCCAAC<br>GATCAAGGCGAGTTACATGATCCCCCATGTTGTGCAAAAAAGCGGTTAGCTCCTTCGGTCCTCCGATCGTTGTCAGAAGTAAGTTGGC<br>CGCAGTGTTATCACTCATGGTTATGGCAGCACTGCATAATTCTCTTACTGTCATGCCATCCGTAAGATGCTTTTCTGTGACTGGTGAGT<br>ACTCAACCAAGTCATTCTGAGAATAGTGTATGCGGCGACCGAGTTGCTCTTGCCCGGCGTCAATACGGGATAATACCGCGCCACATA<br>GCAGAACTTTAAAAGTGCTCATCATTGGAAAACGTTCTTCGGGGCGAAAACTCTCAAGGATCTTACCGCTGTTGAGATCCAGTTCGAT<br>GTAACCCACTCGTGCACCCAACTGATCTTCAGCATCTTTTACTTTCACCAGCGTTTCTGGGTGAGCAAAAACAGGAAGGCAAAATGCC<br>GCAAAAAAGGGAATAAGGGCGACACGGAAATGTTGAATACTCATACTCTTCCTTTTTCAATATTATTGAAGCATTTATCAGGGTTATTGT<br>CTCATGAGCGGATACATATTTGAATGTATTTAGAAAAATAAACAAATAGGGGTTCCGCGCACATTTCCCCGAAAAGTGCCACCTGACGT<br>CTAAGAAACCATTATTATCATGACATTAACCTATAAAAATAGGCGT	
pGRF2X	ATCACGAGGCCCTTTCGTCTTCACCTCGAGGATCCCGGGTACCTGCAGCTAGCGTCGACTGTGGTCTCTACAGGAATTCCCGGTACT<br>GGTCTGACATACCAGAACTTCACCGGTACTGGTCTGACATACCAGAACTTCATCTAGGTTCCACCGGATACCTCCGGACTTGACAGCT<br>AGCTCAGTCCTAGGGATTGTGCTAGCGAATTCATTAAAGAGGAGAAAGGTACCATGTCTAAAGGTGAAGAACTGTTCACCGGTGTTGT<br>TCCGATCCTGGTTGAACTGGATGGTGATGTTAACGGCCACAAATTCTCTGTTCGTGGTGAAGGTGAAGGTGATGCAACCAACGGTAAA<br>CTGACCCTGAAATTCATCTGCACTACCGGTAAACTGCCGGTTCCATGGCCGACTCTGGTGACTACCCTGACCTATGGTGTTCAGTGTT<br>TTTCTCGTTACCCGGATCACATGAAGCAGCATGATTTCTTCAAATCTGCAATGCCGGAAGGTTATGTACAGGAGCGCACCATTTCTTTC<br>AAAGACGATGGCACCTACAAAACCCGTGCAGAGGTTAAATTTGAAGGTGATACTCTGGTGAACCGTATTGAACTGAAAGGCATTGATT<br>TCAAAGAGGACGGCAACATCCTGGGCCACAAACTGGAATATAACTTCAACTCCCATAACGTTTACATCACCGCAGACAAACAGAAGAA<br>CGGTATCAAAGCTAACTTCAAAATTCGCCATAACGTTGAAGACGGTAGCGTACAGCTGGCGGACCACTACCAGCAGAACACTCCGAT<br>CGGTGATGGTCCGGTTCTGCTGCCGGATAACCACTACCTGTCCACCCAGTCTGTTCTGTCCAAAGACCCGAACGAAAAGCGCGACCA<br>CATGGTGCTGCTGGAGTTCGTTACTGCAGCAGGTATCACGCACGGCATGGATGAGCTCTACAAATGATAAGCGGCCGCTCTAGAGGC<br>ATCAAATAAAACGAAAGGCTCAGTCGAAAGACTGGGCCTTTCGTTTTATCTGTTGTTTGTCGGTGAACGCTCTCCTGAGTAGGACAAAT<br>CCGCCGCCCTAGACCTAGGCGGAGATATTCCGCTTCCTCGCTCACTGACTCGCTCCGCTCGGTCGTTCGGCTGCGGCGAGCGGTAC<br>CGGCTTACTGGCGGGGCGGAAATTTCCTGGAAGATGCCAGGAAGAAACTTAACAGGGAAGCGATAAGGCCACGGCAGAGCCGTTTT<br>TCCATAGGCTCCGCCCCCCTGACAAGCATCACGAAATCTGACGCTCAAATCAGTGGTGGCGAAACCCGACAGGACTTAAAGATACCA<br>GGCGTTTCCCCCTGGTGGCTCCCTCGTGCGCTCTCCTGTTCCTGCCTTTCGGTTTACCGGTGTCACTCCGCTGTTATGGCCGCGTTT<br>ATCTCATTCCACGCCTGACACTCGGTTCCGGGAAGGCAGTTCGCTCCAAGCTGGACTGTATGCACGAACCCCCCGTTCAGTCCGACT<br>GCTGCGCCTGTTCCGGTAACTATCGACTTGAGTCCAAGCCGGAAAGACACGACAAAGCGCCACTGGCAGCAGCCACTGGTAAGAGG<br>GTGTACAAGAGATTACTGATGCAGAGTTCTTGAAGTCAGAGCCAAACTACGGCTAAACTGGAAGGACGAATTTGGTGGCAGCGGTCTT<br>GTACACCCTGTTACCACGGTTCAGAAGTTCCCCAACTTACTGAACCTTCGAAAAACCACCTCCCCAGGTGGTTTTTTCGTTTTCAGAGC<br>AAGAGATTACGACGCAACGGAAAGGATCTCAAGAGGTTCATCTTACTAGTGCTTGGATTCTCACCAATAAAAAACGCCCGGCGGCAAC<br>CGAGCGTTCTGAACAAATCCAGATGGAGTTCTGAGGTCATTACTGGATCTATCAACAGGAGTCCAAGCGAGCTCGTAAACTTGGTCTG<br>ACAGTTACCAATGCTTAATCAGTGAGGCACCTATCTCAGCGATCTGTCTATTTCGTTCATCCATAGTTGCCTGACTCCCCGTCGTGTAG<br>ATAACTACGATACGGGAGGGCTTACCATCTGGCCCCAGTGCTGCAATGATACCGCGAGACCCACGCTCACCGGCTCCAGATTTATCA<br>GCAATAAACCAGCCAGCCGGAAGGGCCGAGCGCAGAAGTGGTCCTGCAACTTTATCCGCCTCCATCCAGTCTATTAATTGTTGCCGG<br>GAAGCTAGAGTAAGTAGTTCGCCAGTTAATAGTTTGCGCAACGTTGTTGCCATTGCTACAGGCATCGTGGTGTCACGCTCGTCGTTTG<br>GTATGGCTTCATTCAGCTCCGGTTCCCAACGATCAAGGCGAGTTACATGATCCCCCATGTTGTGCAAAAAAGCGGTTAGCTCCTTCGG<br>TCCTCCGATCGTTGTCAGAAGTAAGTTGGCCGCAGTGTTATCACTCATGGTTATGGCAGCACTGCATAATTCTCTTACTGTCATGCCAT<br>CCGTAAGATGCTTTTCTGTGACTGGTGAGTACTCAACCAAGTCATTCTGAGAATAGTGTATGCGGCGACCGAGTTGCTCTTGCCCGGC<br>GTCAATACGGGATAATACCGCGCCACATAGCAGAACTTTAAAAGTGCTCATCATTGGAAAACGTTCTTCGGGGCGAAAACTCTCAAGG<br>ATCTTACCGCTGTTGAGATCCAGTTCGATGTAACCCACTCGTGCACCCAACTGATCTTCAGCATCTTTTACTTTCACCAGCGTTTCTGG<br>GTGAGCAAAAACAGGAAGGCAAAATGCCGCAAAAAAGGGAATAAGGGCGACACGGAAATGTTGAATACTCATACTCTTCCTTTTTCAA<br>TATTATTGAAGCATTTATCAGGGTTATTGTCTCATGAGCGGATACATATTTGAATGTATTTAGAAAAATAAACAAATAGGGGTTCCGCGC<br>ACATTTCCCCGAAAAGTGCCACCTGACGTCTAAGAAACCATTATTATCATGACATTAACCTATAAAAATAGGCGT	This study
pGRF4X	ATCACGAGGCCCTTTCGTCTTCACCTCGAGGATCCCGGGTACCTGCAGCTAGCGTCGACTGTGGTCTCTACAGGAATTCCCGGTACT<br>GGTCTGACATACCAGAACTTCACCGGTACTGGTCTGACATACCAGAACTTCACCGGTACTGGTCTGACATACCAGAACTTCACCGGTA<br>CTGGTCTGACATACCAGAACTTCATCTAGGTTCCACCGGATACCTCCGGACTTGACAGCTAGCTCAGTCCTAGGGATTGTGCTAGCGA<br>ATTCATTAAAGAGGAGAAAGGTACCATGTCTAAAGGTGAAGAACTGTTCACCGGTGTTGTTCCGATCCTGGTTGAACTGGATGGTGAT<br>GTTAACGGCCACAAATTCTCTGTTCGTGGTGAAGGTGAAGGTGATGCAACCAACGGTAAACTGACCCTGAAATTCATCTGCACTACCG<br>GTAAACTGCCGGTTCCATGGCCGACTCTGGTGACTACCCTGACCTATGGTGTTCAGTGTTTTTCTCGTTACCCGGATCACATGAAGCA<br>GCATGATTTCTTCAAATCTGCAATGCCGGAAGGTTATGTACAGGAGCGCACCATTTCTTTCAAAGACGATGGCACCTACAAAACCCGT<br>GCAGAGGTTAAATTTGAAGGTGATACTCTGGTGAACCGTATTGAACTGAAAGGCATTGATTTCAAAGAGGACGGCAACATCCTGGGCC<br>ACAAACTGGAATATAACTTCAACTCCCATAACGTTTACATCACCGCAGACAAACAGAAGAACGGTATCAAAGCTAACTTCAAAATTCGC<br>CATAACGTTGAAGACGGTAGCGTACAGCTGGCGGACCACTACCAGCAGAACACTCCGATCGGTGATGGTCCGGTTCTGCTGCCGGA<br>TAACCACTACCTGTCCACCCAGTCTGTTCTGTCCAAAGACCCGAACGAAAAGCGCGACCACATGGTGCTGCTGGAGTTCGTTACTGC	This study

	AGCAGGTATCACGCACGGCATGGATGAGCTCTACAAATGATAAGCGGCCGCTCTAGAGGCATCAAATAAAACGAAAGGCTCAGTCGA<br>AAGACTGGGCCTTTCGTTTTATCTGTTGTTTGTCGGTGAACGCTCTCCTGAGTAGGACAAATCCGCCGCCCTAGACCTAGGCGGAGAT<br>ATTCCGCTTCCTCGCTCACTGACTCGCTCCGCTCGGTCGTTCGGCTGCGGCGAGCGGTACCGGCTTACTGGCGGGGCGGAAATTTC<br>CTGGAAGATGCCAGGAAGAAACTTAACAGGGAAGCGATAAGGCCACGGCAGAGCCGTTTTTCCATAGGCTCCGCCCCCCTGACAAG<br>CATCACGAAATCTGACGCTCAAATCAGTGGTGGCGAAACCCGACAGGACTTAAAGATACCAGGCGTTTCCCCCTGGTGGCTCCCTCG<br>TGCGCTCTCCTGTTCCTGCCTTTCGGTTTACCGGTGTCACTCCGCTGTTATGGCCGCGTTTATCTCATTCCACGCCTGACACTCGGTT<br>CCGGGAAGGCAGTTCGCTCCAAGCTGGACTGTATGCACGAACCCCCCGTTCAGTCCGACTGCTGCGCCTGTTCCGGTAACTATCGAC<br>TTGAGTCCAAGCCGGAAAGACACGACAAAGCGCCACTGGCAGCAGCCACTGGTAAGAGGGTGTACAAGAGATTACTGATGCAGAGTT<br>CTTGAAGTCAGAGCCAAACTACGGCTAAACTGGAAGGACGAATTTGGTGGCAGCGGTCTTGTACACCCTGTTACCACGGTTCAGAAG<br>TTCCCCAACTTACTGAACCTTCGAAAAACCACCTCCCCAGGTGGTTTTTTCGTTTTCAGAGCAAGAGATTACGACGCAACGGAAAGGA<br>TCTCAAGAGGTTCATCTTACTAGTGCTTGGATTCTCACCAATAAAAAACGCCCGGCGGCAACCGAGCGTTCTGAACAAATCCAGATGG<br>AGTTCTGAGGTCATTACTGGATCTATCAACAGGAGTCCAAGCGAGCTCGTAAACTTGGTCTGACAGTTACCAATGCTTAATCAGTGAG<br>GCACCTATCTCAGCGATCTGTCTATTTCGTTCATCCATAGTTGCCTGACTCCCCGTCGTGTAGATAACTACGATACGGGAGGGCTTAC<br>CATCTGGCCCCAGTGCTGCAATGATACCGCGAGACCCACGCTCACCGGCTCCAGATTTATCAGCAATAAACCAGCCAGCCGGAAGG<br>GCCGAGCGCAGAAGTGGTCCTGCAACTTTATCCGCCTCCATCCAGTCTATTAATTGTTGCCGGGAAGCTAGAGTAAGTAGTTCGCCA<br>GTTAATAGTTTGCGCAACGTTGTTGCCATTGCTACAGGCATCGTGGTGTCACGCTCGTCGTTTGGTATGGCTTCATTCAGCTCCGGTT<br>CCCAACGATCAAGGCGAGTTACATGATCCCCCATGTTGTGCAAAAAAGCGGTTAGCTCCTTCGGTCCTCCGATCGTTGTCAGAAGTAA<br>GTTGGCCGCAGTGTTATCACTCATGGTTATGGCAGCACTGCATAATTCTCTTACTGTCATGCCATCCGTAAGATGCTTTTCTGTGACTG<br>GTGAGTACTCAACCAAGTCATTCTGAGAATAGTGTATGCGGCGACCGAGTTGCTCTTGCCCGGCGTCAATACGGGATAATACCGCGC<br>CACATAGCAGAACTTTAAAAGTGCTCATCATTGGAAAACGTTCTTCGGGGCGAAAACTCTCAAGGATCTTACCGCTGTTGAGATCCAG<br>TTCGATGTAACCCACTCGTGCACCCAACTGATCTTCAGCATCTTTTACTTTCACCAGCGTTTCTGGGTGAGCAAAAACAGGAAGGCAA<br>AATGCCGCAAAAAAGGGAATAAGGGCGACACGGAAATGTTGAATACTCATACTCTTCCTTTTTCAATATTATTGAAGCATTTATCAGGG<br>TTATTGTCTCATGAGCGGATACATATTTGAATGTATTTAGAAAAATAAACAAATAGGGGTTCCGCGCACATTTCCCCGAAAAGTGCCAC<br>CTGACGTCTAAGAAACCATTATTATCATGACATTAACCTATAAAAATAGGCGT	
pJUB5X	ATCACGAGGCCCTTTCGTCTTCACCTCGAGGATCCCGGGTACCTGCAGCTAGCGTCGACTGTGGTCTCTACAGGAATTCTTTCTTCTG<br>TCGCCGTCTTGTCAACGGCACCAGTTATATATTTCTTCTGTCGCCGTCTTGTCAACGGCACCAGTTATATATTTCTTCTGTCGCCGTCT<br>TGTCAACGGCACCAGTTATATATTGGTTTCTTCTGTCGCCGTCTTGTCAACGGCACCAGTTATATATTTCTTCTGTCGCCGTCTTGTCA<br>ACGGCACCAGTTATATATTGGGTTCCACCGGATACCTCCGGACTTGACAGCTAGCTCAGTCCTAGGGATTGTGCTAGCGAATTCATTA<br>AAGAGGAGAAAGGTACCATGTCTAAAGGTGAAGAACTGTTCACCGGTGTTGTTCCGATCCTGGTTGAACTGGATGGTGATGTTAACGG<br>CCACAAATTCTCTGTTCGTGGTGAAGGTGAAGGTGATGCAACCAACGGTAAACTGACCCTGAAATTCATCTGCACTACCGGTAAACTG<br>CCGGTTCCATGGCCGACTCTGGTGACTACCCTGACCTATGGTGTTCAGTGTTTTTCTCGTTACCCGGATCACATGAAGCAGCATGATT<br>TCTTCAAATCTGCAATGCCGGAAGGTTATGTACAGGAGCGCACCATTTCTTTCAAAGACGATGGCACCTACAAAACCCGTGCAGAGGT<br>TAAATTTGAAGGTGATACTCTGGTGAACCGTATTGAACTGAAAGGCATTGATTTCAAAGAGGACGGCAACATCCTGGGCCACAAACTG<br>GAATATAACTTCAACTCCCATAACGTTTACATCACCGCAGACAAACAGAAGAACGGTATCAAAGCTAACTTCAAAATTCGCCATAACGT<br>TGAAGACGGTAGCGTACAGCTGGCGGACCACTACCAGCAGAACACTCCGATCGGTGATGGTCCGGTTCTGCTGCCGGATAACCACT<br>ACCTGTCCACCCAGTCTGTTCTGTCCAAAGACCCGAACGAAAAGCGCGACCACATGGTGCTGCTGGAGTTCGTTACTGCAGCAGGTA<br>TCACGCACGGCATGGATGAGCTCTACAAATGATAAGCGGCCGCTCTAGAGGCATCAAATAAAACGAAAGGCTCAGTCGAAAGACTGG<br>GCCTTTCGTTTTATCTGTTGTTTGTCGGTGAACGCTCTCCTGAGTAGGACAAATCCGCCGCCCTAGACCTAGGCGGAGATATTCCGCT<br>TCCTCGCTCACTGACTCGCTCCGCTCGGTCGTTCGGCTGCGGCGAGCGGTACCGGCTTACTGGCGGGGCGGAAATTTCCTGGAAGA<br>TGCCAGGAAGAAACTTAACAGGGAAGCGATAAGGCCACGGCAGAGCCGTTTTTCCATAGGCTCCGCCCCCCTGACAAGCATCACGAA<br>ATCTGACGCTCAAATCAGTGGTGGCGAAACCCGACAGGACTTAAAGATACCAGGCGTTTCCCCCTGGTGGCTCCCTCGTGCGCTCTC<br>CTGTTCCTGCCTTTCGGTTTACCGGTGTCACTCCGCTGTTATGGCCGCGTTTATCTCATTCCACGCCTGACACTCGGTTCCGGGAAGG<br>CAGTTCGCTCCAAGCTGGACTGTATGCACGAACCCCCCGTTCAGTCCGACTGCTGCGCCTGTTCCGGTAACTATCGACTTGAGTCCA<br>AGCCGGAAAGACACGACAAAGCGCCACTGGCAGCAGCCACTGGTAAGAGGGTGTACAAGAGATTACTGATGCAGAGTTCTTGAAGTC<br>AGAGCCAAACTACGGCTAAACTGGAAGGACGAATTTGGTGGCAGCGGTCTTGTACACCCTGTTACCACGGTTCAGAAGTTCCCCAAC<br>TTACTGAACCTTCGAAAAACCACCTCCCCAGGTGGTTTTTTCGTTTTCAGAGCAAGAGATTACGACGCAACGGAAAGGATCTCAAGAG<br>GTTCATCTTACTAGTGCTTGGATTCTCACCAATAAAAAACGCCCGGCGGCAACCGAGCGTTCTGAACAAATCCAGATGGAGTTCTGAG<br>GTCATTACTGGATCTATCAACAGGAGTCCAAGCGAGCTCGTAAACTTGGTCTGACAGTTACCAATGCTTAATCAGTGAGGCACCTATC<br>TCAGCGATCTGTCTATTTCGTTCATCCATAGTTGCCTGACTCCCCGTCGTGTAGATAACTACGATACGGGAGGGCTTACCATCTGGCC<br>CCAGTGCTGCAATGATACCGCGAGACCCACGCTCACCGGCTCCAGATTTATCAGCAATAAACCAGCCAGCCGGAAGGGCCGAGCGC<br>AGAAGTGGTCCTGCAACTTTATCCGCCTCCATCCAGTCTATTAATTGTTGCCGGGAAGCTAGAGTAAGTAGTTCGCCAGTTAATAGTTT<br>GCGCAACGTTGTTGCCATTGCTACAGGCATCGTGGTGTCACGCTCGTCGTTTGGTATGGCTTCATTCAGCTCCGGTTCCCAACGATCA<br>AGGCGAGTTACATGATCCCCCATGTTGTGCAAAAAAGCGGTTAGCTCCTTCGGTCCTCCGATCGTTGTCAGAAGTAAGTTGGCCGCA<br>GTGTTATCACTCATGGTTATGGCAGCACTGCATAATTCTCTTACTGTCATGCCATCCGTAAGATGCTTTTCTGTGACTGGTGAGTACTC<br>AACCAAGTCATTCTGAGAATAGTGTATGCGGCGACCGAGTTGCTCTTGCCCGGCGTCAATACGGGATAATACCGCGCCACATAGCAG<br>AACTTTAAAAGTGCTCATCATTGGAAAACGTTCTTCGGGGCGAAAACTCTCAAGGATCTTACCGCTGTTGAGATCCAGTTCGATGTAAC<br>CCACTCGTGCACCCAACTGATCTTCAGCATCTTTTACTTTCACCAGCGTTTCTGGGTGAGCAAAAACAGGAAGGCAAAATGCCGCAAA	This study

	AAAGGGAATAAGGGCGACACGGAAATGTTGAATACTCATACTCTTCCTTTTTCAATATTATTGAAGCATTTATCAGGGTTATTGTCTCAT<br>GAGCGGATACATATTTGAATGTATTTAGAAAAATAAACAAATAGGGGTTCCGCGCACATTTCCCCGAAAAGTGCCACCTGACGTCTAA<br>GAAACCATTATTATCATGACATTAACCTATAAAAATAGGCGT	
pJUB6X	ATCACGAGGCCCTTTCGTCTTCACCTCGAGGATCCCGGGTACCTGCAGCTAGCGTCGACTGTGGTCTCTACAGGAATTCTTTCTTCTG<br>TCGCCGTCTTGTCAACGGCACCAGTTATATATTTCTTCTGTCGCCGTCTTGTCAACGGCACCAGTTATATATTTCTTCTGTCGCCGTCT<br>TGTCAACGGCACCAGTTATATATTTCTTCTGTCGCCGTCTTGTCAACGGCACCAGTTATATATTGGTTTCTTCTGTCGCCGTCTTGTCA<br>ACGGCACCAGTTATATATTTCTTCTGTCGCCGTCTTGTCAACGGCACCAGTTATATATTGGGTTCCACCGGATACCTCCGGACTTGACA<br>GCTAGCTCAGTCCTAGGGATTGTGCTAGCGAATTCATTAAAGAGGAGAAAGGTACCATGTCTAAAGGTGAAGAACTGTTCACCGGTGT<br>TGTTCCGATCCTGGTTGAACTGGATGGTGATGTTAACGGCCACAAATTCTCTGTTCGTGGTGAAGGTGAAGGTGATGCAACCAACGGT<br>AAACTGACCCTGAAATTCATCTGCACTACCGGTAAACTGCCGGTTCCATGGCCGACTCTGGTGACTACCCTGACCTATGGTGTTCAGT<br>GTTTTTCTCGTTACCCGGATCACATGAAGCAGCATGATTTCTTCAAATCTGCAATGCCGGAAGGTTATGTACAGGAGCGCACCATTTCT<br>TTCAAAGACGATGGCACCTACAAAACCCGTGCAGAGGTTAAATTTGAAGGTGATACTCTGGTGAACCGTATTGAACTGAAAGGCATTG<br>ATTTCAAAGAGGACGGCAACATCCTGGGCCACAAACTGGAATATAACTTCAACTCCCATAACGTTTACATCACCGCAGACAAACAGAA<br>GAACGGTATCAAAGCTAACTTCAAAATTCGCCATAACGTTGAAGACGGTAGCGTACAGCTGGCGGACCACTACCAGCAGAACACTCC<br>GATCGGTGATGGTCCGGTTCTGCTGCCGGATAACCACTACCTGTCCACCCAGTCTGTTCTGTCCAAAGACCCGAACGAAAAGCGCGA<br>CCACATGGTGCTGCTGGAGTTCGTTACTGCAGCAGGTATCACGCACGGCATGGATGAGCTCTACAAATGATAAGCGGCCGCTCTAGA<br>GGCATCAAATAAAACGAAAGGCTCAGTCGAAAGACTGGGCCTTTCGTTTTATCTGTTGTTTGTCGGTGAACGCTCTCCTGAGTAGGAC<br>AAATCCGCCGCCCTAGACCTAGGCGGAGATATTCCGCTTCCTCGCTCACTGACTCGCTCCGCTCGGTCGTTCGGCTGCGGCGAGCG<br>GTACCGGCTTACTGGCGGGGCGGAAATTTCCTGGAAGATGCCAGGAAGAAACTTAACAGGGAAGCGATAAGGCCACGGCAGAGCCG<br>TTTTTCCATAGGCTCCGCCCCCCTGACAAGCATCACGAAATCTGACGCTCAAATCAGTGGTGGCGAAACCCGACAGGACTTAAAGATA<br>CCAGGCGTTTCCCCCTGGTGGCTCCCTCGTGCGCTCTCCTGTTCCTGCCTTTCGGTTTACCGGTGTCACTCCGCTGTTATGGCCGCG<br>TTTATCTCATTCCACGCCTGACACTCGGTTCCGGGAAGGCAGTTCGCTCCAAGCTGGACTGTATGCACGAACCCCCCGTTCAGTCCG<br>ACTGCTGCGCCTGTTCCGGTAACTATCGACTTGAGTCCAAGCCGGAAAGACACGACAAAGCGCCACTGGCAGCAGCCACTGGTAAGA<br>GGGTGTACAAGAGATTACTGATGCAGAGTTCTTGAAGTCAGAGCCAAACTACGGCTAAACTGGAAGGACGAATTTGGTGGCAGCGGT<br>CTTGTACACCCTGTTACCACGGTTCAGAAGTTCCCCAACTTACTGAACCTTCGAAAAACCACCTCCCCAGGTGGTTTTTTCGTTTTCAG<br>AGCAAGAGATTACGACGCAACGGAAAGGATCTCAAGAGGTTCATCTTACTAGTGCTTGGATTCTCACCAATAAAAAACGCCCGGCGGC<br>AACCGAGCGTTCTGAACAAATCCAGATGGAGTTCTGAGGTCATTACTGGATCTATCAACAGGAGTCCAAGCGAGCTCGTAAACTTGGT<br>CTGACAGTTACCAATGCTTAATCAGTGAGGCACCTATCTCAGCGATCTGTCTATTTCGTTCATCCATAGTTGCCTGACTCCCCGTCGTG<br>TAGATAACTACGATACGGGAGGGCTTACCATCTGGCCCCAGTGCTGCAATGATACCGCGAGACCCACGCTCACCGGCTCCAGATTTA<br>TCAGCAATAAACCAGCCAGCCGGAAGGGCCGAGCGCAGAAGTGGTCCTGCAACTTTATCCGCCTCCATCCAGTCTATTAATTGTTGC<br>CGGGAAGCTAGAGTAAGTAGTTCGCCAGTTAATAGTTTGCGCAACGTTGTTGCCATTGCTACAGGCATCGTGGTGTCACGCTCGTCGT<br>TTGGTATGGCTTCATTCAGCTCCGGTTCCCAACGATCAAGGCGAGTTACATGATCCCCCATGTTGTGCAAAAAAGCGGTTAGCTCCTT<br>CGGTCCTCCGATCGTTGTCAGAAGTAAGTTGGCCGCAGTGTTATCACTCATGGTTATGGCAGCACTGCATAATTCTCTTACTGTCATG<br>CCATCCGTAAGATGCTTTTCTGTGACTGGTGAGTACTCAACCAAGTCATTCTGAGAATAGTGTATGCGGCGACCGAGTTGCTCTTGCC<br>CGGCGTCAATACGGGATAATACCGCGCCACATAGCAGAACTTTAAAAGTGCTCATCATTGGAAAACGTTCTTCGGGGCGAAAACTCTC<br>AAGGATCTTACCGCTGTTGAGATCCAGTTCGATGTAACCCACTCGTGCACCCAACTGATCTTCAGCATCTTTTACTTTCACCAGCGTTT<br>CTGGGTGAGCAAAAACAGGAAGGCAAAATGCCGCAAAAAAGGGAATAAGGGCGACACGGAAATGTTGAATACTCATACTCTTCCTTTT<br>TCAATATTATTGAAGCATTTATCAGGGTTATTGTCTCATGAGCGGATACATATTTGAATGTATTTAGAAAAATAAACAAATAGGGGTTCC<br>GCGCACATTTCCCCGAAAAGTGCCACCTGACGTCTAAGAAACCATTATTATCATGACATTAACCTATAAAAATAGGCGT	This study
pOSIP-KO_PBAD-JUB1	GAATTCGAGCTCGGTACCCGGGGATCCGCAAAAAAGGGAATAAGGGCGACACGGAAATGTTGAACTTTTCATACTCCCGCCATTCAG<br>AGAAGAAACCAATTGTCCATATTGCATCAGACATTGCCGTCACTGCGTCTTTTACTGGCTCTTCTCGCTAACCAAACCGGTAACCCCG<br>CTTATTAAAAGCATTCTGTAACAAAGCGGGACCAAAGCCATGACAAAAACGCGTAACAAAAGTGTCTATAATCACGGCAGAAAAGTCC<br>ACATTGATTATTTGCACGGCGTCACACTTTGCTATGCCATAGCATTTTTATCCATAAGATTAGCGGATTCTACCTGACGCTTTTTATCGC<br>AACTCTCTACTGTTTCTCCATACCCGTTTTTTTGGGAATTCAAAAGATCTAAAGAGGAGAAAGGATCTGTCGACATGAGTGGCGAAGGT<br>AACTTAGGTAAGGATCATGAAGAAGAAAACGAAGCACCACTTCCTGGGTTCAGGTTTCATCCGACGGATGAAGAGCTTTTAGGATACT<br>ATCTTCGAAGAAAAGTAGAGAACAAAACCATCAAACTCGAACTTATCAAACAGATCGATATCTATAAGTACGATCCTTGGGATCTTCCA<br>AGAGTGAGCAGCGTCGGAGAAAAGGAGTGGTACTTCTTCTGCATGAGAGGTAGGAAATACAGGAATAGCGTTCGACCAAACCGAGTG<br>ACCGGTTCAGGTTTCTGGAAAGCCACTGGTATTGATAAACCGGTTTACTCCAATCTTGACTGTGTTGGTCTCAAGAAATCTCTGGTTTA<br>CTATCTTGGTTCAGCCGGTAAAGGCACCAAAACCGATTGGATGATGCATGAATTCCGCCTCCCCTCCACCACGAAAACCGACTCTCCA<br>GCTCAACAAGCAGAGGTATGGACACTTTGCAGAATCTTCAAACGAGTCACATCTCAAAGAAACCCAACCATCTTACCACCAAACCGAA<br>AACCGGTTATCACTTTAACCGACACTTGTTCTAAGACCAGCAGCTTAGATTCCGACCACACGAGCCACCGTACAGTAGATTCCATGTC<br>CCACGAGCCGCCGCTTCCACAGCCACAGAATCCTTATTGGAACCAACATATAGTTGGTTTTAATCAACCGACATATACTGGTAATGATA<br>ATAACCTCCTGATGAGTTTCTGGAACGGCAACGGTGGAGATTTCATAGGAGACTCAGCAAGTTGGGATGAACTTAGATCTGTTATAGA<br>TGGCAACACTAAACCCGGCGGAGGTGGTTCAATGAGCCACCAAAAAATCATCCAGGACCTTATTGCTTGGATCGATGAGCATATTGAT<br>CAGCCTCTTAATATTGATGTTGTAGCGAAGAAAAGTGGTTACTCGAAGTGGTATTTACAGCGCATGTTTCGTACCGTCACACATCAAAC<br>ATTAGGAGACTATATCCGTCAACGCCGTTTGTTGTTGGCTGCCGTTGAATTGCGGACAACAGAACGTCCGATTTTCGATATTGCCATG<br>GACTTAGGCTATGTGTCGCAGCAAACCTTCTCTCGCGTCTTCGCTCGTCAATTTGACCGGACTCCCAGCGACTACCGTCACCGGCTTT	This study

AACGCAAAAAACCCCGCTTCGGCGGGGTTTTTTCGCACTAGTAGCGGCCGCTGCAGGCATGCCTCGAGATGCATGGCGCCTAACCTA
AACTGACAGGCATCAAATTAAGCAGAAGGCCATCCTGACGGATGGCCTTTTTGCGTTTCGAACAATTGAAAAAACCTCGCGCCTTACC
TGTTGAGTAATAGTCAAAAGCCTCCGGTCGGAGGCTTTTGACTTTCTGCTTACTGAATTTCGGTGGTGCCGTTAATTAACCGGTGGGC
CCTCATGATAATAATGGTTTCTTAGACGTCCGAAGTTCCTATTCTCTAGAAAGTATAGGAACTTCCCTAGGTCAGCCAAACGTCTCTTC
AGGCCACTGACTAGCGATAACTTTCCCCACAACGGAACAACTCTCATTGCATGGGATCATTGGGTACTGTGGGTTTAGTGGTTGTAAA
AACACCTGACCGCTATCCCTGATCAGTTTCTTGAAGGTAAACTCATCACCCCCAAGTCTGGCTATGCAGAAATCACCTGGCTCAACAG
CCTGCTCAGGGTCAACGAGAATTAACATTCCGTCAGGAAAGCTTGGCTTGGAGCCTGTTGGTGCGGTCATGGAATTACCTTCAACCTC
AAGCCAGAATGCAGAATCACTGGCTTTTTTGGTTGTGCTTACCCATCTCTCCGCATCACCTTTGGTAAAGGTTCTAAGCTTAGGTGAGA
ACATCCCTGCCTGAACATGAGAAAAAACAGGGTACTCATACTCACTTCTAAGTGACGGCTGCATACTAACCGCTTCATACATCTCGTAG
ATTTCTCTGGCGATTGAAGGGCTAAATTCTTCAACGCTAACTTTGAGAATTTTTGTAAGCAATGCGGCGTTATAAGCATTTAATGCATTG
ATGCCATTAAATAAAGCACCAACGCCTGACTGCCCCATCCCCATCTTGTCTGCGACAGATTCCTGGGATAAGCCAAGTTCATTTTTCTT
TTTTTCATAAATTGCTTTAAGGCGACGTGCGTCCTCAAGCTGCTCTTGTGTTAATGGTTTCTTTTTTGTGCTCATACGTTAAATCTATCA
CCGCAAGGGATAAATATCTAACACCGTGCGTGTTGACTATTTTACCTCTGGCGGTGATAATGGTTGCATGTACTAAGGAGGTTGTATA
CGCGTTTTGTTTAACTTTAAGAAGGAGATATACATATGACCGTCCGTAAAAATCCGGCTGGCGGTTGGATTTGTGAACTCTACCCAAAC
GGTGCAAAAGGCAAACGTATCAGAAAGAAATTCGCTACTAAAGGCGAGGCTCTGGCGTTTGAGCAGTACACCGTTCAAAACCCGTGG
CAGGAAGAAAAGGAAGACAGGCGCACGTTAAAAGAGCTGGTTGATTCATGGTATAGCGCTCATGGCATTACACTGAAAGATGGTTTGA
AACGCCAGTTAGCCATGCACCATGCTTTTGAGTGTATGGGCGAACCACTCGCACGCGATTTCGATGCGCAGATGTTTTCCCGCTACC
GAGAAAAACGGTTAAAAGGTGAGTATGCCCGTTCAAACAGAGTGAAAGAGGTATCGCCTCGCACGCTTAATCTTGAGCTGGCCTACTT
CCGGGCGGTGTTCAATGAGCTAAACCGCCTCGGAGAATGGAAGGGTGAAAACCCACTAAAAAATATGCGCCCATTCCGCACAGAAGA
AATGGAAATGACTTGGCTAACTCACGACCAAATTTCGCAACTGCTCGGAGAGTGTAACCGACATGACCACCCTGATTTAGAAACCGTG
GTAAGAATCTGTCTCGCAACTGGCGCACGGTGGTCTGAGGCCGAGAGTCTGAGAAAAAGCCAACTCGCGAAATACAAAATCACATAC
ACCAACACGAAAGGTAGAAAAAATCGCACCGTCCCAATCAGCAAAGAGCTCTATGAGTCTCTGCCTGATGATAAAAAGGGCCGGTTGT
TCAGTGATTGTTATGGCGCGTTCCGGTCAGCTTTGGAAAGAACAGGTATCGAACTACCGGCAGGACAACTTACCCACGTTTTACGTCA
CACCTTCGCTAGTCACTTTATGATGAATGGCGGTAATATTTTGGTGTTGCAGCGCGTACTCGGCCATACAGATATAAAAATGACCATGC
GATATGCGCACTTTGCACCCGACCATTTAGAGGATGCAGTTAAACTAAATCCTTTAGTTCACATTACTAACAGCAAATAAGTTATGGAG
GTTGTATGTCAGTTCGGCTAGCCCATGGGTATGGACAGTTTTCCCTTTGATATGTAACGCACGTTGTGTCTCAAAATCTCTGATGTTAC
ATTGCACAAGATAAAAATATATCATCATGAACAATAAAACTGTCTGCTTACATAAACAGTAATACAAGGGGTGTTATGAGCCATATTCAA
CGGGAAACGTCTTGCTCCCGTCCGCGCTTAAACTCCAACATGGACGCTGATTTATATGGGTATAAATGGGCTCGCGATAATGTCGGG
CAATCAGGTGCGACAATCTATCGCTTGTATGGGAAGCCCGATGCGCCAGAGTTGTTTCTGAAACATGGCAAAGGTAGCGTTGCCAAT
GATGTTACAGATGAGATGGTCCGTCTCAACTGGCTGACGGAGTTTATGCCTCTCCCGACCATCAAGCATTTTATCCGTACTCCTGATG
ATGCGTGGTTACTCACCACCGCGATTCCTGGGAAAACAGCCTTCCAGGTATTAGAAGAATATCCTGATTCAGGTGAAAATATTGTTGAT
GCGCTGGCCGTGTTCCTGCGCCGGTTACATTCGATTCCTGTTTGTAATTGTCCTTTTAACAGCGATCGTGTATTTCGTCTTGCTCAGGC
GCAATCACGCATGAATAACGGTTTGGTTGATGCGAGTGATTTTGATGACGAGCGTAATGGCTGGCCTGTTGAACAAGTCTGGAAAGAA
ATGCACAAGCTCTTGCCATTCTCACCGGATTCAGTCGTCACTCATGGTGATTTCTCACTTGATAACCTTATTTTTGACGAGGGGAAATT
AATAGGTTGTATTGATGTTGGACGGGTCGGAATCGCAGACCGTTACCAGGACCTTGCCATTCTTTGGAACTGCCTCGGTGAGTTTTCT
CCTTCATTACAGAAACGGCTTTTTCAAAAATATGGTATTGATAATCCTGATATGAATAAATTGCAGTTTCATTTGATGCTCGATGAGTTTT
TCTAATAATACTAGCAGAAATCATCCTTAGCGAAAGCTAAGGATTTTTTTTATCTGATTACCGCCTTTGAGTGAGCGTCGACCTAGTGC
GGCCGCAAGATCCGGCCACGATGCGTCCGGCGTAGAGGATCTGAAGATCAGCAGTTCAACCTGTTGATAGTACGTACTAAGCTCTCA
TGTTTCACGTACTAAGCTCTCATGTTTAACGTACTAAGCTCTCATGTTTAACGAACTAAACCCTCATGGCTAACGTACTAAGCTCTCATG
GCTAACGTACTAAGCTCTCATGTTTCACGTACTAAGCTCTCATGTTTGAACAATAAAATTAATATAAATCAGCAACTTAAATAGCCTCTA
AGGTTTTAAGTTTTATAAGAAAAAAAAGAATATATAAGGCTTTTAAAGCTTTTAAGGTTTAACGGTTGTGGACAACAAGCCAGGGATGTA
ACGCACTGAGAAGCCCTTAGAGCCTCTCAAAGCAATTTTGAGTGACACAGGAACACTTAACGGCTGACATGGGAATTAGGAAGTTCCT
ATTCCGAAGTTCCTATTCTCTAGAAAGTATAGGAACTTCCATATGCCATGGGCGATGGTTCTGAGTAACAGATAATAGAATGGCGATAA
AGTGGCGGTAGAAATGGCGAATAATAGGTAATTATTGGCAAACAATGGCAATCTATGTCAATGATAAATAACGCATACTATTGATTTTC
GGTTGTTCTTGTAGGAACTCATAATCGCTTGGTCGCTGGTTCAAGTCCAGCAGGGGCCACCAAATTTAAGTAGTAAATACATACAGTTA
GGCCACTCTCTCGAGTGGCCTTTTTGTTGCCTCGTTTTCGAGTGGCGATAGAATGGCGGTGGATTTTTTACCGCCACTTTCCTGAGGA
GTGCATAGCTAGCTTCTTCGTCTGTTTCTACTGGTATTGGCACAAACCTGATTCCAATTTGAGCAAGGCTATGTGCCATCTCGATACTC
GTTCTTAACTCAACAGAAGATGCTTTGTGCATACAGCCCCTCGTTTATTATTTATCTCCTCAGCCAGCCGCTGTGCTTTCAGTGGATTT
CGGATAACAGAAAGGCCGGGAAATACCCAGCCTCGCTTTGTAACGGAGTAGAGACGAAAGTGATTGCGCCTACCCGGATATTATCGT
GAGGATGCGTCATCGCCATTAATTCACTGATCAGTGATAGCTGTCAAACATGAGAATTGATCCGGCTGCCTCGCGCGTTTCGGTGATG
ACGGTGAAAACCTCTGACACATGCAGCTCCCGGAGACGGTCACAGCTTGTCTGTAAGCGGATGCCGGGAGCAGACAAGCCCGTCAG
GGCGCGTCAGCGGGTGTTGGCGGGTGTCGGGGCGCAGCCATGACCCAGTCACGTAGCGATAGCGGAGTGTATGCTGCACATGACA
TTAACCTATAAAAATAGGCGTATCACGAGGCCCTTTCGTCTTCAAGAATTAATTCCCAATTCCCCAGGCATCAAATAAAACGAAAGGCT
CAGTCGAAAGACTGGGCCTTTCGTTTTATCTGTTGTTTGTCGGTGAACGCTCTCCTGAGTAGGACAAATCCGCCGGGAGCGGATTTGA
ACGTTGCGAAGCAACGGCCCGGAGGGTGGCGGGCAGGACGCCCGCCATAAACTGCCAGGAATTAATTCCCCAGGCATCAAATAAAA
CGAAAGGCTCAGTCGAAAGACTGGGCCTTTCGTTTTATCTGTTGTTTGTCGGTGAACGCTCTCCTGAGTAGGACAAATCCGCCGGGA
GCGGATTTGAACGTTGCGAAGCAACGGCCCGGAGGGTGGCGGGCAGGACGCCCGCCATAAACTGCCAGGAATTAATTCCCCAGGCA

	TCAAATAAAACGAAAGGCTCAGTCGAAAGACTGGGCCTTTCGTTTTATCTGTTGTTTGTCGGTGAACGCTCTCCTGAGTAGGACAAATC<br>CGCCGGGAGCGGATTTGAACGTTGCGAAGCAACGGCCCGGAGGGTGGCGGGCAGGACGCCCGCCATAAACTGCCAGGAATTGGGG<br>ATCG	
pOSIP-KO_PBAD-JUB1DBD	AAACCACCGCTACCAGCGGTGGTTTGTTTGCCGGATCAAGAGCTACCAACTCTTTTTCCGAAGGTAACTGGCTTCAGCAGAGCGCAG<br>ATACCAAATACTGTTCTTCTAGTGTAGCCGTAGTTAGGCCACCACTTCAAGAACTCTGTAGCACCGCCTACATACCTCGCTCTGCTAAT<br>CCTGTTACCAGTGGCTGCTGCCAGTGGCGATAAGTCGTGTCTTACCGGGTTGGACTCAAGACGATAGTTACCGGATAAGGCGCAGCG<br>GTCGGGCTGAACGGGGGGTTCGTGCACACAGCCCAGCTTGGAGCGAACGACCTACACCGAACTGAGATACCTACAGCGTGAGCTAT<br>GAGAAAGCGCCACGCTTCCCGAAGGGAGAAAGGCGGACAGGTATCCGGTAAGCGGCAGGGTCGGAACAGGAGAGCGCACGAGGG<br>AGCTTCCAGGGGGAAACGCCTGGTATCTTTATAGTCCTGTCGGGTTTCGCCACCTCTGACTTGAGCGTCGATTTTTGTGATGCTCGTC<br>AGGGGGGCGGAGCCTATGGAAAAACGCCAGCAACGCGGCCTTTTTACGGTTCCTGGCCTTTTGCTGGCCTTTTGCTCATGATATAATT<br>AAATTGAAGCTCTAATTTGTGAGTTTAGTATACATGCATTTACTTATAATACAGTTTTTTAGTTTTGCTGGCCGCATCTTCTCAAATATGC<br>TTCCCAGCCTGCTTTTCTGTAACGTTCACCCTCTACCTTAGCATCCCTTCCCTTTGCAAATAGTCCTCTTCCAACAATAATAATGTCAGA<br>TCCTGTAGAGACCACATCATCCACGGTTCTATACTGTTGACCCAATGCGTCTCCCTTGTCATCTAAACCCACACCGGGTGTCATAATCA<br>ACCAATCGTAACCTTCATCTCTTCCACCCATGTCTCTTTGAGCAATAAAGCCGATAACAAAATCTTTGTCGCTCTTCGCAATGTCAACA<br>GTACCCTTAGTATATTCTCCAGTAGATAGGGAGCCCTTGCATGACAATTCTGCTAACATCAAAAGGCCTCTAGGTTCCTTTGTTACTTC<br>TTCTGCCGCCTGCTTCAAACCGCTAACAATACCTGGGCCCACCACACCGTGTGCATTCGTAATGTCTGCCCATTCTGCTATTCTGTATA<br>CACCCGCAGAGTACTGCAATTTGACTGTATTACCAATGTCAGCAAATTTTCTGTCTTCGAAGAGTAAAAAATTGTACTTGGCGGATAAT<br>GCCTTTAGCGGCTTAACTGTGCCCTCCATGGAAAAATCAGTCAAGATATCCACATGTGTTTTTAGTAAACAAATTTTGGGACCTAATGC<br>TTCAACTAACTCCAGTAATTCCTTGGTGGTACGAACATCCAATGAAGCACACAAGTTTGTTTGCTTTTCGTGCATGATATTAAATAGCTT<br>GGCAGCAACAGGACTAGGATGAGTAGCAGCACGTTCCTTATATGTAGCTTTCGACATGATTTATCTTCGTTTCCTGAGGTTTTTGTTCT<br>GTGCAGTTGGGTTAAGAATACTGGGCAATTTCATGTTTCTTCAACACTACATATGCGTATATATACCAATCTAAGTCTGTGCTCCTTCCT<br>TCGTTCTTCCTTCTGTTCGGAGATTACCGAATCAAAAAAATTTCAAAGAAACCGAAATCAAAAAAAAGAATAAAAAAAAAATGATGAATT<br>GAATTGAAAAGCTAGCTTATCGATGATAAGCTGTCAAAGATGAGAATTAATTCCACGGACTATAGACTATACTAGATACTCCGTCTACT<br>GTACGATACACTTCCGCTCAGGTCCTTGTCCTTTAACGAGGCCTTACCACTCTTTTGTTACTCTATTGATCCAGCTCAGCAAAGGCAGT<br>GTGATCTAAGATTCTATCTTCGCGATGTAGTAAAACTAGCTAGACCGAGAAAGAGACTAGAAATGCAAAAGGCACTTCTACAATGGCT<br>GCCATCATTATTATCCGATGTGACGCTGCAGCTTCTCAATGATATTCGAATACGCTTTGAGGAGATACAGCCTAATATCCGACAAACTG<br>TTTTACAGATTTACGATCGTACTTGTTACCCATCATTGAATTTTGAACATCCGAACCTGGGAGTTTTCCCTGAAACAGATAGTATATTTG<br>AACCTGTATAATAATATATAGTCTAGCGCTTTACGGAAGACAATGTATGTATTTCGGTTCCTGGAGAAACTATTGCATCTATTGCATAGG<br>TAATCTTGCACGTCGCATCCCCGGTTCATTTTCTGCGTTTCCATCTTGCACTTCAATAGCATATCTTTGTTAACGAAGCATCTGTGCTTC<br>ATTTTGTAGAACAAAAATGCAACGCGAGAGCGCTAATTTTTCAAACAAAGAATCTGAGCTGCATTTTTACAGAACAGAAATGCAACGCG<br>AAAGCGCTATTTTACCAACGAAGAATCTGTGCTTCATTTTTGTAAAACAAAAATGCAACGCGACGAGAGCGCTAATTTTTCAAACAAAG<br>AATCTGAGCTGCATTTTTACAGAACAGAAATGCAACGCGAGAGCGCTATTTTACCAACAAAGAATCTATACTTCTTTTTTGTTCTACAAA<br>AATGCATCCCGAGAGCGCTATTTTTCTAACAAAGCATCTTAGATTACTTTTTTTCTCCTTTGTGCGCTCTATAATGCAGTCTCTTGATAA<br>CTTTTTGCACTGTAGGTCCGTTAAGGTTAGAAGAAGGCTACTTTGGTGTCTATTTTCTCTTCCATAAAAAAAGCCTGACTCCACTTCCC<br>GCGTTTACTGATTACTAGCGAAGCTGCGGGTGCATTTTTTCAAGATAAAGGCATCCCCGATTATATTCTATACCGATGTGGATTGCGCA<br>TACTTTGTGAACAGAAAGTGATAGCGTTGATGATTCTTCATTGGTCAGAAAATTATGAACGGTTTCTTCTATTTTGTCTCTATATACTAC<br>GTATAGGAAATGTTTACATTTTCGTATTGTTTTCGATTCACTCTATGAATAGTTCTTACTACAATTTTTTTGTCTAAAGAGTAATACTAGA<br>GATAAACATAAAAAATGTAGAGGTCGAGTTTAGATGCAAGTTCAAGGAGCGAAAGGTGGATGGGTAGGTTATATAGGGATATAGCACA<br>GAGATATATAGCAAAGAGATACTTTTGAGCAATGTTTGTGGAAGCGGTATTCGCAATGGGAAGCTCCACCCCGGTTGATAATCAGAAA<br>AGCCCCAAAAACAGGAAGATTGTATAAGCAAATATTTAAATTGTAATTCTTTCCTGCGTTATCCCCTGATTCTGTGGATAACCGTATTAC<br>CGCCTTTGAGTGAGCTGATACCGCTCGCCGCAGCCGAACGACCGAGCGCAGCGAGTCAGTGAGCGAGGAAGCGGAAGAGGGTCTG<br>ACGCTCAGTGGAACGAAAACTCACGTTAAGGGATTTTGGTCATGAGATTATCAAAAAGGATCTTCACCTAGATCCTTTTAAATTAAAAAT<br>GAAGTTTTAAATCAATCTAAAGTATATATGAGTAAACTTGGTCTGACAGTCAGAAGAACTCGTCAAGAAGGCGATAGAAGGCGATGCG<br>CTGCGAATCGGGAGCGGCGATACCGTAAAGCACGAGGAAGCGGTCAGCCCATTCGCCGCCAAGCTCTTCAGCAATATCACGGGTAG<br>CCAACGCTATGTCCTGATAGCGGTCCGCCACACCCAGCCGGCCACAGTCGATGAATCCAGAAAAGCGGCCATTTTCCACCATGATAT<br>TCGGCAAGCAGGCATCGTCATGGGTCACGACGAGATCCTCGCCGTCGGGCATGCTCGCCTTGAGCCTGGCGAACAGTTCGGCTGGC<br>GCGAGCCCCTGATGCTCTTCGTCCAGATCATCCTGATCGACAAGACCGGCTTCCATCCGAGTACGTGCTCGCTCGATGCGATGTTTC<br>GCTTGGTGGTCGAATGGGCAGGTAGCCGGATCAAGCGTATGCAGCCGCCGCATTGCATCAGCCATGATGGATACTTTCTCGGCAGG<br>AGCAAGGTGAGATGACAGGAGATCCTGCCCCGGCACTTCGCCCAATAGCAGCCAGTCCCTTCCCGCTTCAGTGACAACGTCGAGCA<br>CAGCTGCGCAAGGAACGCCCGTCGTGGCCAGCCACGATAGCCGCGCTGCCTCGTCTTGCAGTTCATTCAGGGCACCGGACAGGTCG<br>GTCTTGACAAAAAGAACCGGGCGCCCCTGCGCTGACAGCCGGAACACGGCGGCATCAGAGCAGCCGATTGTCTGTTGTGCCCAGTC<br>ATAGCCGAATAGCCTCTCCACCCAAGCGGCCGGAGAACCTGCGTGCAATCCATCTTGTTCAATCATACTCTTCCTTTTTCAATATTATT<br>GAAGCATTTATCAGGGTTATTGTCTCATGAGCGGATACATATTTGAATGTATTTAGAAAAATAAACAAATAGGGGTTCCGCGCACATTT<br>CCCCGAAAAGTGCCACCTGCGGACGGATCGCTTGCCTGTAACTTACACGCGCCTCGTCCTGAGAAAGCAACCTGACCTACAGGAAAG<br>AGTTACTCAAGAATAAGAATTTTCGTTTTAAAACCTAAGAGTCACTTTAAAATTTGTATACACTTATTTTTTTTATAACTTATTTAATAATAA<br>AAATCATAAATCATAAGAAATTCGCTTATTTAGAAGTGTCAACAACGTATCTACCAACGATTTGACCCTTTTCCATCTTTTCGTAAATTTC<br>TGGCAAGGTAGACAAGCCGACAACCTTGATTGGAGACTTGACCAAACCTCTGGCGAAGAATTGTTAATATTAAGAGCTCTCACTGCCC	This study

	ACTTTCCAGTCTGGAAACCTGTCTTGCTAGCTGCATGAGTGAATCAGCCAATGCCCGGGGAGAGGCAGTTTGTGTATTGGGTGCCAG<br>GGTGGTTTTTCTCTTCACCAGTGAGACTGGCAACAGCTGATTGCCCTTCACCGCCTGGCCCTGAGAGAGTTGCAGCAAGCGGTCCAC<br>GCTGGTTTGCCCCAGCAGGCGAAAATCCTGTTTGATGGTGGTTAACGGCGGGATATAACATGAGCTGTCTTCGGTATCGTCGTATCC<br>CACTACCGAGATATCTGCACCAACTCTCAGCCCAGACTCAGTAATGGCTCTCATTGCACCCAGTGCCATCTGATCATTGGCAACCAGC<br>ATTGCAGTGGGAACAATGCCCTCATTCAGCATTTGCATGGTTTGTTGAAACCCAGACATGGCACTCCAGTCCCCTTCTCTTTCAGCTAT<br>TGGCTGAATTTGATTCCTAGTGAGATATTTATGCCAGCCGGCCAGTCTCAGCCTTGCTGAGACAGAACTGAGTGGGCCCGCAAGCAG<br>TGCAATTTGCTGGTGTCCCAATGCAACCAGATGCTCCACACCCAGTCTTGTACCATCTTCATGGGAGAAAATAATACTGTTGATGGGT<br>GTCTGGTCAGAGACATCAAGAAAGAGTGCTGGAACATTAGTGCAGGCAGCTTCCACAGCAATGGCATCCTGGTCATCCAGTGGATAG<br>TTAATGATCAGCCCACTGACTCTTTGTGCCAGAAGATTGTGCACAGCAGCTTTACAGGCTTCAACTCCACTTCTTTCTACCATTGACAC<br>CACCACAGAGGCTCCCAGTTGATCAGCTCTAGATTTAATGGCTGCCACAATTTGAGATGGTGCATGCAGGGCCAGACTGGAGGTGGC<br>AACTCCAATCAGCAAGCTCTGTTTGCCTGCCAGTTGTTGTGCCACTCTGTTGGGAATGTAATTCAGCTCTGCCATGGCTGCTTCCACTT<br>TTTCCCTGGTTTTGGCAGAAACATGGCTGGCCTGGTTCACCACTCTGGAAACAGTCTGATAAGAGACACCGGCATACTCTGCGACATC<br>ATACAATGTTACTGGTTTCATTTTAATAACTAGTGCGAAAAAACCCCGCCGAAGCGGGGTTTTTTGCGTTAAAGCCGGTGACGGTAGT<br>CGCTGGGAGTCCGGTCAAATTGACGAGCGAAGACGCGAGAGAAGGTTTGCTGCGACACATAGCCTAAGTCCATGGCAATATCGAAAA<br>TCGGACGTTCTGTTGTCCGCAATTCAACGGCAGCCAACAACAAACGGCGTTGACGGATATAGTCTCCTAATGTTTGATGTGTGACGGT<br>ACGAAACATGCGCTGTAAATACCACTTCGAGTAACCACTTTTCTTCGCTACAACATCAATATTAAGAGGCTGATCAATATGCTCATCGA<br>TCCAAGCAATAAGGTCCTGGATGATTTTTTGGTGGCTCATTGAACCACCTCCGCCGTTTCTTTGAGATGTGACTCGTTTGAAGATTCTG<br>CAAAGTGTCCATACCTCTGCTTGTTGAGCTGGAGAGTCGGTTTTCGTGGTGGAGGGGAGGCGGAATTCATGCATCATCCAATCGGTT<br>TTGGTGCCTTTACCGGCTGAACCAAGATAGTAAACCAGAGATTTCTTGAGACCAACACAGTCAAGATTGGAGTAAACCGGTTTATCAAT<br>ACCAGTGGCTTTCCAGAAACCTGAACCGGTCACTCGGTTTGGTCGAACGCTATTCCTGTATTTCCTACCTCTCATGCAGAAGAAGTAC<br>CACTCCTTTTCTCCGACGCTGCTCACTCTTGGAAGATCCCAAGGATCGTACTTATAGATATCGATCTGTTTGATAAGTTCGAGTTTGAT<br>GGTTTTGTTCTCTACTTTTCTTCGAAGATAGTATCCTAAAAGCTCTTCATCCGTCGGATGAAACCTGAACCCAGGAAGTGGCATGTCGA<br>CAGATCCTTTCTCCTCTTTAGATCTTTTGAATTCCCAAAAAAACGGGTATGGAGAAACAGTAGAGAGTTGCGATAAAAAGCGTCAGGTA<br>GAATCCGCTAATCTTATGGATAAAAATGCTATGGCATAGCAAAGTGTGACGCCGTGCAAATAATCAATGTGGACTTTTCTGCCGTGATT<br>ATAGACACTTTTGTTACGCGTTTTTGTCATGGCTTTGGTCCCGCTTTGTTACAGAATGCTTTTAATAAGCGGGGTTACCGGTTTGGTTA<br>GCGAGAAGAGCCAGTAAAAGACGCAGTGACGGCAATGTCTGATGCAATATGGACAATTGGTTTCTTCTCTGAATGGCGGGAGTATGA<br>AAAGTTCAACATTTCCGTGTCGCCCTTATTCCCTTTTTTGCGGCATTTTGCCTTCCTGTTTTTGCTCACCCAGAAACGCTGGTGAAAGT<br>AAAAGATGCTGAAGATCAGTTGGGTGCACGAGTGGGTTACATCGAACTGGATCTCAACAGCGGTAAGATCCTTGAGAGTTTTCGCCC<br>CGAAGAACGTTTTCCAATGATGAGCACTTTTAAAGTTCTGCTATGTGGCGCGGTATTATCCCGTATTGACGCCGGGCAAGAGCAACTC<br>GGTCGCCGCATACACTATTCTCAGAATGACTTGGTTGAGTACTCACCAGTCACAGAAAAGCATCTTACGGATGGCATGACAGTAAGAG<br>AATTATGCAGTGCTGCCATAACCATGAGTGATAACACTGCGGCCAACTTACTTCTGACAACGATCGGAGGACCGAAGGAGCTAACCG<br>CTTTTTTGCACAACATGGGGGATCATGTAACTCGCCTTGATCGTTGGGAACCGGAGCTGAATGAAGCCATACCAAACGACGAGCGTG<br>ACACCACGATGCCTGTAGCAATGGCAACAACGTTGCGCAAACTATTAACTGGCGAACTACTTACTCTAGCTTCCCGGCAACAATTAAT<br>AGACTGGATGGAGGCGGATAAAGTTGCAGGACCACTTCTGCGCTCGGCCCTTCCGGCTGGCTGGTTTATTGCTGATAAATCTGGAGC<br>CGGTGAGCGTGGGTCTCGCGGTATCATTGCAGCACTGGGGCCAGATGGTAAGCCCTCCCGTATCGTAGTTATCTACACGACGGGGA<br>GTCAGGCAACTATGGATGAACGAAATAGACAGATCGCTGAGATAGGTGCCTCACTGATTAAGCATTGGTAAAATTATTAACGCTTACAA<br>TTTCCTGATGCGGTATTTTCTCCTTACGCATCTGTGCGGTATTTCACACCGCATCAGGTGGCACTTTTCGGGGAAATGTGCGCGGAAC<br>CCCTATTTGTTTATTTTTCTAAATACATTCAAATATGTATCCGCTGGATCTCATGCTGGAGTTCTTCGCCCACCCTTAATTAACCGTAGA<br>AAAGATCAAAGGATCTTCTTGAGATCCTTTTTTTCTGCGCGTAATCTGCTGCTTGCAAACAAAA	
pWRG717	CTAAATTGTAAGCGTTAATATTTTGTTAAAATTCGCGTTAAATTTTTGTTAAATCAGCTCATTTTTTAACCAATAGGCCGAAATCGGCAAA<br>ATCCCTTATAAATCAAAAGAATAGACCGAGATAGGGTTGAGTGTTGTTCCAGTTTGGAACAAGAGTCCACTATTAAAGAACGTGGACTC<br>CAACGTCAAAGGGCGAAAAACCGTCTATCAGGGCGATGGCCCACTACGTGAACCATCACCCTAATCAAGTTTTTTGGGGTCGAGGTG<br>CCGTAAAGCACTAAATCGGAACCCTAAAGGGAGCCCCCGATTTAGAGCTTGACGGGGAAAGCCGGCGAACGTGGCGAGAAAGGAAG<br>GGAAGAAAGCGAAAGGAGCGGGCGCTAGGGCGCTGGCAAGTGTAGCGGTCACGCTGCGCGTAACCACCACACCCGCCGCGCTTAA<br>TGCGCCGCTACAGGGCGCGTCCCATTCGCCATTCAGGCTGCGCAACTGTTGGGAAGGGCGATCGGTGCGGGCCTCTTCGCTATTAC<br>GCCAGCTGGCGAAAGGGGGATGTGCTGCAAGGCGATTAAGTTGGGTAACGCCAGGGTTTTCCCAGTCACGACGTTGTAAAACGACG<br>GCCAGTGAGCGCGCGTAATACGACTCACTATAGGGCGAATTGGGTACCAGTTACGCTAGGGATAACAGGGTAATATAGAGAGCGCTT<br>TTGAAGCTGGGGTGGGCGAAGAACTCCAGCATGAGATCCCCGCGCTGGAGGATCATCCAGCCGGCGTCCCGGAAAACGATTCCGAA<br>GCCCAACCTTTCATAGAAGGCGGCGGTGGAATCGAAATCTCGTGATGGCAGGTTGGGCGTCGCTTGGTCGGTCATTTCGAACCCCAG<br>AGTCCCGCTCAGAAGAACTCGTCAAGAAGGCGATAGAAGGCGATGCGCTGCGAATCGGGAGCGGCGATACCGTAAAGCACGAGGAA<br>GCGGTCAGCCCATTCGCCGCCAAGCTCTTCAGCAATATCACGGGTAGCCAACGCTATGTCCTGATAGCGGTCCGCCACACCCAGCC<br>GGCCACAGTCGATGAATCCAGAAAAGCGGCCATTTTCCACCATGATATTCGGCAAGCAGGCATCGCCATGGGTCACGACGAGATCCT<br>CGCCGTCGGGCATGCGCGCCTTGAGCCTGGCGAACAGTTCGGCTGGCGCGAGCCCCTGATGCTCTTCGTCCAGATCATCCTGATCG<br>ACAAGACCGGCTTCCATCCGAGTACGTGCTCGCTCGATGCGATGTTTCGCTTGGTGGTCGAATGGGCAGGTAGCCGGATCAAGCGTA<br>TGCAGCCGCCGCATTGCATCAGCCATGATGGATACTTTCTCGGCAGGAGCAAGGTGAGATGACAGGAGATCCTGCCCCGGCACTTC<br>GCCCAATAGCAGCCAGTCCCTTCCCGCTTCAGTGACAACGTCGAGCACAGCTGCGCAAGGAACGCCCGTCGTGGCCAGCCACGATA<br>GCCGCGCTGCCTCGTCCTGCAGTTCATTCAGGGCACCGGACAGGTCGGTCTTGACAAAAAGAACCGGGCGCCCCTGCGCTGACAGC	Hoffmann *et al.*, 2017[6]

	CGGAACACGGCGGCATCAGAGCAGCCGATTGTCTGTTGTGCCCAGTCATAGCCGAATAGCCTCTCCACCCAAGCGGCCGGAGAACC<br>TGCGTGCAATCCATCTTGTTCAATCATGCGAAACGATCCTCATCCTGTCTCTTGATCAGATCTTGATCCCCTGCGCCATCAGATCCTTG<br>GCGGCAAGAAAGCCATCCAGTTTACTTTGCAGGGCTTCCCAACCTTACCAGAGGGCGCCCCAGCTGGCAATTCCGGTTCGCTTGCTG<br>TCCATAAAACCGCCCAGTCTAGCTATCGCCATGTAAGCCCACTGCAAGCTACCTGCTTTCTCTTTGCGCTTGCGTTTTCCCTTGTCCAG<br>ATAGCCCAGTCTCGAGGTCGACGGTATCGATAAGCTTGATATCGAATTCCTGCAGCCCGGGGGATCCACTAGTTCTAGAGCGGCCGC<br>CACCGCGGTGGAGCTCCAGCTTTTGTTCCCTTTAGTGAGGGTTAATTGCGCGCTTGGCGTAATCATGGTCATAGCTGTTTCCTGTGTG<br>AAATTGTTATCCGCTCACAATTCCACACAACATACGAGCCGGAAGCATAAAGTGTAAAGCCTGGGGTGCCTAATGAGTGAGCTAACTC<br>ACATTAATTGCGTTGCGCTCACTGCCCGCTTTCCAGTCGGGAAACCTGTCGTGCCAGCTGCATTAATGAATCGGCCAACGCGCGGGG<br>AGAGGCGGTTTGCGTATTGGGCGCTCTTCCGCTTCCTCGCTCACTGACTCGCTGCGCTCGGTCGTTCGGCTGCGGCGAGCGGTATC<br>AGCTCACTCAAAGGCGGTAATACGGTTATCCACAGAATCAGGGGATAACGCAGGAAAGAACATGTGAGCAAAAGGCCAGCAAAAGGC<br>CAGGAACCGTAAAAAGGCCGCGTTGCTGGCGTTTTTCCATAGGCTCCGCCCCCCTGACGAGCATCACAAAAATCGACGCTCAAGTCA<br>GAGGTGGCGAAACCCGACAGGACTATAAAGATACCAGGCGTTTCCCCCTGGAAGCTCCCTCGTGCGCTCTCCTGTTCCGACCCTGCC<br>GCTTACCGGATACCTGTCCGCCTTTCTCCCTTCGGGAAGCGTGGCGCTTTCTCATAGCTCACGCTGTAGGTATCTCAGTTCGGTGTAG<br>GTCGTTCGCTCCAAGCTGGGCTGTGTGCACGAACCCCCCGTTCAGCCCGACCGCTGCGCCTTATCCGGTAACTATCGTCTTGAGTCC<br>AACCCGGTAAGACACGACTTATCGCCACTGGCAGCAGCCACTGGTAACAGGATTAGCAGAGCGAGGTATGTAGGCGGTGCTACAGA<br>GTTCTTGAAGTGGTGGCCTAACTACGGCTACACTAGAAGGACAGTATTTGGTATCTGCGCTCTGCTGAAGCCAGTTACCTTCGGAAAA<br>AGAGTTGGTAGCTCTTGATCCGGCAAACAAACCACCGCTGGTAGCGGTGGTTTTTTTGTTTGCAAGCAGCAGATTACGCGCAGAAAAA<br>AAGGATCTCAAGAAGATCCTTTGATCTTTTCTACGGGGTCTGACGCTCAGTGGAACGAAAACTCACGTTAAGGGATTTTGGTCATGAG<br>ATTATCAAAAAGGATCTTCACCTAGATCCTTTTAAATTAAAAATGAAGTTTTAAATCAATCTAAAGTATATATGAGTAAACTTGGTCTGAC<br>AGTTACCAATGCTTAATCAGTGAGGCACCTATCTCAGCGATCTGTCTATTTCGTTCATCCATAGTTGCCTGACTCCCCGTCGTGTAGAT<br>AACTACGATACGGGAGGGCTTACCATCTGGCCCCAGTGCTGCAATGATACCGCGAGACCCACGCTCACCGGCTCCAGATTTATCAGC<br>AATAAACCAGCCAGCCGGAAGGGCCGAGCGCAGAAGTGGTCCTGCAACTTTATCCGCCTCCATCCAGTCTATTAATTGTTGCCGGGA<br>AGCTAGAGTAAGTAGTTCGCCAGTTAATAGTTTGCGCAACGTTGTTGCCATTGCTACAGGCATCGTGGTGTCACGCTCGTCGTTTGGT<br>ATGGCTTCATTCAGCTCCGGTTCCCAACGATCAAGGCGAGTTACATGATCCCCCATGTTGTGCAAAAAAGCGGTTAGCTCCTTCGGTC<br>CTCCGATCGTTGTCAGAAGTAAGTTGGCCGCAGTGTTATCACTCATGGTTATGGCAGCACTGCATAATTCTCTTACTGTCATGCCATCC<br>GTAAGATGCTTTTCTGTGACTGGTGAGTACTCAACCAAGTCATTCTGAGAATAGTGTATGCGGCGACCGAGTTGCTCTTGCCCGGCGT<br>CAATACGGGATAATACCGCGCCACATAGCAGAACTTTAAAAGTGCTCATCATTGGAAAACGTTCTTCGGGGCGAAAACTCTCAAGGAT<br>CTTACCGCTGTTGAGATCCAGTTCGATGTAACCCACTCGTGCACCCAACTGATCTTCAGCATCTTTTACTTTCACCAGCGTTTCTGGGT<br>GAGCAAAAACAGGAAGGCAAAATGCCGCAAAAAAGGGAATAAGGGCGACACGGAAATGTTGAATACTCATACTCTTCCTTTTTCAATA<br>TTATTGAAGCATTTATCAGGGTTATTGTCTCATGAGCGGATACATATTTGAATGTATTTAGAAAAATAAACAAATAGGGGTTCCGCGCAC<br>ATTTCCCCGAAAAGTGCCAC	
pK2151200	TACTAGAGGAGGTACTAGATGACGGTCTGCGCAAAAAAACACGTTCATCTCACTCGCGATGCTGCGGAGCAGTTACTGGCTGATATTG<br>ATCGACGCCTTGATCAGTTATTGCCCGTGGAGGGAGAACGGGATGTTGTGGGTGCCGCGATGCGTGAAGGTGCGCTGGCACCGGGA<br>AAACGTATTCGCCCCATGTTGCTGTTGCTGACCGCCCGCGATCTGGGTTGCGCTGTCAGCCATGACGGATTACTGGATTTGGCCTGT<br>GCGGTGGAAATGGTCCACGCGGCTTCGCTGATCCTTGACGATATGCCCTGCATGGACGATGCGAAGCTGCGGCGCGGACGCCCTAC<br>CATTCATTCTCATTACGGAGAGCATGTGGCAATACTGGCGGCGGTTGCCTTGCTGAGTAAAGCCTTTGGCGTAATTGCCGATGCAGAT<br>GGCCTCACGCCGCTGGCAAAAAATCGGGCGGTTTCTGAACTGTCAAACGCCATCGGCATGCAAGGATTGGTTCAGGGTCAGTTCAAG<br>GATCTGTCTGAAGGGGATAAGCCGCGCAGCGCTGAAGCTATTTTGATGACGAATCACTTTAAAACCAGCACGCTGTTTTGTGCCTCCA<br>TGCAGATGGCCTCGATTGTTGCGAATGCCTCCAGCGAAGCGCGTGATTGCCTGCATCGTTTTTCACTTGATCTTGGTCAGGCATTTCA<br>ACTGCTGGACGATTTGACCGATGGCATGACCGACACCGGTAAGGATAGCAATCAGGACGCCGGTAAATCGACGCTGGTCAATCTGTT<br>AGGCCCGAGGGCGGTTGAAGAACGTCTGAGACAACATCTTCAGCTTGCCAGTGAGCATCTCTCTGCGGCCTGCCAACACGGGCACG<br>CCACTCAACATTTTATTCAGGCCTGGTTTGACAAAAAACTCGCTGCCGTCAGTTAATAATACTAGAGCTCAAGGAGGTACTAGATGAAT<br>AATCCGTCGTTACTCAATCATGCGGTCGAAACGATGGCAGTTGGCTCGAAAAGTTTTGCGACAGCCTCAAAGTTATTTGATGCAAAAA<br>CCCGGCGCAGCGTACTGATGCTCTACGCCTGGTGCCGCCATTGTGACGATGTTATTGACGATCAGACGCTGGGCTTTCAGGCCCGG<br>CAGCCTGCCTTACAAACGCCCGAACAACGTCTGATGCAACTTGAGATGAAAACGCGCCAGGCCTATGCAGGATCGCAGATGCACGAA<br>CCGGCGTTTGCGGCTTTTCAGGAAGTGGCTATGGCTCATGATATCGCCCCGGCTTACGCGTTTGATCATCTGGAAGGCTTCGCCATG<br>GATGTACGCGAAGCGCAATACAGCCAACTGGATGATACGCTGCGCTATTGCTATCACGTTGCAGGCGTTGTCGGCTTGATGATGGCG<br>CAAATCATGGGCGTGCGGGATAACGCCACGCTGGACCGCGCCTGTGACCTTGGGCTGGCATTTCAGTTGACCAATATTGCTCGCGAT<br>ATTGTGGACGATGCGCATGCGGGCCGCTGTTATCTGCCGGCAAGCTGGCTGGAGCATGAAGGTCTGAACAAAGAGAATTATGCGGC<br>ACCTGAAAACCGTCAGGCGCTGAGCCGTATCGCCCGTCGTTTGGTGCAGGAAGCAGAACCTTACTATTTGTCTGCCACAGCCGGCCT<br>GGCAGGGTTGCCCCTGCGTTCCGCCTGGGCAATCGCTACGGCGAAGCAGGTTTACCGGAAAATAGGTGTCAAAGTTGAACAGGCCG<br>GTCAGCAAGCCTGGGATCAGCGGCAGTCAACGACCACGCCCGAAAAATTAACGCTGCTGCTGGCCGCCTCTGGTCAGGCCCTTACT<br>TCCCGGATGCGGGCTCATCCTCCCCGCCCTGCGCATCTCTGGCAGCGCCCGCTCTAATAATACTAGAGCTCAAGGAGGTACTAGATG<br>AAACCAACTACGGTAATTGGTGCAGGCTTCGGTGGCCTGGCACTGGCAATTCGTCTACAAGCTGCGGGGATCCCCGTCTTACTGCTT<br>GAACAACGTGATAAACCCGGCGGTCGGGCTTATGTCTACGAGGATCAGGGGTTTACCTTTGATGCAGGCCCGACGGTTATCACCGAT<br>CCCAGTGCCATTGAAGAACTGTTTGCACTGGCAGGAAAACAGTTAAAAGAGTATGTCGAACTGCTGCCGGTTACGCCGTTTTACCGCC<br>TGTGTTGGGAGTCAGGGAAGGTCTTTAATTACGATAACGATCAAACCCGGCTCGAAGCGCAGATTCAGCAGTTTAATCCCCGCGATGT	Gifted by Sean Colloms

	CGAAGGTTATCGTCAGTTTCTGGACTATTCACGCGCGGTGTTTAAAGAAGGCTATCTAAAGCTCGGTACTGTCCCTTTTTTATCGTTCA<br>GAGACATGCTTCGCGCCGCACCTCAACTGGCGAAACTGCAAGCATGGAGAAGCGTTTACAGTAAGGTTGCCAGTTACATCGAAGATG<br>AACATCTGCGCCAGGCGTTTTCTTTCCACTCGCTGTTGGTGGGCGGCAATCCCTTCGCCACCTCATCCATTTATACGTTGATACACGC<br>GCTGGAGCGTGAGTGGGGCGTCTGGTTTCCGCGTGGCGGCACCGGCGCATTAGTTCAGGGGATGATAAAGCTGTTTCAGGATCTGG<br>GTGGCGAAGTCGTGTTAAACGCCAGAGTCAGCCATATGGAAACGACAGGAAACAAGATTGAAGCCGTGCATTTAGAGGACGGTCGCA<br>GGTTCCTGACGCAAGCCGTCGCGTCAAATGCAGATGTGGTTCATACCTATCGCGACCTGTTAAGCCAGCACCCTGCCGCGGTTAAGC<br>AGTCCAACAAACTGCAAACTAAGCGCATGAGTAACTCTCTGTTTGTGCTCTATTTTGGTTTGAATCACCATCATGATCAGCTCGCGCAT<br>CACACGGTTTGTTTCGGCCCGCGTTACCGCGAGCTGATTGACGAAATTTTTAATCATGATGGCCTCGCAGAGGACTTCTCACTTTATC<br>TGCACGCGCCCTGTGTCACGGATTCGTCACTGGCGCCTGAAGGTTGCGGCAGTTACTATGTGTTGGCGCCGGTGCCGCATTTAGGC<br>ACCGCGAACCTCGACTGGACGGTTGAGGGGCCAAAACTACGCGACCGTATTTTTGCGTACCTTGAGCAGCATTACATGCCTGGCTTA<br>CGGAGTCAGCTGGTCACGCACCGGATGTTTACGCCGTTTGATTTTCGCGACCAGCTTAATGCCTATCATGGCTCAGCCTTTTCTGTGG<br>AGCCCGTTCTTACCCAGAGCGCCTGGTTTCGGCCGCATAACCGCGATAAAACCATTACTAATCTCTACCTGGTCGGCGCAGGCACGC<br>ATCCCGGCGCAGGCATTCCTGGCGTCATCGGCTCGGCAAAAGCGACAGCAGGTTTGATGCTGGAGGATCTGATATAATAATACTAGA<br>GGAGGTACTAGATGCAACCGCATTATGATCTGATTCTCGTGGGGGCTGGACTCGCGAATGGCCTTATCGCCCTGCGTCTTCAGCAGC<br>AGCAACCTGATATGCGTATTTTGCTTATCGACGCCGCACCCCAGGCGGGCGGGAATCATACGTGGTCATTTCACCACGATGATTTGAC<br>TGAGAGCCAACATCGTTGGATAGCTCCGCTGGTGGTTCATCACTGGCCCGACTATCAGGTACGCTTTCCCACACGCCGTCGTAAGCT<br>GAACAGCGGCTACTTTTGTATTACTTCTCAGCGTTTCGCTGAGGTTTTACAGCGACAGTTTGGCCCGCACTTGTGGATGGATACCGCG<br>GTCGCAGAGGTTAATGCGGAATCTGTTCGGTTGAAAAAGGGTCAGGTTATCGGTGCCCGCGCGGTGATTGACGGGCGGGGTTATGC<br>GGCAAATTCAGCACTGAGCGTGGGCTTCCAGGCGTTTATTGGCCAGGAATGGCGATTGAGCCACCCGCATGGTTTATCGTCTCCCAT<br>TATCATGGATGCCACGGTCGATCAGCAAAATGGTTATCGCTTCGTGTACAGCCTGCCGCTCTCGCCGACCAGATTGTTAATTGAAGAC<br>ACGCACTATATTGATAATGCGACATTAGATCCTGAATGCGCGCGGCAAAATATTTGCGACTATGCCGCGCAACAGGGTTGGCAGCTTC<br>AGACACTGCTGCGAGAAGAACAGGGCGCCTTACCCATTACTCTGTCGGGCAATGCCGACGCATTCTGGCAGCAGCGCCCCCTGGCC<br>TGTAGTGGATTACGTGCCGGTCTGTTCCATCCTACCACCGGCTATTCACTGCCGCTGGCGGTTGCCGTGGCCGACCGCCTGAGTGCA<br>CTTGATGTCTTTACGTCGGCCTCAATTCACCATGCCATTACGCATTTTGCCCGCGAGCGCTGGCAGCAGCAGGGCTTTTTCCGCATGC<br>TGAATCGCATGCTGTTTTTAGCCGGACCCGCCGATTCACGCTGGCGGGTTATGCAGCGTTTTTATGGTTTACCTGAAGATTTAATTGC<br>CCGTTTTTATGCGGGAAAACTCACGCTGACCGATCGGCTACGTATTCTGAGCGGCAAGCCGCCTGTTCCGGTATTAGCAGCATTGCA<br>AGCCATTATGACGACTCATCGTTAATAANNNNACTAGTAGCGGCCGCTGCAGTCCGGCAAAAAAGGGCAAGGTGTCACCACCCTGCC<br>CTTTTTCTTTAAAACCGAAAAGATTACTTCGCGTTATGCAGGCTTCCTCGCTCACTGACTCGCTGCGCTCGGTCGTTCGGCTGCGGCG<br>AGCGGTATCAGCTCACTCAAAGGCGGTAATACGGTTATCCACAGAATCAGGGGATAACGCAGGAAAGAACATGTGAGCAAAAGGCCA<br>GCAAAAGGCCAGGAACCGTAAAAAGGCCGCGTTGCTGGCGTTTTTCCACAGGCTCCGCCCCCCTGACGAGCATCACAAAAATCGAC<br>GCTCAAGTCAGAGGTGGCGAAACCCGACAGGACTATAAAGATACCAGGCGTTTCCCCCTGGAAGCTCCCTCGTGCGCTCTCCTGTTC<br>CGACCCTGCCGCTTACCGGATACCTGTCCGCCTTTCTCCCTTCGGGAAGCGTGGCGCTTTCTCATAGCTCACGCTGTAGGTATCTCA<br>GTTCGGTGTAGGTCGTTCGCTCCAAGCTGGGCTGTGTGCACGAACCCCCCGTTCAGCCCGACCGCTGCGCCTTATCCGGTAACTATC<br>GTCTTGAGTCCAACCCGGTAAGACACGACTTATCGCCACTGGCAGCAGCCACTGGTAACAGGATTAGCAGAGCGAGGTATGTAGGCG<br>GTGCTACAGAGTTCTTGAAGTGGTGGCCTAACTACGGCTACACTAGAAGAACAGTATTTGGTATCTGCGCTCTGCTGAAGCCAGTTAC<br>CTTCGGAAAAAGAGTTGGTAGCTCTTGATCCGGCAAACAAACCACCGCTGGTAGCGGTGGTTTTTTTGTTTGCAAGCAGCAGATTACG<br>CGCAGAAAAAAAGGATCTCAAGAAGATCCTTTGATCTTTTCTACGGGGTCTGACGCTCAGTGGAACGAAAACTCACGTTAAGGGATTT<br>TGGTCATGAGATTATCAAAAAGGATCTTCACCTAGATCCTTTTAAATTAAAAATGAAGTTTTAAATCAATCTAAAGTATATATGAGTAAAC<br>TTGGTCTGACAGCTCGAGGCTTGGATTCTCACCAATAAAAAACGCCCGGCGGCAACCGAGCGTTCTGAACAAATCCAGATGGAGTTC<br>TGAGGTCATTACTGGATCTATCAACAGGAGTCCAAGCGAGCTCGATATCAAATTACGCCCCGCCCTGCCACTCATCGCAGTACTGTTG<br>TAATTCATTAAGCATTCTGCCGACATGGAAGCCATCACAAACGGCATGATGAACCTGAATCGCCAGCGGCATCAGCACCTTGTCGCCT<br>TGCGTATAATATTTGCCCATGGTGAAAACGGGGGCGAAGAAGTTGTCCATATTGGCCACGTTTAAATCAAAACTGGTGAAACTCACCC<br>AGGGATTGGCTGAGACGAAAAACATATTCTCAATAAACCCTTTAGGGAAATAGGCCAGGTTTTCACCGTAACACGCCACATCTTGCGA<br>ATATATGTGTAGAAACTGCCGGAAATCGTCGTGGTATTCACTCCAGAGCGATGAAAACGTTTCAGTTTGCTCATGGAAAACGGTGTAA<br>CAAGGGTGAACACTATCCCATATCACCAGCTCACCGTCTTTCATTGCCATACGAAATTCCGGATGAGCATTCATCAGGCGGGCAAGAA<br>TGTGAATAAAGGCCGGATAAAACTTGTGCTTATTTTTCTTTACGGTCTTTAAAAAGGCCGTAATATCCAGCTGAACGGTCTGGTTATAG<br>GTACATTGAGCAACTGACTGAAATGCCTCAAAATGTTCTTTACGATGCCATTGGGATATATCAACGGTGGTATATCCAGTGATTTTTTTC<br>TCCATTTTAGCTTCCTTAGCTCCTGAAAATCTCGATAACTCAAAAAATACGCCCGGTAGTGATCTTATTTCATTATGGTGAAAGTTGGAA<br>CCTCTTACGTGCCCGATCAACTCGAGTGCCACCTGACGTCTAAGAAACCATTATTATCATGACATTAACCTATAAAAATAGGCGTATCA<br>CGAGGCAGAATTTCAGATAAAAAAAATCCTTAGCTTTCGCTAAGGATGATTTCTGGAATTCGCGGCCGCTTCTAGAG	
pK2151201	TTTACGGCTAGCTCAGTCCTAGGTATAGTGCTAGCTACTAGAGGAGGTACTAGATGACGGTCTGCGCAAAAAAACACGTTCATCTCAC<br>TCGCGATGCTGCGGAGCAGTTACTGGCTGATATTGATCGACGCCTTGATCAGTTATTGCCCGTGGAGGGAGAACGGGATGTTGTGGG<br>TGCCGCGATGCGTGAAGGTGCGCTGGCACCGGGAAAACGTATTCGCCCCATGTTGCTGTTGCTGACCGCCCGCGATCTGGGTTGCG<br>CTGTCAGCCATGACGGATTACTGGATTTGGCCTGTGCGGTGGAAATGGTCCACGCGGCTTCGCTGATCCTTGACGATATGCCCTGCA<br>TGGACGATGCGAAGCTGCGGCGCGGACGCCCTACCATTCATTCTCATTACGGAGAGCATGTGGCAATACTGGCGGCGGTTGCCTTG<br>CTGAGTAAAGCCTTTGGCGTAATTGCCGATGCAGATGGCCTCACGCCGCTGGCAAAAAATCGGGCGGTTTCTGAACTGTCAAACGCC<br>ATCGGCATGCAAGGATTGGTTCAGGGTCAGTTCAAGGATCTGTCTGAAGGGGATAAGCCGCGCAGCGCTGAAGCTATTTTGATGACG	Gifted by Sean Colloms

AATCACTTTAAAACCAGCACGCTGTTTTGTGCCTCCATGCAGATGGCCTCGATTGTTGCGAATGCCTCCAGCGAAGCGCGTGATTGCC
TGCATCGTTTTTCACTTGATCTTGGTCAGGCATTTCAACTGCTGGACGATTTGACCGATGGCATGACCGACACCGGTAAGGATAGCAA
TCAGGACGCCGGTAAATCGACGCTGGTCAATCTGTTAGGCCCGAGGGCGGTTGAAGAACGTCTGAGACAACATCTTCAGCTTGCCAG
TGAGCATCTCTCTGCGGCCTGCCAACACGGGCACGCCACTCAACATTTTATTCAGGCCTGGTTTGACAAAAAACTCGCTGCCGTCAGT
TAATAATACTAGAGCTCAAGGAGGTACTAGATGAATAATCCGTCGTTACTCAATCATGCGGTCGAAACGATGGCAGTTGGCTCGAAAA
GTTTTGCGACAGCCTCAAAGTTATTTGATGCAAAAACCCGGCGCAGCGTACTGATGCTCTACGCCTGGTGCCGCCATTGTGACGATGT
TATTGACGATCAGACGCTGGGCTTTCAGGCCCGGCAGCCTGCCTTACAAACGCCCGAACAACGTCTGATGCAACTTGAGATGAAAAC
GCGCCAGGCCTATGCAGGATCGCAGATGCACGAACCGGCGTTTGCGGCTTTTCAGGAAGTGGCTATGGCTCATGATATCGCCCCGG
CTTACGCGTTTGATCATCTGGAAGGCTTCGCCATGGATGTACGCGAAGCGCAATACAGCCAACTGGATGATACGCTGCGCTATTGCTA
TCACGTTGCAGGCGTTGTCGGCTTGATGATGGCGCAAATCATGGGCGTGCGGGATAACGCCACGCTGGACCGCGCCTGTGACCTTG
GGCTGGCATTTCAGTTGACCAATATTGCTCGCGATATTGTGGACGATGCGCATGCGGGCCGCTGTTATCTGCCGGCAAGCTGGCTGG
AGCATGAAGGTCTGAACAAAGAGAATTATGCGGCACCTGAAAACCGTCAGGCGCTGAGCCGTATCGCCCGTCGTTTGGTGCAGGAAG
CAGAACCTTACTATTTGTCTGCCACAGCCGGCCTGGCAGGGTTGCCCCTGCGTTCCGCCTGGGCAATCGCTACGGCGAAGCAGGTTT
ACCGGAAAATAGGTGTCAAAGTTGAACAGGCCGGTCAGCAAGCCTGGGATCAGCGGCAGTCAACGACCACGCCCGAAAAATTAACG
CTGCTGCTGGCCGCCTCTGGTCAGGCCCTTACTTCCCGGATGCGGGCTCATCCTCCCCGCCCTGCGCATCTCTGGCAGCGCCCGCT
CTAATAATACTAGAGCTCAAGGAGGTACTAGATGAAACCAACTACGGTAATTGGTGCAGGCTTCGGTGGCCTGGCACTGGCAATTCGT
CTACAAGCTGCGGGGATCCCCGTCTTACTGCTTGAACAACGTGATAAACCCGGCGGTCGGGCTTATGTCTACGAGGATCAGGGGTTT
ACCTTTGATGCAGGCCCGACGGTTATCACCGATCCCAGTGCCATTGAAGAACTGTTTGCACTGGCAGGAAAACAGTTAAAAGAGTATG
TCGAACTGCTGCCGGTTACGCCGTTTTACCGCCTGTGTTGGGAGTCAGGGAAGGTCTTTAATTACGATAACGATCAAACCCGGCTCG
AAGCGCAGATTCAGCAGTTTAATCCCCGCGATGTCGAAGGTTATCGTCAGTTTCTGGACTATTCACGCGCGGTGTTTAAAGAAGGCTA
TCTAAAGCTCGGTACTGTCCCTTTTTTATCGTTCAGAGACATGCTTCGCGCCGCACCTCAACTGGCGAAACTGCAAGCATGGAGAAGC
GTTTACAGTAAGGTTGCCAGTTACATCGAAGATGAACATCTGCGCCAGGCGTTTTCTTTCCACTCGCTGTTGGTGGGCGGCAATCCCT
TCGCCACCTCATCCATTTATACGTTGATACACGCGCTGGAGCGTGAGTGGGGCGTCTGGTTTCCGCGTGGCGGCACCGGCGCATTA
GTTCAGGGGATGATAAAGCTGTTTCAGGATCTGGGTGGCGAAGTCGTGTTAAACGCCAGAGTCAGCCATATGGAAACGACAGGAAAC
AAGATTGAAGCCGTGCATTTAGAGGACGGTCGCAGGTTCCTGACGCAAGCCGTCGCGTCAAATGCAGATGTGGTTCATACCTATCGC
GACCTGTTAAGCCAGCACCCTGCCGCGGTTAAGCAGTCCAACAAACTGCAAACTAAGCGCATGAGTAACTCTCTGTTTGTGCTCTATT
TTGGTTTGAATCACCATCATGATCAGCTCGCGCATCACACGGTTTGTTTCGGCCCGCGTTACCGCGAGCTGATTGACGAAATTTTTAAT
CATGATGGCCTCGCAGAGGACTTCTCACTTTATCTGCACGCGCCCTGTGTCACGGATTCGTCACTGGCGCCTGAAGGTTGCGGCAGT
TACTATGTGTTGGCGCCGGTGCCGCATTTAGGCACCGCGAACCTCGACTGGACGGTTGAGGGGCCAAAACTACGCGACCGTATTTTT
GCGTACCTTGAGCAGCATTACATGCCTGGCTTACGGAGTCAGCTGGTCACGCACCGGATGTTTACGCCGTTTGATTTTCGCGACCAG
CTTAATGCCTATCATGGCTCAGCCTTTTCTGTGGAGCCCGTTCTTACCCAGAGCGCCTGGTTTCGGCCGCATAACCGCGATAAAACCA
TTACTAATCTCTACCTGGTCGGCGCAGGCACGCATCCCGGCGCAGGCATTCCTGGCGTCATCGGCTCGGCAAAAGCGACAGCAGGT
TTGATGCTGGAGGATCTGATATAATAATACTAGAGGAGGTACTAGATGCAACCGCATTATGATCTGATTCTCGTGGGGGCTGGACTCG
CGAATGGCCTTATCGCCCTGCGTCTTCAGCAGCAGCAACCTGATATGCGTATTTTGCTTATCGACGCCGCACCCCAGGCGGGCGGGA
ATCATACGTGGTCATTTCACCACGATGATTTGACTGAGAGCCAACATCGTTGGATAGCTCCGCTGGTGGTTCATCACTGGCCCGACTA
TCAGGTACGCTTTCCCACACGCCGTCGTAAGCTGAACAGCGGCTACTTTTGTATTACTTCTCAGCGTTTCGCTGAGGTTTTACAGCGA
CAGTTTGGCCCGCACTTGTGGATGGATACCGCGGTCGCAGAGGTTAATGCGGAATCTGTTCGGTTGAAAAAGGGTCAGGTTATCGGT
GCCCGCGCGGTGATTGACGGGCGGGGTTATGCGGCAAATTCAGCACTGAGCGTGGGCTTCCAGGCGTTTATTGGCCAGGAATGGCG
ATTGAGCCACCCGCATGGTTTATCGTCTCCCATTATCATGGATGCCACGGTCGATCAGCAAAATGGTTATCGCTTCGTGTACAGCCTG
CCGCTCTCGCCGACCAGATTGTTAATTGAAGACACGCACTATATTGATAATGCGACATTAGATCCTGAATGCGCGCGGCAAAATATTT
GCGACTATGCCGCGCAACAGGGTTGGCAGCTTCAGACACTGCTGCGAGAAGAACAGGGCGCCTTACCCATTACTCTGTCGGGCAAT
GCCGACGCATTCTGGCAGCAGCGCCCCCTGGCCTGTAGTGGATTACGTGCCGGTCTGTTCCATCCTACCACCGGCTATTCACTGCCG
CTGGCGGTTGCCGTGGCCGACCGCCTGAGTGCACTTGATGTCTTTACGTCGGCCTCAATTCACCATGCCATTACGCATTTTGCCCGC
GAGCGCTGGCAGCAGCAGGGCTTTTTCCGCATGCTGAATCGCATGCTGTTTTTAGCCGGACCCGCCGATTCACGCTGGCGGGTTATG
CAGCGTTTTTATGGTTTACCTGAAGATTTAATTGCCCGTTTTTATGCGGGAAAACTCACGCTGACCGATCGGCTACGTATTCTGAGCGG
CAAGCCGCCTGTTCCGGTATTAGCAGCATTGCAAGCCATTATGACGACTCATCGTTAATAANNNNACTAGTAGCGGCCGCTGCAGTCC
GGCAAAAAAGGGCAAGGTGTCACCACCCTGCCCTTTTTCTTTAAAACCGAAAAGATTACTTCGCGTTATGCAGGCTTCCTCGCTCACT
GACTCGCTGCGCTCGGTCGTTCGGCTGCGGCGAGCGGTATCAGCTCACTCAAAGGCGGTAATACGGTTATCCACAGAATCAGGGGA
TAACGCAGGAAAGAACATGTGAGCAAAAGGCCAGCAAAAGGCCAGGAACCGTAAAAAGGCCGCGTTGCTGGCGTTTTTCCACAGGCT
CCGCCCCCCTGACGAGCATCACAAAAATCGACGCTCAAGTCAGAGGTGGCGAAACCCGACAGGACTATAAAGATACCAGGCGTTTCC
CCCTGGAAGCTCCCTCGTGCGCTCTCCTGTTCCGACCCTGCCGCTTACCGGATACCTGTCCGCCTTTCTCCCTTCGGGAAGCGTGGC
GCTTTCTCATAGCTCACGCTGTAGGTATCTCAGTTCGGTGTAGGTCGTTCGCTCCAAGCTGGGCTGTGTGCACGAACCCCCCGTTCA
GCCCGACCGCTGCGCCTTATCCGGTAACTATCGTCTTGAGTCCAACCCGGTAAGACACGACTTATCGCCACTGGCAGCAGCCACTGG
TAACAGGATTAGCAGAGCGAGGTATGTAGGCGGTGCTACAGAGTTCTTGAAGTGGTGGCCTAACTACGGCTACACTAGAAGAACAGT
ATTTGGTATCTGCGCTCTGCTGAAGCCAGTTACCTTCGGAAAAAGAGTTGGTAGCTCTTGATCCGGCAAACAAACCACCGCTGGTAGC
GGTGGTTTTTTTGTTTGCAAGCAGCAGATTACGCGCAGAAAAAAAGGATCTCAAGAAGATCCTTTGATCTTTTCTACGGGGTCTGACG
CTCAGTGGAACGAAAACTCACGTTAAGGGATTTTGGTCATGAGATTATCAAAAAGGATCTTCACCTAGATCCTTTTAAATTAAAAATGAA

	GTTTTAAATCAATCTAAAGTATATATGAGTAAACTTGGTCTGACAGCTCGAGGCTTGGATTCTCACCAATAAAAAACGCCCGGCGGCAA<br>CCGAGCGTTCTGAACAAATCCAGATGGAGTTCTGAGGTCATTACTGGATCTATCAACAGGAGTCCAAGCGAGCTCGATATCAAATTAC<br>GCCCCGCCCTGCCACTCATCGCAGTACTGTTGTAATTCATTAAGCATTCTGCCGACATGGAAGCCATCACAAACGGCATGATGAACCT<br>GAATCGCCAGCGGCATCAGCACCTTGTCGCCTTGCGTATAATATTTGCCCATGGTGAAAACGGGGGCGAAGAAGTTGTCCATATTGG<br>CCACGTTTAAATCAAAACTGGTGAAACTCACCCAGGGATTGGCTGAGACGAAAAACATATTCTCAATAAACCCTTTAGGGAAATAGGC<br>CAGGTTTTCACCGTAACACGCCACATCTTGCGAATATATGTGTAGAAACTGCCGGAAATCGTCGTGGTATTCACTCCAGAGCGATGAA<br>AACGTTTCAGTTTGCTCATGGAAAACGGTGTAACAAGGGTGAACACTATCCCATATCACCAGCTCACCGTCTTTCATTGCCATACGAAA<br>TTCCGGATGAGCATTCATCAGGCGGGCAAGAATGTGAATAAAGGCCGGATAAAACTTGTGCTTATTTTTCTTTACGGTCTTTAAAAAGG<br>CCGTAATATCCAGCTGAACGGTCTGGTTATAGGTACATTGAGCAACTGACTGAAATGCCTCAAAATGTTCTTTACGATGCCATTGGGAT<br>ATATCAACGGTGGTATATCCAGTGATTTTTTTCTCCATTTTAGCTTCCTTAGCTCCTGAAAATCTCGATAACTCAAAAAATACGCCCGGT<br>AGTGATCTTATTTCATTATGGTGAAAGTTGGAACCTCTTACGTGCCCGATCAACTCGAGTGCCACCTGACGTCTAAGAAACCATTATTA<br>TCATGACATTAACCTATAAAAATAGGCGTATCACGAGGCAGAATTTCAGATAAAAAAAATCCTTAGCTTTCGCTAAGGATGATTTCTGGA<br>ATTCGCGGCCGCTTCTAGAG	
pCAROT ENE5X	ATCACGAGGCCCTTTCGTCTTCACCTCGAGGATCCCGGGTACCTGCAGCTAGCGTCGACTGTGGTCTCTACAGGAATTCTTTCTTCTG<br>TCGCCGTCTTGTCAACGGCACCAGTTATATATTTCTTCTGTCGCCGTCTTGTCAACGGCACCAGTTATATATTGGTTTCTTCTGTCGCC<br>GTCTTGTCAACGGCACCAGTTATATATTTCTTCTGTCGCCGTCTTGTCAACGGCACCAGTTATATATTTCTTCTGTCGCCGTCTTGTCAA<br>CGGCACCAGTTATATATTGGGTTCCACCGGATACCTCCGGACTTGACAGCTAGCTCAGTCCTAGGGATTGTGCTAGCGAATTCATTAA<br>AGAGGAGAAAGGTACCATGACGGTCTGCGCAAAAAAACACGTTCATCTCACTCGCGATGCTGCGGAGCAGTTACTGGCTGATATTGA<br>TCGACGCCTTGATCAGTTATTGCCCGTGGAGGGAGAACGGGATGTTGTGGGTGCCGCGATGCGTGAAGGTGCGCTGGCACCGGGAA<br>AACGTATTCGCCCCATGTTGCTGTTGCTGACCGCCCGCGATCTGGGTTGCGCTGTCAGCCATGACGGATTACTGGATTTGGCCTGTG<br>CGGTGGAAATGGTCCACGCGGCTTCGCTGATCCTTGACGATATGCCCTGCATGGACGATGCGAAGCTGCGGCGCGGACGCCCTACC<br>ATTCATTCTCATTACGGAGAGCATGTGGCAATACTGGCGGCGGTTGCCTTGCTGAGTAAAGCCTTTGGCGTAATTGCCGATGCAGATG<br>GCCTCACGCCGCTGGCAAAAAATCGGGCGGTTTCTGAACTGTCAAACGCCATCGGCATGCAAGGATTGGTTCAGGGTCAGTTCAAGG<br>ATCTGTCTGAAGGGGATAAGCCGCGCAGCGCTGAAGCTATTTTGATGACGAATCACTTTAAAACCAGCACGCTGTTTTGTGCCTCCAT<br>GCAGATGGCCTCGATTGTTGCGAATGCCTCCAGCGAAGCGCGTGATTGCCTGCATCGTTTTTCACTTGATCTTGGTCAGGCATTTCAA<br>CTGCTGGACGATTTGACCGATGGCATGACCGACACCGGTAAGGATAGCAATCAGGACGCCGGTAAATCGACGCTGGTCAATCTGTTA<br>GGCCCGAGGGCGGTTGAAGAACGTCTGAGACAACATCTTCAGCTTGCCAGTGAGCATCTCTCTGCGGCCTGCCAACACGGGCACGC<br>CACTCAACATTTTATTCAGGCCTGGTTTGACAAAAAACTCGCTGCCGTCAGTTAATAATACTAGAGCTCAAGGAGGTACTAGATGAATA<br>ATCCGTCGTTACTCAATCATGCGGTCGAAACGATGGCAGTTGGCTCGAAAAGTTTTGCGACAGCCTCAAAGTTATTTGATGCAAAAAC<br>CCGGCGCAGCGTACTGATGCTCTACGCCTGGTGCCGCCATTGTGACGATGTTATTGACGATCAGACGCTGGGCTTTCAGGCCCGGC<br>AGCCTGCCTTACAAACGCCCGAACAACGTCTGATGCAACTTGAGATGAAAACGCGCCAGGCCTATGCAGGATCGCAGATGCACGAAC<br>CGGCGTTTGCGGCTTTTCAGGAAGTGGCTATGGCTCATGATATCGCCCCGGCTTACGCGTTTGATCATCTGGAAGGCTTCGCCATGG<br>ATGTACGCGAAGCGCAATACAGCCAACTGGATGATACGCTGCGCTATTGCTATCACGTTGCAGGCGTTGTCGGCTTGATGATGGCGC<br>AAATCATGGGCGTGCGGGATAACGCCACGCTGGACCGCGCCTGTGACCTTGGGCTGGCATTTCAGTTGACCAATATTGCTCGCGATA<br>TTGTGGACGATGCGCATGCGGGCCGCTGTTATCTGCCGGCAAGCTGGCTGGAGCATGAAGGTCTGAACAAAGAGAATTATGCGGCA<br>CCTGAAAACCGTCAGGCGCTGAGCCGTATCGCCCGTCGTTTGGTGCAGGAAGCAGAACCTTACTATTTGTCTGCCACAGCCGGCCTG<br>GCAGGGTTGCCCCTGCGTTCCGCCTGGGCAATCGCTACGGCGAAGCAGGTTTACCGGAAAATAGGTGTCAAAGTTGAACAGGCCGG<br>TCAGCAAGCCTGGGATCAGCGGCAGTCAACGACCACGCCCGAAAAATTAACGCTGCTGCTGGCCGCCTCTGGTCAGGCCCTTACTTC<br>CCGGATGCGGGCTCATCCTCCCCGCCCTGCGCATCTCTGGCAGCGCCCGCTCTAATAATACTAGAGCTCAAGGAGGTACTAGATGAA<br>ACCAACTACGGTAATTGGTGCAGGCTTCGGTGGCCTGGCACTGGCAATTCGTCTACAAGCTGCGGGGATCCCCGTCTTACTGCTTGA<br>ACAACGTGATAAACCCGGCGGTCGGGCTTATGTCTACGAGGATCAGGGGTTTACCTTTGATGCAGGCCCGACGGTTATCACCGATCC<br>CAGTGCCATTGAAGAACTGTTTGCACTGGCAGGAAAACAGTTAAAAGAGTATGTCGAACTGCTGCCGGTTACGCCGTTTTACCGCCTG<br>TGTTGGGAGTCAGGGAAGGTCTTTAATTACGATAACGATCAAACCCGGCTCGAAGCGCAGATTCAGCAGTTTAATCCCCGCGATGTC<br>GAAGGTTATCGTCAGTTTCTGGACTATTCACGCGCGGTGTTTAAAGAAGGCTATCTAAAGCTCGGTACTGTCCCTTTTTTATCGTTCAG<br>AGACATGCTTCGCGCCGCACCTCAACTGGCGAAACTGCAAGCATGGAGAAGCGTTTACAGTAAGGTTGCCAGTTACATCGAAGATGA<br>ACATCTGCGCCAGGCGTTTTCTTTCCACTCGCTGTTGGTGGGCGGCAATCCCTTCGCCACCTCATCCATTTATACGTTGATACACGCG<br>CTGGAGCGTGAGTGGGGCGTCTGGTTTCCGCGTGGCGGCACCGGCGCATTAGTTCAGGGGATGATAAAGCTGTTTCAGGATCTGGG<br>TGGCGAAGTCGTGTTAAACGCCAGAGTCAGCCATATGGAAACGACAGGAAACAAGATTGAAGCCGTGCATTTAGAGGACGGTCGCAG<br>GTTCCTGACGCAAGCCGTCGCGTCAAATGCAGATGTGGTTCATACCTATCGCGACCTGTTAAGCCAGCACCCTGCCGCGGTTAAGCA<br>GTCCAACAAACTGCAAACTAAGCGCATGAGTAACTCTCTGTTTGTGCTCTATTTTGGTTTGAATCACCATCATGATCAGCTCGCGCATC<br>ACACGGTTTGTTTCGGCCCGCGTTACCGCGAGCTGATTGACGAAATTTTTAATCATGATGGCCTCGCAGAGGACTTCTCACTTTATCT<br>GCACGCGCCCTGTGTCACGGATTCGTCACTGGCGCCTGAAGGTTGCGGCAGTTACTATGTGTTGGCGCCGGTGCCGCATTTAGGCA<br>CCGCGAACCTCGACTGGACGGTTGAGGGGCCAAAACTACGCGACCGTATTTTTGCGTACCTTGAGCAGCATTACATGCCTGGCTTAC<br>GGAGTCAGCTGGTCACGCACCGGATGTTTACGCCGTTTGATTTTCGCGACCAGCTTAATGCCTATCATGGCTCAGCCTTTTCTGTGGA<br>GCCCGTTCTTACCCAGAGCGCCTGGTTTCGGCCGCATAACCGCGATAAAACCATTACTAATCTCTACCTGGTCGGCGCAGGCACGCA<br>TCCCGGCGCAGGCATTCCTGGCGTCATCGGCTCGGCAAAAGCGACAGCAGGTTTGATGCTGGAGGATCTGATATAATAATACTAGAG<br>GAGGTACTAGATGCAACCGCATTATGATCTGATTCTCGTGGGGGCTGGACTCGCGAATGGCCTTATCGCCCTGCGTCTTCAGCAGCA	This study

	GCAACCTGATATGCGTATTTTGCTTATCGACGCCGCACCCCAGGCGGGCGGGAATCATACGTGGTCATTTCACCACGATGATTTGACT<br>GAGAGCCAACATCGTTGGATAGCTCCGCTGGTGGTTCATCACTGGCCCGACTATCAGGTACGCTTTCCCACACGCCGTCGTAAGCTG<br>AACAGCGGCTACTTTTGTATTACTTCTCAGCGTTTCGCTGAGGTTTTACAGCGACAGTTTGGCCCGCACTTGTGGATGGATACCGCGG<br>TCGCAGAGGTTAATGCGGAATCTGTTCGGTTGAAAAAGGGTCAGGTTATCGGTGCCCGCGCGGTGATTGACGGGCGGGGTTATGCG<br>GCAAATTCAGCACTGAGCGTGGGCTTCCAGGCGTTTATTGGCCAGGAATGGCGATTGAGCCACCCGCATGGTTTATCGTCTCCCATT<br>ATCATGGATGCCACGGTCGATCAGCAAAATGGTTATCGCTTCGTGTACAGCCTGCCGCTCTCGCCGACCAGATTGTTAATTGAAGACA<br>CGCACTATATTGATAATGCGACATTAGATCCTGAATGCGCGCGGCAAAATATTTGCGACTATGCCGCGCAACAGGGTTGGCAGCTTCA<br>GACACTGCTGCGAGAAGAACAGGGCGCCTTACCCATTACTCTGTCGGGCAATGCCGACGCATTCTGGCAGCAGCGCCCCCTGGCCT<br>GTAGTGGATTACGTGCCGGTCTGTTCCATCCTACCACCGGCTATTCACTGCCGCTGGCGGTTGCCGTGGCCGACCGCCTGAGTGCA<br>CTTGATGTCTTTACGTCGGCCTCAATTCACCATGCCATTACGCATTTTGCCCGCGAGCGCTGGCAGCAGCAGGGCTTTTTCCGCATGC<br>TGAATCGCATGCTGTTTTTAGCCGGACCCGCCGATTCACGCTGGCGGGTTATGCAGCGTTTTTATGGTTTACCTGAAGATTTAATTGC<br>CCGTTTTTATGCGGGAAAACTCACGCTGACCGATCGGCTACGTATTCTGAGCGGCAAGCCGCCTGTTCCGGTATTAGCAGCATTGCA<br>AGCCATTATGACGACTCATCGTTAATAAACTAGTTAAGCGGCCGCTCTAGAGGCATCAAATAAAACGAAAGGCTCAGTCGAAAGACTG<br>GGCCTTTCGTTTTATCTGTTGTTTGTCGGTGAACGCTCTCCTGAGTAGGACAAATCCGCCGCCCTAGACCTAGGCGGAGATATTCCGC<br>TTCCTCGCTCACTGACTCGCTCCGCTCGGTCGTTCGGCTGCGGCGAGCGGTACCGGCTTACTGGCGGGGCGGAAATTTCCTGGAAG<br>ATGCCAGGAAGAAACTTAACAGGGAAGCGATAAGGCCACGGCAGAGCCGTTTTTCCATAGGCTCCGCCCCCCTGACAAGCATCACGA<br>AATCTGACGCTCAAATCAGTGGTGGCGAAACCCGACAGGACTTAAAGATACCAGGCGTTTCCCCCTGGTGGCTCCCTCGTGCGCTCT<br>CCTGTTCCTGCCTTTCGGTTTACCGGTGTCACTCCGCTGTTATGGCCGCGTTTATCTCATTCCACGCCTGACACTCGGTTCCGGGAAG<br>GCAGTTCGCTCCAAGCTGGACTGTATGCACGAACCCCCCGTTCAGTCCGACTGCTGCGCCTGTTCCGGTAACTATCGACTTGAGTCC<br>AAGCCGGAAAGACACGACAAAGCGCCACTGGCAGCAGCCACTGGTAAGAGGGTGTACAAGAGATTACTGATGCAGAGTTCTTGAAGT<br>CAGAGCCAAACTACGGCTAAACTGGAAGGACGAATTTGGTGGCAGCGGTCTTGTACACCCTGTTACCACGGTTCAGAAGTTCCCCAA<br>CTTACTGAACCTTCGAAAAACCACCTCCCCAGGTGGTTTTTTCGTTTTCAGAGCAAGAGATTACGACGCAACGGAAAGGATCTCAAGA<br>GGTTCATCTTACTAGTGCTTGGATTCTCACCAATAAAAAACGCCCGGCGGCAACCGAGCGTTCTGAACAAATCCAGATGGAGTTCTGA<br>GGTCATTACTGGATCTATCAACAGGAGTCCAAGCGAGCTCGTAAACTTGGTCTGACAGTTACCAATGCTTAATCAGTGAGGCACCTAT<br>CTCAGCGATCTGTCTATTTCGTTCATCCATAGTTGCCTGACTCCCCGTCGTGTAGATAACTACGATACGGGAGGGCTTACCATCTGGC<br>CCCAGTGCTGCAATGATACCGCGAGACCCACGCTCACCGGCTCCAGATTTATCAGCAATAAACCAGCCAGCCGGAAGGGCCGAGCG<br>CAGAAGTGGTCCTGCAACTTTATCCGCCTCCATCCAGTCTATTAATTGTTGCCGGGAAGCTAGAGTAAGTAGTTCGCCAGTTAATAGTT<br>TGCGCAACGTTGTTGCCATTGCTACAGGCATCGTGGTGTCACGCTCGTCGTTTGGTATGGCTTCATTCAGCTCCGGTTCCCAACGATC<br>AAGGCGAGTTACATGATCCCCCATGTTGTGCAAAAAAGCGGTTAGCTCCTTCGGTCCTCCGATCGTTGTCAGAAGTAAGTTGGCCGCA<br>GTGTTATCACTCATGGTTATGGCAGCACTGCATAATTCTCTTACTGTCATGCCATCCGTAAGATGCTTTTCTGTGACTGGTGAGTACTC<br>AACCAAGTCATTCTGAGAATAGTGTATGCGGCGACCGAGTTGCTCTTGCCCGGCGTCAATACGGGATAATACCGCGCCACATAGCAG<br>AACTTTAAAAGTGCTCATCATTGGAAAACGTTCTTCGGGGCGAAAACTCTCAAGGATCTTACCGCTGTTGAGATCCAGTTCGATGTAAC<br>CCACTCGTGCACCCAACTGATCTTCAGCATCTTTTACTTTCACCAGCGTTTCTGGGTGAGCAAAAACAGGAAGGCAAAATGCCGCAAA<br>AAAGGGAATAAGGGCGACACGGAAATGTTGAATACTCATACTCTTCCTTTTTCAATATTATTGAAGCATTTATCAGGGTTATTGTCTCAT<br>GAGCGGATACATATTTGAATGTATTTAGAAAAATAAACAAATAGGGGTTCCGCGCACATTTCCCCGAAAAGTGCCACCTGACGTCTAA<br>GAAACCATTATTATCATGACATTAACCTATAAAAATAGGCGT	

Table S3. Sequence of oligonucleotides used in present study.

Oligoneucleotide name	Sequence (3'- 5')
DaraBAD-sfGFP-rev	TTCATCAACGCGCCCCCCATGGGACGCGTTTTTAGAGGCACCTAGGTCTAGGGCGGCGGA
DaraBAD-sfGFP-fv	ACTGTTTCTCCATACCTGTTTTTCTGGATGGAGTAAGACGATGTCTAAAGGTGAAGAACT
ParaE-tetRA-fv	TAATAGCTATGATCAATGCTTTAATTCATTGAAATATTGGTTAAGACCCACTTTCACATT
ParaE-tetRA-rev	AACGCGGCGTTAAAGCAGAGTCATGATTAATAGAGACCATCTAAGCACTTGTCTCCTGTT
PproX-araE-fv	TAATAGCTATGATCAATGCTTTAATTCATTGAAATATTGGCAGCTAACACCACGTCGTCC
PproX-araE-rev	AACGCGGCGTTAAAGCAGAGTCATGATTAATAGAGACCATCTAGTACTTTCCTGTGTGAA
N-dCas9$_{opt}$	ATGGATAAGAAGTACTCCATTGGTTTAGCAATTGGCACGAACAGTGTCGGATGGGCCGTGATTACAGATGAATACAAGGTCCCTTC<br>AAAGAAATTCAAAGTGTTAGGTAATACTGACCGGCACAGTATTAAGAAAAACCTGATCGGGGCACTTCTGTTCGATTCCGGGGAAAC<br>TGCAGAGGCAACACGTTTGAAGCGTACCGCCCGGCGCCGTTATACGCGTCGTAAAAATCGCATCTGTTACTTACAAGAAATCTTCT<br>CAAATGAGATGGCGAAGGTAGACGATTCTTTTTTTCATCGGCTGGAGGAATCGTTTTTGGTAGAAGAGGACAAGAAGCATGAACGC<br>CATCCGATTTTCGGGAATATTGTCGATGAAGTTGCCTATCATGAGAAATATCCGACAATTTACCATTTACGGAAAAAACTTGTGGATT

	CTACGGACAAAGCTGACTTGCGGTTGATCTATCTTGCCCTGGCCCACATGATTAAGTTCCGTGGGCACTTTCTTATCGAAGGGGATT<br>TGAACCCAGATAACTCGGACGTTGACAAGTTGTTTATTCAATTGGTCCAAACCTATAACCAGCTTTTCGAGGAAAATCCGATCAATG<br>CTAGCGGTGTGGACGCGAAAGCTATCCTGAGTGCCCGGTTATCTAAATCACGGCGGCTGGAGAACTTGATTGCCCAGTTGCCCGG<br>AGAGAAGAAAAACGGATTATTCGGTAATCTTATCGCCCTTTCTCTGGGGTTGACGCCCAACTTCAAATCTAACTTTGATCTGGCCGA<br>AGACGCTAAATTACAACTTAGTAAAGATACCTACGACGATGACCTGGATAATTTGTTGGCTCAAATCGGGGATCAATATGCTGACCT<br>TTTCCTTGCGGCGAAGAACTTATCGGACGCCATCTTGCTGTCGGATATCCTGCGTGTCAATACGGAGATTACTAAGGCACCACTTTC<br>CGCATCCATGATCAAGCGTTATGACGAGCATCACCAAGACCTTACGTTGCTGAAAGCCTTAGTCCGTCAACAGTTACCTGAAAAATA<br>TAAAGAAATTTTTTTTGATCAGTCTAAGAATGGATACGCAGGTTATATCGACGGAGGAGCAAGCCAAGAAGAATTTTACAAATTTATT<br>AAACCGATTCTTGAAAAGATGGATGGTACGGAGGAACTTCTGGTTAAGTTAAATCGCGAAGACTTGTTGCGCAAGCAGCGCACTTT<br>CGACAACGGGAGTATCCCCCACCAAATTCATCTTGGTGAATTACATGCGATCCTGCGGCGGCAAGAAGATTTTTATCCTTTCTTAAA<br>AGATAATCGCGAAAAAATTGAAAAAATCTTAACGTTTCGCATTCCTTATTACGTGGGCCCTCTGGCACGCGGTAATAGCCGGTTTGC<br>CTGGATGACCCGCAAATCAGAAGAGACCATCACCCCGTGGAATTTCGAGGAGGTAGTAGACAAGGGTGCCAGTGCACAATCCTTC<br>ATCGAACGCATGACAAATTTTGATAAAAATTTGCCTAATGAGAAGGTCCTGCCGAAACATAGTCTTTTATATGAATACTTTACCGTATA<br>CAACGAGTTGACAAAGGTTAAGTACGTGACGGAGGGGATGCGTAAACCTGCATTTTTGTCAGGTGAGCAAAAAAAAGCGATTGTGG<br>ATTTATTATTTAAAACTAATCGGAAGGTGACAGTGAAGCAACTTAAGGAAGACTATTTCAAGAAGATCGAATGCTTCGATTCGGTAGA<br>AATCAGTGGTGTTGAAGACCGCTTCAACGCAAGCTTAGGGACTTATCACGATCTTCTTAAGATCATTAAGGACAAAGACTTCCTGGA<br>CAACGAGGAAAACGAGGACATTTTGGAAGATATTGTACTTACTTTGACTTTGTTTGAAGACCGGGAGATGATCGAAGAGCGTCTTAA<br>GACGTACGCGCACCTTTTCGATGACAAAGTAATGAAGCAGCTGAAGCGGCGGCGGTACACCGGATGGGGTCGTTTATCTCGGAAA<br>TTGATCAAT
N-dCas9-fv	GATCGCTTGCCTGTAACTTACACGCGCCTCGTGGGGCGCGCCGTCGACATGGATAAGAAGTACTCCAT
N-dCas9-rv	GATCTTTTCTACGGGGCCGGCCCCTGCAGGGTTTGTTTAAACTCTAGAATTGATCAATTTCCGAGATA
C-*dCas9*$_{opt}$-*T*$_{rrnBT1}$	GGCATTCGTGACAAGCAAAGCGGCAAGACGATCTTAGATTTCCTTAAGTCCGATGGATTTGCTAATCGGAACTTTATGCAATTGATT<br>CATGACGATAGTCTTACCTTTAAAGAGGACATCCAAAAGGCACAAGTGTCAGGCCAAGGTGACAGTCTTCACGAACATATTGCTAAC<br>TTAGCCGGTAGTCCAGCTATCAAGAAGGGTATCTTACAGACTGTTAAAGTAGTAGATGAACTGGTGAAAGTAATGGGTCGTCACAAG<br>CCTGAGAATATCGTAATTGAAATGGCGCGGGAAAATCAGACAACTCAAAAAGGTCAAAAAAACAGTCGTGAACGTATGAAACGCATT<br>GAAGAGGGCATCAAGGAGTTGGGCAGTCAGATTTTAAAGGAGCATCCAGTGGAGAACACTCAGCTGCAAAATGAGAAACTTTATCT<br>TTATTACCTTCAGAATGGACGCGATATGTACGTCGATCAAGAACTGGATATTAATCGGTTATCCGATTACGACGTTGATGCCATCGT<br>CCCTCAATCATTTCTGAAAGACGATAGCATCGACAATAAGGTGCTGACACGTAGTGACAAGAACCGTGGGAAGAGCGACAACGTAC<br>CGTCGGAGGAGGTCGTTAAGAAGATGAAGAACTATTGGCGGCAATTGCTGAACGCTAAATTGATTACGCAGCGGAAATTTGACAAC<br>CTGACCAAGGCAGAGCGTGGGGGCCTGAGTGAACTGGATAAAGCTGGCTTTATCAAACGTCAATTGGTAGAGACACGCCAAATTAC<br>GAAGCATGTCGCGCAGATTTTGGACTCGCGCATGAACACTAAGTACGATGAAAACGACAAGTTAATTCGTGAGGTGAAAGTCATTA<br>CTCTTAAGAGTAAACTTGTCTCGGACTTCCGGAAGGACTTTCAATTTTACAAAGTCCGTGAAATTAATAACTATCATCACGCCCACGA<br>CGCATATCTTAACGCGGTCGTTGGAACAGCTTTGATTAAGAAATATCCCAAGCTTGAGAGCGAGTTCGTGTACGGTGATTATAAAGT<br>GTATGACGTACGTAAGATGATCGCGAAGTCCGAACAAGAGATTGGGAAGGCAACTGCTAAGTACTTTTTTTACTCTAATATTATGAA<br>CTTTTTCAAAACCGAGATCACATTGGCTAATGGCGAAATCCGTAAGCGCCCTCTTATTGAGACAAACGGGGAAACTGGGGAGATCG<br>TGTGGGATAAGGGCCGCGATTTTGCAACTGTCCGGAAGGTCCTGTCTATGCCCCAAGTGAACATCGTTAAGAAGACGGAAGTTCAG<br>ACCGGGGGCTTTTCAAAGGAGAGTATTTTACCCAAGCGCAACAGTGACAAGTTGATCGCGCGGAAGAAGGACTGGGATCCCAAAA<br>AGTATGGGGGATTCGACTCACCGACCGTTGCATACTCCGTGCTGGTCGTCGCGAAAGTGGAGAAGGGCAAGAGTAAAAAACTGAA<br>GTCGGTTAAGGAGTTGCTTGGCATCACTATTATGGAACGTAGTAGCTTCGAGAAGAATCCAATCGATTTTTTAGAAGCCAAGGGCTA<br>CAAAGAAGTTAAGAAAGACCTTATTATCAAGCTGCCAAAGTACTCACTTTTCGAATTAGAAAACGGACGTAAGCGCATGTTAGCGTC<br>CGCTGGTGAACTGCAAAAAGGAAATGAGCTGGCACTGCCTTCTAAATACGTGAACTTTTTATATTTGGCGTCCCATTACGAAAAGCT<br>TAAGGGGTCGCCCGAAGATAACGAGCAAAAGCAGCTGTTTGTGGAACAACACAAGCATTATCTGGATGAGATCATCGAGCAGATTA<br>GCGAATTCTCAAAGCGGGTGATCTTAGCCGATGCTAACTTGGATAAAGTGCTGTCCGCTTACAACAAACATCGCGATAAGCCCATC<br>CGTGAGCAGGCGGAGAATATCATTCATCTGTTTACATTGACGAATTTGGGGGCCCCCGCTGCATTCAAGTACTTCGATACTACAATC<br>GACCGTAAACGTTACACTAGTACAAAAGAAGTTTTGGACGCTACCCTGATCCATCAGAGTATCACTGGCCTGTACGAGACACGCAT<br>CGACTTGTCCCAATTGGGGGGGGATTAACTCGAGTAAGGATCTCCAGGCATCAAATAAAACGAAAGGCTCAGTCGAAAGACTGGG<br>CCTTTCGTTTTATCTGTTGTTTGTCGGTGAACGCTCTCTACTAGAGTCACACTGGCTCACCTTCGGGTGGGCCTTTCTGCGTTTATA
C-dCas9-fv	CCGGATGGGGTCGTTTATCTCGGAAATTGATCAATGGCATTCGTGACAAGCAAAG
C-dCas9-rv	GATCTTTTCTACGGGGCCGGCCCCTGCAGGGTTTTATAAACGCAGAAAGGCCCA
gBlock-LacI-AraC-ParaB-fv	TTGCCTGTAACTTACACGCG
gBlock-LacI-AraC-ParaB-rv	GCCAATTGCTAAACCAATGG

dCas9-fv	GGAATTGGGGATCGGAATTCGAGCTCGGTACCCGGGGATCCACTTTTCATACTCCCGCCA
dCas9-rv	CCATGCATCTCGAGGCATGCCTGCAGCGGCCGCTACTAGTTTTCTACGGGGCCGGCCCCT
BBa_J23119-gRNA-J106-fv	TTTTACGGTTCCTGGCCTTTTGCTGGCCTTTTGCTCTCAGATAAAATATTTCTAGA
BBa_J23119-gRNA-J106-rv	CCTATAAAAATAGGCGTATCACGAGGCCCTTTCGTCTCGGCTGGCGATTCAGGTTCATC
PUC19-fv	GGCCAGCAAAAGGCCAGGAA
PUC19-rv	GAGACGAAAGGGCCTCGTGA
crRNA-J105-fv	GTTACCAAAGGCGTCCTTGTTTTAGAGCTAGAAATAGCAAGTTAAAATAAGGCTAGTCCG
crRNA-J105-rev	TTTCTAGCTCTAAAACAAGGACGCCTTTGGTAACCGACTAGTATTATACCTAGGAC
J1-RFP-fv	TTTATAGGTTAATGTCATGATAATAATGGTTTCTTACCTCCCGTGGGGAAAAAATC
J1-RFP-rv	GAGTAAGTAGTTCGCCAGTTAATAGTTTGCGCAACGTTGTTGCCATTGCTACAGGC
TET-R-fv	CTCAGTACAATCTGCTCTGATGCCGCATAGTTAAGCTTCTCTATCACTGATAGGG
TET-R-rv	TTGGAGTGAACGCCGTTTCCATTTAGGTGGGTAC
MCP-SOX-fv	ACGTACCCACCTAAATGGAAACGGCGTTCACTCCAAGCGAAAAAACCCCGCCGAAG
MCP-SOX-rv	GCAAAAAAGGGAATAAGGGCGACACGGAAATGTTGAACTTTTCATACTCCCGCCATT
pAra_optMCP_linker_optSoxA_R93A_BBa_B002, gBlock #104	GCGAAAAAACCCCGCCGAAGCGGGGTTTTTTGCGTTAAAGCCGGTGACGGTAGTCGCTGGGAGTCCGGTCAAATTGACGAGCGAA GACGCGAGAGAAGGTTTGCTGCGACACATAGCCTAAGTCCATGGCAATATCGAAAATCGGACGTTCTGTTGTCCGCAATTCAACGG CAGCCAACAACAAACGGCGTTGACGGATATAGTCTCCTAATGTTTGATGTGTGACGGTACGAAACATGCGCTGTAAATACCACTTCG AGTAACCACTTTTCTTCGCTACAACATCAATATTAAGAGGCTGATCAATATGCTCATCGATCCAAGCAATAAGGTCCTGGATGATTTT TTGGTGGCTCATTGAACCACCTCCGCCGTAAATACCACTGTTGGCCGCGATGGCAGATGGAATCGGATTTCCATCTTTCAGAAGAC CCTGCATCGCTTTCACAATCAACTCACAGTCGCTATTCGTAGCAAAGATCGGAATAGTCAGTTCCATATTAAGGTACGAACGCCATG CACCTTTAGGGACTTCAACTTTGATCGTGTACTTACGGTTTTGCGCACTCGATTGACGTACAGAACAAGTAACTTTGTATGCTTGGCT ACGGCTGTTGGAGGAAATCCACTCGGCAATGCCGTTAGCGAAGTTGCTAGGGGCAACAGTTACATCACCAGTACCACCATTATCCA CAAGAACGAACTGCGTAAAATTGCTCGCGGGACCCATAGATCCTTTCTCCTCTTTAGATCTTTTGAATTCCCAAAAAAACGGGTATG GAGAAACAGTAGAGAGTTGCGATAAAAAGCGTCAGGTAGAATCCGCTAATCTTATGGATAAAAATGCTATGGCATAGCAAAGTGTGA CGCCGTGCAAATAATCAATGTGGACTTTTCTGCCGTGATTATAGACACTTTTGTTACGCGTTTTTGTCATGGCTTTGGTCCCGCTTTG TTACAGAATGCTTTTAATAAGCGGGGTTACCGGTTTGGTTAGCGAGAAGAGCCAGTAAAAGACGCAGTGACGGCAATGTCTGATGC AATATGGACAATTGGTTTCTTCTCTGAATGGCGGGAGTATGAAAAG
TET-MCP-SOX-fv	AGCGTCAGACCCCGTAGAAAAGATCAAAGGATCTTCAAGCAGCATAACCTTTTTCC
TET-MCP-SOX-rv	AAGCAGCAGATTACGCGCAGAAAAAAAGGATCTCAACTTTTCATACTCCCGCCATT
J-crRNA-fv	GAAGATCCTTTGATCTTTTCTACGG
J-crRNA-rv	TTGAGATCCTTTTTTTCTGCGCG
J1-RFP-2x-fv	TTTATAGGTTAATGTCATGATAATAATGGTTTCTTACCTAGGTATAAACGCAGAAA
crRNA-J107-fv	GTGTCCTGCGGTTACCAAGTTTTAGAGCTAGAAATAGCAAGTTAAAATAAGGCTAGTCCG
crRNA-J107-rev	TTTCTAGCTCTAAAACTTGGTAACCGCAGGACACCGACTAGTATTATACCTAGGAC
crRNA-J108-fv	GTAACCGCAGGACACCGCGTTTTAGAGCTAGAAATAGCAAGTTAAAATAAGGCTAGTCCG
crRNA-J108-rev	TTTCTAGCTCTAAAACGCGGTGTCCTGCGGTTACCAACTAGTATTATACCTAGGAC
crRNA-J111-fv	GCGACCTCAGGTATCCTGGTTTTAGAGCTAGAAATAGCAAGTTAAAATAAGGCTAGTCCG
crRNA-J111-rev	TTTCTAGCTCTAAAACCAGGATACCTGAGGTCGCCCACTAGTATTATACCTAGGAC

PBAD-SOX-fv	AGATCCTTTCTCCTCTTTAGA
PBAD-SOX-rv	GGCGGAGGTGGTTCAATGAGCCACC
JUB-fv	GATGATTTTTTGGTGGCTCATTGAACCACCTCCGCCGGGTTTAGTGTTGCCATCTA
JUB-rv	CTCTACTGTTTCTCCATACCCGTTTTTTTGGGAATTCAAAAGATCTAAAGAGGAGAAAGGATCTGTCGAC
JUBDBD-fv	ATGATTTTTTGGTGGCTCATTGAACCACCTCCGCCGTTTCTTTGAGATGTGACTC
GRF-fv	GATGATTTTTTGGTGGCTCATTGAACCACCTCCGCCCCCTTGAGGAGCAAAATTCC
ANAC-fv	GATGATTTTTTGGTGGCTCATTGAACCACCTCCGCCAACACCTGGTGAAAACAAAGAC
GRF-fv	
JUB0X-rv	CTTCACCTCGAGGATCCCGGGTACCTGCAGCTAGCTGTTCCACCGGATACCTCCGG
JUB0X-fv	CTAGCACAATCCCTAGGACTGAGCTAGCTGTCAAGTCCGGAGGTATCCGGTGGAAC
BS-fv	GTCTTCACCTCGAGGATCCCGGGTACCTGCAGCTAGCGTCGACTGTGGTCTCTACAGG
BS-rv	CCGGAGGTATCCGGTGGAAC
JUB5X-fv	TTTCTTCTGTCGCCGTCTTGTCAACGGCAC
JUB5X-rv	GTGCCGTTGACAAGACGGCGACAGAAGAAAGAATTCCTGTAGAGACCACA
plantTF-fv	GGAATTCGAGCTCGGTACCCGGGGATCCGCAAAAAAGGGAATAAGGG
plantTF-rv	CCATGCATCTCGAGGCATGCCTGCAGCGGCCGCTACTAGTGCGAAAAAACCCCGCCGAAG
RamR-Kan-fv	AGTAATGCTTGTTTTTTGTCTTCACTCTTCGGACGAGCCAAGGGTTTTCCCAGTCACGAC
RamR-Kan-rv	GCAATATGCTTTTCTACCACTTCATGCGGCAGCCCTTGATTGCTTCCGGCTCGTATGTTG
SELEX alkaloid sensor	AGTAATGCTTGTTTTTTGTCTTCACTCTTCGGACGAGCCATTACACGTTACCCTTATGTCTGGAAAAACATGATTGAATCATGCCCGT<br>TGTCGCGTCGCAACGGTGAATGTCAACCTTTGAAAAGTACCTTGACGGCGTATCTTTGCTTTCTATAATGAGTGCTTACTCACTCATA<br>AGCTGTCACCGGATGTGCTTTCCGGTCTGATGAGTCCGTGAGGACGAAACAGCCTCTACAAATAATTTTGTTTAAGCTAGCATTAAA<br>CAGGATAATAAGCACTAGAATGTCTAAAGGTGAAGAACTGTTCACCGGTGTTGTTCCGATCCTGGTTGAACTGGATGGTGATGTTAA<br>CGGCCACAAATTCTCTGTTCGTGGTGAAGGTGAAGGTGATGCAACCAACGGTAAACTGACCCTGAAATTCATCTGCACTACCGGTA<br>AACTGCCGGTTCCATGGCCGACTCTGGTGACTACCCTGACCTATGGTGTTCAGTGTTTTTCTCGTTACCCGGATCACATGAAGCAG<br>CATGATTTCTTCAAATCTGCAATGCCGGAAGGTTATGTACAGGAGCGCACCATTTCTTTCAAAGACGATGGCACCTACAAAACCCGT<br>GCAGAGGTTAAATTTGAAGGTGATACTCTGGTGAACCGTATTGAACTGAAAGGCATTGATTTCAAAGAGGACGGCAACATCCTGGG<br>CCACAAACTGGAATATAACTTCAACTCCCATAACGTTTACATCACCGCAGACAAACAGAAGAACGGTATCAAAGCTAACTTCAAAATT<br>CGCCATAACGTTGAAGACGGTAGCGTACAGCTGGCGGACCACTACCAGCAGAACACTCCGATCGGTGATGGTCCGGTTCTGCTGC<br>CGGATAACCACTACCTGTCCACCCAGTCTGTTCTGTCCAAAGACCCGAACGAAAAGCGCGACCACATGGTGCTGCTGGAGTTCGTT<br>ACTGCAGCAGGTATCACGCACGGCATGGATGAGCTCTACAAATGATAAGCGGCCGCTCTAGAGGCATCAAATAAAACGAAAGGCTC<br>AGTCGAAAGACTGGGCCTTTCGTTTTATCTGTTGTTTGTCGGTGAACGCTCTCCTGAGTAGGACAAATCCGCCGCCCTAGACCTAG<br>GATCAAGGGCTGCCGCATGAAGTGGTAGAAAAGCATATTGC
RamR-Sensor-fv	AGTAATGCTTGTTTTTTGTCTTCACTCTTCGG
RamR-Sensor-rv	GCAATATGCTTTTCTACCACTTCATGC
ATF alkaloid sensor	AGTAATGCTTGTTTTTTGTCTTCACTCTTCGGACGAGCCATGGTACCTTTCTCCTCTTTAATGAATTCGCTAGCACAATCCCTAGGAC<br>TGAGCTAGCTGTCAAGTCCGGAGGTATCCGGTGGAACACCAATATATAACTGGTGCCGTTGACAAGACGGCGACAGAAGAAATATA<br>TAACTGGTGCCGTTGACAAGACGGCGACAGAAGAAACCAATATATAACTGGTGCCGTTGACAAGACGGCGACAGAAGAAATATATA<br>ACTGGTGCCGTTGACAAGACGGCGACAGAAGAAAGAATTCCTGTAGAGACCACAGTCGACGCTAGCTGCAGGTACCTTTGAAAAGT<br>ACCTTGACGGCGTATCTTTGCTTTCTATAATGAGTGCTTACTCACTCATAAGCTGTCACCGGATGTGCTTTCCGGTCTGATGAGTCC<br>GTGAGGACGAAACAGCCTCTACAAATAATTTTGTTTAAGCTAGCATTAAACAGGATAATAAGCACTAGAATGTCTAAAGGTGAAGAA<br>CTGTTCACCGGTGTTGTTCCGATCCTGGTTGAACTGGATGGTGATGTTAACGGCCACAAATTCTCTGTTCGTGGTGAAGGTGAAGG<br>TGATGCAACCAACGGTAAACTGACCCTGAAATTCATCTGCACTACCGGTAAACTGCCGGTTCCATGGCCGACTCTGGTGACTACCC<br>TGACCTATGGTGTTCAGTGTTTTTCTCGTTACCCGGATCACATGAAGCAGCATGATTTCTTCAAATCTGCAATGCCGGAAGGTTATG<br>TACAGGAGCGCACCATTTCTTTCAAAGACGATGGCACCTACAAAACCCGTGCAGAGGTTAAATTTGAAGGTGATACTCTGGTGAAC<br>CGTATTGAACTGAAAGGCATTGATTTCAAAGAGGACGGCAACATCCTGGGCCACAAACTGGAATATAACTTCAACTCCCATAACGTT

	TACATCACCGCAGACAAACAGAAGAACGGTATCAAAGCTAACTTCAAAATTCGCCATAACGTTGAAGACGGTAGCGTACAGCTGGCGGACCACTACCAGCAGAACACTCCGATCGGTGATGGTCCGGTTCTGCTGCCGGATAACCACTACCTGTCCACCCAGTCTGTTCTGTCCAAAGACCCGAACGAAAAGCGCGACCACATGGTGCTGCTGGAGTTCGTTACTGCAGCAGGTATCACGCACGGCATGGATGAGCTCTACAAATGATAAGCGGCCGCTCTAGAGGCATCAAATAAAACGAAAGGCTCAGTCGAAAGACTGGGCCTTTCGTTTTATCTGTTGTTTGTCGGTGAACGCTCTCCTGAGTAGGACAAATCCGCCGCCCTAGACCTAGGATCAAGGGCTGCCGCATGAAGTGGTAGAAAAGCATATTGC
5X-fr	ATTGCAAGCCATTATGACGACTCATCGTTAATAAACTAGTTAAGCGGCCGCTCTAGAGGC
5X-rv	GAGTGAGATGAACGTGTTTTTTTGCGCAGACCGTCATGGTACCTTTCTCCTCTTTAA
K2151200-fv	ATGACGGTCTGCGCAAAAAA
K2151200-rv	TTATTAACGATGAGTCGTCA

References

1. Dong C, Fontana J, Patel A, Carothers JM, Zalatan JG. Synthetic CRISPR-Cas gene activators for transcriptional reprogramming in bacteria. *Nat Commun* **9**, 2489 (2018).

2. Ho HI, Fang JR, Cheung J, Wang HH. Programmable CRISPR-Cas transcriptional activation in bacteria. *Mol Syst Biol* **16**, e9427 (2020).

3. Lian J, HamediRad M, Hu S, Zhao H. Combinatorial metabolic engineering using an orthogonal tri-functional CRISPR system. *Nat Commun* **8**, 1688 (2017).

4. Datsenko KA, Wanner BL. One-step inactivation of chromosomal genes in <i>Escherichia coli</i> K-12 using PCR products. *PNAS* **97**, 6640-6645 (2000).

5. Bochner BR, Huang HC, Schieven GL, Ames BN. Positive selection for loss of tetracycline resistance *J Bacteriol* **143**, 926 (1980).

6. Hoffmann S, Schmidt C, Walter S, Bender JK, Gerlach RG. Scarless deletion of up to seven methyl-accepting chemotaxis genes with an optimized method highlights key function of CheM in Salmonella Typhimurium. *PLoS One* **12**, e0172630 (2017).

7. Naseri G, Behrend J, Rieper L, Mueller-Roeber B. COMPASS for rapid combinatorial optimization of biochemical pathways based on artificial transcription factors. *Nat Commun* **10**, (2019).

8. St-Pierre F, Cui L, Priest DG, Endy D, Dodd IB, Shearwin KE. One-Step Cloning and Chromosomal Integration of DNA. *ACS Synth Biol* **2**, 537-541 (2013).

9. Naseri G, Prause K, Hamdo HH, Arenz C. Artificial Transcription Factors for Tuneable Gene Expression in Pichia pastoris. *Front Bioeng Biotechnol* **9**, 676900 (2021).

10. Strauch E, Voigt I, Broll H, Appel B. Use of a plasmid of a Yersinia enterocolitica biogroup 1A strain for the construction of cloning vectors. *J Biotechnol* **79**, 63-72 (2000).

11. St-Pierre F, Cui L, Priest DG, Endy D, Dodd IB, Shearwin KE. One-step cloning and chromosomal integration of DNA. *ACS Synth Biol* **2**, 537-541 (2013).
