# Supplementary material for: A versatile regulatory toolkit of arabinose-inducible artificial transcription factors for Enterobacteriaceae
Source: Commun Biol. 2023 Oct 3;6:1005. doi: 10.1038/s42003-023-05363-3 (PMC10547716; doi:10.1038/s42003-023-05363-3)
Supplement: Supplementary file 3 — Description of Supplementary Materials [file 42003_2023_5363_MOESM3_ESM.docx]

**Description of Additional Supplementary Files**

**File name:** Supplementary Data 1

**Description:** The source data behind the Figure 2.

**File name:** Supplementary Data 2

**Description:** The source data behind the Supplementary Figure 2.

**File name:** Supplementary Data 3

**Description:** The source data behind the Supplementary Figure 3.

**File name:** Supplementary Data 4

**Description:** The source data behind the Figure 3.

**File name:** Supplementary Data 5

**Description:** The source data behind the Supplementary Figure 4.

**File name:** Supplementary Data 6

**Description:** The source data behind the Supplementary Figure 6.

**File name:** Supplementary Data 7

**Description:** The source data behind the Supplementary Figure 7.

**File name:** Supplementary Data 8

**Description:** The source data behind the Supplementary Figure 8.

**File name:** Supplementary Data 9

**Description:** The source data behind the Supplementary Figure 11.

**File name:** Supplementary Data 10

**Description:** The source data behind the Supplementary Figure 13.

**File name:** Supplementary Data 11

**Description:** The source data behind the Figure 4.

**File name:** Supplementary Data 12

**Description:** The source data behind the Supplementary Figure 14.

**File name:** Supplementary Data 13

**Description:** The source data behind the Supplementary Figure 15.

**File name:** Supplementary Data 14

**Description:** The source data behind the Supplementary Figure 16.

**File name:** Supplementary Data 15

**Description:** The source data behind the Supplementary Figure 17.

**File name:** Supplementary Data 16

**Description:** The source data behind the Supplementary Figure 18.

**File name:** Supplementary Data 17

**Description:** The source data behind the Supplementary Figure 19.

**File name:** Supplementary Data 18

**Description:** The source data behind the Figure 5.

**File name:** Supplementary Data 19

**Description:** The source data behind the Figure 6.

**File name:** Supplementary Data 20

**Description:** The source data behind the Supplementary Figure 20.
